# Quantitative mass spectrometry analysis reveals a panel of nine proteins as diagnostic markers for colon adenocarcinomas

**DOI:** 10.18632/oncotarget.24418

**Published:** 2018-02-05

**Authors:** Apurva Atak, Samiksha Khurana, Kishore Gollapalli, Panga Jaipal Reddy, Roei Levy, Stav Ben-Salmon, Dror Hollander, Maya Donyo, Anke Heit, Agnes Hotz-Wagenblatt, Hadas Biran, Roded Sharan, Shailendra Rane, Ashutosh Shelar, Gil Ast, Sanjeeva Srivastava

**Affiliations:** ^1^ Proteomics Laboratory, Department of Biosciences and Bioengineering, Indian Institute of Technology Bombay, Powai, Mumbai 400076, India; ^2^ Department of Human Molecular Genetics and Biochemistry, Sackler Medical School, Tel Aviv University, Tel Aviv 69978, Israel; ^3^ Bioinformatics Group, Genomics and Proteomics Core Facility (GPCF), German Cancer Research Center (DKFZ), Heidelberg 69120, Germany; ^4^ Blavatnik School of Computer Science, Tel Aviv University, Tel Aviv 69978, Israel; ^5^ Shimadzu Analytical (India) Pvt. Ltd, 1A/B, Rushabh Chambers, Makwana Road, Marol, Andheri (E), Mumbai 400059, India

**Keywords:** colon adenocarcinoma, proteomics, biomarkers, tissue, iTRAQ

## Abstract

Adenocarcinomas are cancers originating from the gland forming cells of the colon and rectal lining, and are known to be the most common type of colorectal cancers. The current diagnosis strategies for colorectal cancers include biopsy, laboratory tests, and colonoscopy which are time consuming. Identification of protein biomarkers could aid in the detection of colon adenocarcinomas (CACs). In this study, tissue proteome of colon adenocarcinomas (*n* = 11) was compared with the matched control specimens (*n* = 11) using isobaric tags for relative and absolute quantitation (iTRAQ) based liquid chromatography-mass spectrometry (LC-MS/MS) approach. A list of 285 significantly altered proteins was identified in colon adenocarcinomas as compared to its matched controls, which are associated with growth and malignancy of the tumors. Protein interaction analysis revealed the association of altered proteins in colon adenocarcinomas with various transcription factors and their targets. A panel of nine proteins was validated using multiple reaction monitoring (MRM). Additionally, S100A9 was also validated using immunoblotting. The identified panel of proteins may serve as potential biomarkers and thereby aid in the detection of colon adenocarcinomas.

## INTRODUCTION

Colorectal cancer is one of the most common cancers detected worldwide, with around 1.4 million cases diagnosed in 2012 [1]. Adenocarcinomas are the most common type of colon cancers, whereas small fraction of lymphoma and squamous cell carcinoma cases can also be found. Removal of high-risk adenomas and tumors at early stage of colorectal cancer (CRC) can avert the onset/progression into higher grades of cancer. Early diagnosis shows a 5-year survival rate among 90% patients with localized tumors, whereas it drops down to less than 10% when the CRC is metastasized to other organs [2, 3]. This makes the implementation of screening methods aimed at early detection paramount for reducing mortality rate.

At present, the CRC screening methods range from a simple stool test to highly invasive colonoscopy [4]. Major limitations in the early diagnosis of colon cancers are low sensitivity of existing diagnostic methods, cost and access to the modern imaging techniques like computed tomography (CT) and magnetic resonance colonography. There is a need for more specific and sensitive molecular tests than the currently available diagnostic methods [5]. The ideal approach would be to discover and validate genetic and/or protein biomarkers which could serve as an efficient tool for cancer screening, diagnosis of recurrences and prognosis.

Identification of novel biomarkers for colorectal cancer with both- prognostic and predictive value, not only aids in improving our understanding of the disease but also helps in the treatment of disease at an early stage, and management at an advanced stage. This not only offers new tools to estimate disease outcome but also helps in predicting overall patient outcome. Over the years, several tissue and serum specific biomarkers have been identified. Histological evaluation of tissue samples from gastrointestinal neoplasms are commonly carried out by immunohistochemistry (IHC) analysis of target proteins against CDX2 [6], Mucins (MUC2) [7], Special AT-rich sequence binding protein 2 (SATB2) [8], Cytokeratins [9, 10], β-catenin [11], Carcinoembryonic antigen (CEA) [12], and Cadherin 17 [13, 14]. Furthermore, protein targets like CDX2 and SATB2 have also been linked to be important prognostic markers for colorectal cancer patients. For instance, loss of CDX2 expression has been commonly associated with proximal location, advanced T, N, M, overall stage; and has been considered as an independent poor prognostic marker of overall survival and progression-free survival [15]. Besides performing tests on faeces, several tests against tumor associated antigens as potential biomarkers in early diagnosis have also been identified. In this regard, Serum CEA and Ca 19.9 have been commonly used as classical tumor markers in colorectal cancer patients. Several studies support the prognostic value of high pre-operative CEA levels as a surrogate for useful investigation [16–19]. Additionally, besides being more cost effective than radiological detection methods, CEA is also the most frequent determinant of recurrence in asymptomatic patients [20]. An increase in Ca 19.9 levels, an alternate marker for colorectal cancers, has been significantly associated with higher frequency of metastasis and lower survival rates, thereby making it prognostic marker for adverse colorectal cancer patients [20]. Hence, there is a need of other novel biomarkers which could improve patient outcome.

This study aimed at investigating proteomic alterations in human CAC tissues from a heterogeneous patient population using isobaric tagging for relative and absolute quantification (iTRAQ) to identify potential biomarkers. The identified panel of proteins was validated by Multiple Reaction Monitoring (MRM) assays. Further bioinformatic analysis aided in understanding the biological networks associated with the disease pathogenesis.

## RESULTS

### Identification of differentially expressed proteins in iTRAQ labelled colon adenocarcinoma tissue lysates

**Figure 1:**
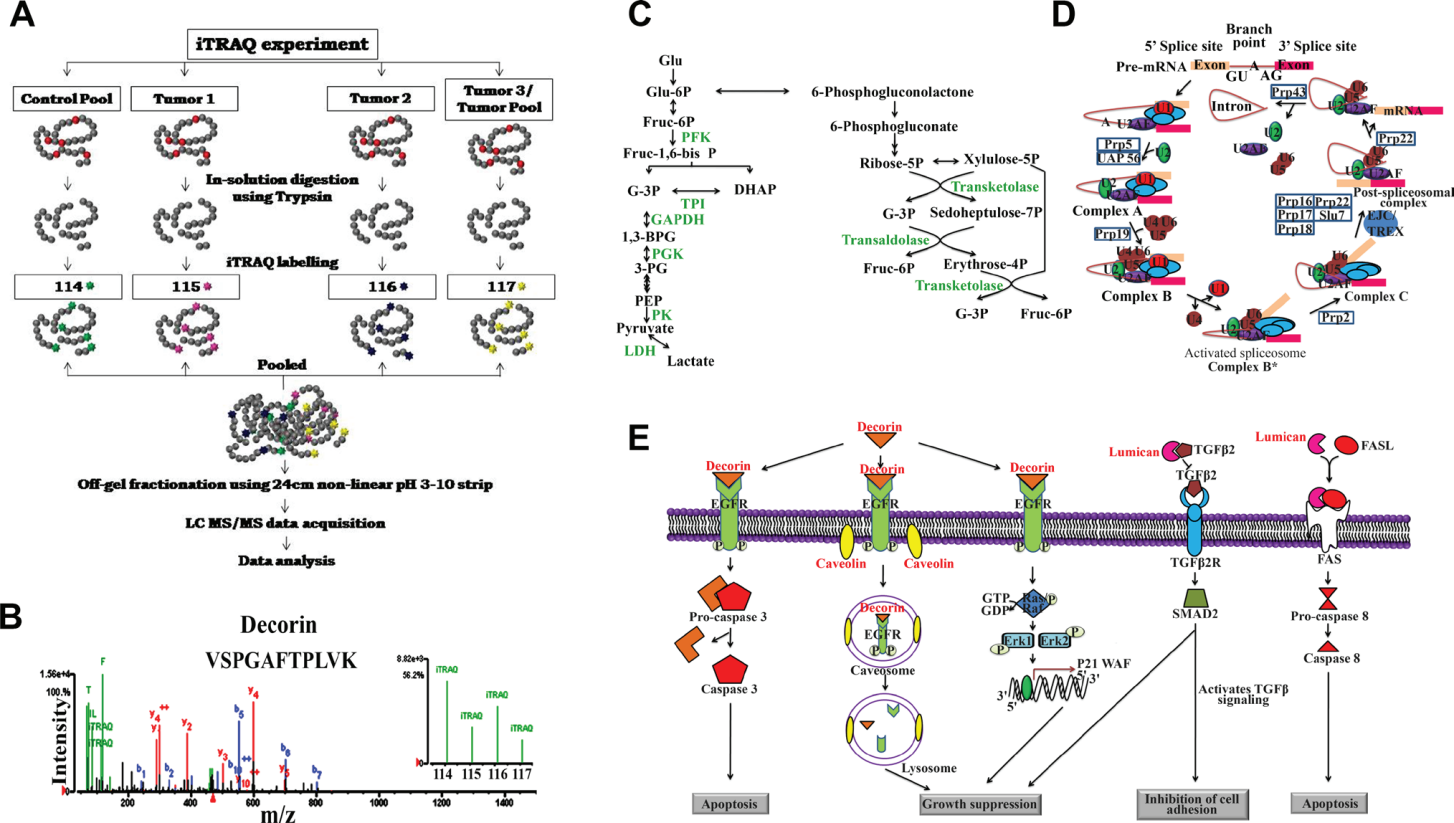
Quantitative proteomic analysis of human colon adenocarcinomas and the significantly altered metabolic pathway (**A**) Schematic representation of the iTRAQ labelling strategy and the mass spectrometry based approach used for the identification of proteomic alterations in colon adenocarcinoma tissue lysates. (**B**) Representative spectra for proteins identified to be altered in colon adenocarcinoma tissue lysates (Decorin spectra was used for representation). Significantly altered proteins identified from iTRAQ based mass spectrometry experiments were subjected to pathway analysis using Database for Annotation, Visualization and Integrated Discovery (DAVID) Functional Annotation Bioinformatics Analysis v6.8, an online tool, which revealed (**C**) Glycolysis (**D**) Spliceosome mediated splicing and (**E**) Proteoglycan mediated signaling to be majorly altered in colon adenocarcinomas.

The CAC tumor and their matched control tissue lysates were digested using trypsin followed by iTRAQ labelling. Each of the four 4-plex reactions had a pool of all the control tissue lysates (*n* = 11) labelled with the iTRAQ label 114 and three of the individual tumor tissue lysates labelled using 115, 116 and 117 iTRAQ labels. Additionally, one of the four sets consisted of the pool of all the tumor tissue lysates (*n* = 11) labelled with the iTRAQ label 117. The iTRAQ labelled samples were pooled and subjected to off-gel fractionation followed by LC-MS/MS. The LC-MS/MS data was analyzed independently by Spectrum Mill (SM). 514, 354, 569 and 581 proteins were identified with 2 or more unique peptides in set 1, set 2, set 3 and set 4 respectively, of the iTRAQ labelled samples from SM analysis. Of these, 401 proteins were common in at least 3 out of 4 sets (Supplementary Table 1A). Further, 285 proteins passed an average fold change cut-off of 1.5 and 147 proteins followed the same trend of dysregulation in at least 7 of the 11 patients (64% population). A representative mass spectrum for Decorin, which is down-regulated in CACs, has been shown in Figure 1.

In order to increase our confidence of the proteins identified using Spectrum Mill, the raw files from the LC-MS/MS run were also analysed using the Trans-Proteomice Pipeline (TPP) software [21]. 944, 725, 1040 and 1099 proteins were identified with 2 or more peptides in set 1, set 2, set 3 and set 4, respectively from TPP. 808 proteins were common in at least 3 out of the 4 sets (Supplementary Table 1B), of which, 510 proteins passed an average fold change cut-off of 1.5; and 232 proteins followed the same trend of dysregulation in at least 7 of the 11 patients.

These 232 proteins were compared with the 147 proteins from Spectrum Mill dataset, which passed all the criteria, and a list of 94 proteins was generated which were common between Spectrum Mill and Trans-Proteomic Pipeline dataset (Supplementary Table 1C). This list of 94 significantly altered proteins was compared with the literature on CACs and CRCs reported by Jankova *et al.* and Wisniewski *et al.* to identify the overlap [22, 23] (Supplementary Table 1D). Additionally, comparison of results from current study with the data from Zhang *et al.* [24] at CPTAC data portal (https://cptac-data-portal.georgetown.edu/cptacPublic/) revealed 34 common proteins exhibiting same trends in both the data sets (Table 1). Peptide transitions for these 34 proteins were studied using TPP, SM, SRM atlas and Skyline. The list of 94 proteins identified to be significantly dysregulated from this study was compared for expression levels of these proteins in colorectal tumor tissues available at the HPA. Protein expression was listed in HPA as High, Medium, and Low or Not detected. Proteins showing medium or high expression in ≥50% population were considered to be significantly up-regulated. With this comparison, 18 of the 94 proteins were found to be up-regulated in SM as well as TPP analysis, CPTAC and HPA data. 3 of these proteins were included in validation by MRM. Additionally, another 36 proteins were found to be altered with same trend of dysregulation in SM and TPP analysis as that from the HPA data.

**Table 1:** List of differentially expressed proteins obtained from TPP and spectrum mill analysis and following the same trend in the literature (Zhang *et al.*, 2014).

Protein details			Average fold change (Tumor/Normal)			*p-value*
Accession number	Protein name	Gene name	TPP	Spectrum mill	CPTAC	Computed from TPP data
P06702^*^	Protein S100-A9	S100A9	3.2	3	7.9	0.00034
P05109^*^	Protein S100-A8	S100A8	3.2	2.8	6.4	0.00088
P08195	4F2 cell-surface antigen heavy chain	SLC3A2	3	2.8	1.6	0.00100
P61626	Lysozyme C	LYZ	2.8	2.8	4.1	0.00143
P05164	Myeloperoxidase	MPO	2.2	2.3	2.3	0.00145
P11940	Polyadenylate-binding protein 1	PABPC1	2.2	2.4	1.5	0.00032
P31949^*^	Protein S100-A11	S100A11	2.1	2.3	1.7	0.00029
P78527	DNA-dependent protein kinase catalytic subunit	PRKDC	2	2.9	39.9	0.00023
Q14103	Heterogeneous nuclear ribonucleoprotein D0	HNRNPD	2	2	23.9	0.00581
Q12906	Interleukin enhancer-binding factor 3	ILF3	2	2	13.2	0.00005
P49327	Fatty acid synthase	FASN	2	2.1	1.6	0.00145
Q07955	Serine/arginine-rich splicing factor 1	SRSF1	1.9	1.8	10.8	0.00380
P00338^*^	L-lactate dehydrogenase A chain	LDHA	1.9	1.9	3.5	0.00152
P50454	Serpin H1	SERPINH1	1.9	2.2	2.8	0.00010
P61247	40S ribosomal protein S3a	RPS3A	1.9	2	1.8	0.00093
O60506	Heterogeneous nuclear ribonucleoprotein Q	SYNCRIP	1.9	1.7	1.8	0.00529
P40121	Macrophage-capping protein	CAPG	1.8	1.9	9.6	0.00054
P07108	Acyl-CoA-binding protein	DBI	1.8	2	5.8	0.00622
P60842	Eukaryotic initiation factor 4A-I	EIF4A1	1.8	2	3.1	0.00476
P51858^*^	Hepatoma-derived growth factor	HDGF	1.8	1.9	2.9	0.00007
O14980	Exportin-1	XPO1	1.8	2.2	2	0.01283
P29401	Transketolase	TKT	1.7	2	68	0.00066
P14618^*^	Pyruvate kinase	PKM	1.7	1.6	34.5	0.00038
P06748	Nucleophosmin	NPM1	1.6	2.1	42.4	0.00697
P26599	Polypyrimidine tract-binding protein 1	PTBP1	1.6	1.6	33.6	0.00120
P78417	Glutathione S-transferase omega-1	GSTO1	1.6	2.1	26.2	0.00160
P51884^*^	Lumican	LUM	0.6	0.6	0.6	0.00013
P12277	Creatine kinase B-type	CKB	0.6	0.7	0.2	0.01292
P07585	Decorin	DCN	0.5	0.5	0.6	0.00001
Q9NZN4^*^	EH domain-containing protein 2	EHD2	0.5	0.6	0.5	0.02325
Q16853^*^	Membrane primary amine oxidase	AOC3	0.5	0.5	0.4	0.01800
P00918	Carbonic anhydrase 2	CA2	0.5	0.5	0.1	0.00720
P00915	Carbonic anhydrase 1	CA1	0.5	0.7	0.1	0.01223
O14558	Heat shock protein beta-6	HSPB6	0.4	0.4	0.6	0.01205

^*^ Proteins validated using MRM.

### Pathways analysis using dysregulated proteins from spectrum mill and Trans-Proteomic Pipeline dataset

Significantly dysregulated proteins showing the same trend in at least 7 out of 11 patients identified using TPP and SM software were combined to identify 285 unique proteins. These proteins were submitted as input list to identify the alterations in the underlying pathways using the Database for Annotation, Visualization and Integrated Discovery (DAVID) Functional Annotation Bioinformatics Analysis v6.8 [25, 26]. Most of the up-regulated proteins were associated with glycolysis, pentose phosphate pathway, pyruvate metabolism, protein processing, antigen processing and presentation, spliceosome, proteosome mediated protein degradation and ribosomal proteins while the down-regulated proteins were associated with focal adhesions and proteoglycans in cancer (Supplementary Table 1E; Figure 1).

### Immunoblotting for S100A9 protein expression in colon adenocarcinoma

**Figure 2:**
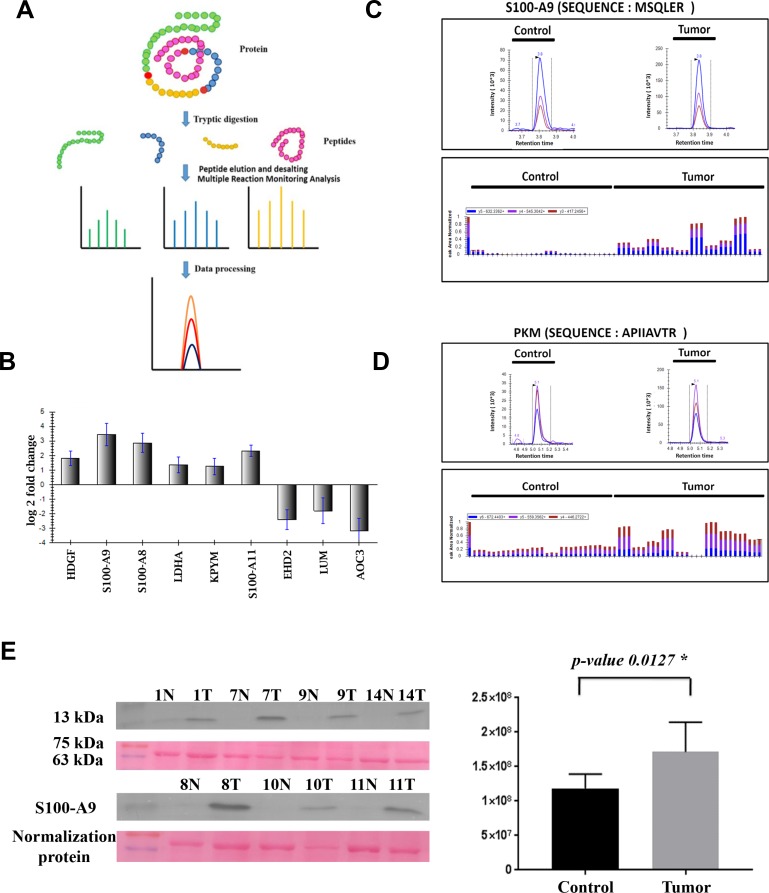
MRM and immunoblotting based validation of the top dysregulated proteins identified in the present study (**A**) Schematic representation of the MRM workflow. (**B**) Graphical representation of the Log2 fold change of differentially expressed proteins identified using MRM. (**C** and **D**) Representative chromatograms depicting transitions for peptides specific to S100-A9 and Pyruvate kinase proteins, respectively. (**E**) Validation of expression levels of S100-A9 protein in colon adenocarcinoma tissue lysates and their matched controls using immunoblotting.

Tumor and control tissue levels of S100A9 protein were validated using immunoblotting method. House-keeping proteins such as beta actin and GAPDH were found to be differentially expressed from the shotgun proteomics study. Therefore, S100A9 expression levels were normalised against a uniform band (approx. 70 kDa) from the ponceau image obtained post transfer of the samples on the PVDF membrane. After normalisation, S100A9 was found to be up-regulated in all the tumor samples compared to its controls with *p* value < 0.05, which was consistent with the iTRAQ analysis (Figure 2).

### Validation of differentially expressed proteins identified from the iTRAQ approach in colon adenocarcinoma tissue lysates using multiple reaction monitoring

Peptide sequences of 13 potential protein biomarkers were taken forward for MRM-based validation in 10 of the 11 CAC tissue lysates included in iTRAQ study. Total 46 peptides were shortlisted for the nine proteins, of which only 27 peptides corresponding to 9 proteins were detected in the tissue lysates and their retention times are given in Supplementary Table 1F.

MS stats R-package was used to perform statistical analysis to identify and quantify the significantly dysregulated proteins. Proteins showing fold change ≥1.5 and *p*-value of ≤ 0.05 were categorised as significantly altered proteins. All nine proteins showed significant dysregulation in CAC versus their matched control (Table 1). HDGF, LDHA, PKM, S100A8, S100A9 and S100A11 were found to be significantly up-regulated and EHD2, LUM and AOC3 were found to be significantly down-regulated in CAC versus their matched control (Figure 2). The MRM measurements of all nine target proteins were found to be consistent with the data obtained from the iTRAQ based proteomics experiment, which could be potential markers for diagnosing CACs.

### Finding associated protein-protein interaction on a background network

**Figure 3:**
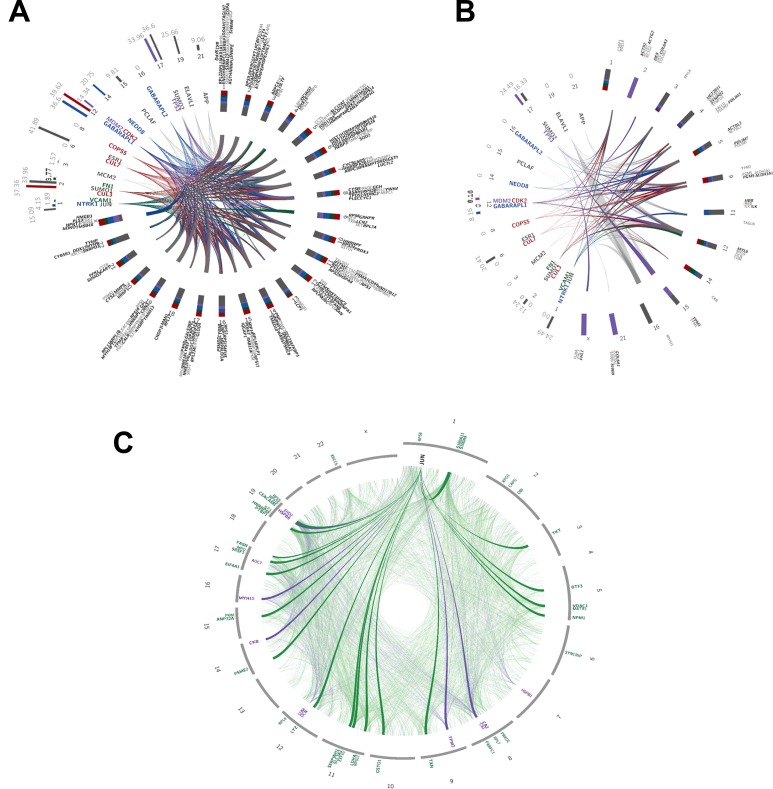
Interaction network analysis Plot depicting the relationships and interactions between the (**A**) up-regulated (fold change from 1.5 and over) and (**B**) down-regulated (fold change from -1.5 and under) proteins identified using SM and TPP. Color-codes represent functions manually retrieved from the literature: grey = DNA and RNA regulation; green = immunological system functions; red = ubiquitin tagging; purple = cell cycle and p53 regulation; blue = neuronal regulation. (A) Inner circle lines connect between the 20 most common 1st-degree neighboring proteins (∼9 to 12 hours; colored names) discovered by propagation analysis, and 241 up-regulated proteins (∼12 to 9 hours; grey hued names) identified by SM and TPP. Italic-bold names represent proteins up-regulated according to SM; bold according to TPP; regular according to both. Colored bar-plots (∼9 to 12 hours) depict the relative active networks that the protein is part of according to MetaCore analysis, and their associated numbers represent the percentages of actual networks (from the total number of input networks). Colored stacked bar-plots (∼12 to 9 hours) represent the relative contribution of each function to proteins gathered on the same chromosome. Integers are the chromosome numbers. (B) Inner circle lines connect between the 20 most common 1st-degree neighboring proteins (∼7 to 11 hours; colored names) discovered by propagation analysis, and 41 down-regulated proteins (∼12 to 7 hours; grey hued names) identified by SM and TPP. Italic-bold names represent proteins down-regulated according to SM; bold according to TPP; regular according to both. Colored bar-plots (∼7 to 11 hours) depict the relative active networks that the protein is part of according to MetaCore analysis, and their associated numbers represent the percentages of actual networks (from the total number of input networks). Colored stacked bar-plots (∼12 to 7 hours) represent the relative contribution of each function to proteins gathered on the same chromosome. Integers are the chromosome numbers. (**C**) JUN interaction network. Faded links represent interactions between the topmost 1st degree neighbors (identified via network propagation algorithm) and the prior 94 genes further filtered to include only genes with evidence from SM, TPP, and CPTAC. Bold links are prior genes linked directly to JUN; specifically, 21/44 up-regulated genes (fold change of 1.5 and over) and 5/11 down-regulated genes (fold change of -1.5 and under) are connected directly to JUN. Outer circle labels are the chromosomes’ numbers; gene names are relatively positioned along the chromosomes.

We adapted the propagation procedure described in Vanunu *et al.* 2010 [27] to identify additional disease related proteins. Briefly, a list of prior-knowledge proteins was diffused over a background network-here, the HIPPIE protein-protein interaction (PPI) network [28] – and a pre-defined cut-off based on relatedness score was used to choose the strongest first degree neighbours. We separately propagated the up- and down-regulated proteins as they appear in the list of 285 proteins discovered by TPP and/or SM, as discussed above (Figure 3). To find the strongest common top 20 proteins for this list, we pooled together the up and down-regulated proteins and obtained the 50 strongest PPI interactions. We then propagated each protein separately and obtained the 50 strongest PPI interactions. Then, we intersected the resulting lists and picked the top 20 strongest interactions. Figures 3A and 3B show the interactions - as links, color coded by common functions, which we manually derived from the literature-between the 20 post-proteins (colored names) and the 285 prior-proteins (grey hues). In the 20 post-proteins list, some notable members like NTRK1 (common to 163/285 proteins; see Supplementary Table 1G for a list of scores and number of common proteins for each of the 20 proteins), SUMO2 and SUMO1 (common to 160 and 111/285 proteins, respectively), JUN (common to 143/285 proteins), and TP53 (common to 91/285 prior proteins) are associated with tumor pathobiology.

## DISCUSSION

CAC is one of the major causes of cancer related deaths. Cancers are developed due to the manifestation of mutations in various proto-oncogenes and oncogenes, which can be identified using genomics. However, phenotype of the cells is majorly decided by the protein expression, their modifications and interaction. Hence, studying the cancers at proteome level would yield plausible markers for the disease diagnosis/prognosis/therapy [29].

Previously, Fung *et al.* reported a three protein panel as blood-based biomarkers for the diagnosis of colorectal cancers. The panel consists of Insulin-like growth factor-binding protein 2 (IGFBP2), Dickkopf-related protein 3 (DKK3) and Pyruvate kinase (PKM2), which could detect the CRCs with a sensitivity of 73% and specificity of 95% [30]. Later in the year of 2016, Jones *et al.* identified a panel of 13 proteins (15 transitions), from the plasma specimens of CRC patients using multiple reaction monitoring (MRM) based mass spectrometry approach, which could discriminate the CRCs from controls with a sensitivity of 87% and specificity of 81% [31]. In another study, Blume *et al.* identified the plasma proteomic alterations in colorectal cancers and validated a panel of eight proteins using ELISA as diagnostic markers for CRCs, while another panel consisting of four proteins was proposed as a biomarker panel for advanced adenomas [32]. In the current study, iTRAQ based tissue proteomic analysis of CAC and matched controls revealed alteration in proteins involved in various metabolic and biological processes, which affect the growth and development of tumors. Comparison of our current study results with the existing mRNA /protein expression data would help to identify protein markers with high confidence. In this light, protein expression data from our study was compared with IHC data from HPA and label-free mass spectrometry data from Clinical Proteomic Tumor Analysis Consortium Components (CPTAC) for CRCs and most of the identified proteins in the present study are in sync with the HPA and CPTAC studies for CRCs (Supplementary Table 1H).

In order to maintain high proliferation rates, tumor cells require large amounts of ATP and precursors for the synthesis of nucleic acids. Tumor cells often exhibit increased flux of glucose into glycolysis to generate ample amounts of ATP. To continue the glycolysis process without interruption, cancer cells increase the expression of lactate dehydrogenase, which converts the end product of glycolysis (pyruvate) to lactate [33]. The H+ ions generated in this process decreases the pH of the tumor microenvironment, thereby making way for the tumor cells to grow by killing the adjacent /nearby cells. This acidic environment facilitates tumor growth, invasion and metastasis in malignant cancers [34]. In the present study, most of the glycolysis and pentose phosphate pathway proteins were found to be up-regulated. Increased levels of these proteins might be useful in increasing flux of glucose into glycolysis and pentose phosphate pathway, thus aiding in tumor growth and malignancy. Pyruvate kinase (PKM) and lactate dehydrogenase A chain, which are associated with the glucose metabolism were found to be significantly increased in the CACs from the present study. Pyruvate kinase is the last enzyme in the glycolysis pathway, which converts phosphoenol pyruvate to pyruvate and an ATP molecule is released in this process (2 ATP molecules per one glucose molecule) [35]. Thus PKM supports the tumor growth by providing ATP. PKM2 is a variant of pyruvate kinase, produced by the alternative splicing of PKM pre-mRNA. PKM2 expression was found to be increased in various malignancies [36]. Lactate dehydrogenase converts pyruvate to lactate in anaerobic conditions. However tumor cells can produce excess amount of lactate under normoxic conditions. Increased levels of lactate induces the secretion of hyaluronan, thus promoting the metastasis of tumors [37]. Hypoxia inducing factor and c-Myc can induce the expression of lactate dehydrogenase A (LDHA) [38, 39]. LDHA silencing in HCT116 cancer cell line resulted in apoptosis of the cancer cells, which indicates the significance of LDHA in tumor growth and progression [40]. Thus, increased expression of PKM and LDHA in CACs might be resulting in the tumor cell survival by preventing the entry of glycolytic pathway into feedback inhibition loop by the end product of the pathway.

Hepatoma-derived growth factor (HDGF), a secreted protein which was first purified from the culture medium of Huh-7 hepatoma cells, was found to be up-regulated in various cancers like hepatocellular carcinoma, colorectal, pancreatic and non-small cell lung cancers [41–44]. Expression of HDGF altered with changes in the expression of β-catenin and vice versa. HDGF knock-down in HCT116 cell lines resulted in the decreased cell proliferation by arresting the cells in G1 phase, finally leading to apoptosis. Besides the tumor growth, HDGF knock-down decreased the tumor cell migration and invasion in HCT116 cells [42]. Increased expression of HDGF was reported as a poor prognostic marker in various cancers like hepatocellular carcinoma, gastric and lung cancers [44–46]. HDGF was found to be up-regulated in CACs from the current study. HDGF could be potential target in CAC patients exhibiting increased tumor levels of this protein to improve the clinical outcomes.

Ribosomes are protein synthesizing machinery in the living cells, which are composed of rRNAs and ribosomal proteins (RPs). Besides maintaining the structural integrity of ribosomes by stabilizing folding of rRNAs, RPs are also involved in protein synthesis [47]. RPs are known to play extra ribosomal functions, which includes RNA splicing, DNA repair, cell survival, proto-oncogene regulation, tumor malignancy and differentiation [48]. Our study revealed that the colon tumor tissue exhibit increased expression of various ribosomal proteins associated with the smaller and larger subunits. RPs are reported to be dysregulated in cancers and various genetic disorders [49–54]. Kasai *et al.* identified increased expression of RPL7 using IHC in human CRCs, which has an endocrine function [55]. Proteins associated with tumor development and suppression like MYC and PTEN play a major role in the turnover of ribosomes by regulating the expression of RPs and S6K activity, respectively [47]. This indicates that the RPs differential expression is associated with tumorigenesis. Over-expression of RPS3a in NIH-3T3 cells resulted in the malignant transformation of the cells by inducing the expression of various anti-apoptotic proteins [56]. Increased expression level of RPS13 in gastric cancer cells protects the cells from drug induced apoptosis and enhances cell proliferation rates [57, 58]. RPS6, RPS19 and RPS25 helps in the transcription initiation of viral genes [59] while RPL5 and RPL11 acts as tumor suppressors by degrading the mRNA of c-*myc,* through RNA induced silencing complex (RISC) [60]. Thus RPs act as tumor suppressors and also promotes the tumor growth under different circumstances.

The proteins which showed down-regulation from this study were found to be involved in pathways related to focal adhesion and proteoglycans. Focal adhesion complexes are required for the cells to migrate from one region of the tissue to other. Filamin A and C, and Caveolin 1 were found to be down-regulated by at least 2 folds while myosin regulatory light polypeptide 9 (MYL9) was found to be 4.5 fold down-regulated. The filamin family proteins acts as actin-binding proteins and play a role in compositing the actin cytoskeleton and associated cell surface adhesion proteins [61]. Filamin A has also been suggested to suppress cancer cell invasion and migration by regulating focal adhesion [62]. While the role of myosin regulatory light polypeptide 9 (MYL9) remains poorly documented in terms of its functional association in human cancers including colon carcinoma, a recent report by Yan *et al.*, suggest lower expressions of MYL9 was associated with lower median survival rates in colon cancer patients [63]. Furthermore, the role of Caveolin 1 has been reported in multiple cancers towards both tumor progression and tumor suppression. However, in case of colon cancers, the association of Caveolin 1 expression with tumor progression has been variably reported [64–67]. Further studies should be carried out for understanding the decreased expression of focal adhesion proteins role in tumor growth and progression.

Proteoglycans are heavily glycosylated key molecules regulating the activity of several signalling pathways and the interactions between a cell and its microenvironment. Their polyhedic nature allows for their ability to interact with both ligands and receptors to regulate neoplastic growth, progression, and neovascularisation [68]. Over the years, the role of proteoglycans in cancer has been studied extensively. Heparan sulphate proteoglycans (HSPGs) are one such example that has a defined role as a pro-angiogenic factor by binding growth factors and exhibiting them to their cognate receptors [68, 69]. Decorin is known to be a tumor suppressing gene involved in colon cancer and metastasis [70]. A large number of studies are focussing on considering Decorin as a molecule of high therapeutic potential in case of colon cancer [70, 71]. Decorin deficient mice showed increased rate of tumor formation and poor cell differentiation [72]. It is found to be down-regulated in colorectal tumor tissues as compared to normal colon tissues [72, 73]. The same trend was observed in this study as well wherein the expression of Decorin was 2-fold down-regulated in colon tumor tissues. Lumican is a leucine rich proteoglycan present in the extracellular matrix. This protein has been reported to be up-regulated in breast, colorectal, pancreatic, uterine, cervical, and prostate cancers [74–78]. Ectopic expression of lumican in the prostate cancer cell lines showed, decreased cell proliferation, migration and invasion [78]. With increase in the stage of the breast tumors, the expression of lumican was decreased [79]. Decreased expression of lumican and decorin reported to be poor prognostic markers for node-negative invasive breast cancers [80]. In the present study, proteoglycans like decorin and lumican were down-regulated in CACs.

Another major group of proteins which were found to be dysregulated from this study includes the S100 protein family which consists of 21 distinct proteins sharing very high percentage of sequence and structural similarity. These proteins consist of two calcium binding motifs which are connected by a hinge/loop called target binding domain. These proteins act as Ca^2+^ sensors and regulate the intracellular calcium levels by sequestering them. Binding of calcium ions to the S100 proteins induces conformational change, which exposes the hydrophobic cleft for target binding to the hinge/target binding domain. Binding of the target to S100 protein enhances its affinity for calcium binding by 5–300 folds. These intracellular S100 proteins bind to various target proteins and regulate their activity. Extracellular S100 proteins bind to various cell surface receptors and facilitate intercellular communication. In most of the human cancers S100 family proteins are up-regulated. However, expression levels of these proteins also vary in different cancers based on the stage or subtype.

S100A8/A9 are associated with various biological functions including immune suppression, angiogenesis and pre-metastatic niche formation. Tumor cells induce production of S100A9 protein in the myeloid precursors, which prevents the differentiation of myeloid cells to macrophages and dendritic cells resulting in the development of myeloid-derived suppressor cells (MDSCs) in the tumor microenvironment. In this way tumor cells evade from the host immune system [81]. S100A8/A9 homo/heterodimers can interact with the tumor cell surface receptors like RAGE and TLR4 resulting in the activation of MAPK and NF-κB signalling pathways, which promotes tumor growth and invasion [81, 82]. Various growth factors released from the primary tumor induce the expression of S100A8/A9 and creates a pro-inflammatory milieu, which facilitates the metastasis at the target site [83, 84].

Protein interaction analysis revealed the association of altered proteins in CACs with various transcriptional factors and their targets. NTRK1 (Neurotrophic Receptor Tyrosine Kinase 1; encodes TrkA) was previously reported to impose oncogenic activity in lung cancer cells by fusing with CD74 and, independently, with MPRIP [85]. Further, NTRK1 rearrangements were associated with metastatic CRC in clinical patients expressing TrkA [86]. SUMO1 and TP53 were found to be interrelated in colon cancer patients; Zhang *et al.* [87] have found SUMO1 to be significantly over-expressed in colon cancer patients, and that this over-expression leads to accumulation of P53 which in turn promotes colon tumor formation. JUN is a transcription factor that recognizes and binds to the enhancer heptamer motif 5-TGA[CG]TCA-3. It promotes activity of NR5A1 when phosphorylated by HIPK3; this leads to increased steroidogenic gene expression upon cAMP signaling pathway stimulation. It is also involved in activated KRAS-mediated transcriptional activation of USP28 and binds to the USP28 promoter in colorectal cancer cells [88]. A study by Suto *et al.* in 2004 also suggested that a dominant mutant of JUN inhibits the activity of the transcription factor AP-1 and is thus helpful for relieving colon cancer tumorigenesis [89]. Remarkably, JUN was also found to be the strongest and the only common protein after propagating the list of 94 proteins common to TPP and SM (Figure 3C).

The major strength of the present study lies in the usage of tumor and matched control specimens. This eliminates the person-to-person variation, due to their genetic makeup, lifestyle and environmental conditions. Despite using tumor specimens from the patients with different ethnic origin, the changes in the protein expression were consistent among the CAC patients. Due to the unavailability of large number of clinical sample, both discovery and validation were performed on small sample cohort, which is a major limitation of the present study. Synthetic peptide usage in the MRM experiments aids in the accurate quantification of peptides. MRM assays reported in the study were performed without synthetic peptides due to their unavailability; hence, relative abundance of the peptides for the significantly altered proteins was measured, instead of absolute quantification, which is the second limitation of the study. Significantly altered proteins identified from the present study can be further validated on larger patient cohorts and the significance of the altered proteins in the tumor growth/progression can be studied using cell line and animal models.

In conclusion, we have identified a few significantly altered proteins like ribosomal proteins, spliceosomal proteins, proteoglycans and various metabolic pathway enzymes which play a key role in the tumor development of CAC. Various transcriptional factors were found to be associated with the altered proteins. A panel of nine proteins, which were validated as differentially expressed proteins could be potential diagnostic markers for CACs. Further, validation of these proteins on larger patient cohorts would be useful in translating the bench side protein markers to bedside.

## MATERIALS AND METHODS

### Tissue lysates and ethics statement

CAC tissue lysates (*n* = 11) and their matched control tissue lysates (colon tissue specimens of same patient from a region distant from the site of tumor) were received from Department of Human Molecular Genetics & Biochemistry, Sackler Medical School, Tel Aviv University, Israel, where the tissue samples were mechanically homogenized in a hypotonic lysis buffer containing 50 mM Tris-HCl, pH 7.5, 1% NP40, 150 mM NaCl, 0.1% SDS, 0.5% deoxycholic acid, 1 mM EDTA supplied with protease inhibitor (Roche) and phosphatase inhibitor cocktails I and II (Sigma) [90]. Protein concentration was measured using Bio-Rad Protein Assay by iMARK Microplate reader (Bio-Rad). The sample details have been provided in Supplementary Table 1I. The further experiments were performed at Prof. Srivastava’s laboratory at Indian Institute of Technology, Bombay (IIT-B) after receiving the approval from the institute ethics committee as per the proposal number IITB-IEC/2016/029.

### Buffer exchange and in-solution protein digestion

After quantification using Bradford’s assay, 250 µg of protein sample from tumor tissue lysates (*n* = 11) and their matched control tissue lysates (*n* = 11) were buffer exchanged with 0.5M Triethyl ammonium bicarbonate (TEAB) buffer followed by in-solution digestion of 75 µg of protein as described previously [91].

### iTRAQ 4-plex labelling and off-gel fractionation

The iTRAQ 4-plex labelling of the trypsin digested protein samples was done according to the manufacturer’s instructions and the labelling strategy is illustrated in Figure 1A. Further, off-gel fractionation (3100 off-gel fractionator, Agilent) was performed on a high resolution (24 cm length) non-linear 3–10 pH range immobiline pH gradient (IPG) strips. The fractions were vacuum concentrated and desalted using C18 material packed ZipTips prior to LC-MS/MS data acquisition for the peptide samples.

### LC-MS/MS data acquisition and analysis using spectrum mill

Mass spectrometry data acquisition was performed using Agilent 6550 iFunnel Q-TOF (Agilent Technologies) mass spectrometer equipped with chipcube interfaced nanoflow LC system (Agilent Technologies). The fractionated peptides were enriched on C18 enrichment column. The enriched peptides were separated on a 75 µm x 150 mm analytical/separation column of polaris-high resolution-chip using a gradient mobile phase of 0.1% formic acid solution (Solvent A) and 90% acetonitrile (Solvent B) at a flow rate of 0.5 µl/min. The gradient method used over a period of 100 min for separation of peptides was: 0–2 min, 0–03% solvent B; 2–70 min, 03–35% solvent B; 70–75 min, 35–45% solvent B; 75–85 min, 45–95% solvent B; 85–88 min, 95–03% solvent B. From 88–100 min, the enrichment and analytical columns were equilibrated with 03% solvent B and 97% solvent A, prior to the next peptide fraction run on the LC-MS system. Other parameters include nitrogen gas maintained at 250°C with a 9 L/min flow rate; mass range of 300–3000 (m/z) in MS with a MS Scan rate of 8 spectra/sec; mass range of 50–3000 (m/z) in MS/MS with a MS/MS scan rate of 4 spectra/sec. From MS, top 12 intense peptides having a charge ≥ 2 were selected for MS/MS and the mass spectral data was acquired in the centroid mode. The mass spectrometry data was analyzed using Spectrum Mill MS Proteomics Workbench software (Agilent Technologies); searched against SwissProt database using *Homo sapiens* as taxonomy; iTRAQ (N-term, K) & carbamidomethylation (C) as fixed modifications and oxidation of methionine as a variable modification with a precursor and product mass tolerance of 20 and 50 ppm, respectively. The mass spectrometry data files for the shotgun proteomics experiments have been deposited to the ProteomeXchange Consortium via the PRIDE [92] partner repository with the dataset identifier PXD006509.

### Mass spectrometry data analysis using Trans-Proteomic Pipeline

The mass spectrometry raw files were converted into .mzXML format using MS convert version 3.0.5533 [93]. The converted files were searched against *Homo sapiens* database using comet version 2014.02 rev. 2 [94]. The UniProt *Homo sapiens* protein database contained 26,000 protein sequences and we also included decoy sequences (decoy sequences was generated using Decoy tool in Trans-Proteomic Pipeline by using “randomized sequences and interleave entries” decoy algorithm) and contaminant sequences from cRAP database (http://www.thegpm.org/crap/). The comet search parameters such as 25 ppm peptide mass tolerance in peptide prophet, 1.0005 m/z fragment bin tolerance, 0.4 m/z monoisotopic offset, semi-digested trypsin termini with two allowed missed cleavage, carbamidomethylation of cysteine (+57.021464 Da) and iTRAQ labeling on N-terminal and lysine (+144.10206 Da) as a static modification and oxidation of methionine and tryptophan as variable modifications were used. Trans-Proteomic Pipeline (TPP) v5.0 typhoon was used for protein identification as well as for quantitation. PeptideProphet tool was used to assess the peptide spectral match (PSM) for individual files whereas iProphet tool was used to combine individual PeptideProphet files. Further, ProteinProphet was used for the identification of the proteins on the iProphet files and proteins with 1.0% FDR were considered as a true identification for protein quantitation [95–97]. The quantitation of each protein based on the iTRAQ reporter ion was measured using Libra algorithm in TPP module [21].

### Comparison of the expression levels of proteins identified from this study with the data available in literature

Significantly altered proteins from this study were compared against the list of proteins identified to be dysregulated in the study at CPTAC portal [24]. Further, the expression levels of these proteins in colorectal tumors available at the Human Protein Atlas was compared to shortlist the targets for validation.

### Bioinformatics analysis

The iTRAQ data was subjected to bioinformatic analysis using the DAVID Functional Annotation Bioinformatics Analysis v6.8 to identify the pathways affected in the colon adenocarcinoma.

An enrichment analysis of the common differentially expressed proteins identified from iTRAQ analysis using Spectrum Mill and Trans-Proteomic Pipeline together with their fold changes was performed using MetaCore version 6.30, build 68780 (Thomson Reuters). Thereby, the top ten statistically enriched pathway maps, process networks, diseases (by biomarkers) and GO Processes were identified for the up-, and the down-regulated proteins separately, and then for all dysregulated proteins together.

In addition, the MetaCore “Interactions by Protein Functions” tool was applied with a FDR threshold of 0.05 to identify proteins that are functionally over-connected with proteins (up-, down-regulated and all dysregulated proteins) in the data set.

Furthermore, MetaCore was used to identify transcription factors for which targets are enriched in the data set (up-, down-regulated and all dysregulated proteins) with a FDR threshold of 0.05.

We used the network propagation method by Vanunu *et al.* 2010 [27] for the identification of disease-relevant proteins. The algorithm was given a list of known disease genes to be used as prior knowledge, and it simulated a diffusion process over the HIPPIE protein-protein network [28] by iteratively applying the formula:

$$F^{t}:=\alpha W^{\prime }F^{t-1}+\left(1-\alpha \right)Y$$

where *F* is a prioritization function which reflects the relevance of all proteins in the network to the disease, *W*’ is a matrix whose values are the normalized confidence scores of the network edges, *Y* is the prior knowledge vector (which in our case contains ‘1’s for known disease-relevant proteins and ‘0’s for other proteins), *t* is a given time point, and α∈(0,1) weighs the relative importance of the network and prior knowledge terms. More details can be found in the study by Vanunu *et al.* 2010 [27].

### Validation by western blot

Of the 11 paired samples used for shotgun experiments, colon adenocarcinoma tissue lysates from seven individuals and their corresponding matched control tissue lysates were used for the validation of S100A9 using western blotting (WB) method. Blocking of membrane was done using 5% BSA in TBST at RT followed by overnight incubation with 1:1000 dilution of polyclonal primary antibody against S100A9 at 4° C. The blot was incubated with 1:3000 dilution of secondary antibody conjugated with HRP (GeNei (MERCK)-621140380011730 or 621140680011730). A chromogenic substance, TMB/H2O2 (GeNei) was used for visualization of protein bands and the target protein bands were normalized against the uniform intensity band (approx. 70 kDa) in the ponceau image of the blot. Quantitation of the S100A9 protein was performed using ImageQuantTL software version 5.0 (GE Healthcare).

### MRM based validation

Validation of significantly altered proteins was done using multiple reaction monitoring (MRM) method, where ten tumor and matched control lysates (subset of the 11 samples used for shotgun experiments) were trypsin digested (96 µg each) and reconstituted in 50 µl of 0.1% formic acid. The MRM assay was performed using LC-MS 8050 triple quadrupole (QQQ) mass spectrometer (Shimadzu) coupled with Nexera UHPLC. For each protein at least three peptides and for each peptide minimum three transitions were monitored. Peptides were analysed on QQQ in the MRM mode with dwell time of 0.005 sec and a 0.003 sec pause time for every transition. All transitions were scheduled using a retention time window of ± 1 minute. The list of the peptide sequences and their retention times for nine proteins monitored using MRM has been provided in Supplementary Table 1F. A total of 138 transitions were monitored using a single method file. Each sample was run in triplicates (injection volume – 3 × 5ul) with a run time of 18 minutes each. For acquisition of data in MRM mode following parameters were used: Q1 resolution, 0.7 FWHM, Q3 resolution, 0.7 FWHM; with a collision gas pressure of 270kPa. Peptides separation was done on a Shim-pack XR-ODS II (75 mm L × 3 mm I.D.; 2.2 µm) column, using linear gradient of 0.1% formic acid in milli Q water (solvent A) and acetonitrile/water at a flow rate of 0.4 ml/min with the following gradient method: 0.01–1.5 min., 3% solvent B; 1.5–10 min., 3–50% solvent B; 10–11 min., 50–95% solvent B; 11–14 min., 95% solvent B; 14–14.1 min., 95–3% solvent B; 14.1–18 min., 3% solvent B. MRM data was analysed using Skyline software version 3.6 [98] to validate the significantly dysregulated proteins.

## SUPPLEMENTARY MATERIALS TABLES





















## Abbreviations

CAC: Colon Adenocarcinoma
iTRAQ: Isobaric Tagging for Relative and Absolute Quantification
LC-MS/MS: Liquid Chromatography-Tandem Mass Spectrometry
MRM: Multiple Reaction Monitoring
TPP: Trans-Proteomic Pipeline
HPA: Human Protein Atlas
CPTAC: Clinical Proteome Tumor Analysis Consortium
SM: Spectrum Mill
