# Supplementary material for: Quantitative mass spectrometry analysis reveals a panel of nine proteins as diagnostic markers for colon adenocarcinomas
Source: Oncotarget. 2018 Feb 5;9(17):13530–44. doi: 10.18632/oncotarget.24418 (PMC5862596; doi:10.18632/oncotarget.24418)
Supplement: Supplementary file 1 [file oncotarget-09-13530-s001.pdf]

Quantitative mass spectrometry analysis reveals a panel of nine proteins as diagnostic markers for colon adenocarcinomas

SUPPLEMENTARY MATERIALS

Supplementary Table 1A: List of differentially expressed proteins identified from iTRAQ analysis using Spectrum Mill (SM). See Supplementary_Table_1A

Supplementary Table 1B: List of differentially expressed proteins identified from iTRAQ analysis using Trans Proteome Pipeline (TPP). See Supplementary_Table_1B

Supplementary Table 1C: List of significantly differentially expressed proteins identified from iTRAQ analysis using Spectrum Mill and Trans Proteome Pipeline (Common between the two). See Supplementary_Table_1C

Supplementary Table 1D: Comparison of significantly differentially expressed proteins identified in present study (Supplementary Table 1C) with significantly differentially expressed proteins identified from Jankova *et al*. 2011 and Wisniewski *et al*. 2012. See Supplementary_Table_1D

Supplementary Table 1E: Proteins involved in biological processes (Output from KEGG pathway database, Database for Annotation, Visualization and Integrated Discovery (DAVID) Functional Annotation Bioinformatics Analysis v6.8) using the 285 dysregulated proteins identified using TPP or Spectrum Mill) altered in colon adenocarcinoma. See Supplementary_Table_1E

Supplementary Table 1F: Peptide details for the 9 proteins validated using MRM. See Supplementary_Table_1F

Supplementary Table 1G: 20 commonest proteins as discovered by the diffusion propagation analysis. See Supplementary_Table_1G

Supplementary Table 1H: List of differentially expressed proteins identified from iTRAQ analysis using Spectrum Mill and/or Trans Proteome Pipeline and their comparison with CPTAC and Human Protein Atlas data for colorectal cancer. See Supplementary_Table_1H

Supplementary Table 1I: Sample details. See Supplementary_Table_1I
