# Supplementary material for: Quantitative mass spectrometry analysis reveals a panel of nine proteins as diagnostic markers for colon adenocarcinomas
Source: Oncotarget. 2018 Feb 5;9(17):13530–44. doi: 10.18632/oncotarget.24418 (PMC5862596; doi:10.18632/oncotarget.24418)
Supplement: Supplementary file 2 [file oncotarget-09-13530-s002.docx]

| **Supplementary Table 1A: List of differentially expressed proteins identified from iTRAQ analysis using Spectrum Mill (SM)** | | | | | | | | | | | | | | | | | | | | | | | |
| --- | --- | --- | --- | --- | --- | --- | --- | --- | --- | --- | --- | --- | --- | --- | --- | --- | --- | --- | --- | --- | --- | --- | --- |
| accession_number | entry_name | protein_mw | Set 1_Number of unique peptides | Set 1_percentCoverage | Set 1_Fold Change_115/114 | Set 1_Fold Change_116/114 | Set 1_Fold Change_117/114 | Set 2_Number of unique peptides | Set 2_percentCoverage | Set 2_Fold Change_115/114 | Set 2_Fold Change_116/114 | Set 2_Fold Change_117/114 | Set 3_Number of unique peptides | Set 3_percentCoverage | Set 3_Fold Change_115/114 | Set 3_Fold Change_116/114 | Set 3_Fold Change_117/114 | Set 4_Number of unique peptides | Set 4_percentCoverage | Set 4_Fold Change_115/114 | Set 4_Fold Change_116/114 | Set 4_Fold Change_117/114 (Pool) | Average Fold change (n=11) |
| Q01105 | Protein SET | 36803.8 | * | * | * | * | * | 3 | 12.7 | 2.0 | 0.8 | 1.9 | 2 | 7.9 | 11.4 | 7.5 | 7.0 | 2 | 15.5 | 2.1 | 1.5 | 2.0 | 4.267 |
| P06702 | Protein S100-A9 | 14884.5 | 7 | 66.6 | 2.3 | 3.3 | 1.1 | 8 | 68.4 | 6.3 | 1.6 | 3.7 | 6 | 65.7 | 2.9 | 3.3 | 3.2 | 8 | 81.5 | 3.3 | 2.3 | 2.8 | 3.011 |
| P78527 | DNA-dependent protein kinase catalytic subunit | 513830.6 | 2 | 0.4 | 1.7 | 5.1 | 3.4 | * | * | * | * | * | 5 | 1.5 | 4.1 | 2.7 | 2.1 | 3 | 0.9 | 2.8 | 1.1 | 1.8 | 2.874 |
| P08195 | 4F2 cell-surface antigen heavy chain | 72401.4 | 2 | 3.9 | 2.3 | 2.8 | 1.6 | * | * | * | * | * | 2 | 4.9 | 4.1 | 2.8 | 3.8 | 3 | 8.2 | 2.7 | 2.5 | 2.1 | 2.825 |
| P61626 | Lysozyme C | 17858.1 | 2 | 13.5 | 2.5 | 2.9 | 3.8 | 2 | 12.8 | 2.8 | 1.1 | 1.8 | * | * | * | * | * | 3 | 18.2 | 5.6 | 2.1 | 2.5 | 2.815 |
| P80188 | Neutrophil gelatinase-associated lipocalin | 25065.3 | * | * | * | * | * | 6 | 33.3 | 7.8 | 1.0 | 1.4 | 3 | 19.6 | 2.0 | 4.2 | 1.5 | 5 | 31.8 | 2.9 | 1.6 | 1.9 | 2.810 |
| P05109 | Protein S100-A8 | 12621.2 | 4 | 31.1 | 2.8 | 3.3 | 1.0 | 6 | 39.7 | 3.4 | 1.3 | 2.7 | 4 | 34.4 | 2.8 | 2.5 | 3.3 | 8 | 41.9 | 5.3 | 2.0 | 2.2 | 2.777 |
| P19971 | Thymidine phosphorylase | 51939.7 | 2 | 7 | 2.8 | 3.6 | 0.7 | 4 | 16.1 | 5.3 | 3.2 | 0.9 | * | * | * | * | * | 5 | 18 | 1.9 | 3.7 | 1.9 | 2.769 |
| Q8NC51 | Plasminogen activator inhibitor 1 RNA-binding protein | 50067 | 4 | 11 | 1.4 | 4.0 | 2.1 | * | * | * | * | * | 2 | 7.1 | 3.9 | 5.0 | 2.8 | 2 | 8.3 | 1.2 | 1.7 | 1.1 | 2.766 |
| P31948 | Stress-induced-phosphoprotein 1 | 72347.5 | 5 | 9.5 | 0.9 | 2.0 | 1.9 | * | * | * | * | * | 6 | 11.2 | 3.3 | 4.3 | 3.1 | 3 | 5.3 | 2.8 | 3.6 | 2.3 | 2.745 |
| P00491 | Purine nucleoside phosphorylase | 34075.4 | 2 | 10.3 | 1.4 | 2.6 | 1.5 | * | * | * | * | * | 3 | 21.1 | 3.2 | 4.4 | 3.6 | 2 | 10.3 | 2.9 | 2.3 | 1.7 | 2.717 |
| Q92841 | Probable ATP-dependent RNA helicase DDX17 | 85424.4 | * | * | * | * | * | 3 | 6.1 | 2.6 | 0.7 | 4.3 | 2 | 2.8 | 4.0 | 3.5 | 1.2 | 3 | 5.6 | 2.3 | 3.1 | 2.1 | 2.695 |
| Q9Y3D6 | Mitochondrial fission 1 protein | 19589.2 | 2 | 7.8 | 1.1 | 1.5 | 1.2 | * | * | * | * | * | 2 | 7.8 | 3.4 | 6.9 | 2.8 | 2 | 7.8 | 2.6 | 2.0 | 2.1 | 2.688 |
| Q05707 | Collagen alpha-1(XIV) chain | 206993.2 | 2 | 1.6 | 1.4 | 1.1 | 0.8 | 4 | 2.9 | 0.5 | 0.9 | 1.0 | * | * | * | * | * | 3 | 2.8 | 13.5 | 2.2 | 3.5 | 2.684 |
| P34932 | Heat shock 70 kDa protein 4 | 106861.5 | 2 | 3.9 | 2.8 | 5.4 | 1.3 | * | * | * | * | * | 2 | 3.9 | 1.7 | 5.9 | 0.7 | 2 | 3.9 | 1.8 | 1.2 | 1.0 | 2.587 |
| P19338 | Nucleolin | 89643.9 | 12 | 20.1 | 1.2 | 3.4 | 1.6 | 9 | 16.3 | 2.4 | 1.3 | 2.4 | 18 | 27.8 | 3.6 | 3.4 | 2.6 | 13 | 20.8 | 3.0 | 2.6 | 2.8 | 2.506 |
| O95881 | Thioredoxin domain-containing protein 12 | 21250.6 | 2 | 13.9 | 1.8 | 3.1 | 1.2 | * | * | * | * | * | 3 | 22 | 3.9 | 4.5 | 2.6 | 3 | 22 | 1.0 | 1.9 | 1.2 | 2.497 |
| P23246 | Splicing factor, proline- and glutamine-rich | 81019.2 | 2 | 5.3 | 0.7 | 1.7 | 0.9 | * | * | * | * | * | 2 | 4.1 | 2.8 | 3.6 | 2.2 | 2 | 5.6 | 3.4 | 4.7 | 1.7 | 2.490 |
| P26641 | Elongation factor 1-gamma | 55073.1 | 4 | 10.7 | 1.4 | 3.9 | 2.7 | 5 | 12.1 | 1.8 | 1.2 | 2.3 | 6 | 16.2 | 2.6 | 3.2 | 2.4 | 7 | 18 | 3.6 | 2.3 | 1.8 | 2.488 |
| P68431 | Histone H3.1 | 17391.9 | 3 | 14.7 | 2.1 | 1.8 | 1.1 | 3 | 14.7 | 3.6 | 1.7 | 2.6 | 2 | 9.5 | 5.0 | 2.5 | 2.5 | 3 | 14.7 | 2.4 | 2.0 | 1.8 | 2.475 |
| Q86V81 | THO complex subunit 4 | 28473.1 | 3 | 8.9 | 1.1 | 2.5 | 1.3 | * | * | * | * | * | 2 | 7 | 2.3 | 3.1 | 1.9 | 2 | 7 | 5.0 | 2.6 | 2.6 | 2.475 |
| Q9UQ80 | Proliferation-associated protein 2G4 | 50182.9 | 6 | 23.3 | 1.1 | 2.8 | 1.3 | * | * | * | * | * | 4 | 15.9 | 3.3 | 4.7 | 1.9 | 4 | 16.7 | 2.7 | 2.0 | 1.9 | 2.471 |
| P53999 | Activated RNA polymerase II transcriptional coactivator p15 | 17134 | 2 | 19.6 | 1.7 | 3.0 | 1.8 | * | * | * | * | * | 2 | 19.6 | 2.6 | 2.9 | 3.1 | 3 | 19.6 | 2.5 | 1.8 | 1.6 | 2.435 |
| P36542 | ATP synthase subunit gamma, mitochondrial | 36800.5 | 2 | 7.3 | 0.7 | 1.8 | 1.0 | 2 | 7.7 | 1.9 | 1.0 | 2.1 | 2 | 7.7 | 3.8 | 7.3 | 2.0 | * | * | * | * | * | 2.411 |
| P39019 | 40S ribosomal protein S19gi\|73917925\|sp\|Q5R8M9.3\|RS19_PONAB RecName | 18222.5 | 3 | 20.6 | 1.6 | 3.0 | 1.0 | * | * | * | * | * | 2 | 13.7 | 3.1 | 3.3 | 2.3 | 2 | 6.8 | 2.8 | 2.0 | 2.4 | 2.401 |
| P20290 | Transcription factor BTF3 | 24531.1 | 2 | 19.9 | 0.8 | 1.8 | 3.6 | * | * | * | * | * | 3 | 20.3 | 3.1 | 3.5 | 2.1 | 3 | 20.3 | 2.5 | 1.9 | 1.4 | 2.398 |
| P41250 | Glycine--tRNA ligase | 91401.8 | 2 | 4.4 | 0.8 | 2.3 | 0.9 | * | * | * | * | * | 5 | 11.7 | 3.8 | 3.7 | 2.2 | 3 | 7.9 | 3.1 | 2.3 | 1.5 | 2.374 |
| P11940 | Polyadenylate-binding protein 1 | 76808.2 | 5 | 10.6 | 0.9 | 1.5 | 1.1 | 2 | 4.7 | 1.4 | 2.8 | 3.0 | 6 | 12.2 | 3.2 | 4.1 | 2.8 | 7 | 14.6 | 2.8 | 2.5 | 2.0 | 2.366 |
| Q13838 | Spliceosome RNA helicase DDX39B | 53627.4 | 5 | 14.4 | 1.6 | 3.0 | 1.7 | * | * | * | * | * | 4 | 10 | 2.7 | 3.3 | 1.9 | 4 | 10 | 2.6 | 2.1 | 2.5 | 2.363 |
| P05164 | Myeloperoxidase | 88008.4 | 2 | 3.2 | 2.1 | 2.7 | 1.7 | 8 | 12.8 | 3.6 | 1.4 | 2.7 | 2 | 3.2 | 2.5 | 1.0 | 1.6 | 12 | 23.2 | 4.1 | 2.1 | 2.1 | 2.327 |
| Q15181 | Inorganic pyrophosphatase | 37152.2 | 4 | 23.1 | 1.2 | 2.1 | 1.3 | * | * | * | * | * | 6 | 26.2 | 3.3 | 5.1 | 1.9 | 3 | 17.3 | 1.4 | 2.3 | 2.0 | 2.324 |
| P19075 | Tetraspanin-8 | 28602 | 2 | 10.1 | 1.8 | 3.4 | 2.4 | * | * | * | * | * | 2 | 10.1 | 2.4 | 3.1 | 1.6 | 2 | 10.1 | 2.0 | 1.8 | 2.7 | 2.323 |
| P36578 | 60S ribosomal protein L4 | 56198.5 | 3 | 8.1 | 1.7 | 3.4 | 0.9 | 4 | 10.5 | 1.7 | 1.1 | 1.9 | 5 | 11.9 | 3.7 | 4.1 | 2.0 | 2 | 5.1 | 2.4 | 2.4 | 2.1 | 2.316 |
| P13010 | X-ray repair cross-complementing protein 5 | 92355.3 | * | * | * | * | * | 2 | 2.8 | 1.2 | 1.1 | 0.9 | 5 | 12.1 | 2.7 | 2.7 | 2.0 | 3 | 6.2 | 5.4 | 2.4 | 2.0 | 2.307 |
| P06731 | Carcinoembryonic antigen-related cell adhesion molecule 5 | 79985.6 | * | * | * | * | * | 2 | 4.8 | 2.4 | 0.7 | 1.8 | 4 | 9.8 | 2.5 | 2.7 | 3.0 | 4 | 11.8 | 3.0 | 2.3 | 2.3 | 2.307 |
| Q03252 | Lamin-B2 | 73423.7 | * | * | * | * | * | 2 | 3.8 | 1.2 | 1.3 | 1.5 | 2 | 3 | 2.8 | 4.0 | 2.8 | 4 | 6.3 | 2.9 | 1.8 | 1.6 | 2.293 |
| P46783 | 40S ribosomal protein S10gi\|108860951\|sp\|Q3T0F4.1\|RS10_BOVIN RecName | 20771.4 | 2 | 9.6 | 1.5 | 3.9 | 0.7 | 2 | 9.6 | 2.5 | 1.2 | 0.6 | 5 | 29.6 | 4.1 | 5.8 | 2.3 | 5 | 33.3 | 0.6 | 1.9 | 1.6 | 2.278 |
| O60812 | Heterogeneous nuclear ribonucleoprotein C-like 1 | 36523.5 | * | * | * | * | * | 3 | 8.1 | 2.2 | 1.3 | 2.3 | 3 | 11.6 | 2.6 | 3.6 | 1.7 | 4 | 15 | 2.5 | 2.1 | 2.1 | 2.274 |
| P62241 | 40S ribosomal protein S8gi\|54039527\|sp\|P62243.2\|RS8_RAT RecName | 28958.8 | 2 | 11.5 | 1.3 | 3.7 | 1.6 | 3 | 15.8 | 1.5 | 1.1 | 1.0 | 2 | 11.5 | 3.7 | 3.6 | 3.2 | 2 | 11.5 | 2.2 | 2.1 | 2.2 | 2.272 |
| P31949 | Protein S100-A11 | 13295.9 | 6 | 51.4 | 2.9 | 3.0 | 1.6 | 5 | 41.9 | 1.4 | 1.6 | 2.2 | 5 | 47.6 | 2.2 | 2.8 | 1.8 | 6 | 45.7 | 2.1 | 3.3 | 2.3 | 2.254 |
| P21796 | Voltage-dependent anion-selective channel protein 1 | 34490 | 3 | 15.1 | 1.8 | 1.7 | 1.8 | 3 | 10.6 | 1.3 | 0.8 | 2.1 | 8 | 35.3 | 4.5 | 3.5 | 2.5 | 5 | 25.7 | 2.5 | 2.2 | 2.2 | 2.248 |
| P50454 | Serpin H1 | 51455.1 | 6 | 24.6 | 2.0 | 1.9 | 1.1 | 6 | 23.2 | 2.4 | 2.0 | 2.3 | 6 | 23.2 | 2.6 | 3.1 | 3.3 | 10 | 35.4 | 1.5 | 2.4 | 2.1 | 2.234 |
| P49720 | Proteasome subunit beta type-3 | 24963.6 | 2 | 16.5 | 1.2 | 2.2 | 1.2 | * | * | * | * | * | 3 | 20 | 2.3 | 4.5 | 1.9 | 4 | 25.8 | 2.1 | 2.3 | 1.6 | 2.208 |
| P04114 | Apolipoprotein B-100 | 568283.2 | 3 | 0.9 | 3.7 | 5.7 | 2.0 | 7 | 2.3 | 1.8 | 1.1 | 0.8 | 2 | 0.8 | 2.1 | 3.1 | 1.1 | 3 | 1.3 | 1.0 | 1.9 | 1.8 | 2.205 |
| P46781 | 40S ribosomal protein S9gi\|52783339\|sp\|Q6ZWN5.3\|RS9_MOUSE RecName | 25387 | 3 | 15.4 | 0.7 | 1.6 | 0.7 | 2 | 7.7 | 1.1 | 0.8 | 2.4 | 3 | 14.9 | 3.1 | 4.8 | 2.4 | 6 | 26.8 | 3.9 | 2.5 | 1.7 | 2.189 |
| O14980 | Exportin-1 | 133462.3 | 2 | 1.9 | 2.0 | 3.0 | 2.3 | * | * | * | * | * | 6 | 7.1 | 2.2 | 2.1 | 1.5 | 4 | 5.2 | 2.3 | 2.0 | 1.6 | 2.178 |
| P18621 | 60S ribosomal protein L17 | 25228.7 | 2 | 14.6 | 1.3 | 2.4 | 1.3 | 3 | 13.5 | 2.0 | 1.4 | 1.6 | 3 | 19.5 | 3.4 | 3.4 | 2.8 | * | * | * | * | * | 2.178 |
| P37837 | Transaldolase | 42035.2 | 5 | 16 | 1.3 | 2.3 | 1.1 | 5 | 16 | 1.0 | 0.9 | 1.7 | 8 | 22.5 | 3.6 | 4.5 | 3.2 | 11 | 30.8 | 2.3 | 2.1 | 2.0 | 2.177 |
| P04080 | Cystatin-B | 12493.8 | 5 | 64.2 | 1.7 | 2.1 | 2.2 | 4 | 42.8 | 2.2 | 1.3 | 2.7 | 5 | 64.2 | 1.7 | 2.5 | 1.8 | 6 | 64.2 | 2.9 | 2.6 | 2.0 | 2.163 |
| Q08211 | ATP-dependent RNA helicase A | 151638.2 | 2 | 2.6 | 1.3 | 2.4 | 1.0 | * | * | * | * | * | 9 | 9.6 | 2.6 | 3.3 | 2.4 | 4 | 3.9 | 2.2 | 2.0 | 2.1 | 2.158 |
| P43243 | Matrin-3 | 104649.2 | 3 | 6.2 | 1.7 | 1.9 | 1.5 | 4 | 7.2 | 2.3 | 1.4 | 1.9 | 7 | 14.6 | 3.7 | 2.6 | 1.5 | 5 | 9.3 | 2.9 | 2.1 | 1.7 | 2.155 |
| P50991 | T-complex protein 1 subunit delta | 64058.7 | 3 | 7.7 | 0.9 | 2.5 | 1.1 | * | * | * | * | * | 4 | 13.7 | 1.5 | 2.7 | 1.2 | 2 | 5.3 | 5.2 | 2.2 | 1.8 | 2.153 |
| Q02878 | 60S ribosomal protein L6 | 40280.4 | 6 | 25.3 | 1.8 | 2.9 | 1.6 | 3 | 14.2 | 0.9 | 1.0 | 1.8 | 6 | 23.6 | 2.4 | 3.6 | 2.2 | 4 | 17.7 | 2.9 | 2.5 | 1.8 | 2.133 |
| Q08EQ4 | Thymosin beta-4-like protein 3 | 6368 | 4 | 43.1 | 1.5 | 3.0 | 1.3 | 4 | 43.1 | 1.3 | 1.6 | 1.6 | 5 | 45.4 | 2.2 | 3.7 | 2.0 | 4 | 43.1 | 2.5 | 2.7 | 1.7 | 2.125 |
| P30048 | Thioredoxin-dependent peroxide reductase, mitochondrial | 29908.5 | 2 | 9.7 | 0.8 | 1.8 | 1.4 | * | * | * | * | * | 2 | 9.7 | 2.9 | 3.7 | 2.2 | 2 | 9.7 | 1.9 | 2.1 | 1.1 | 2.107 |
| P12724 | Eosinophil cationic protein | 19186.8 | 2 | 16.8 | 0.8 | 3.2 | 0.7 | * | * | * | * | * | 2 | 16.8 | 2.4 | 4.9 | 1.9 | 2 | 16.8 | 2.1 | 0.9 | 2.0 | 2.104 |
| P10809 | 60 kDa heat shock protein, mitochondrial | 68864.9 | 26 | 46.2 | 1.3 | 3.3 | 1.0 | 22 | 41.7 | 2.7 | 1.0 | 1.0 | 27 | 47.4 | 3.7 | 3.4 | 2.3 | 22 | 41.8 | 1.1 | 2.3 | 1.8 | 2.103 |
| O43242 | 26S proteasome non-ATPase regulatory subunit 3 | 66135.9 | 2 | 5.2 | 1.4 | 2.2 | 0.9 | * | * | * | * | * | 2 | 5.2 | 2.4 | 4.9 | 1.8 | 2 | 5.2 | 1.5 | 1.8 | 1.1 | 2.100 |
| Q13765 | Nascent polypeptide-associated complex subunit alpha | 25690 | 2 | 13 | 1.4 | 2.4 | 1.1 | * | * | * | * | * | 2 | 13 | 2.2 | 3.9 | 1.1 | 3 | 19.5 | 2.8 | 1.9 | 1.7 | 2.098 |
| P78417 | Glutathione S-transferase omega-1 | 31166.2 | 3 | 11.2 | 2.0 | 1.7 | 1.3 | 2 | 9.5 | 1.3 | 1.3 | 1.9 | 7 | 26.1 | 2.9 | 3.3 | 1.8 | 6 | 21.1 | 2.8 | 2.5 | 1.9 | 2.097 |
| Q9Y383 | Putative RNA-binding protein Luc7-like 2 | 51438.3 | 2 | 4.8 | 1.2 | 2.0 | 0.9 | * | * | * | * | * | 2 | 4.8 | 3.1 | 3.3 | 2.5 | 2 | 4.3 | 2.2 | 1.7 | 1.5 | 2.092 |
| P15880 | 40S ribosomal protein S2 | 35068.8 | 2 | 8.5 | 1.3 | 3.7 | 1.3 | 2 | 7.8 | 2.2 | 1.6 | 2.6 | 4 | 16 | 2.6 | 2.9 | 1.5 | 3 | 12.2 | 1.4 | 1.9 | 1.7 | 2.081 |
| P22626 | Heterogeneous nuclear ribonucleoproteins A2/B1 | 40225 | 12 | 39.9 | 1.1 | 3.0 | 1.5 | 10 | 34.2 | 1.8 | 1.1 | 2.1 | 16 | 45.6 | 2.6 | 3.6 | 1.8 | 10 | 35.6 | 2.2 | 2.0 | 1.9 | 2.066 |
| P40926 | Malate dehydrogenase, mitochondrial | 39707.1 | 9 | 35.7 | 1.1 | 2.5 | 1.3 | 9 | 35.7 | 2.2 | 1.0 | 1.6 | 12 | 42.3 | 3.0 | 3.4 | 2.3 | 12 | 42.8 | 2.2 | 2.1 | 1.7 | 2.066 |
| P49327 | Fatty acid synthase | 288299.2 | 4 | 3.1 | 1.0 | 1.6 | 0.9 | 2 | 1.1 | 2.3 | 2.5 | 2.7 | 8 | 4.8 | 2.2 | 3.8 | 1.4 | 10 | 8 | 2.5 | 1.8 | 2.0 | 2.064 |
| Q96KP4 | Cytosolic non-specific dipeptidase | 58409.4 | 6 | 17.4 | 1.4 | 2.2 | 0.9 | 2 | 3.7 | 0.9 | 4.1 | 1.8 | 4 | 11.1 | 2.0 | 3.1 | 1.8 | 6 | 16.2 | 2.1 | 2.4 | 1.6 | 2.059 |
| P55327 | Tumor protein D52 | 27410.9 | 4 | 18.3 | 0.9 | 2.6 | 1.4 | 3 | 13.3 | 2.2 | 0.9 | 1.7 | 3 | 17.8 | 2.2 | 5.5 | 2.1 | 2 | 13.3 | 1.9 | 1.2 | 1.2 | 2.056 |
| P06748 | Nucleophosmin | 37502.7 | 6 | 24.4 | 1.5 | 2.5 | 1.4 | 5 | 17.3 | 2.2 | 1.5 | 1.7 | 10 | 38.4 | 2.9 | 3.7 | 1.7 | 6 | 19.7 | 1.5 | 1.9 | 1.8 | 2.054 |
| Q96AG4 | Leucine-rich repeat-containing protein 59 | 40662.9 | 3 | 12 | 1.2 | 2.6 | 0.8 | * | * | * | * | * | 3 | 12 | 2.6 | 3.4 | 1.4 | 3 | 12 | 1.7 | 2.5 | 1.9 | 2.040 |
| P42765 | 3-ketoacyl-CoA thiolase, mitochondrial | 46127.8 | 5 | 19.8 | 1.7 | 1.3 | 1.4 | * | * | * | * | * | 4 | 12.3 | 2.5 | 3.2 | 1.6 | 3 | 11.5 | 1.4 | 3.4 | 1.5 | 2.038 |
| P09429 | High mobility group protein B1 | 31263.1 | 8 | 28.8 | 1.3 | 2.7 | 1.4 | 4 | 26 | 1.7 | 1.1 | 1.9 | 12 | 39 | 2.6 | 3.1 | 1.8 | 9 | 33.9 | 2.4 | 2.3 | 2.2 | 2.034 |
| Q9UL46 | Proteasome activator complex subunit 2 | 30743.7 | 5 | 26.7 | 1.5 | 2.4 | 1.4 | 4 | 21.3 | 1.6 | 1.3 | 2.0 | 4 | 19.2 | 2.7 | 3.0 | 2.0 | 5 | 24.6 | 2.2 | 2.2 | 2.0 | 2.031 |
| P09525 | Annexin A4 | 39281.7 | 9 | 33.2 | 1.3 | 1.9 | 1.2 | 5 | 15.3 | 1.5 | 0.9 | 1.5 | 11 | 34.4 | 2.6 | 3.2 | 2.6 | 11 | 30.4 | 2.7 | 3.0 | 1.9 | 2.030 |
| P14314 | Glucosidase 2 subunit beta | 65151.4 | 3 | 8.7 | 1.6 | 1.9 | 1.0 | 2 | 6.8 | 1.1 | 0.8 | 0.8 | 2 | 7 | 3.7 | 3.9 | 2.5 | 3 | 9.2 | 3.1 | 2.0 | 1.4 | 2.024 |
| Q99729 | Heterogeneous nuclear ribonucleoprotein A/B | 40518.8 | 4 | 14.4 | 1.7 | 2.7 | 1.0 | * | * | * | * | * | 4 | 11.4 | 1.9 | 3.4 | 1.3 | 5 | 11.7 | 1.9 | 2.1 | 1.8 | 2.019 |
| Q12906 | Interleukin enhancer-binding factor 3 | 105106.1 | 4 | 5.7 | 1.2 | 2.0 | 1.6 | 2 | 2.4 | 1.8 | 1.1 | 1.8 | 5 | 7.8 | 2.3 | 2.5 | 1.9 | 5 | 8.1 | 4.0 | 1.8 | 2.2 | 2.008 |
| P07108 | Acyl-CoA-binding protein | 11918.2 | 4 | 62 | 1.5 | 2.7 | 1.3 | 3 | 50.5 | 1.4 | 0.9 | 1.6 | 3 | 52.8 | 2.6 | 3.6 | 1.9 | 4 | 62 | 2.5 | 2.2 | 1.9 | 2.006 |
| Q00839 | Heterogeneous nuclear ribonucleoprotein U | 101415.3 | 8 | 14.4 | 0.8 | 2.4 | 1.0 | 4 | 5.8 | 2.4 | 1.5 | 2.1 | 9 | 13.3 | 2.9 | 3.5 | 1.6 | 6 | 9.2 | 1.7 | 2.2 | 2.2 | 2.005 |
| Q14103 | Heterogeneous nuclear ribonucleoprotein D0 | 43217.6 | 4 | 13.2 | 1.5 | 2.4 | 1.2 | 2 | 7.3 | 1.5 | 1.0 | 0.8 | 5 | 10.9 | 3.5 | 3.0 | 2.6 | 4 | 7.3 | 2.2 | 2.2 | 2.4 | 1.992 |
| P07339 | Cathepsin D | 48380.4 | 7 | 20.1 | 1.7 | 1.8 | 1.0 | 5 | 14 | 1.4 | 1.2 | 1.0 | 4 | 12.3 | 2.6 | 3.5 | 2.3 | 10 | 25.2 | 1.5 | 4.0 | 1.7 | 1.986 |
| P02794 | Ferritin heavy chain | 23270.4 | 4 | 20.2 | 3.3 | 1.4 | 0.8 | * | * | * | * | * | 2 | 14.2 | 0.6 | 1.3 | 1.2 | 4 | 20.7 | 2.4 | 4.8 | 2.4 | 1.984 |
| P29401 | Transketolase | 74615.5 | 11 | 25.5 | 1.2 | 2.0 | 1.2 | 8 | 23.2 | 2.3 | 1.4 | 2.2 | 10 | 25.3 | 2.4 | 2.9 | 1.6 | 13 | 29.8 | 2.5 | 2.1 | 1.9 | 1.979 |
| P01019 | Angiotensinogen | 56465.7 | 4 | 10.9 | 3.5 | 2.0 | 1.5 | 3 | 11.9 | 2.6 | 2.7 | 1.2 | 2 | 8.4 | 1.1 | 2.6 | 1.6 | 3 | 11.3 | 1.8 | 1.3 | 1.0 | 1.979 |
| P00558 | Phosphoglycerate kinase 1 | 51067.8 | 20 | 47.9 | 1.5 | 2.3 | 1.1 | 18 | 43.1 | 1.9 | 1.3 | 1.5 | 20 | 58.2 | 2.4 | 3.6 | 2.0 | 20 | 55.1 | 1.8 | 2.4 | 1.7 | 1.977 |
| P60842 | Eukaryotic initiation factor 4A-I | 49264.4 | 3 | 10.5 | 1.0 | 2.8 | 1.2 | 2 | 6.6 | 1.8 | 1.2 | 2.1 | 4 | 13 | 2.2 | 2.7 | 1.6 | 6 | 17.9 | 2.3 | 2.8 | 1.9 | 1.975 |
| P61247 | 40S ribosomal protein S3a | 35650.5 | 6 | 23.8 | 1.2 | 2.8 | 1.5 | 3 | 12.8 | 1.6 | 1.0 | 1.5 | 4 | 15.1 | 2.8 | 3.3 | 2.1 | 3 | 12.8 | 2.3 | 1.6 | 1.7 | 1.974 |
| P48643 | T-complex protein 1 subunit epsilon | 66325.1 | 4 | 10.3 | 1.6 | 2.6 | 1.3 | 2 | 4.4 | 3.3 | 1.7 | 1.2 | 7 | 15.8 | 2.2 | 2.9 | 1.6 | 5 | 12.9 | 1.5 | 1.9 | 1.3 | 1.974 |
| P04843 | Dolichyl-diphosphooligosaccharide--protein glycosyltransferase subunit 1 | 74448.4 | 10 | 21.9 | 1.6 | 2.3 | 1.4 | 5 | 10.3 | 1.4 | 1.3 | 1.9 | 7 | 14.1 | 2.8 | 2.8 | 2.1 | 12 | 26.1 | 2.0 | 1.9 | 1.6 | 1.967 |
| P59998 | Actin-related protein 2/3 complex subunit 4 | 21654.8 | 2 | 11.3 | 1.7 | 2.3 | 0.9 | 2 | 10.1 | 1.7 | 1.0 | 2.0 | 2 | 10.1 | 2.5 | 3.2 | 2.5 | * | * | * | * | * | 1.964 |
| Q04837 | Single-stranded DNA-binding protein, mitochondrial | 18412.6 | 3 | 25.6 | 1.8 | 2.6 | 1.8 | 2 | 20.2 | 2.1 | 1.0 | 0.8 | 3 | 25.6 | 2.4 | 3.1 | 1.8 | 3 | 25.6 | 2.7 | 1.5 | 1.6 | 1.960 |
| P14923 | Junction plakoglobin | 87098 | 3 | 6.1 | 1.8 | 1.7 | 0.9 | * | * | * | * | * | 3 | 5.5 | 3.0 | 2.3 | 1.9 | 3 | 6.1 | 1.5 | 2.5 | 1.7 | 1.960 |
| Q14847 | LIM and SH3 domain protein 1 | 33143.3 | 5 | 21.4 | 1.4 | 1.3 | 1.5 | * | * | * | * | * | 6 | 23.7 | 1.7 | 3.5 | 2.1 | 4 | 18 | 2.5 | 1.7 | 2.0 | 1.959 |
| P39687 | Acidic leucine-rich nuclear phosphoprotein 32 family member C | 31005.5 | 4 | 14.4 | 1.5 | 1.8 | 1.3 | 3 | 11.2 | 1.3 | 1.6 | 2.1 | 3 | 9.2 | 2.3 | 2.4 | 1.8 | 4 | 14.4 | 2.9 | 2.3 | 1.9 | 1.955 |
| P49257 | Protein ERGIC-53 | 62157.8 | 3 | 11.5 | 2.0 | 2.1 | 1.5 | * | * | * | * | * | 2 | 5.2 | 2.3 | 2.7 | 1.3 | 2 | 6 | 2.0 | 1.6 | 1.6 | 1.952 |
| P12956 | X-ray repair cross-complementing protein 6 | 78632.1 | 7 | 16 | 0.9 | 1.9 | 1.4 | 5 | 12.1 | 2.0 | 1.3 | 1.3 | 12 | 28.7 | 3.2 | 2.9 | 2.1 | 9 | 18.5 | 2.2 | 2.3 | 1.7 | 1.952 |
| P00338 | L-lactate dehydrogenase A chain | 41009.6 | 14 | 41.8 | 1.5 | 2.2 | 1.2 | 10 | 28.9 | 1.8 | 1.2 | 1.3 | 18 | 46.6 | 2.2 | 3.3 | 2.6 | 12 | 38.2 | 1.9 | 2.1 | 1.5 | 1.936 |
| P35270 | Sepiapterin reductase | 30351.4 | 3 | 19.5 | 0.9 | 2.9 | 0.2 | * | * | * | * | * | 3 | 19.5 | 3.0 | 3.1 | 1.9 | 3 | 19.5 | 1.0 | 2.4 | 1.6 | 1.935 |
| P23284 | Peptidyl-prolyl cis-trans isomerase B | 27547.2 | 10 | 47.6 | 1.4 | 2.4 | 1.5 | 10 | 38.4 | 1.6 | 1.1 | 1.5 | 10 | 38.4 | 3.5 | 3.0 | 1.7 | 10 | 38.4 | 1.9 | 1.7 | 1.6 | 1.934 |
| P07741 | Adenine phosphoribosyltransferase | 20931.8 | 2 | 17.2 | 1.3 | 2.9 | 1.3 | * | * | * | * | * | 4 | 31.6 | 2.7 | 3.1 | 1.9 | 2 | 17.2 | 1.2 | 1.1 | 1.7 | 1.934 |
| P05141 | ADP/ATP translocase 2 | 36395.4 | 6 | 22.8 | 1.3 | 2.4 | 0.7 | 5 | 19.4 | 1.9 | 1.1 | 1.7 | 7 | 25.1 | 2.8 | 3.5 | 2.0 | * | * | * | * | * | 1.932 |
| P62277 | 40S ribosomal protein S13gi\|51316609\|sp\|Q6ITC7.3\|RS13_CHICK RecName | 19816.8 | 4 | 23.8 | 0.9 | 2.6 | 1.3 | 2 | 17.8 | 1.7 | 1.2 | 1.8 | 6 | 43.7 | 2.0 | 3.0 | 1.9 | 4 | 23.8 | 2.9 | 1.7 | 1.6 | 1.919 |
| P13611 | Versican core protein | 396433.2 | 2 | 0.9 | 2.3 | 1.9 | 0.8 | 2 | 0.9 | 1.8 | 3.6 | 2.2 | * | * | * | * | * | 2 | 0.9 | 1.1 | 1.8 | 1.7 | 1.919 |
| P11586 | C-1-tetrahydrofolate synthase, cytoplasmic | 111179.6 | 2 | 1.9 | 2.3 | 2.9 | 1.1 | * | * | * | * | * | 5 | 7 | 1.6 | 3.0 | 1.1 | 3 | 5.8 | 1.6 | 1.7 | 1.5 | 1.914 |
| P08708 | 40S ribosomal protein S17gi\|338819320\|sp\|P0CW22.1\|RS17L_HUMAN RecName | 17480.9 | 2 | 32.5 | 1.5 | 2.3 | 0.7 | * | * | * | * | * | 2 | 23.7 | 2.7 | 2.8 | 1.8 | 2 | 23.7 | 1.6 | 1.8 | 1.5 | 1.911 |
| P38646 | Stress-70 protein, mitochondrial | 81460.5 | 9 | 17.3 | 1.1 | 2.2 | 1.1 | 6 | 11.4 | 1.8 | 1.2 | 1.9 | 13 | 24.7 | 2.6 | 3.1 | 1.6 | 9 | 17.3 | 2.3 | 2.0 | 1.9 | 1.904 |
| P61769 | Beta-2-microglobulin | 14981.6 | * | * | * | * | * | 2 | 16.8 | 2.2 | 0.9 | 1.3 | 2 | 10.9 | 2.4 | 2.4 | 1.6 | 3 | 21.8 | 2.7 | 1.6 | 1.7 | 1.899 |
| P51991 | Heterogeneous nuclear ribonucleoprotein A3 | 42849.7 | 4 | 15 | 1.4 | 2.2 | 1.3 | 2 | 5.5 | 1.9 | 1.1 | 1.6 | 3 | 9.2 | 2.4 | 3.5 | 1.7 | 4 | 15.6 | 2.0 | 1.8 | 1.8 | 1.897 |
| P07237 | Protein disulfide-isomerase | 64289.9 | 18 | 45.2 | 1.5 | 2.1 | 1.3 | 14 | 31.8 | 1.3 | 1.1 | 1.2 | 19 | 47.2 | 3.0 | 3.2 | 1.6 | 18 | 40.3 | 2.4 | 2.0 | 1.7 | 1.892 |
| P51858 | Hepatoma-derived growth factor | 30794.1 | 3 | 18.3 | 1.5 | 2.4 | 1.8 | 4 | 21.6 | 1.5 | 1.1 | 2.0 | 3 | 18.3 | 2.6 | 2.6 | 1.5 | 5 | 25.8 | 2.0 | 1.7 | 1.6 | 1.892 |
| P40121 | Macrophage-capping protein | 42242.8 | 2 | 7.1 | 1.8 | 1.4 | 0.9 | 3 | 9.1 | 1.7 | 1.1 | 1.5 | 3 | 10.6 | 2.5 | 3.3 | 2.7 | 8 | 34.4 | 1.5 | 2.5 | 1.1 | 1.890 |
| P20674 | Cytochrome c oxidase subunit 5A, mitochondrial | 17942.1 | 2 | 20.6 | 0.9 | 1.1 | 0.4 | 2 | 30 | 5.7 | 1.0 | 2.0 | 5 | 41.3 | 2.4 | 3.4 | 1.6 | 2 | 21.3 | 0.5 | 1.7 | 1.1 | 1.888 |
| P01833 | Polymeric immunoglobulin receptor | 90534.6 | 5 | 12.8 | 1.3 | 1.0 | 1.4 | 7 | 10.8 | 4.3 | 1.1 | 1.4 | 4 | 8.6 | 1.4 | 3.0 | 2.2 | 6 | 12.9 | 2.3 | 1.4 | 1.7 | 1.885 |
| O14773 | Tripeptidyl-peptidase 1 | 62913.5 | 3 | 9.7 | 1.5 | 1.3 | 0.7 | 2 | 7.2 | 2.9 | 3.1 | 0.9 | 3 | 9.7 | 1.6 | 2.9 | 1.3 | 2 | 7.2 | 1.4 | 3.1 | 1.3 | 1.881 |
| P04179 | Superoxide dismutase [Mn], mitochondrial | 27055.1 | 6 | 29.7 | 1.4 | 1.2 | 1.5 | 5 | 28.3 | 1.4 | 1.7 | 2.1 | 5 | 28.3 | 1.9 | 2.6 | 2.6 | 8 | 36.4 | 1.8 | 2.4 | 1.7 | 1.867 |
| P22392 | Nucleoside diphosphate kinase B | 19285.8 | 6 | 50 | 1.5 | 2.6 | 1.2 | 4 | 41.4 | 1.4 | 1.0 | 1.5 | 8 | 55.2 | 2.4 | 3.2 | 1.6 | 6 | 50 | 2.2 | 2.0 | 1.8 | 1.866 |
| P20618 | Proteasome subunit beta type-1 | 28591.1 | 4 | 23.6 | 1.5 | 1.6 | 1.5 | 4 | 25.3 | 3.1 | 1.7 | 2.0 | 3 | 21.5 | 1.6 | 1.5 | 1.4 | 5 | 31.5 | 2.1 | 2.3 | 1.1 | 1.865 |
| O60547 | GDP-mannose 4,6 dehydratase | 46039.3 | 2 | 4.5 | 0.8 | 2.0 | 1.7 | * | * | * | * | * | 2 | 7.5 | 1.8 | 1.9 | 1.4 | 2 | 3.7 | 3.7 | 1.4 | 1.4 | 1.852 |
| Q12905 | Interleukin enhancer-binding factor 2 | 45740.2 | 5 | 15.8 | 1.5 | 2.2 | 1.3 | 3 | 10.5 | 1.4 | 1.1 | 1.9 | 6 | 20.2 | 2.4 | 2.2 | 1.6 | 4 | 13.3 | 2.8 | 1.9 | 2.2 | 1.851 |
| P13639 | Elongation factor 2 | 105676.4 | 13 | 20.8 | 1.2 | 2.2 | 1.2 | 9 | 17 | 1.7 | 1.1 | 1.6 | 16 | 24.2 | 2.1 | 3.0 | 1.8 | 17 | 27.7 | 2.2 | 2.2 | 1.5 | 1.850 |
| P60174 | Triosephosphate isomerase | 33958.8 | 13 | 45.8 | 1.3 | 2.3 | 1.3 | 12 | 43.3 | 1.7 | 1.2 | 1.5 | 13 | 45.8 | 2.0 | 3.1 | 1.8 | 13 | 45.8 | 2.1 | 2.1 | 1.5 | 1.849 |
| P50995 | Annexin A11 | 58190.8 | 2 | 4.5 | 0.9 | 1.4 | 0.7 | * | * | * | * | * | 3 | 8.3 | 3.6 | 2.8 | 1.4 | 2 | 4.5 | 1.3 | 2.7 | 1.9 | 1.845 |
| Q9Y2Q3 | Glutathione S-transferase kappa 1 | 27484.5 | 2 | 12.8 | 0.8 | 2.0 | 1.0 | 2 | 12.8 | 1.5 | 1.2 | 1.4 | 5 | 28.7 | 4.1 | 3.4 | 2.2 | 2 | 13.7 | 1.1 | 1.6 | 1.3 | 1.844 |
| P62937 | Peptidyl-prolyl cis-trans isomerase A | 20258.5 | 9 | 40 | 1.3 | 2.5 | 1.2 | 7 | 36.3 | 1.7 | 1.0 | 1.3 | 9 | 40 | 2.6 | 3.2 | 2.0 | 9 | 40 | 1.9 | 1.5 | 1.6 | 1.839 |
| Q06210 | Glutamine--fructose-6-phosphate aminotransferase [isomerizing] 1 | 86090.7 | 4 | 8.2 | 1.2 | 1.6 | 0.6 | 3 | 6.1 | 2.6 | 3.2 | 2.9 | 4 | 7.7 | 1.6 | 1.7 | 1.0 | 4 | 7.4 | 2.4 | 1.3 | 1.1 | 1.825 |
| P78371 | T-complex protein 1 subunit beta | 63163.2 | 4 | 12.1 | 1.1 | 1.9 | 0.5 | 2 | 6.5 | 3.3 | 2.4 | 1.1 | 8 | 23.1 | 1.9 | 3.1 | 1.5 | 6 | 17.3 | 1.6 | 1.7 | 1.5 | 1.824 |
| Q13162 | Peroxiredoxin-4 | 32641.5 | 7 | 30.6 | 1.3 | 2.0 | 1.4 | 4 | 17.3 | 1.7 | 1.0 | 1.2 | 4 | 17.3 | 2.2 | 2.3 | 1.8 | 6 | 25.8 | 2.8 | 2.5 | 2.0 | 1.822 |
| P10599 | Thioredoxin | 13752.4 | 6 | 42.8 | 1.0 | 2.3 | 1.8 | 4 | 40.9 | 1.3 | 0.7 | 1.3 | 6 | 42.8 | 1.6 | 3.0 | 1.8 | 6 | 42.8 | 3.2 | 2.1 | 1.7 | 1.820 |
| P06454 | Prothymosin alpha | 13356 | 4 | 18 | 0.5 | 2.2 | 0.9 | 3 | 17.1 | 3.7 | 1.6 | 3.4 | 3 | 17.1 | 1.3 | 1.3 | 0.9 | 4 | 23.4 | 2.1 | 2.1 | 1.5 | 1.818 |
| O75390 | Citrate synthase, mitochondrial | 55687.7 | 4 | 9.8 | 1.0 | 2.4 | 0.7 | 3 | 6.6 | 1.4 | 1.2 | 2.3 | 6 | 15 | 2.8 | 3.7 | 1.8 | 3 | 8.3 | 1.2 | 1.4 | 1.4 | 1.817 |
| P23396 | 40S ribosomal protein S3gi\|91207641\|sp\|Q3T169.1\|RS3_BOVIN RecName | 29742.1 | 4 | 20.9 | 1.4 | 1.7 | 1.3 | 3 | 17.2 | 1.8 | 0.9 | 0.8 | 5 | 26.3 | 3.0 | 2.9 | 1.9 | 5 | 28.3 | 2.8 | 1.4 | 1.4 | 1.814 |
| Q07955 | Serine/arginine-rich splicing factor 1 | 29011.4 | 3 | 12.5 | 1.5 | 2.5 | 1.6 | 2 | 8 | 1.4 | 1.5 | 1.1 | 6 | 24.1 | 2.4 | 2.6 | 2.1 | 4 | 20.5 | 1.9 | 1.5 | 1.7 | 1.812 |
| P30085 | UMP-CMP kinase | 25333.2 | 7 | 46.4 | 0.9 | 2.3 | 1.2 | * | * | * | * | * | 6 | 45.9 | 2.4 | 3.4 | 2.0 | 4 | 30.6 | 0.6 | 1.6 | 0.9 | 1.802 |
| O00299 | Chloride intracellular channel protein 1 | 29859.3 | 6 | 35.6 | 1.8 | 2.2 | 0.7 | 4 | 27.3 | 1.2 | 0.9 | 1.9 | 5 | 30.7 | 2.7 | 2.2 | 2.0 | 7 | 39 | 2.6 | 1.7 | 1.9 | 1.801 |
| P30044 | Peroxiredoxin-5, mitochondrial | 24188.2 | 6 | 31.7 | 1.1 | 2.7 | 0.8 | 6 | 31.7 | 1.8 | 0.9 | 1.0 | 7 | 36.9 | 4.3 | 3.2 | 2.0 | 5 | 23.8 | 0.8 | 1.2 | 1.9 | 1.800 |
| P35268 | 60S ribosomal protein L22 | 17582.7 | 3 | 30.4 | 1.5 | 2.1 | 0.9 | 2 | 18.7 | 1.3 | 1.2 | 0.9 | 2 | 18.7 | 2.5 | 3.1 | 3.0 | 2 | 18.7 | 1.9 | 1.5 | 1.4 | 1.800 |
| O95336 | 6-phosphogluconolactonase | 28984.9 | * | * | * | * | * | 3 | 18.9 | 2.0 | 2.2 | 0.9 | 4 | 25.1 | 1.7 | 2.2 | 1.9 | 4 | 25.1 | 1.9 | 1.6 | 1.1 | 1.798 |
| Q9HC38 | Glyoxalase domain-containing protein 4 | 38796.1 | 3 | 9.9 | 1.8 | 2.5 | 2.3 | 2 | 6.3 | 2.4 | 1.6 | 1.5 | 3 | 9.9 | 1.5 | 2.2 | 0.9 | 3 | 9.9 | 1.3 | 1.7 | 1.7 | 1.795 |
| Q99879 | Histone H2B type 1-M | 16872.1 | 6 | 46.8 | 1.7 | 1.5 | 0.6 | 8 | 53.1 | 2.5 | 1.4 | 1.1 | 7 | 46.8 | 4.1 | 1.9 | 2.0 | 7 | 46.8 | 1.4 | 1.6 | 1.4 | 1.794 |
| P09972 | Fructose-bisphosphate aldolase C | 42881.8 | 3 | 9 | 1.4 | 1.6 | 1.7 | 5 | 15.1 | 1.9 | 1.1 | 1.7 | 4 | 13.4 | 1.9 | 3.0 | 1.6 | 7 | 21.1 | 2.3 | 1.6 | 1.3 | 1.794 |
| P13667 | Protein disulfide-isomerase A4 | 82643.4 | 16 | 31.6 | 1.1 | 2.2 | 1.3 | 9 | 17 | 1.3 | 1.2 | 1.3 | 16 | 28.5 | 2.6 | 3.0 | 1.9 | 14 | 26.2 | 1.9 | 1.9 | 1.6 | 1.792 |
| O14818 | Proteasome subunit alpha type-7 | 31228.9 | 4 | 21.3 | 1.3 | 3.1 | 2.2 | 3 | 18.5 | 1.6 | 1.0 | 1.8 | 4 | 25.4 | 2.2 | 1.7 | 1.5 | 6 | 33 | 1.9 | 1.5 | 1.0 | 1.788 |
| P27797 | Calreticulin | 54366.3 | 9 | 23.2 | 1.3 | 1.7 | 1.3 | 6 | 21.5 | 1.3 | 1.4 | 1.4 | 8 | 23.2 | 2.6 | 2.8 | 2.0 | 9 | 22.5 | 1.6 | 2.2 | 1.7 | 1.785 |
| Q14152 | Eukaryotic translation initiation factor 3 subunit A | 179363.1 | 2 | 2 | 1.7 | 2.8 | 1.3 | * | * | * | * | * | 3 | 2.7 | 1.8 | 3.2 | 1.7 | 2 | 2 | 0.6 | 1.3 | 0.8 | 1.779 |
| P20700 | Lamin-B1 | 72314.5 | 4 | 9 | 1.4 | 1.6 | 1.5 | 2 | 3.9 | 1.7 | 0.9 | 1.9 | 2 | 3.5 | 2.0 | 2.2 | 1.6 | 5 | 10.5 | 2.8 | 1.9 | 1.6 | 1.778 |
| P29966 | Myristoylated alanine-rich C-kinase substrate | 35761.6 | 3 | 20.7 | 1.0 | 0.9 | 0.8 | 5 | 28.6 | 1.5 | 0.7 | 2.1 | * | * | * | * | * | 3 | 15.6 | 4.2 | 3.0 | 2.4 | 1.774 |
| P62249 | 40S ribosomal protein S16gi\|54039370\|sp\|P62250.2\|RS16_RAT RecName | 18865.6 | 2 | 12.3 | 1.5 | 3.7 | 0.7 | 2 | 12.3 | 1.1 | 0.8 | 0.6 | 3 | 19.8 | 2.0 | 4.2 | 2.1 | 2 | 11.6 | 0.7 | 2.1 | 1.5 | 1.774 |
| P51659 | Peroxisomal multifunctional enzyme type 2 | 88069.8 | 3 | 8.1 | 1.5 | 2.1 | 0.9 | 2 | 5.9 | 1.4 | 0.6 | 2.1 | 4 | 9.7 | 2.9 | 2.9 | 1.8 | 4 | 9.7 | 1.5 | 1.7 | 1.9 | 1.772 |
| P52597 | Heterogeneous nuclear ribonucleoprotein F | 48319.8 | 5 | 18.3 | 1.2 | 2.0 | 1.5 | 4 | 14.4 | 1.7 | 1.2 | 2.0 | * | * | * | * | * | 5 | 16.3 | 2.7 | 1.8 | 1.7 | 1.772 |
| Q92499 | ATP-dependent RNA helicase DDX1 | 92338.1 | 2 | 5.6 | 1.2 | 4.4 | 1.6 | * | * | * | * | * | 3 | 7.9 | 1.5 | 3.1 | 0.6 | 3 | 7.9 | 0.8 | 0.9 | 1.2 | 1.769 |
| Q8WU39 | Marginal zone B- and B1-cell-specific protein | 21613 | 2 | 20.1 | 1.0 | 1.5 | 1.5 | 2 | 10.5 | 1.5 | 0.9 | 1.2 | 3 | 26.9 | 4.1 | 2.6 | 1.5 | 2 | 20.1 | 1.7 | 1.8 | 1.4 | 1.765 |
| P12429 | Annexin A3 | 40293.8 | 3 | 17 | 0.6 | 2.7 | 2.5 | 2 | 7.1 | 1.6 | 1.3 | 1.5 | * | * | * | * | * | 2 | 9.5 | 2.7 | 1.2 | 1.6 | 1.762 |
| P55072 | Transitional endoplasmic reticulum ATPase | 96780.1 | 17 | 24 | 1.3 | 2.4 | 1.5 | 11 | 18.4 | 1.6 | 1.3 | 1.2 | 16 | 23.8 | 2.0 | 3.5 | 1.5 | 16 | 27.7 | 1.5 | 1.6 | 1.5 | 1.759 |
| Q15084 | Protein disulfide-isomerase A6 | 52700.3 | 9 | 31.5 | 1.1 | 1.7 | 1.3 | 6 | 20.2 | 1.6 | 1.1 | 1.5 | 10 | 33.6 | 2.6 | 2.2 | 1.7 | 10 | 33.8 | 2.4 | 2.1 | 1.3 | 1.753 |
| P07858 | Cathepsin B | 40953.1 | 2 | 7.6 | 1.2 | 1.4 | 0.6 | * | * | * | * | * | 2 | 7.6 | 2.4 | 2.2 | 1.2 | 2 | 7.6 | 1.5 | 3.5 | 2.3 | 1.751 |
| P62263 | 40S ribosomal protein S14gi\|50403753\|sp\|P62264.3\|RS14_MOUSE RecName | 18173.4 | 2 | 23.8 | 0.9 | 1.8 | 1.3 | 2 | 15.2 | 1.5 | 0.6 | 1.1 | 2 | 15.8 | 2.7 | 3.5 | 2.4 | * | * | * | * | * | 1.751 |
| P28838 | Cytosol aminopeptidase | 61754.3 | 6 | 17.1 | 1.5 | 1.6 | 1.4 | 5 | 14 | 1.5 | 1.1 | 1.9 | 7 | 18.3 | 1.8 | 2.8 | 1.2 | 10 | 29.2 | 1.8 | 2.6 | 1.7 | 1.748 |
| P35232 | Prohibitingi\|88909243\|sp\|Q3T165.1\|PHB_BOVIN RecName | 31590.4 | 7 | 39.3 | 1.2 | 2.2 | 1.2 | 3 | 19.1 | 2.1 | 0.9 | 1.0 | 9 | 49.6 | 2.4 | 3.0 | 1.7 | 4 | 23.8 | 2.0 | 1.5 | 1.2 | 1.745 |
| P14550 | Alcohol dehydrogenase [NADP(+)] | 39797.8 | 2 | 6.4 | 1.5 | 2.4 | 1.6 | 2 | 6.4 | 1.3 | 1.0 | 1.1 | 2 | 6.4 | 1.7 | 2.1 | 1.4 | 3 | 9.8 | 2.3 | 2.8 | 1.2 | 1.741 |
| P40227 | T-complex protein 1 subunit zeta | 64966.5 | 4 | 12.9 | 1.1 | 2.0 | 0.9 | 4 | 9.7 | 1.4 | 0.6 | 1.3 | 4 | 9.6 | 2.1 | 3.7 | 3.1 | 7 | 17.1 | 1.1 | 1.8 | 1.7 | 1.739 |
| Q14974 | Importin subunit beta-1 | 104679.3 | 4 | 5.1 | 1.6 | 2.5 | 0.9 | 2 | 3.4 | 1.2 | 0.8 | 0.6 | 3 | 5.2 | 2.1 | 4.8 | 2.5 | 4 | 5.9 | 0.8 | 1.3 | 0.7 | 1.737 |
| P22234 | Multifunctional protein ADE2 | 52865.5 | 5 | 16 | 1.1 | 2.9 | 1.0 | * | * | * | * | * | 2 | 7.2 | 1.7 | 2.1 | 1.5 | 3 | 10.3 | 2.1 | 1.5 | 1.2 | 1.733 |
| O60506 | Heterogeneous nuclear ribonucleoprotein Q | 76460.7 | 3 | 7.2 | 1.1 | 3.3 | 1.5 | 3 | 5.7 | 1.0 | 1.5 | 1.4 | 8 | 15.8 | 1.9 | 2.5 | 1.3 | 6 | 13 | 1.7 | 1.8 | 1.2 | 1.729 |
| P09622 | Dihydrolipoyl dehydrogenase, mitochondrial | 60224.7 | 3 | 8 | 1.3 | 1.8 | 1.0 | * | * | * | * | * | 4 | 10 | 1.8 | 3.3 | 2.0 | 2 | 5.8 | 1.5 | 1.2 | 1.4 | 1.728 |
| P49368 | T-complex protein 1 subunit gamma | 66293 | 5 | 9.3 | 1.2 | 2.3 | 0.5 | * | * | * | * | * | 6 | 14.4 | 1.9 | 2.8 | 1.4 | 3 | 5.3 | 2.1 | 1.7 | 1.7 | 1.726 |
| Q01082 | Spectrin beta chain, non-erythrocytic 1 | 301206.7 | 11 | 6.6 | 1.0 | 1.6 | 1.0 | * | * | * | * | * | 7 | 5.3 | 2.0 | 2.6 | 2.1 | 13 | 8.8 | 2.1 | 1.4 | 1.2 | 1.726 |
| P23381 | Tryptophan--tRNA ligase, cytoplasmic | 58552.2 | 2 | 7.8 | 1.0 | 1.5 | 1.0 | 2 | 7.8 | 1.9 | 1.3 | 1.1 | 2 | 7.8 | 1.9 | 2.1 | 1.5 | 7 | 22.9 | 3.5 | 2.1 | 1.8 | 1.725 |
| Q15149 | Plectin | 569239.9 | 4 | 1.2 | 1.2 | 1.5 | 1.1 | 2 | 0.5 | 1.1 | 2.2 | 2.1 | 7 | 1.7 | 2.0 | 2.0 | 1.6 | 10 | 2.3 | 2.3 | 1.9 | 1.6 | 1.724 |
| P31946 | 14-3-3 protein beta/alpha | 31079.1 | 8 | 38.2 | 1.1 | 2.0 | 0.9 | 7 | 28.4 | 1.7 | 1.1 | 1.4 | 16 | 65 | 2.5 | 3.1 | 2.0 | 12 | 60.1 | 1.5 | 1.7 | 1.5 | 1.724 |
| Q16851 | UTP--glucose-1-phosphate uridylyltransferase | 63020.7 | 4 | 8.8 | 1.2 | 1.9 | 1.0 | * | * | * | * | * | 3 | 7.6 | 2.0 | 3.3 | 1.8 | 4 | 11.2 | 1.2 | 1.4 | 1.1 | 1.711 |
| P42704 | Leucine-rich PPR motif-containing protein, mitochondrial | 173804 | 5 | 4.1 | 1.2 | 2.4 | 1.5 | 5 | 4 | 1.0 | 0.6 | 0.9 | 11 | 9.3 | 2.9 | 2.7 | 1.8 | 8 | 7.8 | 2.2 | 1.6 | 1.6 | 1.708 |
| P18124 | 60S ribosomal protein L7 | 34183.5 | 7 | 29.4 | 1.5 | 2.7 | 1.1 | 3 | 17.3 | 1.5 | 0.9 | 1.5 | 5 | 25 | 2.0 | 2.5 | 1.5 | 5 | 25.4 | 2.0 | 1.6 | 1.2 | 1.705 |
| P05387 | 60S acidic ribosomal protein P2 | 13106.3 | 5 | 70.4 | 1.3 | 2.1 | 0.9 | 5 | 70.4 | 1.7 | 0.9 | 1.7 | 4 | 69.5 | 2.5 | 2.6 | 1.2 | 4 | 69.5 | 2.3 | 1.6 | 2.0 | 1.704 |
| P17931 | Galectin-3 | 27362.1 | 7 | 31.2 | 1.1 | 1.9 | 1.3 | 5 | 24 | 1.4 | 0.8 | 2.2 | 5 | 22.8 | 1.7 | 2.7 | 1.0 | 6 | 28.4 | 2.5 | 2.2 | 1.6 | 1.703 |
| P27824 | Calnexin | 75952 | 6 | 11.1 | 1.5 | 2.1 | 1.3 | 4 | 8.6 | 1.5 | 1.0 | 1.5 | 8 | 15.8 | 1.9 | 2.7 | 1.6 | 7 | 14.8 | 1.7 | 1.8 | 1.5 | 1.700 |
| Q14764 | Major vault protein | 105808.9 | 4 | 10.4 | 1.9 | 1.5 | 1.2 | 2 | 3.2 | 2.1 | 0.9 | 1.1 | 8 | 17.6 | 1.8 | 2.9 | 2.1 | 7 | 12.9 | 1.4 | 1.6 | 1.5 | 1.697 |
| P11216 | Glycogen phosphorylase, brain form | 105451.5 | 4 | 6.1 | 1.1 | 1.4 | 0.7 | * | * | * | * | * | 6 | 9.8 | 1.9 | 2.5 | 1.8 | 5 | 7.5 | 2.2 | 2.0 | 0.9 | 1.691 |
| P06744 | Glucose-6-phosphate isomerase | 68419.6 | 10 | 24 | 1.1 | 2.0 | 1.1 | 9 | 24.1 | 2.1 | 1.1 | 1.4 | 12 | 32.2 | 1.5 | 2.9 | 1.6 | 18 | 44.8 | 2.1 | 1.6 | 1.3 | 1.685 |
| P13796 | Plastin-2 | 77344.6 | 9 | 20.8 | 2.1 | 1.9 | 1.2 | 9 | 20 | 1.1 | 1.3 | 1.5 | * | * | * | * | * | 15 | 31.2 | 2.1 | 2.3 | 1.6 | 1.682 |
| P54819 | Adenylate kinase 2, mitochondrial | 29444.4 | 4 | 28.4 | 1.3 | 1.3 | 1.1 | 2 | 17.1 | 2.0 | 2.0 | 0.7 | 4 | 24.2 | 2.1 | 2.4 | 1.5 | 3 | 23.8 | 2.2 | 1.9 | 1.5 | 1.681 |
| Q9GZV4 | Eukaryotic translation initiation factor 5A-2 | 19153.4 | * | * | * | * | * | 2 | 13 | 1.3 | 1.2 | 1.5 | 2 | 13 | 2.1 | 2.7 | 1.5 | 3 | 13.7 | 1.4 | 1.7 | 1.5 | 1.675 |
| P37802 | Transgelin-2 | 24292.1 | 6 | 38.1 | 2.2 | 3.2 | 1.3 | 5 | 31.1 | 1.3 | 1.0 | 1.6 | 8 | 49.7 | 1.0 | 1.1 | 1.0 | 3 | 21.6 | 2.0 | 2.7 | 2.0 | 1.675 |
| P30101 | Protein disulfide-isomerase A3 | 64100.1 | 19 | 33.6 | 1.2 | 2.1 | 1.3 | 15 | 35.4 | 1.7 | 1.1 | 1.3 | 17 | 35.4 | 2.7 | 2.5 | 1.6 | 19 | 38.2 | 1.3 | 1.7 | 1.4 | 1.670 |
| Q06323 | Proteasome activator complex subunit 1 | 33218.4 | 5 | 28.5 | 1.4 | 1.4 | 1.5 | 5 | 27.7 | 1.2 | 1.1 | 1.4 | 8 | 44.5 | 3.1 | 2.0 | 1.6 | 9 | 49.3 | 2.0 | 1.6 | 1.2 | 1.660 |
| Q9Y3I0 | tRNA-splicing ligase RtcB homolog | 60335.7 | 2 | 4.9 | 0.9 | 1.5 | 0.8 | * | * | * | * | * | 2 | 5.9 | 3.1 | 2.0 | 1.7 | 2 | 4.9 | 1.7 | 1.5 | 1.7 | 1.658 |
| P36871 | Phosphoglucomutase-1 | 66922.8 | 2 | 9.7 | 3.6 | 4.6 | 1.2 | * | * | * | * | * | 3 | 11.5 | 0.9 | 0.9 | 0.5 | 4 | 16 | 0.7 | 0.9 | 0.9 | 1.656 |
| P02788 | Lactotransferrin | 86694.4 | 4 | 8.4 | 1.4 | 1.5 | 0.8 | 10 | 16.6 | 2.4 | 1.0 | 1.6 | 5 | 11.2 | 2.1 | 1.7 | 1.3 | 7 | 14 | 2.9 | 1.4 | 1.4 | 1.654 |
| P00387 | NADH-cytochrome b5 reductase 3 | 36768.9 | 3 | 17.9 | 1.9 | 2.0 | 0.9 | 2 | 13.9 | 2.1 | 0.8 | 1.0 | 3 | 17.9 | 2.0 | 2.2 | 1.9 | 4 | 19.6 | 2.1 | 1.3 | 1.1 | 1.651 |
| O75874 | Isocitrate dehydrogenase [NADP] cytoplasmic | 52277.4 | 8 | 22.2 | 1.2 | 1.9 | 1.4 | 8 | 23.4 | 1.3 | 1.1 | 1.3 | 12 | 36.2 | 2.7 | 2.2 | 1.4 | 8 | 24.8 | 1.2 | 2.6 | 1.7 | 1.650 |
| P25786 | Proteasome subunit alpha type-1 | 31570.2 | 2 | 12.1 | 1.2 | 2.2 | 1.3 | * | * | * | * | * | 4 | 16.3 | 1.3 | 2.1 | 1.6 | 3 | 14.4 | 2.0 | 1.5 | 1.1 | 1.649 |
| O60664 | Perilipin-3 | 50705 | 2 | 6.6 | 1.3 | 1.6 | 0.8 | * | * | * | * | * | 4 | 13.8 | 2.0 | 3.0 | 2.1 | 3 | 14 | 0.7 | 1.6 | 0.7 | 1.648 |
| P23526 | Adenosylhomocysteinase | 52466.3 | 4 | 11.3 | 1.2 | 1.8 | 1.1 | 3 | 7.6 | 1.4 | 1.1 | 1.5 | 7 | 19.4 | 2.4 | 2.3 | 1.4 | 5 | 13.8 | 2.3 | 1.5 | 1.7 | 1.641 |
| P46777 | 60S ribosomal protein L5 | 39635.6 | 4 | 15.1 | 0.9 | 1.9 | 1.6 | 2 | 8 | 0.9 | 0.9 | 0.8 | 4 | 14.4 | 2.1 | 3.1 | 1.9 | 2 | 8 | 2.3 | 1.6 | 1.6 | 1.641 |
| P13804 | Electron transfer flavoprotein subunit alpha, mitochondrial | 39169.3 | 6 | 30 | 1.3 | 2.0 | 1.1 | 4 | 22.5 | 2.0 | 1.7 | 1.0 | 8 | 34.8 | 2.1 | 2.7 | 1.2 | 6 | 30 | 0.9 | 1.9 | 1.5 | 1.641 |
| Q14697 | Neutral alpha-glucosidase AB | 111220.6 | 11 | 15.7 | 1.1 | 1.6 | 0.8 | 8 | 12.3 | 2.1 | 1.6 | 1.4 | 9 | 14.6 | 2.7 | 2.3 | 1.6 | 14 | 18.9 | 1.5 | 1.4 | 1.4 | 1.640 |
| P26599 | Polypyrimidine tract-binding protein 1 | 62148.5 | 7 | 25.9 | 0.9 | 2.2 | 1.5 | 6 | 18.6 | 1.6 | 1.0 | 1.6 | 7 | 25.9 | 1.6 | 2.2 | 1.5 | 11 | 41.6 | 2.0 | 2.0 | 2.0 | 1.639 |
| Q99623 | Prohibitin-2 | 36034.7 | 4 | 18 | 1.3 | 1.9 | 1.0 | 3 | 13.7 | 1.5 | 0.8 | 1.5 | 7 | 29 | 2.7 | 2.9 | 1.4 | 5 | 21.7 | 1.1 | 1.7 | 1.4 | 1.632 |
| P24534 | Elongation factor 1-beta | 28105.8 | 3 | 16.4 | 1.1 | 2.5 | 0.8 | 3 | 16.4 | 2.2 | 1.3 | 0.7 | 4 | 19.5 | 2.3 | 2.4 | 1.4 | * | * | * | * | * | 1.625 |
| P52272 | Heterogeneous nuclear ribonucleoprotein M | 83565.8 | 3 | 4.5 | 0.9 | 1.6 | 1.1 | 2 | 2.7 | 1.8 | 1.3 | 2.0 | 8 | 15.8 | 1.9 | 2.6 | 1.5 | 4 | 7.6 | 1.2 | 1.9 | 1.6 | 1.613 |
| Q9NR45 | Sialic acid synthase | 45232 | 4 | 16.1 | 1.0 | 1.5 | 0.6 | * | * | * | * | * | 4 | 18.3 | 2.0 | 3.3 | 2.0 | 3 | 16.1 | 1.1 | 1.4 | 1.2 | 1.608 |
| Q9P2E9 | Ribosome-binding protein 1 | 178960.8 | 9 | 8.9 | 1.5 | 2.0 | 1.7 | 6 | 7.4 | 1.8 | 0.8 | 0.8 | 7 | 7.6 | 1.6 | 2.5 | 1.1 | 12 | 10.2 | 2.4 | 1.6 | 1.6 | 1.606 |
| Q08380 | Galectin-3-binding protein | 68981.6 | 3 | 7.1 | 1.4 | 1.8 | 1.3 | 3 | 7.1 | 2.8 | 1.1 | 1.1 | * | * | * | * | * | 3 | 7.1 | 1.3 | 2.2 | 1.3 | 1.605 |
| P50990 | T-complex protein 1 subunit theta | 66100.4 | 5 | 10.7 | 0.6 | 2.3 | 0.9 | * | * | * | * | * | 7 | 12.5 | 1.6 | 2.7 | 1.4 | 4 | 8.2 | 1.7 | 1.7 | 1.4 | 1.604 |
| Q16543 | Hsp90 co-chaperone Cdc37 | 50891.3 | * | * | * | * | * | 2 | 10 | 0.5 | 0.8 | 1.4 | 3 | 12.9 | 1.1 | 1.7 | 1.1 | 3 | 12.4 | 3.7 | 2.5 | 1.7 | 1.603 |
| P46940 | Ras GTPase-activating-like protein IQGAP1 | 209192.8 | 10 | 10.5 | 1.8 | 1.9 | 1.3 | 9 | 8 | 1.2 | 0.8 | 1.1 | 7 | 6.8 | 1.6 | 2.5 | 1.5 | 16 | 15.1 | 2.1 | 1.8 | 1.3 | 1.602 |
| P09874 | Poly [ADP-ribose] polymerase 1 | 132043.9 | 2 | 3.2 | 0.9 | 1.3 | 1.1 | * | * | * | * | * | 3 | 4.4 | 2.2 | 1.9 | 2.0 | 2 | 2.4 | 1.9 | 1.5 | 1.4 | 1.602 |
| Q15691 | Microtubule-associated protein RP/EB family member 1 | 33341.1 | 2 | 11.1 | 2.2 | 3.7 | 0.9 | 2 | 12.6 | 0.7 | 0.6 | 0.3 | 3 | 17.9 | 1.7 | 2.2 | 1.4 | 2 | 12.6 | 2.1 | 1.7 | 1.4 | 1.601 |
| P06733 | Alpha-enolase | 52988.3 | 18 | 43.7 | 1.5 | 1.7 | 0.7 | 18 | 43.5 | 1.7 | 1.1 | 1.1 | 17 | 47.2 | 2.2 | 2.6 | 1.8 | 19 | 44 | 1.3 | 1.9 | 1.3 | 1.595 |
| O43169 | Cytochrome b5 type B | 17743.3 | 2 | 8.9 | 1.1 | 1.5 | 1.4 | * | * | * | * | * | 2 | 8.9 | 1.9 | 1.6 | 1.1 | 2 | 8.9 | 2.4 | 1.7 | 1.9 | 1.594 |
| Q5VTE0 | Elongation factor 1-alpha 1 | 57445.8 | 14 | 28.1 | 0.9 | 2.3 | 0.8 | 12 | 27.9 | 1.4 | 1.1 | 0.8 | 13 | 29.4 | 2.6 | 3.2 | 1.5 | 13 | 28.5 | 1.3 | 1.7 | 1.3 | 1.594 |
| P51149 | Ras-related protein Rab-7agi\|46397834\|sp\|P51150.2\|RAB7A_MOUSE RecName | 26225.2 | * | * | * | * | * | 2 | 13 | 1.3 | 1.4 | 1.4 | 2 | 13 | 1.6 | 2.4 | 1.8 | 2 | 13 | 1.5 | 1.3 | 1.1 | 1.593 |
| Q06830 | Peroxiredoxin-1 | 25077.1 | * | * | * | * | * | 10 | 44.2 | 1.5 | 0.9 | 1.2 | 12 | 46.2 | 1.9 | 2.2 | 1.4 | 13 | 53.7 | 2.0 | 1.6 | 1.3 | 1.592 |
| P22314 | Ubiquitin-like modifier-activating enzyme 1 | 126571.1 | 12 | 15.3 | 1.5 | 2.2 | 1.0 | 11 | 17.8 | 1.4 | 0.9 | 1.1 | 17 | 25.2 | 2.2 | 2.8 | 1.6 | 15 | 21.1 | 1.2 | 1.7 | 1.1 | 1.592 |
| O60716 | Catenin delta-1 | 116523 | 5 | 6.9 | 1.0 | 2.2 | 1.0 | 2 | 2.8 | 1.5 | 2.4 | 1.1 | 3 | 4.2 | 2.2 | 2.3 | 1.4 | 3 | 4.2 | 0.9 | 1.4 | 0.9 | 1.591 |
| Q00610 | Clathrin heavy chain 1 | 207218.6 | 8 | 7.8 | 1.1 | 1.6 | 0.9 | 2 | 1.6 | 1.0 | 1.1 | 2.5 | 10 | 8.3 | 1.8 | 3.0 | 1.2 | 14 | 10.9 | 1.6 | 1.6 | 1.3 | 1.589 |
| P50395 | Rab GDP dissociation inhibitor beta | 56452.5 | 5 | 13.9 | 1.4 | 2.1 | 1.0 | 6 | 20.2 | 1.4 | 1.1 | 1.0 | 7 | 22.9 | 2.4 | 2.8 | 1.4 | 6 | 18.2 | 1.2 | 1.7 | 1.3 | 1.587 |
| P68036 | Ubiquitin-conjugating enzyme E2 L3 | 20627.2 | 2 | 24 | 1.1 | 1.7 | 1.0 | 2 | 15.5 | 1.6 | 1.2 | 0.7 | 2 | 24 | 2.6 | 3.2 | 2.0 | 3 | 29.8 | 1.2 | 1.2 | 1.1 | 1.586 |
| P14618 | Pyruvate kinase isozymes M1/M2 | 63840.1 | 21 | 44.4 | 1.7 | 2.0 | 1.0 | 16 | 27.1 | 1.6 | 1.5 | 1.0 | 16 | 31 | 1.4 | 2.5 | 1.6 | 20 | 42.9 | 1.2 | 1.9 | 1.6 | 1.584 |
| P07384 | Calpain-1 catalytic subunit | 87849.8 | 3 | 6 | 1.3 | 1.4 | 1.2 | 2 | 4.2 | 1.9 | 0.5 | 1.8 | 2 | 4.3 | 2.4 | 2.3 | 1.4 | 4 | 6.5 | 1.8 | 1.4 | 1.1 | 1.583 |
| Q8NBS9 | Thioredoxin domain-containing protein 5 | 52060.6 | 8 | 18.2 | 1.0 | 1.4 | 1.2 | 4 | 11.3 | 1.5 | 1.7 | 1.1 | 7 | 20.1 | 2.4 | 2.5 | 1.3 | 10 | 25.2 | 1.8 | 1.4 | 1.5 | 1.582 |
| P47756 | F-actin-capping protein subunit beta | 34518.3 | 3 | 13.3 | 0.9 | 1.0 | 0.8 | 2 | 8.6 | 1.2 | 1.3 | 1.5 | 2 | 8.3 | 1.6 | 2.7 | 2.6 | 4 | 20.5 | 1.8 | 1.9 | 1.4 | 1.576 |
| P29692 | Elongation factor 1-delta | 34118.5 | 5 | 23.8 | 1.4 | 2.3 | 0.9 | 3 | 13.1 | 1.6 | 0.9 | 1.4 | 7 | 30.9 | 2.1 | 2.4 | 1.6 | 5 | 23.8 | 1.5 | 1.3 | 1.6 | 1.571 |
| P12532 | Creatine kinase U-type, mitochondrial | 49885.9 | 6 | 16.3 | 0.7 | 1.8 | 1.5 | 2 | 7.4 | 1.4 | 0.8 | 1.9 | 4 | 10.5 | 1.5 | 4.0 | 1.2 | 3 | 7.4 | 1.2 | 1.0 | 1.2 | 1.556 |
| Q15907 | Ras-related protein Rab-11B | 26476.2 | 4 | 15.1 | 0.9 | 1.2 | 0.9 | * | * | * | * | * | 3 | 14.6 | 1.6 | 2.1 | 1.5 | 3 | 15.1 | 2.2 | 2.1 | 1.4 | 1.555 |
| P17987 | T-complex protein 1 subunit alpha | 66622.2 | * | * | * | * | * | 2 | 4.4 | 1.4 | 0.9 | 0.9 | 6 | 10.6 | 1.4 | 2.1 | 1.3 | 2 | 3.9 | 2.6 | 1.8 | 1.7 | 1.555 |
| P0CG48 | Polyubiquitin-C | 86118.9 | 5 | 6.8 | 1.1 | 1.7 | 1.0 | 3 | 4.9 | 1.6 | 1.0 | 1.6 | 5 | 5.6 | 1.8 | 2.6 | 1.6 | 6 | 10.6 | 1.7 | 1.4 | 1.2 | 1.554 |
| P35222 | Catenin beta-1 | 89870.7 | 4 | 7.5 | 0.6 | 1.6 | 0.8 | * | * | * | * | * | 2 | 3.7 | 3.0 | 2.0 | 2.3 | 2 | 3.7 | 1.0 | 1.2 | 1.4 | 1.551 |
| P10768 | S-formylglutathione hydrolase | 35147.1 | 3 | 18.4 | 1.1 | 1.3 | 1.0 | * | * | * | * | * | 4 | 28 | 2.6 | 2.1 | 1.2 | 3 | 23.4 | 1.4 | 1.6 | 1.3 | 1.550 |
| Q99714 | 3-hydroxyacyl-CoA dehydrogenase type-2 | 28736.5 | 2 | 17.2 | 1.4 | 1.7 | 0.9 | * | * | * | * | * | 4 | 25.6 | 2.0 | 2.3 | 1.7 | 2 | 10.7 | 1.2 | 1.2 | 1.3 | 1.549 |
| P08865 | 40S ribosomal protein SA | 34553.3 | 5 | 26.7 | 1.2 | 2.1 | 0.8 | 4 | 18.9 | 1.5 | 0.9 | 1.2 | 7 | 28.1 | 2.2 | 2.8 | 1.5 | 5 | 25 | 1.0 | 1.8 | 1.4 | 1.545 |
| P00505 | Aspartate aminotransferase, mitochondrial | 52096.6 | 4 | 13.2 | 0.8 | 1.2 | 0.7 | 3 | 11.3 | 1.7 | 1.3 | 1.1 | 4 | 14.8 | 2.5 | 3.2 | 1.8 | 4 | 13.4 | 0.8 | 1.8 | 1.2 | 1.539 |
| P61088 | Ubiquitin-conjugating enzyme E2 N | 18491.9 | 2 | 19.7 | 1.2 | 1.6 | 0.7 | 3 | 29.6 | 1.3 | 1.0 | 1.1 | 3 | 29.6 | 2.2 | 3.0 | 1.5 | 5 | 40.7 | 1.6 | 1.7 | 1.6 | 1.538 |
| P42166 | Lamina-associated polypeptide 2, isoform alpha | 83845.5 | 2 | 4.3 | 1.7 | 2.3 | 1.4 | * | * | * | * | * | 3 | 6.1 | 1.3 | 2.3 | 0.9 | 2 | 4.3 | 1.4 | 1.0 | 1.0 | 1.530 |
| P04075 | Fructose-bisphosphate aldolase A | 43623.7 | 11 | 40.6 | 1.0 | 1.5 | 0.9 | 15 | 40.1 | 1.5 | 0.9 | 1.2 | 14 | 46.7 | 1.7 | 2.9 | 1.5 | 17 | 50.2 | 2.2 | 1.5 | 1.3 | 1.530 |
| P02545 | Prelamin-A/C | 80189.6 | 16 | 30.1 | 1.3 | 1.3 | 1.1 | 16 | 30.1 | 1.0 | 1.1 | 1.6 | 15 | 29 | 2.0 | 2.3 | 1.3 | 21 | 39.4 | 2.1 | 1.5 | 1.2 | 1.530 |
| Q9Y4L1 | Hypoxia up-regulated protein 1 | 122661.5 | 2 | 3.4 | 1.6 | 1.1 | 0.4 | * | * | * | * | * | 5 | 7 | 2.8 | 1.9 | 1.2 | 4 | 4.7 | 2.2 | 1.0 | 1.0 | 1.529 |
| Q12864 | Cadherin-17 | 99103.9 | 8 | 16.2 | 0.9 | 2.2 | 0.9 | * | * | * | * | * | 7 | 15.3 | 1.9 | 2.8 | 1.3 | 8 | 16.5 | 0.9 | 1.2 | 1.2 | 1.526 |
| P31939 | Bifunctional purine biosynthesis protein PURH | 70461.9 | 2 | 5.7 | 0.6 | 1.5 | 0.6 | 2 | 4.7 | 1.9 | 0.7 | 1.9 | 4 | 8.7 | 2.4 | 2.7 | 1.8 | 3 | 5.5 | 1.6 | 1.0 | 1.2 | 1.525 |
| O00571 | ATP-dependent RNA helicase DDX3X | 78254.4 | 5 | 10.8 | 1.0 | 2.6 | 1.1 | * | * | * | * | * | 6 | 12.8 | 1.8 | 1.8 | 1.4 | 4 | 9.2 | 1.0 | 1.4 | 1.4 | 1.525 |
| Q15582 | Transforming growth factor-beta-induced protein ig-h3 | 80064.2 | 5 | 9.8 | 2.1 | 1.6 | 1.3 | 3 | 6.4 | 0.5 | 1.7 | 0.9 | 6 | 11.8 | 1.5 | 1.3 | 1.3 | 6 | 10.9 | 2.4 | 2.3 | 2.2 | 1.522 |
| P07195 | L-lactate dehydrogenase B chain | 40671.1 | 8 | 31.4 | 1.0 | 2.0 | 0.9 | 8 | 25.1 | 1.4 | 0.9 | 1.6 | 9 | 29.6 | 1.7 | 2.9 | 1.5 | 8 | 27.5 | 1.0 | 1.6 | 1.1 | 1.518 |
| P30153 | Serine/threonine-protein phosphatase 2A 65 kDa regulatory subunit A alpha isoform | 70719.1 | 5 | 12.7 | 1.3 | 1.5 | 0.7 | 3 | 6.2 | 1.3 | 1.0 | 1.6 | 4 | 10 | 2.3 | 2.6 | 1.4 | 4 | 11.3 | 1.5 | 1.5 | 1.2 | 1.516 |
| Q14204 | Cytoplasmic dynein 1 heavy chain 1 | 577373.8 | 8 | 2.3 | 0.9 | 1.5 | 1.0 | 3 | 1.1 | 1.2 | 0.7 | 1.3 | 5 | 1.5 | 1.9 | 2.5 | 1.0 | 7 | 2.3 | 2.7 | 1.9 | 1.6 | 1.514 |
| P48047 | ATP synthase subunit O, mitochondrial | 25985.8 | 4 | 21.5 | 1.1 | 1.9 | 1.0 | * | * | * | * | * | 5 | 31.9 | 2.1 | 2.7 | 2.0 | 2 | 12.2 | 0.7 | 0.5 | 0.8 | 1.503 |
| P05388 | 60S acidic ribosomal protein P0 | 37183 | 6 | 29.3 | 1.2 | 2.6 | 1.1 | 4 | 20.5 | 1.2 | 1.0 | 0.9 | 5 | 23.9 | 1.7 | 2.5 | 1.3 | * | * | * | * | * | 1.500 |
| P07355 | Annexin A2 | 43444.5 | 24 | 59.2 | 1.5 | 1.9 | 1.1 | 18 | 54.2 | 1.2 | 1.0 | 1.0 | 21 | 58.4 | 2.1 | 2.4 | 1.5 | 20 | 55.4 | 1.3 | 1.6 | 1.2 | 1.495 |
| P09211 | Glutathione S-transferase P | 25313.5 | 5 | 38 | 1.2 | 1.1 | 1.2 | 5 | 38 | 1.4 | 1.3 | 1.1 | 5 | 38 | 2.8 | 1.8 | 1.5 | 7 | 50.4 | 1.1 | 1.9 | 1.4 | 1.492 |
| P04406 | Glyceraldehyde-3-phosphate dehydrogenase | 39971.7 | 17 | 48 | 1.4 | 2.1 | 0.7 | 12 | 41.7 | 1.6 | 1.2 | 0.6 | 17 | 46.2 | 1.7 | 3.0 | 1.5 | 18 | 48 | 0.9 | 1.6 | 1.2 | 1.487 |
| P04632 | Calpain small subunit 1 | 29783.8 | 2 | 27.9 | 1.9 | 1.5 | 0.7 | * | * | * | * | * | 2 | 27.9 | 2.1 | 0.9 | 1.9 | 2 | 27.9 | 1.5 | 1.5 | 0.9 | 1.484 |
| P55084 | Trifunctional enzyme subunit beta, mitochondrial | 56624.2 | 4 | 7.3 | 1.2 | 1.7 | 1.2 | * | * | * | * | * | 7 | 11.3 | 1.7 | 2.0 | 1.1 | 6 | 10.5 | 1.0 | 2.0 | 1.4 | 1.481 |
| P04206 | Ig kappa chain V-III region GOL | 12376.7 | 2 | 30.2 | 1.3 | 1.0 | 0.6 | 3 | 36.6 | 3.1 | 1.3 | 1.7 | 2 | 30.2 | 1.5 | 1.5 | 1.4 | 2 | 30.2 | 1.1 | 1.6 | 0.9 | 1.480 |
| P30040 | Endoplasmic reticulum resident protein 29 | 32942.1 | 2 | 10.3 | 1.0 | 1.7 | 0.8 | 3 | 14.1 | 1.5 | 1.0 | 0.7 | 2 | 8.4 | 2.0 | 3.3 | 1.8 | 3 | 16 | 0.9 | 1.6 | 1.2 | 1.480 |
| P35527 | Keratin, type I cytoskeletal 9 | 66039.5 | 5 | 17.1 | 1.2 | 1.3 | 1.0 | 7 | 20.5 | 1.1 | 1.1 | 1.7 | 4 | 12.5 | 2.1 | 2.0 | 1.6 | 6 | 15 | 1.7 | 1.5 | 1.5 | 1.478 |
| P18669 | Phosphoglycerate mutase 1 | 31512.3 | 9 | 47.2 | 1.3 | 1.3 | 1.0 | 5 | 32.2 | 1.4 | 1.0 | 1.3 | 9 | 44.8 | 1.9 | 2.4 | 1.3 | 9 | 44 | 2.0 | 1.4 | 1.3 | 1.471 |
| P30837 | Aldehyde dehydrogenase X, mitochondrial | 62130.5 | 8 | 21 | 1.0 | 1.3 | 0.5 | 9 | 23.7 | 1.8 | 1.4 | 0.7 | 9 | 20.8 | 2.1 | 3.1 | 1.8 | 5 | 14.3 | 0.9 | 1.5 | 1.2 | 1.464 |
| Q16836 | Hydroxyacyl-coenzyme A dehydrogenase, mitochondrial | 38818.8 | 5 | 27.7 | 0.9 | 2.3 | 1.3 | 3 | 11.1 | 1.7 | 1.2 | 1.2 | 5 | 28.9 | 2.2 | 1.9 | 1.5 | 5 | 36.3 | 0.6 | 1.4 | 1.0 | 1.462 |
| O43809 | Cleavage and polyadenylation specificity factor subunit 5 | 28302 | 2 | 19.3 | 1.7 | 2.4 | 1.6 | 3 | 15.8 | 0.6 | 1.0 | 1.1 | * | * | * | * | * | 3 | 23.7 | 1.7 | 1.7 | 1.6 | 1.462 |
| Q13148 | TAR DNA-binding protein 43 | 47964.5 | 2 | 8.6 | 0.6 | 1.2 | 0.7 | * | * | * | * | * | 3 | 11.5 | 1.8 | 2.2 | 2.1 | 2 | 8.6 | 1.6 | 1.5 | 1.2 | 1.462 |
| Q16181 | Septin-11 | 57595.4 | * | * | * | * | * | 2 | 5.9 | 1.0 | 1.2 | 1.1 | 2 | 5.9 | 1.5 | 2.4 | 1.5 | 4 | 13.5 | 1.3 | 1.6 | 1.3 | 1.461 |
| P30050 | 60S ribosomal protein L12gi\|47117092\|sp\|P61284.1\|RL12_BOVIN RecName | 20151.7 | 2 | 18.7 | 0.8 | 1.6 | 0.8 | * | * | * | * | * | 3 | 24.2 | 2.0 | 2.0 | 1.4 | 2 | 18.7 | 1.8 | 1.4 | 1.5 | 1.458 |
| Q86VP6 | Cullin-associated NEDD8-dissociated protein 1 | 149328.9 | 6 | 8.2 | 1.0 | 1.6 | 0.7 | 3 | 3.1 | 1.3 | 0.9 | 0.9 | 8 | 10.2 | 2.6 | 2.1 | 2.0 | 8 | 12 | 1.3 | 1.5 | 1.3 | 1.453 |
| P40939 | Trifunctional enzyme subunit alpha, mitochondrial | 93974.8 | 9 | 19.1 | 1.0 | 1.9 | 0.8 | 8 | 13.3 | 1.6 | 1.0 | 1.0 | 13 | 20.8 | 2.5 | 2.5 | 1.6 | 11 | 17.8 | 0.9 | 1.1 | 1.0 | 1.449 |
| Q99497 | Protein DJ-1 | 22368.3 | 4 | 26.4 | 1.4 | 1.4 | 0.8 | 2 | 21.6 | 1.2 | 1.7 | 1.0 | 5 | 33.3 | 1.9 | 2.2 | 1.7 | 4 | 30.6 | 1.6 | 1.1 | 1.5 | 1.447 |
| P52565 | Rho GDP-dissociation inhibitor 1 | 26002.7 | 2 | 15.1 | 0.9 | 2.2 | 0.8 | 2 | 15.1 | 1.0 | 0.9 | 1.9 | * | * | * | * | * | 2 | 14.2 | 1.7 | 2.1 | 0.6 | 1.446 |
| P55786 | Puromycin-sensitive aminopeptidase | 112175.8 | 4 | 6.3 | 1.0 | 2.1 | 0.7 | 2 | 2.9 | 1.3 | 1.2 | 0.8 | 7 | 11.9 | 1.8 | 2.5 | 1.4 | 5 | 7.8 | 1.5 | 1.6 | 1.3 | 1.445 |
| P26639 | Threonine--tRNA ligase, cytoplasmic | 93139.8 | 2 | 4.5 | 0.8 | 1.2 | 1.6 | * | * | * | * | * | 2 | 3 | 1.6 | 1.7 | 1.1 | 3 | 5.8 | 2.0 | 1.5 | 1.2 | 1.445 |
| P05787 | Keratin, type II cytoskeletal 8 | 58316.2 | 34 | 65.4 | 1.1 | 1.5 | 1.0 | * | * | * | * | * | 34 | 68.5 | 1.8 | 2.1 | 1.2 | 29 | 55.6 | 1.4 | 1.3 | 1.3 | 1.445 |
| P00367 | Glutamate dehydrogenase 1, mitochondrial | 66496.2 | 3 | 7.3 | 1.2 | 1.4 | 0.9 | * | * | * | * | * | 6 | 14.8 | 1.9 | 2.3 | 1.4 | 5 | 14.8 | 1.0 | 1.4 | 1.0 | 1.442 |
| P04844 | Dolichyl-diphosphooligosaccharide--protein glycosyltransferase subunit 2 | 73577.3 | 4 | 15.5 | 0.9 | 1.3 | 0.8 | * | * | * | * | * | 6 | 18.7 | 1.4 | 2.3 | 1.5 | 5 | 18.3 | 1.3 | 2.1 | 1.4 | 1.441 |
| P00441 | Superoxide dismutase [Cu-Zn] | 17749.4 | 3 | 17.5 | 1.0 | 1.8 | 1.3 | 3 | 22.7 | 1.6 | 0.8 | 1.4 | 3 | 22.7 | 1.5 | 2.0 | 1.7 | 2 | 13.6 | 1.4 | 1.3 | 1.0 | 1.440 |
| Q99832 | T-complex protein 1 subunit eta | 65789.4 | 2 | 3.6 | 0.9 | 1.9 | 0.7 | 3 | 6.8 | 1.4 | 1.1 | 1.3 | 4 | 8.8 | 2.1 | 2.4 | 1.3 | 4 | 8.6 | 1.3 | 1.3 | 1.5 | 1.436 |
| Q92945 | Far upstream element-binding protein 2 | 77868 | 2 | 3.6 | 0.9 | 1.3 | 0.7 | 3 | 8.7 | 1.4 | 0.7 | 1.3 | 7 | 15.7 | 2.2 | 2.2 | 2.2 | 5 | 11.8 | 1.5 | 1.3 | 1.2 | 1.433 |
| Q13011 | Delta(3,5)-Delta(2,4)-dienoyl-CoA isomerase, mitochondrial | 38608.5 | 3 | 16.1 | 1.5 | 1.2 | 1.1 | 2 | 7.9 | 1.6 | 1.8 | 1.4 | 3 | 16.1 | 1.5 | 2.3 | 1.8 | 2 | 11.5 | 1.0 | 0.6 | 1.0 | 1.429 |
| P35908 | Keratin, type II cytoskeletal 2 epidermal | 70762.4 | 5 | 13.3 | 1.1 | 1.4 | 0.9 | * | * | * | * | * | 5 | 10.3 | 1.8 | 2.1 | 1.4 | 10 | 21.4 | 1.2 | 1.4 | 1.4 | 1.427 |
| Q13813 | Spectrin alpha chain, non-erythrocytic 1 | 313875.5 | 18 | 10.8 | 1.0 | 1.7 | 1.2 | 5 | 3.1 | 1.5 | 1.0 | 1.1 | 12 | 6.3 | 1.4 | 2.1 | 1.5 | 20 | 13.2 | 1.7 | 1.3 | 1.0 | 1.415 |
| P05155 | Plasma protease C1 inhibitor | 59561.9 | 4 | 13.6 | 1.4 | 1.6 | 1.3 | * | * | * | * | * | 2 | 5.4 | 1.7 | 1.9 | 1.4 | 2 | 5.8 | 0.8 | 1.2 | 1.1 | 1.410 |
| P02751 | Fibronectin | 277458.2 | 9 | 6.9 | 2.1 | 1.3 | 0.8 | 7 | 4.8 | 1.3 | 1.3 | 1.3 | 2 | 1.3 | 1.4 | 1.5 | 1.5 | 10 | 7.5 | 1.7 | 1.3 | 1.5 | 1.408 |
| Q14651 | Plastin-1 | 78003.4 | 5 | 11.9 | 1.9 | 2.8 | 0.9 | 2 | 5.5 | 0.7 | 0.6 | 1.4 | * | * | * | * | * | 3 | 7.4 | 2.1 | 0.8 | 1.4 | 1.408 |
| P05023 | Sodium/potassium-transporting ATPase subunit alpha-1 | 122278.9 | 9 | 10.9 | 1.2 | 1.8 | 1.3 | 7 | 9.1 | 1.0 | 0.8 | 0.9 | 13 | 15.6 | 1.4 | 2.3 | 1.2 | 12 | 12.5 | 2.0 | 1.7 | 1.5 | 1.406 |
| P18085 | ADP-ribosylation factor 4gi\|68565025\|sp\|Q5RCF1.3\|ARF4_PONAB RecName | 22210.2 | 4 | 34.4 | 1.1 | 1.2 | 0.9 | 3 | 22.7 | 1.6 | 0.9 | 1.4 | * | * | * | * | * | 3 | 28.8 | 2.8 | 1.4 | 1.8 | 1.404 |
| P25787 | Proteasome subunit alpha type-2 | 28030.4 | 4 | 30.3 | 1.3 | 1.7 | 1.0 | 4 | 30.3 | 2.1 | 0.7 | 1.0 | 6 | 36.3 | 1.9 | 2.3 | 1.0 | 5 | 36.7 | 1.1 | 1.4 | 0.9 | 1.404 |
| P15954 | Cytochrome c oxidase subunit 7C, mitochondrial | 7879 | 2 | 28.5 | 0.6 | 1.0 | 1.6 | 2 | 28.5 | 1.2 | 2.3 | 2.1 | 2 | 28.5 | 1.0 | 1.7 | 1.1 | * | * | * | * | * | 1.403 |
| P26038 | Moesin | 76870.5 | 12 | 24 | 1.3 | 1.6 | 0.9 | 12 | 20.9 | 1.4 | 1.2 | 1.2 | * | * | * | * | * | 19 | 35.1 | 1.9 | 1.7 | 1.2 | 1.401 |
| Q07065 | Cytoskeleton-associated protein 4 | 71901.5 | 8 | 18.7 | 1.4 | 1.6 | 1.3 | 5 | 11.9 | 1.4 | 0.9 | 0.8 | 5 | 12.6 | 1.5 | 1.9 | 1.2 | 10 | 23.7 | 1.4 | 1.8 | 1.3 | 1.391 |
| P56470 | Galectin-4 | 37928.6 | 7 | 23.5 | 0.8 | 1.7 | 1.3 | 4 | 13 | 0.8 | 0.7 | 1.0 | 6 | 21.6 | 2.1 | 2.9 | 1.5 | 6 | 21.6 | 1.7 | 0.8 | 1.0 | 1.390 |
| P62820 | Ras-related protein Rab-1A | 25356.2 | 3 | 21.4 | 0.7 | 1.4 | 0.8 | 3 | 19 | 1.3 | 1.0 | 1.3 | * | * | * | * | * | 3 | 21.4 | 3.2 | 1.4 | 1.1 | 1.388 |
| P06703 | Protein S100-A6 | 11534 | 6 | 64.4 | 1.8 | 2.1 | 0.6 | 5 | 61.1 | 0.6 | 1.0 | 0.7 | 7 | 75.5 | 1.4 | 3.6 | 1.4 | 6 | 64.4 | 0.7 | 1.4 | 1.2 | 1.380 |
| P08727 | Keratin, type I cytoskeletal 19 | 46123.4 | 24 | 70.5 | 1.0 | 1.9 | 1.4 | 9 | 28.7 | 0.8 | 0.7 | 1.5 | * | * | * | * | * | 20 | 54.5 | 2.3 | 1.5 | 1.6 | 1.377 |
| P08758 | Annexin A5 | 39164.6 | 12 | 45.9 | 1.5 | 1.3 | 0.6 | 14 | 56.5 | 0.9 | 1.4 | 1.0 | 11 | 43.7 | 1.8 | 1.8 | 1.8 | 14 | 41.2 | 1.1 | 1.9 | 1.3 | 1.376 |
| P04040 | Catalase | 64307.9 | 4 | 12.5 | 1.3 | 1.5 | 1.4 | 6 | 19.1 | 1.2 | 0.8 | 1.5 | 3 | 10.2 | 1.4 | 2.0 | 1.3 | 5 | 13.8 | 1.3 | 1.4 | 1.5 | 1.376 |
| Q01518 | Adenylyl cyclase-associated protein 1 | 58699.7 | 7 | 21 | 1.6 | 2.1 | 0.7 | 5 | 16.8 | 1.3 | 1.2 | 0.9 | 5 | 14.5 | 1.2 | 1.5 | 1.5 | 10 | 29.8 | 0.9 | 2.1 | 1.5 | 1.369 |
| P25705 | ATP synthase subunit alpha, mitochondrial | 64188.3 | 17 | 39.2 | 1.1 | 1.6 | 0.8 | 12 | 31.1 | 1.2 | 0.9 | 1.1 | 21 | 43.7 | 2.0 | 2.3 | 1.3 | 17 | 38.3 | 1.2 | 1.5 | 1.2 | 1.366 |
| P68371 | Tubulin beta-4B chain | 52448.9 | 17 | 45.8 | 1.0 | 1.8 | 0.8 | 17 | 45.8 | 1.2 | 1.0 | 1.0 | 17 | 46.5 | 1.7 | 2.4 | 1.5 | 16 | 45.6 | 1.0 | 1.5 | 1.2 | 1.364 |
| P01857 | Ig gamma-1 chain C region | 40655 | 9 | 24.5 | 2.2 | 0.9 | 0.7 | 10 | 31.2 | 2.9 | 0.9 | 1.1 | 8 | 24.5 | 1.2 | 1.5 | 1.3 | 9 | 24.5 | 1.0 | 1.3 | 1.3 | 1.362 |
| P52209 | 6-phosphogluconate dehydrogenase, decarboxylating | 58697.9 | 5 | 15.1 | 1.0 | 2.3 | 0.9 | * | * | * | * | * | 2 | 8.9 | 1.0 | 1.9 | 1.5 | 2 | 7.6 | 1.2 | 1.0 | 1.7 | 1.361 |
| O43491 | Band 4.1-like protein 2 | 125929.2 | 2 | 2.5 | 0.6 | 1.1 | 1.0 | 2 | 1.6 | 0.8 | 0.8 | 0.9 | 3 | 3 | 1.8 | 2.4 | 1.4 | 4 | 4.2 | 1.7 | 2.4 | 1.8 | 1.360 |
| A6NIZ1 | Ras-related protein Rap-1b-like protein | 23026.7 | 4 | 21.1 | 1.3 | 1.5 | 1.0 | 2 | 14.6 | 1.5 | 0.9 | 1.6 | 2 | 14.6 | 1.5 | 1.9 | 1.7 | 2 | 14.6 | 1.0 | 1.1 | 1.1 | 1.359 |
| P04083 | Annexin A1 | 43554.7 | 8 | 34.6 | 1.7 | 1.4 | 0.5 | 9 | 34.1 | 1.4 | 1.4 | 0.9 | 7 | 32 | 1.1 | 2.0 | 1.8 | 8 | 31.2 | 1.0 | 1.9 | 1.1 | 1.358 |
| P36957 | Dihydrolipoyllysine-residue succinyltransferase component of 2-oxoglutarate dehydrogenase complex, mitochondrial | 52988.9 | 4 | 11.4 | 1.2 | 1.8 | 1.1 | 3 | 9.4 | 1.4 | 1.1 | 1.5 | 5 | 14.3 | 1.1 | 1.8 | 1.2 | 3 | 7.9 | 0.9 | 1.7 | 0.5 | 1.343 |
| P02766 | Transthyretin | 17154.1 | 5 | 49.6 | 2.0 | 1.3 | 1.4 | 5 | 44.8 | 1.0 | 0.7 | 1.3 | 3 | 25.1 | 1.7 | 1.4 | 1.0 | 2 | 31.2 | 1.5 | 1.3 | 1.8 | 1.329 |
| P02765 | Alpha-2-HS-glycoprotein | 42429.2 | 2 | 8.1 | 1.5 | 1.8 | 0.7 | 2 | 8.1 | 1.4 | 1.1 | 1.9 | * | * | * | * | * | 2 | 4.3 | 1.3 | 0.9 | 0.8 | 1.327 |
| P01834 | Ig kappa chain C region | 12933 | 4 | 52.8 | 1.9 | 1.0 | 0.8 | 3 | 48.1 | 1.9 | 0.8 | 1.0 | 4 | 52.8 | 1.3 | 1.8 | 1.5 | 4 | 52.8 | 1.1 | 1.3 | 1.1 | 1.324 |
| P10606 | Cytochrome c oxidase subunit 5B, mitochondrial | 14788.6 | 4 | 24.8 | 0.8 | 1.3 | 0.8 | 2 | 15.5 | 1.1 | 0.6 | 1.2 | 4 | 25.5 | 1.6 | 2.4 | 1.8 | 5 | 25.5 | 1.4 | 1.5 | 1.2 | 1.322 |
| P26447 | Protein S100-A4 | 13686.4 | 3 | 26.7 | 1.5 | 1.3 | 0.5 | 3 | 26.7 | 0.6 | 0.8 | 1.1 | 3 | 27.7 | 1.6 | 2.1 | 1.8 | 3 | 26.7 | 1.5 | 1.7 | 1.2 | 1.320 |
| P49411 | Elongation factor Tu, mitochondrial | 54207.6 | 6 | 18.8 | 0.5 | 1.5 | 0.5 | 6 | 17.6 | 1.3 | 1.0 | 1.5 | 12 | 35.6 | 1.7 | 2.2 | 1.5 | 6 | 17.4 | 1.2 | 1.3 | 1.1 | 1.314 |
| P05026 | Sodium/potassium-transporting ATPase subunit beta-1 | 39928.8 | 2 | 8.2 | 1.0 | 1.3 | 1.3 | 2 | 8.2 | 1.0 | 0.4 | 1.1 | 3 | 16.5 | 2.3 | 2.4 | 0.9 | 3 | 16.5 | 1.4 | 1.3 | 1.2 | 1.313 |
| P0C0L4 | Complement C4-A | 205465.3 | 14 | 14.6 | 1.4 | 1.3 | 0.7 | 12 | 12.1 | 1.8 | 1.1 | 1.5 | 8 | 7.2 | 1.8 | 1.8 | 1.4 | 12 | 13.2 | 1.0 | 0.8 | 1.1 | 1.312 |
| P31930 | Cytochrome b-c1 complex subunit 1, mitochondrial | 55491.8 | 4 | 14.7 | 1.0 | 2.1 | 1.1 | 2 | 10.2 | 1.2 | 0.7 | 0.6 | 5 | 16.8 | 1.6 | 2.5 | 1.4 | 2 | 6.6 | 0.9 | 1.3 | 1.1 | 1.305 |
| P07954 | Fumarate hydratase, mitochondrial | 59420.1 | 4 | 13.7 | 0.8 | 1.5 | 0.7 | 4 | 14.5 | 1.3 | 1.3 | 0.9 | 6 | 19.6 | 1.1 | 2.4 | 1.2 | 7 | 21.5 | 1.7 | 1.3 | 1.3 | 1.302 |
| Q15019 | Septin-2 | 45463 | 3 | 14.9 | 1.2 | 1.5 | 0.8 | 2 | 9.9 | 1.1 | 1.0 | 0.6 | 2 | 11.9 | 1.5 | 2.5 | 1.2 | 6 | 28.8 | 1.4 | 1.5 | 1.3 | 1.298 |
| P05556 | Integrin beta-1 | 99939.7 | 3 | 5.5 | 0.7 | 1.0 | 0.6 | * | * | * | * | * | 3 | 6.5 | 2.8 | 2.1 | 1.6 | 4 | 7.6 | 0.8 | 0.9 | 0.7 | 1.284 |
| Q9BQE3 | Tubulin alpha-1C chain | 53318 | 11 | 32.5 | 0.9 | 1.7 | 0.6 | 14 | 41.6 | 1.2 | 1.1 | 0.8 | 14 | 39.1 | 1.6 | 2.4 | 1.4 | 16 | 42.7 | 1.0 | 1.4 | 1.0 | 1.283 |
| P48735 | Isocitrate dehydrogenase [NADP], mitochondrial | 56554.3 | 9 | 23.2 | 0.8 | 1.7 | 1.0 | 4 | 11.9 | 1.2 | 0.7 | 1.4 | * | * | * | * | * | 10 | 25.2 | 1.5 | 1.9 | 1.3 | 1.277 |
| O75083 | WD repeat-containing protein 1 | 72643.1 | 7 | 20.4 | 1.1 | 2.2 | 0.9 | 3 | 8.5 | 1.3 | 0.8 | 1.4 | 8 | 19.3 | 1.3 | 2.0 | 1.4 | 6 | 16.3 | 0.7 | 0.9 | 0.7 | 1.272 |
| P36873 | Serine/threonine-protein phosphatase PP1-gamma catalytic subunit | 41040.4 | 2 | 8.6 | 0.9 | 1.0 | 0.6 | * | * | * | * | * | 2 | 9.5 | 1.8 | 1.2 | 1.8 | 3 | 13 | 1.7 | 1.1 | 1.3 | 1.270 |
| P23528 | Cofilin-1 | 22334.2 | 10 | 54.2 | 1.1 | 1.7 | 0.7 | 10 | 57.2 | 1.4 | 1.0 | 0.7 | 8 | 47.5 | 2.0 | 2.5 | 1.1 | 7 | 53.6 | 0.7 | 0.9 | 0.7 | 1.264 |
| Q16795 | NADH dehydrogenase [ubiquinone] 1 alpha subcomplex subunit 9, mitochondrial | 45851.3 | 3 | 13.5 | 1.2 | 1.6 | 0.7 | 2 | 8.2 | 1.5 | 0.8 | 0.5 | 2 | 8.2 | 1.3 | 2.0 | 1.6 | 2 | 10.3 | 1.0 | 1.7 | 2.1 | 1.259 |
| P00450 | Ceruloplasmin | 132572.9 | 5 | 8 | 1.4 | 1.2 | 0.7 | 3 | 4.7 | 1.8 | 1.2 | 0.8 | 4 | 5.5 | 1.5 | 1.4 | 1.7 | 3 | 4.7 | 0.9 | 1.2 | 1.1 | 1.257 |
| P07148 | Fatty acid-binding protein, liver | 16427.5 | 4 | 44.8 | 0.4 | 1.1 | 0.8 | 4 | 33.8 | 0.8 | 0.4 | 2.2 | 6 | 56.6 | 1.2 | 3.7 | 1.7 | 5 | 38.5 | 0.7 | 0.9 | 1.2 | 1.252 |
| O95865 | N(G),N(G)-dimethylarginine dimethylaminohydrolase 2 | 30937.7 | 2 | 8.4 | 1.5 | 0.9 | 1.0 | * | * | * | * | * | 2 | 8.4 | 1.0 | 1.3 | 1.1 | 2 | 13.6 | 2.1 | 1.2 | 1.8 | 1.250 |
| P0CG05 | Ig lambda-2 chain C regions | 12617.8 | 4 | 48.1 | 1.7 | 0.9 | 0.8 | 4 | 48.1 | 2.0 | 0.8 | 0.9 | 4 | 48.1 | 1.1 | 1.7 | 1.2 | * | * | * | * | * | 1.248 |
| Q9Y6N5 | Sulfide | 55146.3 | 7 | 22 | 0.8 | 1.7 | 1.4 | 4 | 14.8 | 1.8 | 0.4 | 0.6 | 7 | 27.1 | 2.0 | 1.8 | 1.1 | 7 | 25.7 | 1.1 | 1.0 | 0.9 | 1.247 |
| P54868 | Hydroxymethylglutaryl-CoA synthase, mitochondrial | 61328.4 | 3 | 10.2 | 0.7 | 1.4 | 1.9 | 3 | 8 | 0.9 | 0.8 | 1.0 | * | * | * | * | * | 2 | 6.1 | 1.9 | 1.3 | 1.1 | 1.233 |
| P05091 | Aldehyde dehydrogenase, mitochondrial | 61074.2 | 8 | 19.5 | 0.9 | 1.3 | 0.6 | 5 | 12.7 | 1.4 | 0.8 | 0.5 | 12 | 24.5 | 1.8 | 2.5 | 1.4 | * | * | * | * | * | 1.231 |
| Q99798 | Aconitate hydratase, mitochondrial | 94094 | 4 | 9.2 | 1.1 | 1.1 | 0.5 | * | * | * | * | * | 6 | 14.1 | 1.8 | 2.0 | 1.4 | 4 | 7.5 | 0.8 | 1.2 | 0.8 | 1.224 |
| P30086 | Phosphatidylethanolamine-binding protein 1 | 23332.8 | 6 | 54.5 | 1.0 | 1.3 | 1.0 | 7 | 58.8 | 0.9 | 0.8 | 1.0 | 6 | 51.3 | 1.5 | 1.7 | 1.3 | 7 | 60.4 | 1.3 | 1.5 | 1.0 | 1.204 |
| P20671 | Histone H2A type 1-D | 16125.4 | 4 | 49.2 | 1.7 | 1.3 | 0.8 | 4 | 52.3 | 2.2 | 1.0 | 0.5 | * | * | * | * | * | 4 | 49.2 | 0.7 | 1.5 | 1.0 | 1.201 |
| P13645 | Keratin, type I cytoskeletal 10 | 62369.8 | 7 | 13.8 | 0.9 | 1.1 | 1.0 | 10 | 21.2 | 1.0 | 0.7 | 1.4 | 7 | 12.1 | 1.5 | 1.8 | 1.1 | 8 | 18.3 | 1.3 | 1.3 | 1.2 | 1.198 |
| P16402 | Histone H1.3 | 31142.9 | 6 | 15.3 | 0.8 | 1.3 | 0.8 | 3 | 10.8 | 1.0 | 0.9 | 0.8 | 8 | 22.6 | 2.0 | 1.7 | 1.4 | * | * | * | * | * | 1.196 |
| P06576 | ATP synthase subunit beta, mitochondrial | 59874.4 | 19 | 50.4 | 0.9 | 1.8 | 0.5 | 14 | 37.2 | 1.1 | 0.7 | 0.7 | 18 | 40.8 | 1.8 | 2.5 | 1.2 | 15 | 37.8 | 0.7 | 1.1 | 0.9 | 1.196 |
| P02675 | Fibrinogen beta chain | 61801.4 | 9 | 26 | 1.3 | 1.4 | 1.0 | 10 | 27.2 | 1.2 | 0.7 | 1.3 | 6 | 16.4 | 1.3 | 1.7 | 1.3 | 8 | 20.5 | 0.9 | 0.9 | 1.2 | 1.193 |
| P04899 | Guanine nucleotide-binding protein G(i) subunit alpha-2 | 44912.9 | * | * | * | * | * | 2 | 7.6 | 1.1 | 0.7 | 0.6 | 2 | 7.8 | 1.2 | 2.5 | 1.2 | 3 | 10.1 | 1.2 | 1.2 | 1.1 | 1.192 |
| P01024 | Complement C3 | 204974.5 | 30 | 26.9 | 1.3 | 1.4 | 0.7 | 24 | 22.3 | 1.3 | 1.0 | 1.3 | 15 | 14.4 | 1.4 | 1.6 | 1.2 | 21 | 18.8 | 1.0 | 0.9 | 1.1 | 1.190 |
| P00403 | Cytochrome c oxidase subunit 2 | 26312.3 | 2 | 13.2 | 0.8 | 1.5 | 0.6 | 2 | 13.2 | 0.8 | 0.7 | 0.4 | 2 | 13.2 | 1.7 | 2.7 | 1.3 | 2 | 13.2 | 0.8 | 1.7 | 0.9 | 1.188 |
| P04217 | Alpha-1B-glycoprotein | 56408.7 | 3 | 11.5 | 1.4 | 1.2 | 1.1 | 4 | 13.9 | 1.6 | 0.6 | 1.1 | 3 | 11.5 | 1.2 | 1.2 | 1.2 | 3 | 11.5 | 1.6 | 0.9 | 1.1 | 1.188 |
| P30041 | Peroxiredoxin-6 | 27743.4 | 7 | 40.6 | 1.1 | 1.4 | 0.5 | 4 | 20.5 | 0.8 | 0.8 | 1.1 | 6 | 33 | 1.9 | 2.0 | 1.3 | 8 | 41 | 0.9 | 1.0 | 0.9 | 1.164 |
| Q15056 | Eukaryotic translation initiation factor 4H | 29027.3 | 2 | 15.3 | 0.9 | 0.9 | 1.1 | * | * | * | * | * | 2 | 15.3 | 1.1 | 1.7 | 0.7 | 2 | 15.3 | 1.2 | 1.7 | 1.9 | 1.156 |
| P01871 | Ig mu chain C region | 53161.7 | 5 | 16.5 | 1.0 | 1.5 | 1.5 | 7 | 20.1 | 1.6 | 1.0 | 0.8 | 4 | 12.3 | 1.0 | 1.2 | 0.9 | 5 | 16.5 | 1.0 | 1.0 | 1.3 | 1.155 |
| P22695 | Cytochrome b-c1 complex subunit 2, mitochondrial | 52073 | 3 | 12.1 | 0.7 | 1.4 | 0.6 | 2 | 8.6 | 0.9 | 0.5 | 0.9 | 5 | 18.3 | 1.5 | 2.1 | 1.6 | 7 | 25.1 | 1.2 | 1.3 | 1.1 | 1.150 |
| O95994 | Anterior gradient protein 2 homolog | 22630.7 | 10 | 38.8 | 0.8 | 1.8 | 1.4 | 5 | 32.5 | 0.8 | 0.3 | 0.9 | 8 | 45.7 | 1.3 | 2.4 | 0.9 | 7 | 38.2 | 1.2 | 0.7 | 0.9 | 1.149 |
| P01023 | Alpha-2-macroglobulin | 177544 | 13 | 13.2 | 1.0 | 1.1 | 0.7 | 11 | 10.4 | 1.8 | 1.0 | 1.2 | 10 | 10.3 | 1.3 | 1.5 | 1.3 | 11 | 12.4 | 1.0 | 0.7 | 0.8 | 1.146 |
| P16152 | Carbonyl reductase [NADPH] 1 | 33254.4 | 4 | 22.3 | 1.0 | 1.2 | 0.9 | 4 | 26.7 | 1.4 | 0.6 | 0.7 | 2 | 11.1 | 1.5 | 1.4 | 1.1 | 4 | 22.3 | 1.2 | 1.5 | 1.0 | 1.135 |
| P02652 | Apolipoprotein A-II | 12730.5 | 4 | 43 | 1.1 | 1.1 | 0.9 | 2 | 11 | 1.6 | 1.1 | 1.1 | 4 | 44 | 1.0 | 1.5 | 1.0 | 2 | 21 | 1.2 | 1.0 | 1.0 | 1.131 |
| P07737 | Profilin-1 | 16666.7 | 9 | 57.1 | 1.0 | 1.2 | 0.7 | 8 | 57.1 | 0.9 | 0.8 | 1.2 | 9 | 57.1 | 1.3 | 1.8 | 1.2 | 9 | 57.1 | 1.2 | 1.2 | 1.0 | 1.130 |
| P02671 | Fibrinogen alpha chain | 101911.7 | 11 | 13.5 | 1.3 | 1.2 | 0.8 | 11 | 15.4 | 1.2 | 0.6 | 1.1 | 8 | 10.5 | 1.2 | 1.8 | 1.0 | 13 | 17.3 | 1.2 | 0.9 | 1.0 | 1.127 |
| P01876 | Ig alpha-1 chain C region | 40383.8 | 6 | 18.9 | 1.5 | 1.0 | 0.9 | 7 | 20.3 | 1.6 | 0.5 | 0.8 | 6 | 18.4 | 1.2 | 2.0 | 1.0 | 6 | 18.9 | 0.9 | 0.8 | 1.0 | 1.126 |
| Q07960 | Rho GTPase-activating protein 1 | 54816.5 | 2 | 6.1 | 1.0 | 1.2 | 1.1 | 2 | 5.6 | 0.6 | 0.6 | 0.5 | 4 | 16.6 | 1.2 | 1.4 | 1.4 | 4 | 17.9 | 1.0 | 2.3 | 0.9 | 1.123 |
| P02679 | Fibrinogen gamma chain | 57039.5 | 7 | 23.6 | 1.5 | 1.1 | 0.7 | 7 | 22.7 | 1.3 | 0.7 | 1.1 | 6 | 20.9 | 1.0 | 1.7 | 1.2 | 7 | 23.3 | 1.0 | 0.9 | 0.9 | 1.113 |
| P00751 | Complement factor B | 94540.8 | 3 | 5.1 | 1.0 | 1.1 | 0.5 | 2 | 3.1 | 1.1 | 0.6 | 0.8 | 2 | 3.2 | 1.4 | 2.3 | 1.0 | * | * | * | * | * | 1.102 |
| Q14195 | Dihydropyrimidinase-related protein 3 | 67262.8 | 5 | 12.6 | 1.2 | 1.0 | 0.5 | 5 | 15.4 | 0.9 | 0.8 | 0.8 | 2 | 5.9 | 1.2 | 1.4 | 1.9 | 4 | 11.5 | 0.9 | 1.4 | 0.7 | 1.087 |
| P02787 | Serotransferrin | 87705.6 | 17 | 22.6 | 1.2 | 1.2 | 0.8 | 21 | 27.6 | 1.3 | 0.9 | 1.0 | 14 | 19.3 | 1.2 | 1.5 | 1.2 | 17 | 23.2 | 0.7 | 0.9 | 0.9 | 1.079 |
| O95571 | Protein ETHE1, mitochondrial | 29106.7 | 2 | 12.2 | 0.8 | 1.4 | 0.7 | * | * | * | * | * | 2 | 12.2 | 1.0 | 1.9 | 0.7 | 3 | 15.7 | 0.9 | 1.1 | 1.0 | 1.076 |
| P02647 | Apolipoprotein A-I | 33948.6 | 21 | 59.1 | 1.4 | 0.8 | 0.7 | 21 | 65.9 | 1.2 | 0.9 | 1.1 | 17 | 56.5 | 1.4 | 1.2 | 1.2 | 18 | 57.3 | 0.9 | 0.9 | 1.0 | 1.071 |
| P02774 | Vitamin D-binding protein | 60903 | 4 | 13.7 | 1.3 | 1.0 | 0.6 | 6 | 16.6 | 1.4 | 0.7 | 1.3 | 4 | 11.6 | 1.0 | 1.5 | 1.1 | 4 | 13.7 | 0.9 | 1.0 | 1.2 | 1.058 |
| A8K7I4 | Calcium-activated chloride channel regulator 1 | 108605.6 | 9 | 15.6 | 0.8 | 1.3 | 2.6 | * | * | * | * | * | 5 | 8.6 | 0.7 | 1.0 | 0.5 | 5 | 9.4 | 0.8 | 0.7 | 0.9 | 1.050 |
| P00352 | Retinal dehydrogenase 1 | 60966.3 | 2 | 4.5 | 0.8 | 1.0 | 0.5 | 4 | 11.9 | 1.2 | 0.7 | 0.4 | 3 | 7.1 | 1.2 | 2.5 | 1.1 | 8 | 21.1 | 0.9 | 1.1 | 0.8 | 1.038 |
| P09382 | Galectin-1 | 16211 | 5 | 26.6 | 1.2 | 0.8 | 0.6 | 5 | 31.1 | 0.4 | 0.9 | 0.5 | 4 | 19.2 | 1.1 | 1.5 | 1.8 | 5 | 26.6 | 1.0 | 1.3 | 0.9 | 1.005 |
| Q13228 | Selenium-binding protein 1 | 56996.9 | 10 | 29 | 0.5 | 1.5 | 0.7 | 10 | 27.9 | 0.7 | 0.4 | 0.6 | 13 | 34.1 | 1.5 | 2.9 | 0.9 | 7 | 20.3 | 0.6 | 0.5 | 0.9 | 1.005 |
| P00738 | Haptoglobin | 50934.5 | 14 | 24.8 | 1.8 | 0.7 | 0.6 | 12 | 24.3 | 1.6 | 0.7 | 0.8 | 13 | 24.6 | 0.7 | 1.6 | 0.9 | 14 | 26.3 | 0.8 | 0.8 | 0.8 | 0.999 |
| O43707 | Alpha-actinin-4 | 113669.5 | 29 | 41.7 | 0.9 | 0.9 | 0.9 | 22 | 30 | 0.7 | 0.7 | 0.9 | 23 | 32.3 | 1.2 | 1.2 | 0.9 | 31 | 44.8 | 1.2 | 1.2 | 0.8 | 0.986 |
| P30043 | Flavin reductase (NADPH) | 23530.5 | 4 | 28.1 | 1.7 | 1.5 | 0.8 | 2 | 12.1 | 0.8 | 0.7 | 1.0 | 5 | 47.5 | 1.0 | 1.3 | 0.7 | 5 | 36.4 | 0.7 | 0.6 | 0.8 | 0.985 |
| P08133 | Annexin A6 | 83710.3 | 12 | 26 | 0.9 | 0.9 | 0.5 | 9 | 16.1 | 0.9 | 1.0 | 0.9 | 12 | 24.2 | 1.2 | 1.3 | 1.2 | 16 | 32 | 0.7 | 1.0 | 0.7 | 0.958 |
| Q09666 | Neuroblast differentiation-associated protein AHNAK | 748819.1 | 59 | 13.9 | 0.9 | 1.1 | 0.7 | 32 | 6.4 | 0.6 | 0.9 | 0.8 | 29 | 5.8 | 1.0 | 1.2 | 0.9 | 52 | 11.3 | 1.2 | 1.0 | 0.8 | 0.952 |
| P02768 | Serum albumin | 80011.6 | 33 | 47.6 | 1.3 | 0.9 | 0.6 | 32 | 37.4 | 1.3 | 0.8 | 0.8 | 29 | 35.7 | 0.9 | 1.1 | 1.1 | 28 | 35.1 | 0.6 | 0.7 | 0.8 | 0.924 |
| Q9Y490 | Talin-1 | 295283.4 | 24 | 16.6 | 0.8 | 0.9 | 0.5 | 32 | 23 | 0.8 | 0.9 | 0.8 | 25 | 17.6 | 1.3 | 1.4 | 1.1 | 31 | 20.7 | 0.7 | 0.8 | 0.6 | 0.914 |
| P06396 | Gelsolin | 92582.2 | 15 | 29.4 | 0.9 | 0.9 | 0.6 | 10 | 20.7 | 1.0 | 0.7 | 0.8 | 9 | 21.6 | 0.9 | 1.4 | 0.9 | 14 | 23.4 | 0.6 | 1.0 | 0.6 | 0.883 |
| P02790 | Hemopexin | 55444.3 | 4 | 10.8 | 1.4 | 1.1 | 0.6 | 3 | 6.2 | 1.5 | 0.7 | 0.7 | * | * | * | * | * | 3 | 7.3 | 0.5 | 0.5 | 0.8 | 0.865 |
| P18206 | Vinculin | 135611.7 | 32 | 37.6 | 0.7 | 0.9 | 0.5 | 30 | 34.9 | 0.8 | 0.9 | 0.8 | 19 | 22.5 | 0.9 | 1.1 | 1.1 | 31 | 35.8 | 0.7 | 0.8 | 0.7 | 0.843 |
| Q93052 | Lipoma-preferred partner | 71640.3 | 2 | 6.3 | 0.4 | 1.2 | 0.8 | 4 | 10.1 | 0.7 | 0.6 | 1.4 | 4 | 9.1 | 0.7 | 0.9 | 0.6 | 4 | 10.6 | 0.7 | 0.8 | 0.6 | 0.799 |
| Q15661 | Tryptase alpha/beta-1 | 32124.2 | 2 | 9.8 | 0.5 | 0.7 | 0.4 | * | * | * | * | * | 2 | 9.8 | 1.2 | 1.1 | 0.9 | 3 | 14.9 | 0.6 | 0.9 | 0.8 | 0.796 |
| P40925 | Malate dehydrogenase, cytoplasmic | 41122.5 | 2 | 7.4 | 0.7 | 0.9 | 0.4 | 2 | 6.5 | 0.9 | 0.3 | 0.6 | 3 | 11 | 0.9 | 1.3 | 0.9 | 4 | 12.8 | 0.8 | 1.1 | 0.6 | 0.794 |
| Q6NZI2 | Polymerase I and transcript release factor | 49097.4 | 3 | 12 | 0.7 | 0.7 | 0.6 | 4 | 15.6 | 0.5 | 0.6 | 0.8 | 2 | 7.4 | 1.1 | 1.1 | 0.9 | * | * | * | * | * | 0.781 |
| P01009 | Alpha-1-antitrypsin | 51808.1 | 18 | 47.1 | 1.6 | 0.7 | 0.4 | 16 | 37.7 | 1.1 | 0.8 | 0.4 | 13 | 33.7 | 0.8 | 1.0 | 0.7 | 18 | 43 | 0.4 | 0.7 | 0.7 | 0.780 |
| P68871 | Hemoglobin subunit beta | 17697.9 | 10 | 70 | 1.0 | 0.7 | 0.7 | 10 | 70 | 0.5 | 0.5 | 0.3 | 10 | 70 | 1.5 | 1.3 | 0.9 | 10 | 70 | 0.6 | 0.6 | 0.6 | 0.767 |
| P12111 | Collagen alpha-3(VI) chain | 368151 | 10 | 4.8 | 1.0 | 1.1 | 0.4 | 10 | 4.8 | 1.0 | 0.8 | 1.0 | 4 | 2.2 | 0.3 | 0.6 | 0.4 | 10 | 5.1 | 1.0 | 0.8 | 0.6 | 0.766 |
| P20962 | Parathymosin | 13403.7 | 2 | 15.6 | 0.4 | 0.6 | 0.3 | 2 | 15.6 | 0.9 | 0.7 | 1.0 | 2 | 15.6 | 0.9 | 1.1 | 0.9 | * | * | * | * | * | 0.757 |
| P69905 | Hemoglobin subunit alpha | 16900 | 9 | 67.6 | 0.9 | 0.8 | 0.4 | 8 | 64 | 0.6 | 0.5 | 0.3 | 9 | 67.6 | 1.6 | 1.3 | 0.8 | 9 | 64 | 0.3 | 0.4 | 0.5 | 0.725 |
| Q05682 | Caldesmon | 108278.5 | 13 | 20 | 0.8 | 0.7 | 0.3 | 15 | 19.6 | 0.5 | 0.6 | 0.6 | 8 | 15.1 | 0.9 | 1.2 | 0.8 | 16 | 23.7 | 0.5 | 0.5 | 0.4 | 0.678 |
| P00915 | Carbonic anhydrase 1 | 31521.5 | 7 | 36.7 | 0.5 | 0.8 | 0.6 | 4 | 20.3 | 0.5 | 0.5 | 0.4 | 8 | 46.3 | 1.0 | 1.2 | 0.7 | 6 | 26.8 | 0.5 | 0.5 | 0.5 | 0.668 |
| P12277 | Creatine kinase B-type | 45956 | 14 | 48.8 | 0.4 | 1.2 | 0.6 | 13 | 50.9 | 0.7 | 0.4 | 0.5 | 10 | 38.3 | 0.6 | 1.1 | 0.6 | 14 | 51.1 | 0.6 | 0.4 | 0.5 | 0.654 |
| Q9NZN4 | EH domain-containing protein 2 | 66809.3 | 4 | 8.8 | 0.4 | 0.4 | 0.2 | 4 | 8.8 | 0.7 | 1.0 | 0.6 | 4 | 8.8 | 0.6 | 0.9 | 0.7 | 4 | 8.8 | 0.6 | 0.6 | 0.4 | 0.627 |
| Q562R1 | Beta-actin-like protein 2 | 44939.5 | 12 | 20.7 | 0.5 | 0.6 | 0.4 | * | * | * | * | * | 13 | 28.7 | 0.7 | 0.9 | 0.7 | 11 | 19.6 | 0.6 | 0.6 | 0.5 | 0.626 |
| Q8WX93 | Palladin | 161877.1 | 2 | 2.2 | 0.5 | 0.7 | 0.3 | 4 | 4.9 | 0.6 | 0.7 | 0.7 | 2 | 2.2 | 0.7 | 0.6 | 0.8 | 2 | 2.6 | 0.6 | 0.8 | 0.4 | 0.626 |
| P04792 | Heat shock protein beta-1 | 23848.2 | 12 | 64.8 | 0.4 | 0.9 | 0.4 | 10 | 66.8 | 0.5 | 0.6 | 0.6 | 10 | 66.8 | 0.7 | 0.6 | 0.8 | 12 | 64.8 | 0.6 | 0.6 | 0.4 | 0.611 |
| P51884 | Lumican | 42374.5 | 8 | 26.9 | 1.2 | 0.7 | 0.3 | 9 | 33.1 | 0.6 | 0.7 | 0.4 | 4 | 15.3 | 0.5 | 0.7 | 0.4 | 9 | 32.5 | 0.4 | 0.8 | 0.5 | 0.601 |
| Q15746 | Myosin light chain kinase, smooth muscle | 234352.2 | 7 | 3.9 | 0.5 | 0.6 | 0.4 | 7 | 3.5 | 0.4 | 0.5 | 0.6 | 5 | 2.1 | 0.8 | 0.9 | 0.9 | 6 | 3.7 | 0.6 | 0.3 | 0.3 | 0.592 |
| P61160 | Actin-related protein 2 | 48649.1 | 4 | 12.4 | 0.5 | 0.5 | 0.3 | 3 | 8.3 | 0.4 | 0.6 | 0.6 | 4 | 10.4 | 0.5 | 0.6 | 0.8 | 5 | 14.7 | 0.8 | 0.8 | 0.5 | 0.584 |
| P21333 | Filamin-A | 306537.1 | 69 | 35.8 | 0.6 | 0.5 | 0.3 | 74 | 37.5 | 0.5 | 0.7 | 0.6 | 66 | 36.4 | 0.6 | 0.7 | 0.7 | 78 | 39.6 | 0.5 | 0.6 | 0.4 | 0.573 |
| Q13418 | Integrin-linked protein kinase | 55679.8 | 2 | 5.3 | 0.5 | 0.5 | 0.3 | 3 | 8.4 | 0.4 | 0.5 | 0.3 | 3 | 6.8 | 0.8 | 0.7 | 0.7 | 3 | 7.3 | 0.5 | 0.7 | 0.6 | 0.550 |
| P00918 | Carbonic anhydrase 2 | 32762.2 | 4 | 21.9 | 0.5 | 0.7 | 0.5 | 3 | 16.9 | 0.3 | 0.3 | 0.4 | 3 | 16.9 | 0.8 | 1.1 | 0.5 | 5 | 26.5 | 0.5 | 0.5 | 0.4 | 0.536 |
| P07585 | Decorin | 43980.5 | 5 | 15.5 | 0.8 | 0.5 | 0.3 | 4 | 12.8 | 0.3 | 0.5 | 0.3 | 4 | 14.4 | 0.5 | 0.4 | 0.4 | 3 | 10 | 0.7 | 1.0 | 0.7 | 0.520 |
| P35749 | Myosin-11 | 257598.5 | 85 | 44.5 | 0.3 | 0.5 | 0.3 | 98 | 48.8 | 0.4 | 0.5 | 0.5 | 70 | 38.5 | 0.6 | 0.8 | 0.6 | 80 | 41 | 0.5 | 0.5 | 0.4 | 0.499 |
| P51888 | Prolargin | 46226.6 | 5 | 13 | 1.2 | 0.7 | 0.3 | 4 | 13.6 | 0.3 | 0.7 | 0.5 | 4 | 11.2 | 0.3 | 0.3 | 0.4 | 5 | 14.6 | 0.4 | 0.4 | 0.4 | 0.497 |
| Q16853 | Membrane primary amine oxidase | 87209 | 2 | 2.4 | 0.4 | 0.4 | 0.2 | 5 | 8.7 | 0.5 | 0.5 | 0.7 | 2 | 3.8 | 0.5 | 0.6 | 0.6 | 6 | 11.1 | 0.5 | 0.4 | 0.3 | 0.489 |
| P21291 | Cysteine and glycine-rich protein 1 | 24738.4 | 3 | 27.9 | 0.4 | 0.4 | 0.2 | 3 | 27.9 | 0.3 | 0.6 | 0.4 | 3 | 27.9 | 0.4 | 0.6 | 0.7 | 2 | 19.1 | 0.4 | 0.3 | 0.3 | 0.433 |
| P24844 | Myosin regulatory light polypeptide 9 | 21902.1 | 6 | 43 | 0.4 | 0.5 | 0.3 | 6 | 37.7 | 0.3 | 0.6 | 0.3 | * | * | * | * | * | 2 | 16.2 | 0.5 | 0.5 | 0.9 | 0.425 |
| Q15124 | Phosphoglucomutase-like protein 5 | 65966 | 2 | 5.2 | 0.3 | 0.2 | 0.3 | 4 | 8.4 | 0.3 | 0.8 | 0.8 | * | * | * | * | * | 3 | 6.8 | 0.4 | 0.4 | 0.2 | 0.425 |
| O14558 | Heat shock protein beta-6 | 17624.8 | 3 | 38.7 | 0.3 | 0.1 | 0.3 | 3 | 46.2 | 0.6 | 0.5 | 0.6 | * | * | * | * | * | 5 | 61.8 | 0.5 | 0.4 | 0.3 | 0.416 |
| Q03135 | Caveolin-1gi\|90111837\|sp\|Q2QLF2.1\|CAV1_PANTR RecName | 22372.2 | 2 | 12.3 | 0.5 | 0.5 | 0.2 | * | * | * | * | * | 3 | 16.8 | 0.6 | 0.4 | 0.3 | 3 | 17.9 | 0.3 | 0.2 | 0.3 | 0.368 |
| P07951 | Tropomyosin beta chain | 38586.3 | 27 | 53.5 | 0.4 | 0.4 | 0.2 | 29 | 60.9 | 0.3 | 0.5 | 0.3 | * | * | * | * | * | 26 | 60.2 | 0.4 | 0.4 | 0.3 | 0.349 |
| Q01995 | Transgelin | 25118.2 | 16 | 67.6 | 0.4 | 0.3 | 0.1 | 17 | 72.6 | 0.3 | 0.6 | 0.3 | 15 | 72.1 | 0.3 | 0.4 | 0.5 | 16 | 72.1 | 0.2 | 0.3 | 0.3 | 0.342 |
| P51911 | Calponin-1 | 36656.6 | 12 | 41.4 | 0.3 | 0.3 | 0.1 | 12 | 42.7 | 0.3 | 0.5 | 0.4 | 10 | 37.7 | 0.3 | 0.5 | 0.5 | 13 | 43.7 | 0.2 | 0.2 | 0.2 | 0.324 |
