# Supplementary material for: Quantitative mass spectrometry analysis reveals a panel of nine proteins as diagnostic markers for colon adenocarcinomas
Source: Oncotarget. 2018 Feb 5;9(17):13530–44. doi: 10.18632/oncotarget.24418 (PMC5862596; doi:10.18632/oncotarget.24418)
Supplement: Supplementary file 3 [file oncotarget-09-13530-s003.docx]

| **Supplementary Table 1B: List of differentially expressed proteins identified from iTRAQ analysis using Trans Proteome Pipeline (TPP)** | | | | | | | | | | | | | | | | | | | | | | |
| --- | --- | --- | --- | --- | --- | --- | --- | --- | --- | --- | --- | --- | --- | --- | --- | --- | --- | --- | --- | --- | --- | --- |
| accession_number | entry_name | Set 1_LIBRA number peptides | Set 1_percent coverage | Set 1_Fold Change_115/114 | Set 1_Fold Change_116/114 | Set 1_Fold Change_117/114 | Set 2_LIBRA number peptides | Set 2_percent coverage | Set 2_Fold Change_115/114 | Set 2_Fold Change_116/114 | Set 2_Fold Change_117/114 | Set 3_LIBRA number peptides | Set 3_percent coverage | Set 3_Fold Change_115/114 | Set 3_Fold Change_116/114 | Set 3_Fold Change_117/114 | Set 4_LIBRA number peptides | Set 4_percent coverage | Set 4_Fold Change_115/114 | Set 4_Fold Change_116/114 | Set 4_Fold Change_117/114 (Pool) | Average Fold change (n=11) |
| P23368 | NAD-dependent malic enzyme, mitochondrial OS=Homo sapiens GN=ME2 PE=1 SV=1 | 2 | 11.6 | 8.0 | 10.7 | 13.3 | * | * | * | * | * | 4 | 3.1 | 0.8 | 2.2 | 0.6 | 13 | 10.1 | 1.9 | 1.5 | 1.3 | 4.868 |
| P80723 | Brain acid soluble protein 1 OS=Homo sapiens GN=BASP1 PE=1 SV=2 | * | * | * | * | * | 7 | 38.3 | 1.3 | 1.0 | 1.5 | 2 | 15.4 | 8.6 | 5.2 | 5.0 | 8 | 38.3 | 2.8 | 2.6 | 2.2 | 3.499 |
| P37108 | Signal recognition particle 14 kDa protein OS=Homo sapiens GN=SRP14 PE=1 SV=2 | 7 | 17.6 | 0.8 | 1.5 | 0.8 | 2 | 10.3 | 9.8 | 1.5 | 12.5 | 10 | 34.6 | 2.8 | 4.1 | 0.8 | 9 | 17.6 | 1.7 | 1.3 | 1.5 | 3.403 |
| O15355 | Protein phosphatase 1G OS=Homo sapiens GN=PPM1G PE=1 SV=1 | 3 | 2.6 | 3.1 | 7.9 | 1.4 | * | * | * | * | * | 5 | 4 | 1.4 | 3.2 | 1.1 | 3 | 2.6 | 4.5 | 3.5 | 3.3 | 3.277 |
| P06702 | Protein S100-A9 OS=Homo sapiens GN=S100A9 PE=1 SV=1 | 112 | 81.6 | 2.3 | 3.5 | 1.1 | 232 | 81.6 | 5.6 | 1.1 | 3.8 | 148 | 81.6 | 3.3 | 3.4 | 4.0 | 217 | 82.5 | 5.5 | 2.0 | 2.8 | 3.223 |
| P05109 | Protein S100-A8 OS=Homo sapiens GN=S100A8 PE=1 SV=1 | 45 | 48.4 | 2.3 | 3.5 | 1.2 | 101 | 50.5 | 4.2 | 1.0 | 4.1 | 53 | 58.1 | 3.1 | 2.9 | 3.6 | 90 | 65.6 | 8.0 | 1.6 | 2.7 | 3.216 |
| Q93009 | Ubiquitin carboxyl-terminal hydrolase 7 OS=Homo sapiens GN=USP7 PE=1 SV=2 | 2 | 1.3 | 4.2 | 9.0 | 2.3 | * | * | * | * | * | 12 | 3.5 | 1.9 | 2.0 | 1.5 | 8 | 3.5 | 2.9 | 1.6 | 1.8 | 3.183 |
| P62244 | 40S ribosomal protein S15a OS=Homo sapiens GN=RPS15A PE=1 SV=2 | 7 | 27.7 | 1.8 | 3.4 | 2.0 | * | * | * | * | * | 11 | 25.4 | 4.0 | 5.4 | 3.0 | 9 | 35.4 | 2.2 | 2.6 | 1.6 | 3.046 |
| P02792 | Ferritin light chain OS=Homo sapiens GN=FTL PE=1 SV=2 | 17 | 36.0 | 3.4 | 1.3 | 0.7 | 8 | 17.7 | 2.5 | 3.0 | 3.0 | 2 | 17.7 | 3.4 | 3.0 | 6.4 | 21 | 40 | 1.5 | 4.9 | 1.9 | 3.017 |
| P08195 | 4F2 cell-surface antigen heavy chain OS=Homo sapiens GN=SLC3A2 PE=1 SV=3 | 3 | 4.0 | 2.6 | 4.4 | 2.3 | 2 | 7.0 | 7.5 | 1.8 | 5.3 | 6 | 12.4 | 1.5 | 1.5 | 1.1 | 9 | 12.4 | 2.5 | 2.5 | 1.1 | 3.009 |
| P63313 | Thymosin beta-10 OS=Homo sapiens GN=TMSB10 PE=1 SV=2 | 7 | 29.5 | 2.8 | 4.4 | 2.6 | * | * | * | * | * | 8 | 29.5 | 2.6 | 4.3 | 1.5 | 8 | 50 | 2.7 | 2.6 | 2.5 | 2.943 |
| Q92882 | Osteoclast-stimulating factor 1 OS=Homo sapiens GN=OSTF1 PE=1 SV=2 | * | * | * | * | * | 3 | 5.6 | 0.9 | 0.3 | 1.1 | 11 | 5.6 | 1.4 | 3.2 | 1.2 | 5 | 5.6 | 8.0 | 7.3 | 7.3 | 2.913 |
| P34897 | Serine hydroxymethyltransferase, mitochondrial OS=Homo sapiens GN=SHMT2 PE=1 SV=3 | 8 | 8.9 | 1.1 | 3.5 | 1.1 | * | * | * | * | * | 6 | 11.1 | 6.2 | 5.8 | 2.8 | 11 | 12.9 | 0.7 | 1.8 | 1.0 | 2.887 |
| P68431 | Histone H3.1 OS=Homo sapiens GN=HIST1H3A PE=1 SV=2;Histone H3.3 OS=Homo sapiens GN=H3F3A PE=1 SV=2;Histone H3.1t OS=Homo sapiens GN=HIST3H3 PE=1 SV=3;Histone H3.2 OS=Homo sapiens GN=HIST2H3A PE=1 SV=3 | * | * | * | * | * | 31 | 17.6 | 5.4 | 1.8 | 3.4 | 7 | 11.8 | 4.2 | 1.7 | 2.8 | 7 | 10.3 | 1.9 | 2.0 | 1.6 | 2.883 |
| P61604 | 10 kDa heat shock protein, mitochondrial OS=Homo sapiens GN=HSPE1 PE=1 SV=2 | 153 | 77.5 | 1.3 | 4.5 | 1.7 | 115 | 77.5 | 2.3 | 1.1 | 2.8 | 155 | 81.4 | 5.3 | 4.1 | 2.7 | 107 | 70.6 | 3.0 | 2.8 | 2.6 | 2.877 |
| Q15046 | Lysine--tRNA ligase OS=Homo sapiens GN=KARS PE=1 SV=3 | 6 | 6.2 | 0.6 | 2.3 | 1.1 | * | * | * | * | * | 6 | 6.7 | 2.3 | 3.7 | 2.0 | 4 | 3.5 | 7.7 | 3.2 | 4.3 | 2.848 |
| P61626 | sp LYSC_HUMAN ;Lysozyme C OS=Homo sapiens GN=LYZ PE=1 SV=1 | 19 | 18.2 | 1.4 | 2.1 | 3.9 | 16 | 24.3 | 3.2 | 1.0 | 2.8 | 9 | 13.5 | 2.3 | 5.3 | 2.9 | 28 | 24.3 | 4.5 | 1.2 | 1.9 | 2.761 |
| O60749 | Sorting nexin-2 OS=Homo sapiens GN=SNX2 PE=1 SV=2 | 3 | 6.9 | 1.6 | 2.3 | 1.9 | * | * | * | * | * | 2 | 4.8 | 3.0 | 6.3 | 2.9 | 6 | 6.9 | 1.9 | 1.7 | 1.6 | 2.699 |
| Q56VL3 | OCIA domain-containing protein 2 OS=Homo sapiens GN=OCIAD2 PE=1 SV=1 | 9 | 31.2 | 2.7 | 7.0 | 2.9 | 5 | 8.4 | 0.7 | 1.4 | 1.5 | 11 | 8.4 | 2.0 | 4.6 | 2.6 | 6 | 14.3 | 1.8 | 2.4 | 1.8 | 2.678 |
| P62249 | 40S ribosomal protein S16 OS=Homo sapiens GN=RPS16 PE=1 SV=2 | 21 | 32.9 | 1.2 | 4.4 | 0.5 | 4 | 30.1 | 8.1 | 1.6 | 2.3 | 40 | 34.9 | 2.2 | 4.1 | 2.0 | 22 | 32.9 | 0.8 | 2.1 | 1.6 | 2.665 |
| Q92597 | Protein NDRG1 OS=Homo sapiens GN=NDRG1 PE=1 SV=1 | 6 | 13.2 | 1.1 | 2.2 | 0.9 | 5 | 4.1 | 1.6 | 0.9 | 1.9 | 11 | 11.7 | 1.8 | 3.2 | 1.7 | 2 | 7.6 | 4.6 | 9.2 | 5.0 | 2.655 |
| O43390 | Heterogeneous nuclear ribonucleoprotein R OS=Homo sapiens GN=HNRNPR PE=1 SV=1 | 4 | 10.4 | 1.1 | 3.4 | 1.3 | * | * | * | * | * | 9 | 20.7 | 3.4 | 4.3 | 2.9 | 4 | 9 | 2.4 | 2.5 | 2.0 | 2.641 |
| P22102 | Trifunctional purine biosynthetic protein adenosine-3 OS=Homo sapiens GN=GART PE=1 SV=1 | 2 | 2.2 | 1.7 | 5.6 | 2.0 | * | * | * | * | * | 6 | 2.9 | 2.6 | 3.6 | 2.1 | 2 | 2.8 | 2.1 | 1.3 | 0.6 | 2.609 |
| P06731 | Carcinoembryonic antigen-related cell adhesion molecule 5 OS=Homo sapiens GN=CEACAM5 PE=1 SV=3 | 6 | 8.3 | 2.4 | 3.7 | 2.3 | 36 | 17.5 | 2.1 | 1.0 | 1.9 | 24 | 14.2 | 3.4 | 3.2 | 2.7 | 22 | 17.5 | 3.5 | 2.2 | 2.2 | 2.595 |
| P09327 | Villin-1 OS=Homo sapiens GN=VIL1 PE=1 SV=4 | 12 | 13.7 | 1.1 | 2.5 | 1.5 | 3 | 4.6 | 3.9 | 0.9 | 4.8 | 23 | 11.6 | 4.4 | 4.1 | 2.1 | 8 | 9.2 | 1.6 | 1.5 | 1.5 | 2.585 |
| P35637 | RNA-binding protein FUS OS=Homo sapiens GN=FUS PE=1 SV=1 | 4 | 7.6 | 1.2 | 2.5 | 3.3 | 2 | 7.6 | 1.2 | 0.5 | 1.2 | 3 | 9.9 | 4.1 | 4.6 | 3.6 | 12 | 8.7 | 4.0 | 2.2 | 2.3 | 2.569 |
| Q13838 | Spliceosome RNA helicase DDX39B OS=Homo sapiens GN=DDX39B PE=1 SV=1 | 18 | 14.5 | 1.3 | 3.5 | 1.4 | * | * | * | * | * | 28 | 17.5 | 2.9 | 3.4 | 2.2 | 14 | 10 | 3.3 | 2.6 | 2.8 | 2.569 |
| P55060 | Exportin-2 OS=Homo sapiens GN=CSE1L PE=1 SV=3 | 6 | 7.5 | 1.0 | 3.0 | 1.4 | * | * | * | * | * | 5 | 5.6 | 3.1 | 4.3 | 3.5 | 3 | 6.5 | 2.9 | 1.0 | 1.5 | 2.520 |
| P13164 | Interferon-induced transmembrane protein 1 OS=Homo sapiens GN=IFITM1 PE=1 SV=3;Interferon-induced transmembrane protein 3 OS=Homo sapiens GN=IFITM3 PE=1 SV=2;Interferon-induced transmembrane protein 2 OS=Homo sapiens GN=IFITM2 PE=1 SV=2 | 5 | 13.6 | 1.9 | 3.1 | 2.4 | 4 | 12.8 | 3.6 | 1.2 | 1.5 | 13 | 12.8 | 3.4 | 2.5 | 1.7 | 5 | 13.6 | 4.1 | 2.2 | 2.7 | 2.513 |
| P00492 | Hypoxanthine-guanine phosphoribosyltransferase OS=Homo sapiens GN=HPRT1 PE=1 SV=2 | 6 | 20.2 | 1.3 | 2.3 | 1.7 | 5 | 15.6 | 1.8 | 1.1 | 1.1 | 6 | 15.6 | 2.4 | 3.2 | 3.2 | 4 | 5 | 4.7 | 4.7 | 5.7 | 2.508 |
| P47897 | Glutamine--tRNA ligase OS=Homo sapiens GN=QARS PE=1 SV=1 | * | * | * | * | * | 2 | 1.9 | 3.5 | 2.6 | 2.7 | 7 | 8.6 | 1.2 | 2.1 | 1.2 | 3 | 4.6 | 4.0 | 2.5 | 2.2 | 2.471 |
| A6NHT5 | Homeobox protein HMX3 OS=Homo sapiens GN=HMX3 PE=1 SV=1 | * | * | * | * | * | 2 | 4.2 | 0.7 | 0.2 | 0.8 | 4 | 4.2 | 2.0 | 4.0 | 2.7 | 2 | 4.5 | 4.1 | 5.1 | 3.9 | 2.468 |
| Q9UBR2 | Cathepsin Z OS=Homo sapiens GN=CTSZ PE=1 SV=1 | 8 | 7.3 | 1.9 | 2.9 | 0.7 | * | * | * | * | * | 11 | 7.3 | 3.1 | 3.8 | 2.3 | 10 | 7.3 | 1.6 | 3.4 | 1.9 | 2.465 |
| P14780 | Matrix metalloproteinase-9 OS=Homo sapiens GN=MMP9 PE=1 SV=3 | * | * | * | * | * | 5 | 3.7 | 2.5 | 1.5 | 1.8 | 5 | 3.7 | 3.2 | 3.8 | 2.7 | 4 | 3.7 | 2.4 | 1.9 | 1.7 | 2.458 |
| P51665 | 26S proteasome non-ATPase regulatory subunit 7 OS=Homo sapiens GN=PSMD7 PE=1 SV=2 | 5 | 8.6 | 0.9 | 2.3 | 0.9 | 2 | 5.2 | 5.9 | 5.3 | 1.9 | 6 | 13.3 | 2.5 | 2.5 | 2.3 | 10 | 9.9 | 1.2 | 1.3 | 1.3 | 2.448 |
| O43169 | Cytochrome b5 type B OS=Homo sapiens GN=CYB5B PE=1 SV=2 | 6 | 8.9 | 1.1 | 2.1 | 1.7 | * | * | * | * | * | 5 | 6.2 | 2.9 | 2.4 | 3.1 | 9 | 8.9 | 4.1 | 2.1 | 3.3 | 2.437 |
| Q07020 | 60S ribosomal protein L18 OS=Homo sapiens GN=RPL18 PE=1 SV=2 | 8 | 12.8 | 0.9 | 1.7 | 1.0 | 8 | 12.8 | 2.7 | 1.8 | 1.6 | 11 | 19.7 | 1.7 | 2.1 | 1.4 | 3 | 12.8 | 4.7 | 7.2 | 3.2 | 2.424 |
| Q53GQ0 | Very-long-chain 3-oxoacyl-CoA reductase OS=Homo sapiens GN=HSD17B12 PE=1 SV=2 | 3 | 12.8 | 3.0 | 3.2 | 1.2 | 3 | 12.8 | 4.4 | 2.0 | 2.0 | 16 | 21.8 | 2.4 | 3.2 | 2.0 | 5 | 17.3 | 1.9 | 1.5 | 2.0 | 2.422 |
| P46781 | 40S ribosomal protein S9 OS=Homo sapiens GN=RPS9 PE=1 SV=3 | 17 | 21.1 | 1.1 | 2.2 | 1.0 | 11 | 8.8 | 1.4 | 0.8 | 2.2 | 9 | 13.4 | 4.6 | 5.0 | 2.7 | 22 | 21.1 | 3.8 | 1.7 | 2.0 | 2.402 |
| Q9NR31 | GTP-binding protein SAR1a OS=Homo sapiens GN=SAR1A PE=1 SV=1 | 2 | 26.8 | 0.9 | 2.5 | 0.5 | * | * | * | * | * | 15 | 11.1 | 3.4 | 6.6 | 2.7 | 11 | 50 | 0.8 | 1.8 | 1.3 | 2.399 |
| P23246 | Splicing factor, proline- and glutamine-rich OS=Homo sapiens GN=SFPQ PE=1 SV=2 | 9 | 15.1 | 0.9 | 2.0 | 0.9 | 9 | 13.3 | 1.0 | 0.9 | 2.3 | 7 | 13 | 4.1 | 5.9 | 2.6 | 10 | 14.6 | 3.5 | 2.2 | 1.9 | 2.393 |
| P80188 | Neutrophil gelatinase-associated lipocalin OS=Homo sapiens GN=LCN2 PE=1 SV=2 | 12 | 19.7 | 1.3 | 1.5 | 1.5 | 66 | 46.0 | 6.8 | 0.9 | 1.4 | 28 | 31.8 | 2.1 | 3.8 | 1.4 | 27 | 24.2 | 3.8 | 1.6 | 2.2 | 2.381 |
| P01019 | Angiotensinogen OS=Homo sapiens GN=AGT PE=1 SV=1 | 12 | 15.9 | 2.4 | 1.4 | 1.4 | 6 | 14.8 | 1.7 | 0.9 | 1.3 | 4 | 12 | 6.0 | 5.5 | 3.3 | 12 | 12.4 | 1.2 | 1.0 | 0.8 | 2.374 |
| P38159 | RNA-binding motif protein, X chromosome OS=Homo sapiens GN=RBMX PE=1 SV=3 | 22 | 9.0 | 0.9 | 3.0 | 0.9 | * | * | * | * | * | 22 | 16.4 | 3.8 | 5.0 | 2.1 | 11 | 11 | 1.6 | 1.5 | 2.1 | 2.353 |
| P62851 | 40S ribosomal protein S25 OS=Homo sapiens GN=RPS25 PE=1 SV=1 | 47 | 32.8 | 1.2 | 2.9 | 1.3 | 26 | 15.2 | 1.9 | 0.8 | 2.3 | 38 | 32.8 | 3.6 | 3.6 | 2.4 | 21 | 24.8 | 3.3 | 2.5 | 2.0 | 2.335 |
| Q01105 | Protein SET OS=Homo sapiens GN=SET PE=1 SV=3 | 8 | 19.0 | 1.0 | 1.9 | 1.9 | 19 | 23.8 | 3.6 | 0.9 | 2.6 | 7 | 23.8 | 3.6 | 3.8 | 2.6 | 15 | 19 | 2.2 | 1.5 | 2.5 | 2.329 |
| P80511 | Protein S100-A12 OS=Homo sapiens GN=S100A12 PE=1 SV=2 | 11 | 30.4 | 1.9 | 4.1 | 0.7 | 10 | 30.4 | 3.8 | 1.2 | 1.8 | 3 | 21.7 | 3.2 | 2.4 | 3.7 | 19 | 31.5 | 1.6 | 1.3 | 1.5 | 2.318 |
| P19338 | Nucleolin OS=Homo sapiens GN=NCL PE=1 SV=3 | 124 | 28.7 | 1.1 | 3.5 | 1.6 | 92 | 28.2 | 2.2 | 0.9 | 1.9 | 138 | 31.4 | 3.6 | 3.3 | 2.2 | 115 | 26.8 | 2.8 | 2.4 | 2.3 | 2.313 |
| Q92841 | Probable ATP-dependent RNA helicase DDX17 OS=Homo sapiens GN=DDX17 PE=1 SV=2 | 7 | 12.8 | 1.0 | 2.5 | 1.1 | 4 | 16.6 | 5.8 | 0.9 | 1.9 | 20 | 11.2 | 2.6 | 3.1 | 1.7 | 8 | 14 | 2.6 | 2.3 | 2.1 | 2.311 |
| P46783 | 40S ribosomal protein S10 OS=Homo sapiens GN=RPS10 PE=1 SV=1 | 25 | 25.5 | 1.1 | 4.2 | 0.4 | 13 | 25.5 | 3.1 | 1.0 | 0.6 | 41 | 29.7 | 3.9 | 6.0 | 2.3 | 39 | 44.2 | 0.9 | 1.9 | 1.8 | 2.310 |
| P13797 | Plastin-3 OS=Homo sapiens GN=PLS3 PE=1 SV=4 | 5 | 13.2 | 3.6 | 1.5 | 0.9 | 3 | 11.7 | 0.5 | 1.4 | 1.1 | 3 | 9.5 | 3.4 | 3.1 | 4.5 | 10 | 15.4 | 2.1 | 3.3 | 1.7 | 2.301 |
| P54727 | UV excision repair protein RAD23 homolog B OS=Homo sapiens GN=RAD23B PE=1 SV=1 | 5 | 18.6 | 1.3 | 1.4 | 1.6 | * | * | * | * | * | 4 | 11.2 | 4.1 | 3.5 | 1.8 | 12 | 23.2 | 2.8 | 1.7 | 2.0 | 2.276 |
| O43143 | Pre-mRNA-splicing factor ATP-dependent RNA helicase DHX15 OS=Homo sapiens GN=DHX15 PE=1 SV=2 | 6 | 6.2 | 1.6 | 2.6 | 1.8 | * | * | * | * | * | 7 | 10.8 | 1.5 | 1.7 | 2.5 | 8 | 4.5 | 3.7 | 2.7 | 1.9 | 2.271 |
| P26641 | Elongation factor 1-gamma OS=Homo sapiens GN=EEF1G PE=1 SV=3 | 66 | 27.5 | 1.3 | 3.3 | 2.0 | 68 | 28.8 | 1.6 | 1.3 | 2.4 | 74 | 25.4 | 2.7 | 2.6 | 2.0 | 92 | 27.2 | 3.6 | 2.0 | 1.8 | 2.267 |
| Q99439 | Calponin-2 OS=Homo sapiens GN=CNN2 PE=1 SV=4 | 16 | 12.6 | 3.4 | 2.7 | 1.2 | 8 | 14.2 | 1.2 | 1.6 | 1.1 | 14 | 17.8 | 2.1 | 3.6 | 3.0 | 17 | 17.8 | 1.8 | 3.2 | 1.7 | 2.257 |
| Q9UJ70 | N-acetyl-D-glucosamine kinase OS=Homo sapiens GN=NAGK PE=1 SV=4 | 5 | 7.6 | 1.2 | 2.1 | 1.3 | * | * | * | * | * | 4 | 4.9 | 2.7 | 3.0 | 2.0 | 11 | 12.2 | 2.5 | 3.1 | 1.8 | 2.244 |
| P11940 | Polyadenylate-binding protein 1 OS=Homo sapiens GN=PABPC1 PE=1 SV=2 | 24 | 28.8 | 1.0 | 2.2 | 1.3 | 20 | 13.7 | 1.6 | 1.3 | 2.7 | 34 | 15.9 | 3.3 | 3.8 | 2.3 | 26 | 21.7 | 3.0 | 2.1 | 2.0 | 2.240 |
| P62328 | Thymosin beta-4 OS=Homo sapiens GN=TMSB4X PE=1 SV=2 | 22 | 45.5 | 1.5 | 3.3 | 1.2 | 31 | 45.5 | 1.1 | 1.3 | 1.5 | 24 | 43.2 | 2.4 | 3.0 | 2.5 | 30 | 50 | 3.1 | 3.9 | 2.8 | 2.233 |
| P02649 | Apolipoprotein E OS=Homo sapiens GN=APOE PE=1 SV=1 | 2 | 4.7 | 1.0 | 1.5 | 1.3 | 4 | 12.9 | 1.5 | 0.8 | 0.8 | * | * | * | * | * | 2 | 7.6 | 7.7 | 3.3 | 4.3 | 2.226 |
| Q15233 | Non-POU domain-containing octamer-binding protein OS=Homo sapiens GN=NONO PE=1 SV=4 | 2 | 15.5 | 1.5 | 6.6 | 3.1 | 2 | 9.3 | 0.8 | 0.8 | 0.9 | 5 | 13.6 | 1.6 | 3.3 | 1.4 | 4 | 15.5 | 2.4 | 2.0 | 3.0 | 2.225 |
| P42224 | Signal transducer and activator of transcription 1-alpha/beta OS=Homo sapiens GN=STAT1 PE=1 SV=2 | * | * | * | * | * | 10 | 15.9 | 1.7 | 0.7 | 1.3 | 5 | 12 | 3.4 | 3.9 | 3.6 | 36 | 17.1 | 1.8 | 1.5 | 1.0 | 2.222 |
| P06454 | Prothymosin alpha OS=Homo sapiens GN=PTMA PE=1 SV=2 | 13 | 31.5 | 0.9 | 2.6 | 2.3 | 8 | 13.5 | 3.0 | 2.8 | 3.2 | 3 | 12.6 | 1.8 | 2.8 | 0.6 | 13 | 12.6 | 2.8 | 1.6 | 1.8 | 2.221 |
| P10412 | Histone H1.4 OS=Homo sapiens GN=HIST1H1E PE=1 SV=2 | 98 | 32.9 | 0.7 | 1.2 | 0.7 | 4 | 34.7 | 1.2 | 0.4 | 2.4 | 3 | 31.1 | 10.5 | 2.7 | 2.0 | 94 | 32.9 | 1.0 | 1.4 | 1.0 | 2.198 |
| P09429 | High mobility group protein B1 OS=Homo sapiens GN=HMGB1 PE=1 SV=3 | 31 | 34.9 | 1.3 | 3.0 | 1.6 | 24 | 34.9 | 1.5 | 1.0 | 1.9 | 58 | 38.6 | 3.8 | 3.7 | 2.1 | 46 | 35.3 | 2.4 | 1.9 | 2.2 | 2.194 |
| P26368 | Splicing factor U2AF 65 kDa subunit OS=Homo sapiens GN=U2AF2 PE=1 SV=4 | 2 | 12.4 | 0.8 | 0.2 | 0.5 | * | * | * | * | * | 4 | 23.2 | 3.3 | 4.9 | 3.9 | 6 | 23.2 | 2.0 | 2.0 | 1.4 | 2.191 |
| P13010 | X-ray repair cross-complementing protein 5 OS=Homo sapiens GN=XRCC5 PE=1 SV=3 | * | * | * | * | * | 8 | 7.4 | 0.9 | 1.0 | 1.3 | 24 | 11.2 | 2.9 | 2.4 | 2.3 | 19 | 16.9 | 4.3 | 2.3 | 2.4 | 2.181 |
| P63244 | Receptor of activated protein C kinase 1 OS=Homo sapiens GN=RACK1 PE=1 SV=3 | 7 | 15.1 | 0.8 | 3.3 | 1.1 | * | * | * | * | * | 12 | 15.1 | 2.6 | 4.3 | 1.4 | 5 | 13.6 | 1.1 | 2.9 | 1.5 | 2.169 |
| P39656 | Dolichyl-diphosphooligosaccharide--protein glycosyltransferase 48 kDa subunit OS=Homo sapiens GN=DDOST PE=1 SV=4 | 13 | 18.2 | 1.3 | 1.8 | 1.1 | * | * | * | * | * | 22 | 18 | 3.0 | 2.7 | 1.6 | 20 | 15.8 | 3.2 | 2.6 | 2.4 | 2.166 |
| P15531 | Nucleoside diphosphate kinase A OS=Homo sapiens GN=NME1 PE=1 SV=1 | 9 | 53.3 | 2.7 | 3.9 | 2.8 | 7 | 46.7 | 1.0 | 0.9 | 1.3 | 9 | 46.7 | 4.2 | 1.6 | 1.7 | 11 | 46.7 | 2.1 | 1.6 | 1.7 | 2.163 |
| P05164 | Myeloperoxidase OS=Homo sapiens GN=MPO PE=1 SV=1 | 16 | 16.9 | 1.1 | 2.1 | 1.0 | 89 | 25.6 | 2.6 | 0.9 | 2.0 | 9 | 9.4 | 3.3 | 2.0 | 3.2 | 83 | 29.7 | 4.0 | 1.5 | 1.9 | 2.157 |
| P62847 | 40S ribosomal protein S24 OS=Homo sapiens GN=RPS24 PE=1 SV=1 | 22 | 29.3 | 1.2 | 3.7 | 1.3 | 11 | 18.0 | 2.9 | 1.2 | 1.8 | 26 | 18 | 2.4 | 3.7 | 2.2 | 40 | 29.3 | 1.7 | 1.6 | 1.5 | 2.148 |
| Q9Y224 | UPF0568 protein C14orf166 OS=Homo sapiens GN=C14orf166 PE=1 SV=1 | 13 | 18.0 | 0.6 | 1.4 | 1.0 | * | * | * | * | * | 6 | 23.8 | 3.4 | 3.8 | 2.3 | 8 | 18 | 2.8 | 1.8 | 1.8 | 2.146 |
| P53999 | Activated RNA polymerase II transcriptional coactivator p15 OS=Homo sapiens GN=SUB1 PE=1 SV=3 | 13 | 26.0 | 1.4 | 2.6 | 1.6 | 9 | 26.8 | 1.6 | 1.1 | 1.6 | 8 | 19.7 | 2.8 | 4.3 | 2.3 | 15 | 19.7 | 2.6 | 1.6 | 1.6 | 2.144 |
| O15143 | Actin-related protein 2/3 complex subunit 1B OS=Homo sapiens GN=ARPC1B PE=1 SV=3 | 5 | 10.2 | 1.6 | 1.5 | 1.2 | 4 | 6.7 | 1.3 | 1.3 | 2.3 | 6 | 10.2 | 2.9 | 4.5 | 3.0 | 6 | 6.7 | 1.8 | 2.3 | 2.3 | 2.143 |
| P62081 | 40S ribosomal protein S7 OS=Homo sapiens GN=RPS7 PE=1 SV=1 | 31 | 28.4 | 0.9 | 1.9 | 1.0 | 3 | 25.8 | 4.9 | 2.2 | 2.2 | 14 | 30.4 | 3.6 | 3.6 | 1.6 | 13 | 28.4 | 0.7 | 1.0 | 1.3 | 2.140 |
| P09651 | Heterogeneous nuclear ribonucleoprotein A1 OS=Homo sapiens GN=HNRNPA1 PE=1 SV=5 | 77 | 32.0 | 1.0 | 3.9 | 1.8 | 66 | 30.9 | 1.7 | 0.9 | 2.0 | 76 | 29.8 | 2.4 | 3.5 | 2.1 | 69 | 35.8 | 2.5 | 1.7 | 1.8 | 2.138 |
| Q9UBQ7 | Glyoxylate reductase/hydroxypyruvate reductase OS=Homo sapiens GN=GRHPR PE=1 SV=1 | 2 | 7.9 | 1.8 | 0.8 | 1.1 | 2 | 15.2 | 2.5 | 2.4 | 2.1 | 10 | 15.2 | 2.1 | 3.9 | 2.6 | * | * | * | * | * | 2.134 |
| P62318 | Small nuclear ribonucleoprotein Sm D3 OS=Homo sapiens GN=SNRPD3 PE=1 SV=1 | 33 | 15.1 | 1.4 | 3.2 | 1.5 | 6 | 15.1 | 2.1 | 0.8 | 2.1 | 28 | 15.1 | 2.8 | 3.8 | 1.6 | 21 | 15.1 | 2.2 | 1.9 | 1.7 | 2.130 |
| P62899 | 60S ribosomal protein L31 OS=Homo sapiens GN=RPL31 PE=1 SV=1 | 16 | 32.8 | 1.1 | 3.2 | 0.9 | 3 | 26.4 | 2.6 | 0.8 | 0.5 | 20 | 32.8 | 3.9 | 5.3 | 1.6 | 6 | 17.6 | 1.3 | 2.3 | 1.7 | 2.129 |
| P62829 | 60S ribosomal protein L23 OS=Homo sapiens GN=RPL23 PE=1 SV=1 | 11 | 23.6 | 0.9 | 2.5 | 1.3 | 6 | 23.6 | 3.6 | 1.9 | 3.2 | 29 | 31.4 | 2.2 | 2.6 | 1.5 | 9 | 16.4 | 1.8 | 1.9 | 1.7 | 2.129 |
| P62424 | 60S ribosomal protein L7a OS=Homo sapiens GN=RPL7A PE=1 SV=2 | 13 | 25.2 | 1.5 | 3.4 | 1.3 | 14 | 13.5 | 2.2 | 1.0 | 2.2 | 18 | 22.2 | 1.9 | 4.6 | 1.9 | 15 | 18.8 | 1.6 | 1.8 | 1.6 | 2.123 |
| P07910 | Heterogeneous nuclear ribonucleoproteins C1/C2 OS=Homo sapiens GN=HNRNPC PE=1 SV=4 | 34 | 28.8 | 0.9 | 3.0 | 1.3 | 32 | 29.1 | 2.6 | 1.1 | 1.9 | 42 | 23.5 | 2.8 | 3.4 | 2.1 | 40 | 26.5 | 2.2 | 2.0 | 2.1 | 2.118 |
| P31949 | Protein S100-A11 OS=Homo sapiens GN=S100A11 PE=1 SV=2 | 59 | 41.9 | 3.5 | 2.4 | 1.4 | 60 | 39.0 | 1.4 | 1.6 | 2.3 | 57 | 45.7 | 1.6 | 2.3 | 1.8 | 69 | 45.7 | 1.5 | 3.5 | 2.2 | 2.114 |
| P62263 | 40S ribosomal protein S14 OS=Homo sapiens GN=RPS14 PE=1 SV=3 | 8 | 15.2 | 0.7 | 2.1 | 0.6 | 11 | 23.8 | 1.7 | 0.8 | 1.5 | 8 | 23.8 | 4.9 | 4.4 | 3.1 | 10 | 15.2 | 1.8 | 1.6 | 1.9 | 2.110 |
| P04080 | Cystatin-B OS=Homo sapiens GN=CSTB PE=1 SV=2 | 56 | 85.7 | 1.6 | 2.1 | 2.1 | 69 | 76.5 | 1.9 | 0.9 | 2.5 | 58 | 53.1 | 1.8 | 2.8 | 1.6 | 61 | 76.5 | 2.8 | 2.9 | 2.0 | 2.105 |
| P62241 | 40S ribosomal protein S8 OS=Homo sapiens GN=RPS8 PE=1 SV=2 | 16 | 28.4 | 1.2 | 3.5 | 1.6 | 18 | 19.2 | 1.2 | 1.2 | 1.7 | 15 | 22.1 | 2.7 | 3.0 | 2.1 | 19 | 30.3 | 2.8 | 2.1 | 2.0 | 2.103 |
| P61313 | 60S ribosomal protein L15 OS=Homo sapiens GN=RPL15 PE=1 SV=2 | 38 | 20.6 | 1.5 | 3.8 | 1.4 | 11 | 13.7 | 2.0 | 1.3 | 1.4 | 32 | 16.2 | 3.2 | 3.3 | 2.2 | 37 | 13.7 | 1.3 | 1.6 | 1.2 | 2.102 |
| P40926 | Malate dehydrogenase, mitochondrial OS=Homo sapiens GN=MDH2 PE=1 SV=3 | 223 | 44.1 | 1.1 | 2.6 | 1.3 | 182 | 40.5 | 1.9 | 0.9 | 1.7 | 334 | 47.6 | 3.1 | 3.8 | 2.4 | 223 | 50.6 | 2.4 | 2.0 | 1.8 | 2.099 |
| P47914 | 60S ribosomal protein L29 OS=Homo sapiens GN=RPL29 PE=1 SV=2 | 2 | 14.5 | 2.4 | 2.7 | 0.9 | 9 | 14.5 | 0.8 | 1.0 | 1.4 | 11 | 14.5 | 3.6 | 4.0 | 2.1 | * | * | * | * | * | 2.093 |
| P20160 | Azurocidin OS=Homo sapiens GN=AZU1 PE=1 SV=3 | 2 | 10.4 | 0.5 | 1.0 | 1.0 | 7 | 10.4 | 2.3 | 0.8 | 2.7 | * | * | * | * | * | 13 | 10.4 | 7.1 | 1.3 | 2.0 | 2.090 |
| Q8NC51 | Plasminogen activator inhibitor 1 RNA-binding protein OS=Homo sapiens GN=SERBP1 PE=1 SV=2 | 6 | 13.7 | 1.5 | 2.5 | 2.2 | * | * | * | * | * | 4 | 16.4 | 1.9 | 1.8 | 1.8 | 2 | 9.3 | 2.6 | 2.5 | 0.8 | 2.086 |
| P21796 | Voltage-dependent anion-selective channel protein 1 OS=Homo sapiens GN=VDAC1 PE=1 SV=2 | 34 | 43.1 | 1.4 | 1.5 | 1.8 | 15 | 14.5 | 1.1 | 0.8 | 2.5 | 45 | 42.4 | 3.8 | 3.2 | 2.4 | 35 | 42.4 | 2.4 | 2.0 | 1.9 | 2.079 |
| P19971 | Thymidine phosphorylase OS=Homo sapiens GN=TYMP PE=1 SV=2 | 5 | 9.8 | 1.9 | 2.2 | 0.4 | 10 | 18.9 | 3.1 | 2.8 | 0.9 | 21 | 20.7 | 2.2 | 2.7 | 1.8 | 30 | 33.4 | 1.5 | 3.3 | 1.8 | 2.074 |
| Q00688 | Peptidyl-prolyl cis-trans isomerase FKBP3 OS=Homo sapiens GN=FKBP3 PE=1 SV=1 | 2 | 11.6 | 1.7 | 2.2 | 3.4 | 2 | 19.6 | 1.0 | 0.8 | 0.9 | 5 | 14.7 | 1.7 | 2.6 | 1.3 | 2 | 12.9 | 3.3 | 4.0 | 1.8 | 2.074 |
| P08238 | Heat shock protein HSP 90-beta OS=Homo sapiens GN=HSP90AB1 PE=1 SV=4 | 135 | 46.4 | 1.5 | 3.0 | 1.1 | 127 | 46.7 | 2.2 | 1.1 | 2.2 | 132 | 47.4 | 2.5 | 3.0 | 1.8 | 136 | 49.2 | 2.7 | 1.6 | 1.8 | 2.068 |
| Q92820 | Gamma-glutamyl hydrolase OS=Homo sapiens GN=GGH PE=1 SV=2 | 5 | 15.4 | 0.7 | 2.5 | 1.1 | 5 | 10.1 | 2.2 | 0.8 | 1.7 | 17 | 24.5 | 4.6 | 3.9 | 2.4 | 4 | 14.8 | 1.5 | 1.3 | 1.9 | 2.067 |
| P20290 | Transcription factor BTF3 OS=Homo sapiens GN=BTF3 PE=1 SV=1 | 5 | 19.9 | 1.0 | 1.5 | 1.6 | 2 | 19.9 | 4.2 | 0.8 | 4.3 | 6 | 19.9 | 2.0 | 2.0 | 1.8 | 10 | 19.9 | 2.3 | 1.2 | 1.1 | 2.060 |
| P49321 | Nuclear autoantigenic sperm protein OS=Homo sapiens GN=NASP PE=1 SV=2 | 4 | 4.2 | 0.3 | 1.2 | 0.9 | * | * | * | * | * | 2 | 4.4 | 4.1 | 4.8 | 2.5 | 14 | 4.4 | 1.4 | 1.4 | 1.2 | 2.059 |
| Q9UBQ0 | Vacuolar protein sorting-associated protein 29 OS=Homo sapiens GN=VPS29 PE=1 SV=1 | 5 | 5.5 | 0.7 | 1.5 | 0.7 | 2 | 12.6 | 0.7 | 0.8 | 0.7 | 3 | 5.5 | 5.8 | 6.5 | 2.7 | 9 | 25.8 | 1.2 | 1.4 | 1.2 | 2.058 |
| Q86V81 | THO complex subunit 4 OS=Homo sapiens GN=ALYREF PE=1 SV=3 | 10 | 10.1 | 0.8 | 2.7 | 1.0 | 8 | 10.1 | 1.1 | 0.7 | 2.4 | 9 | 12.8 | 3.4 | 3.3 | 2.0 | 7 | 12.8 | 3.6 | 1.6 | 2.4 | 2.058 |
| P18621 | 60S ribosomal protein L17 OS=Homo sapiens GN=RPL17 PE=1 SV=3 | 15 | 20.1 | 1.3 | 3.2 | 1.3 | 9 | 19.0 | 1.6 | 0.7 | 1.9 | 13 | 19.6 | 2.4 | 3.0 | 1.6 | 9 | 15.8 | 3.1 | 2.5 | 2.5 | 2.050 |
| P23284 | Peptidyl-prolyl cis-trans isomerase B OS=Homo sapiens GN=PPIB PE=1 SV=2 | 87 | 32.9 | 1.4 | 2.8 | 1.6 | 86 | 48.1 | 1.5 | 1.1 | 1.6 | 72 | 32.9 | 3.8 | 2.9 | 1.9 | 68 | 32.9 | 2.0 | 1.9 | 1.8 | 2.050 |
| P26583 | High mobility group protein B2 OS=Homo sapiens GN=HMGB2 PE=1 SV=2 | * | * | * | * | * | 7 | 18.2 | 1.0 | 0.5 | 1.9 | 10 | 22 | 2.6 | 3.1 | 2.2 | 6 | 16.3 | 3.5 | 1.7 | 2.1 | 2.049 |
| O00231 | 26S proteasome non-ATPase regulatory subunit 11 OS=Homo sapiens GN=PSMD11 PE=1 SV=3 | 5 | 9.5 | 0.6 | 1.7 | 0.9 | * | * | * | * | * | 9 | 14.9 | 2.4 | 4.3 | 2.4 | 11 | 5.9 | 2.3 | 1.6 | 1.3 | 2.048 |
| O00584 | Ribonuclease T2 OS=Homo sapiens GN=RNASET2 PE=1 SV=2 | 4 | 6.2 | 0.7 | 1.3 | 1.4 | * | * | * | * | * | 3 | 6.2 | 2.5 | 3.2 | 2.9 | 8 | 6.2 | 2.5 | 1.9 | 1.6 | 2.046 |
| P08574 | Cytochrome c1, heme protein, mitochondrial OS=Homo sapiens GN=CYC1 PE=1 SV=3 | 6 | 8.3 | 0.8 | 1.9 | 1.4 | 5 | 8.0 | 1.6 | 0.7 | 1.7 | 6 | 8.3 | 4.0 | 3.5 | 3.6 | 6 | 12.6 | 1.6 | 1.7 | 1.4 | 2.039 |
| Q9UQ80 | Proliferation-associated protein 2G4 OS=Homo sapiens GN=PA2G4 PE=1 SV=3 | 21 | 34.8 | 1.3 | 4.0 | 1.4 | 2 | 7.6 | 1.6 | 1.2 | 2.3 | 13 | 20.6 | 1.3 | 3.5 | 1.1 | 13 | 25.6 | 2.5 | 2.2 | 1.8 | 2.038 |
| P27797 | Calreticulin OS=Homo sapiens GN=CALR PE=1 SV=1 | 77 | 29.3 | 1.4 | 2.1 | 1.2 | 81 | 35.7 | 1.6 | 1.1 | 1.6 | 69 | 31.7 | 3.9 | 3.2 | 2.2 | 70 | 31.2 | 2.0 | 2.1 | 1.6 | 2.036 |
| Q16762 | Thiosulfate sulfurtransferase OS=Homo sapiens GN=TST PE=1 SV=4 | 5 | 10.1 | 0.8 | 2.9 | 0.8 | * | * | * | * | * | 10 | 22.9 | 2.3 | 3.4 | 1.3 | 5 | 13.5 | 2.9 | 1.8 | 2.0 | 2.035 |
| P62805 | Histone H4 OS=Homo sapiens GN=HIST1H4A PE=1 SV=2 | 191 | 60.2 | 1.8 | 1.4 | 1.1 | 287 | 60.2 | 2.5 | 1.3 | 1.8 | 296 | 59.2 | 4.2 | 1.7 | 2.3 | 268 | 60.2 | 2.5 | 1.8 | 1.8 | 2.034 |
| P31948 | Stress-induced-phosphoprotein 1 OS=Homo sapiens GN=STIP1 PE=1 SV=1 | 20 | 12.9 | 1.0 | 1.8 | 1.1 | 7 | 11.4 | 0.9 | 0.6 | 1.7 | 24 | 14.9 | 3.0 | 3.9 | 2.6 | 11 | 15.3 | 3.4 | 2.4 | 2.4 | 2.026 |
| Q9UMX0 | Ubiquilin-1 OS=Homo sapiens GN=UBQLN1 PE=1 SV=2 | * | * | * | * | * | 5 | 2.2 | 1.6 | 1.2 | 1.9 | 5 | 4.8 | 1.3 | 2.5 | 2.8 | 3 | 2.2 | 3.7 | 1.3 | 1.6 | 2.023 |
| P13693 | Translationally-controlled tumor protein OS=Homo sapiens GN=TPT1 PE=1 SV=1 | 5 | 26.2 | 0.7 | 1.5 | 1.0 | 3 | 9.3 | 1.3 | 1.3 | 1.5 | 2 | 9.3 | 3.8 | 1.4 | 1.8 | 4 | 9.3 | 4.4 | 3.4 | 3.9 | 2.023 |
| P36578 | 60S ribosomal protein L4 OS=Homo sapiens GN=RPL4 PE=1 SV=5 | 25 | 24.6 | 1.0 | 3.4 | 1.1 | 26 | 24.1 | 1.4 | 0.9 | 1.4 | 59 | 25.5 | 2.7 | 3.7 | 2.0 | 18 | 15.7 | 2.5 | 2.2 | 2.0 | 2.023 |
| P78527 | DNA-dependent protein kinase catalytic subunit OS=Homo sapiens GN=PRKDC PE=1 SV=3 | 6 | 2.7 | 1.5 | 2.9 | 2.3 | 5 | 1.4 | 1.8 | 0.8 | 1.8 | 21 | 3.9 | 3.3 | 2.5 | 1.7 | 17 | 3.2 | 1.9 | 1.7 | 2.2 | 2.021 |
| P31943 | Heterogeneous nuclear ribonucleoprotein H OS=Homo sapiens GN=HNRNPH1 PE=1 SV=4 | 10 | 14.9 | 0.9 | 2.5 | 1.1 | 7 | 13.4 | 1.8 | 1.2 | 3.1 | 11 | 19.4 | 2.1 | 2.6 | 2.1 | 12 | 19.4 | 2.5 | 2.3 | 3.4 | 2.020 |
| P62304 | Small nuclear ribonucleoprotein E OS=Homo sapiens GN=SNRPE PE=1 SV=1 | 7 | 25.0 | 1.6 | 4.7 | 2.2 | 9 | 12.0 | 1.5 | 0.9 | 0.5 | 16 | 25 | 2.2 | 2.6 | 1.3 | 7 | 25 | 1.8 | 2.8 | 2.8 | 2.019 |
| O60814 | Histone H2B type 1-K OS=Homo sapiens GN=HIST1H2BK PE=1 SV=3;Histone H2B type F-S OS=Homo sapiens GN=H2BFS PE=1 SV=2;Histone H2B type 1-D OS=Homo sapiens GN=HIST1H2BD PE=1 SV=2;Histone H2B type 1-C/E/F/G/I OS=Homo sapiens GN=HIST1H2BC PE=1 SV=4;Histone H2B type 2-F OS=Homo sapiens GN=HIST2H2BF PE=1 SV=3;Histone H2B type 1-H OS=Homo sapiens GN=HIST1H2BH PE=1 SV=3;Histone H2B type 1-N OS=Homo sapiens GN=HIST1H2BN PE=1 SV=3;Histone H2B type 1-M OS=Homo sapiens GN=HIST1H2BM PE=1 SV=3 | 18 | 47.6 | 1.5 | 1.7 | 1.2 | 46 | 48.4 | 1.6 | 0.6 | 2.4 | 163 | 42.1 | 4.3 | 1.5 | 2.3 | 24 | 38.1 | 2.9 | 2.2 | 1.8 | 2.019 |
| P09525 | Annexin A4 OS=Homo sapiens GN=ANXA4 PE=1 SV=4 | 32 | 49.8 | 1.3 | 2.4 | 1.9 | 22 | 36.4 | 2.0 | 1.1 | 1.4 | 27 | 34.2 | 2.5 | 2.8 | 2.0 | 54 | 49.5 | 2.4 | 2.5 | 1.6 | 2.016 |
| P20962 | Parathymosin OS=Homo sapiens GN=PTMS PE=1 SV=2 | 12 | 22.5 | 2.0 | 3.1 | 1.8 | 21 | 22.5 | 0.8 | 0.9 | 1.1 | 6 | 22.5 | 3.4 | 3.1 | 2.4 | 10 | 22.5 | 1.7 | 1.7 | 1.7 | 2.015 |
| P61019 | Ras-related protein Rab-2A OS=Homo sapiens GN=RAB2A PE=1 SV=1 | 6 | 18.4 | 0.6 | 1.6 | 0.6 | 5 | 6.6 | 1.5 | 0.8 | 1.1 | 3 | 12.3 | 4.4 | 6.4 | 2.3 | 6 | 23.6 | 1.2 | 1.6 | 1.2 | 2.014 |
| P10599 | Thioredoxin OS=Homo sapiens GN=TXN PE=1 SV=3;sp THIO_HUMAN | 61 | 50.0 | 0.9 | 2.1 | 1.7 | 49 | 46.2 | 1.4 | 0.6 | 1.8 | 39 | 43.3 | 2.2 | 3.8 | 2.0 | 62 | 46.2 | 3.5 | 2.1 | 2.0 | 2.012 |
| Q16531 | DNA damage-binding protein 1 OS=Homo sapiens GN=DDB1 PE=1 SV=1 | 3 | 5.9 | 1.9 | 2.4 | 1.0 | * | * | * | * | * | 2 | 3.8 | 2.5 | 2.2 | 2.9 | 2 | 1.9 | 1.8 | 1.4 | 1.5 | 2.010 |
| Q15436 | Protein transport protein Sec23A OS=Homo sapiens GN=SEC23A PE=1 SV=2 | 2 | 3.9 | 1.2 | 2.5 | 0.4 | 5 | 2.7 | 1.6 | 1.8 | 1.2 | 10 | 6.5 | 2.4 | 3.5 | 2.2 | 6 | 8.4 | 1.7 | 3.6 | 1.8 | 2.010 |
| O75367 | Core histone macro-H2A.1 OS=Homo sapiens GN=H2AFY PE=1 SV=4 | 2 | 5.4 | 1.7 | 1.6 | 0.5 | * | * | * | * | * | 6 | 9.9 | 5.0 | 2.6 | 1.4 | 4 | 8.9 | 1.3 | 2.1 | 2.1 | 2.006 |
| Q9UL46 | Proteasome activator complex subunit 2 OS=Homo sapiens GN=PSME2 PE=1 SV=4 | 30 | 42.7 | 1.5 | 2.1 | 1.3 | 22 | 41.8 | 1.3 | 0.9 | 1.5 | 22 | 29.7 | 2.4 | 2.3 | 1.8 | 39 | 42.3 | 4.2 | 2.8 | 2.2 | 2.005 |
| Q96AE4 | Far upstream element-binding protein 1 OS=Homo sapiens GN=FUBP1 PE=1 SV=3 | 5 | 6.8 | 1.7 | 1.8 | 1.1 | * | * | * | * | * | 7 | 13.4 | 3.2 | 3.6 | 0.8 | 6 | 3.6 | 2.1 | 1.6 | 1.5 | 2.002 |
| P62277 | 40S ribosomal protein S13 OS=Homo sapiens GN=RPS13 PE=1 SV=2 | 37 | 52.3 | 1.2 | 2.2 | 1.1 | 24 | 43.7 | 1.8 | 1.1 | 2.0 | 37 | 51 | 2.5 | 3.1 | 2.2 | 25 | 45 | 3.2 | 1.7 | 2.0 | 1.998 |
| P19075 | Tetraspanin-8 OS=Homo sapiens GN=TSPAN8 PE=1 SV=1 | 6 | 10.1 | 1.6 | 1.2 | 3.2 | * | * | * | * | * | 3 | 10.1 | 1.6 | 2.9 | 1.3 | 4 | 10.1 | 2.4 | 1.7 | 2.8 | 1.993 |
| P14314 | Glucosidase 2 subunit beta OS=Homo sapiens GN=PRKCSH PE=1 SV=2 | 16 | 19.7 | 1.4 | 1.7 | 1.0 | 14 | 12.7 | 2.1 | 1.4 | 1.5 | 15 | 9.1 | 3.3 | 3.0 | 2.1 | 19 | 13.8 | 2.7 | 1.6 | 1.3 | 1.993 |
| Q7Z406 | Myosin-14 OS=Homo sapiens GN=MYH14 PE=1 SV=2 | 20 | 12.9 | 0.8 | 1.7 | 2.1 | 4 | 8.8 | 1.9 | 1.7 | 4.3 | 2 | 5.7 | 1.8 | 2.5 | 1.4 | 14 | 13.3 | 2.5 | 1.3 | 1.5 | 1.991 |
| Q9Y3D6 | Mitochondrial fission 1 protein OS=Homo sapiens GN=FIS1 PE=1 SV=2 | 11 | 7.9 | 0.8 | 1.5 | 1.3 | * | * | * | * | * | 12 | 24.3 | 2.9 | 3.4 | 1.8 | 11 | 7.9 | 2.4 | 1.9 | 1.8 | 1.990 |
| Q9Y383 | Putative RNA-binding protein Luc7-like 2 OS=Homo sapiens GN=LUC7L2 PE=1 SV=2 | 7 | 6.6 | 0.8 | 1.8 | 0.9 | 10 | 7.9 | 1.8 | 1.5 | 2.5 | 15 | 13.5 | 3.4 | 3.1 | 2.8 | 8 | 2.3 | 2.0 | 1.3 | 1.9 | 1.990 |
| Q13423 | NAD(P) transhydrogenase, mitochondrial OS=Homo sapiens GN=NNT PE=1 SV=3 | 2 | 2.0 | 1.8 | 1.8 | 0.3 | * | * | * | * | * | 8 | 4.2 | 1.9 | 1.7 | 1.4 | 3 | 5.2 | 3.4 | 3.7 | 1.3 | 1.989 |
| P10809 | 60 kDa heat shock protein, mitochondrial OS=Homo sapiens GN=HSPD1 PE=1 SV=2 | 282 | 61.1 | 1.1 | 3.6 | 0.8 | 225 | 54.5 | 2.7 | 1.0 | 1.2 | 451 | 60.9 | 3.3 | 2.8 | 1.9 | 253 | 61.8 | 1.1 | 2.3 | 1.9 | 1.987 |
| P46777 | 60S ribosomal protein L5 OS=Homo sapiens GN=RPL5 PE=1 SV=3 | 20 | 17.2 | 1.2 | 2.8 | 2.1 | 11 | 14.5 | 1.2 | 0.9 | 1.0 | 26 | 17.2 | 2.4 | 3.3 | 2.0 | 12 | 16.2 | 3.0 | 2.2 | 1.8 | 1.986 |
| Q12906 | Interleukin enhancer-binding factor 3 OS=Homo sapiens GN=ILF3 PE=1 SV=3 | 22 | 11.9 | 1.4 | 2.3 | 1.7 | 23 | 9.2 | 1.7 | 1.0 | 2.4 | 44 | 15.4 | 2.3 | 2.7 | 1.8 | 22 | 11.1 | 2.8 | 1.8 | 2.2 | 1.983 |
| Q14103 | Heterogeneous nuclear ribonucleoprotein D0 OS=Homo sapiens GN=HNRNPD PE=1 SV=1 | 21 | 28.5 | 1.5 | 3.2 | 1.5 | 18 | 13.0 | 1.1 | 0.9 | 1.2 | 17 | 13.5 | 3.3 | 2.2 | 2.0 | 23 | 19.7 | 2.5 | 2.5 | 2.5 | 1.978 |
| P36776 | Lon protease homolog, mitochondrial OS=Homo sapiens GN=LONP1 PE=1 SV=2 | 5 | 3.1 | 0.3 | 2.8 | 1.7 | * | * | * | * | * | 13 | 5.3 | 2.6 | 3.0 | 2.2 | 15 | 10.2 | 1.0 | 2.2 | 1.3 | 1.975 |
| P10515 | Dihydrolipoyllysine-residue acetyltransferase component of pyruvate dehydrogenase complex, mitochondrial OS=Homo sapiens GN=DLAT PE=1 SV=3 | * | * | * | * | * | 7 | 11.1 | 1.1 | 0.9 | 0.8 | 15 | 13.4 | 1.9 | 2.4 | 1.7 | 3 | 7.7 | 4.6 | 2.3 | 3.0 | 1.971 |
| P17858 | ATP-dependent 6-phosphofructokinase, liver type OS=Homo sapiens GN=PFKL PE=1 SV=6 | 9 | 12.4 | 1.2 | 2.0 | 0.9 | 4 | 5.6 | 2.9 | 0.9 | 1.6 | 9 | 4.5 | 3.1 | 3.6 | 2.8 | 11 | 7.3 | 1.5 | 1.2 | 1.1 | 1.970 |
| P39060 | Collagen alpha-1(XVIII) chain OS=Homo sapiens GN=COL18A1 PE=1 SV=5 | * | * | * | * | * | 3 | 1.1 | 1.6 | 1.9 | 2.0 | 4 | 2.1 | 3.7 | 3.2 | 2.1 | 8 | 4 | 0.6 | 0.7 | 0.7 | 1.966 |
| P34932 | Heat shock 70 kDa protein 4 OS=Homo sapiens GN=HSPA4 PE=1 SV=4 | 10 | 5.6 | 2.2 | 3.6 | 1.5 | * | * | * | * | * | 16 | 5.6 | 1.9 | 2.1 | 1.4 | 9 | 8.5 | 1.1 | 1.9 | 1.2 | 1.964 |
| O75340 | Programmed cell death protein 6 OS=Homo sapiens GN=PDCD6 PE=1 SV=1 | 5 | 28.3 | 1.0 | 2.0 | 1.6 | * | * | * | * | * | 8 | 26.7 | 2.7 | 2.8 | 2.6 | 5 | 28.3 | 1.4 | 1.6 | 1.6 | 1.961 |
| P49327 | Fatty acid synthase OS=Homo sapiens GN=FASN PE=1 SV=3 | 12 | 10.5 | 1.3 | 3.1 | 1.2 | 8 | 3.9 | 1.7 | 1.0 | 2.4 | 21 | 11.2 | 1.8 | 3.2 | 1.5 | 53 | 17.1 | 2.7 | 1.6 | 1.7 | 1.960 |
| P49755 | Transmembrane emp24 domain-containing protein 10 OS=Homo sapiens GN=TMED10 PE=1 SV=2 | 7 | 10.5 | 1.5 | 1.6 | 1.1 | 4 | 10.5 | 2.7 | 1.2 | 1.5 | 10 | 14.6 | 3.5 | 3.1 | 1.9 | 12 | 14.6 | 1.8 | 1.8 | 1.6 | 1.955 |
| Q8NFV4 | Protein ABHD11 OS=Homo sapiens GN=ABHD11 PE=1 SV=1 | 6 | 33.0 | 0.6 | 1.9 | 0.7 | * | * | * | * | * | 2 | 27.9 | 2.2 | 4.4 | 3.0 | 11 | 14 | 1.0 | 1.7 | 1.3 | 1.952 |
| P08708 | 40S ribosomal protein S17 OS=Homo sapiens GN=RPS17 PE=1 SV=2 | 17 | 40.0 | 1.1 | 2.9 | 0.9 | 10 | 23.7 | 1.2 | 0.8 | 1.2 | 33 | 23.7 | 3.4 | 3.8 | 2.0 | 9 | 23.7 | 2.4 | 1.7 | 1.6 | 1.951 |
| P02794 | Ferritin heavy chain OS=Homo sapiens GN=FTH1 PE=1 SV=2 | 9 | 20.2 | 4.1 | 1.1 | 0.6 | 13 | 20.2 | 1.2 | 1.7 | 1.8 | 5 | 20.2 | 1.6 | 1.1 | 2.1 | 23 | 20.8 | 2.1 | 4.0 | 2.2 | 1.951 |
| P02786 | Transferrin receptor protein 1 OS=Homo sapiens GN=TFRC PE=1 SV=2 | * | * | * | * | * | 3 | 6.2 | 1.1 | 0.5 | 0.8 | 13 | 8.7 | 1.3 | 3.1 | 1.5 | 4 | 2.9 | 3.8 | 3.5 | 3.4 | 1.949 |
| O95831 | Apoptosis-inducing factor 1, mitochondrial OS=Homo sapiens GN=AIFM1 PE=1 SV=1 | 8 | 12.9 | 1.1 | 1.5 | 1.0 | * | * | * | * | * | 15 | 16.6 | 1.5 | 3.2 | 1.2 | 7 | 9.1 | 2.2 | 3.8 | 1.9 | 1.946 |
| P41250 | Glycine--tRNA ligase OS=Homo sapiens GN=GARS PE=1 SV=3 | 6 | 8.0 | 1.1 | 2.3 | 0.6 | 6 | 6.8 | 3.2 | 1.2 | 1.5 | 25 | 13.3 | 2.9 | 3.2 | 1.8 | 11 | 13.1 | 1.6 | 2.0 | 1.3 | 1.946 |
| P04843 | Dolichyl-diphosphooligosaccharide--protein glycosyltransferase subunit 1 OS=Homo sapiens GN=RPN1 PE=1 SV=1 | 84 | 39.0 | 1.3 | 2.5 | 1.4 | 48 | 23.1 | 1.5 | 0.9 | 1.3 | 70 | 34.9 | 2.8 | 3.5 | 1.9 | 104 | 44.6 | 2.6 | 1.8 | 1.8 | 1.946 |
| O60506 | Heterogeneous nuclear ribonucleoprotein Q OS=Homo sapiens GN=SYNCRIP PE=1 SV=2 | 10 | 19.7 | 0.9 | 3.3 | 1.5 | 19 | 14.9 | 1.4 | 0.9 | 1.9 | 28 | 17.3 | 3.2 | 3.2 | 2.1 | 15 | 24.9 | 1.9 | 1.1 | 1.3 | 1.945 |
| P43243 | Matrin-3 OS=Homo sapiens GN=MATR3 PE=1 SV=2 | 17 | 12.3 | 1.4 | 1.6 | 1.2 | 13 | 7.2 | 2.2 | 2.1 | 2.3 | 61 | 16.6 | 2.6 | 2.5 | 1.4 | 38 | 9.3 | 2.2 | 2.0 | 2.0 | 1.944 |
| Q9Y5Z4 | Heme-binding protein 2 OS=Homo sapiens GN=HEBP2 PE=1 SV=1 | 16 | 13.7 | 1.8 | 2.1 | 1.3 | 2 | 13.7 | 3.9 | 1.8 | 2.2 | 15 | 13.7 | 1.2 | 1.3 | 1.6 | 6 | 18 | 2.4 | 1.7 | 1.6 | 1.943 |
| P20810 | Calpastatin OS=Homo sapiens GN=CAST PE=1 SV=4 | 4 | 10.2 | 0.8 | 1.8 | 1.2 | * | * | * | * | * | 2 | 7.3 | 4.3 | 3.7 | 1.0 | 10 | 6.6 | 1.6 | 1.3 | 1.5 | 1.942 |
| Q9UHV9 | Prefoldin subunit 2 OS=Homo sapiens GN=PFDN2 PE=1 SV=1 | * | * | * | * | * | 3 | 7.8 | 1.4 | 2.1 | 0.8 | 10 | 16.9 | 2.4 | 3.8 | 2.2 | 4 | 16.9 | 1.8 | 1.0 | 1.3 | 1.940 |
| P61247 | 40S ribosomal protein S3a OS=Homo sapiens GN=RPS3A PE=1 SV=2 | 22 | 31.4 | 1.1 | 2.9 | 1.3 | 29 | 30.3 | 1.7 | 0.9 | 2.2 | 12 | 26.5 | 2.5 | 2.6 | 1.8 | 9 | 17.4 | 2.8 | 1.5 | 1.6 | 1.935 |
| Q96AG4 | Leucine-rich repeat-containing protein 59 OS=Homo sapiens GN=LRRC59 PE=1 SV=1 | 13 | 14.7 | 1.4 | 2.5 | 0.8 | 10 | 11.4 | 0.8 | 0.9 | 0.8 | 25 | 16.3 | 4.0 | 4.8 | 2.0 | 16 | 16.3 | 1.4 | 1.9 | 1.5 | 1.927 |
| Q08380 | Galectin-3-binding protein OS=Homo sapiens GN=LGALS3BP PE=1 SV=1 | 12 | 8.7 | 0.7 | 1.6 | 1.0 | 11 | 10.6 | 3.2 | 1.5 | 2.6 | 10 | 11.8 | 2.3 | 2.7 | 2.5 | 22 | 14.2 | 1.4 | 1.8 | 1.3 | 1.926 |
| P37837 | Transaldolase OS=Homo sapiens GN=TALDO1 PE=1 SV=2 | 26 | 30.9 | 0.9 | 1.9 | 1.1 | 20 | 25.8 | 1.3 | 1.2 | 1.5 | 47 | 31.8 | 3.4 | 3.3 | 2.4 | 67 | 41.5 | 2.2 | 1.9 | 1.6 | 1.926 |
| P62917 | 60S ribosomal protein L8 OS=Homo sapiens GN=RPL8 PE=1 SV=2 | 18 | 20.6 | 0.9 | 2.6 | 1.4 | 10 | 16.3 | 1.9 | 0.8 | 1.3 | 13 | 17.9 | 2.5 | 4.0 | 2.1 | 7 | 19.8 | 1.1 | 2.5 | 1.9 | 1.920 |
| Q13409 | Cytoplasmic dynein 1 intermediate chain 2 OS=Homo sapiens GN=DYNC1I2 PE=1 SV=3 | 2 | 5.5 | 3.2 | 2.2 | 1.2 | 2 | 5.5 | 1.5 | 1.1 | 2.8 | * | * | * | * | * | 4 | 7.4 | 2.6 | 0.9 | 1.6 | 1.920 |
| P48444 | Coatomer subunit delta OS=Homo sapiens GN=ARCN1 PE=1 SV=1 | 19 | 15.3 | 1.0 | 2.3 | 1.9 | * | * | * | * | * | 20 | 11.4 | 1.4 | 2.8 | 1.5 | 11 | 5.9 | 2.6 | 1.8 | 1.4 | 1.908 |
| Q9Y5L4 | Mitochondrial import inner membrane translocase subunit Tim13 OS=Homo sapiens GN=TIMM13 PE=1 SV=1 | 3 | 14.7 | 2.1 | 3.5 | 1.8 | 2 | 14.7 | 3.4 | 1.3 | 1.7 | 5 | 14.7 | 1.4 | 1.5 | 0.8 | 3 | 14.7 | 0.9 | 2.7 | 1.5 | 1.908 |
| Q07955 | Serine/arginine-rich splicing factor 1 OS=Homo sapiens GN=SRSF1 PE=1 SV=2 | 37 | 30.2 | 1.4 | 2.6 | 1.6 | 6 | 19.8 | 0.9 | 1.0 | 1.7 | 44 | 28.2 | 2.6 | 3.0 | 1.9 | 32 | 28.2 | 2.5 | 1.7 | 2.2 | 1.904 |
| P39019 | 40S ribosomal protein S19 OS=Homo sapiens GN=RPS19 PE=1 SV=2 | 37 | 42.8 | 1.3 | 2.6 | 1.1 | 28 | 24.8 | 1.4 | 0.8 | 1.5 | 32 | 43.4 | 2.6 | 3.4 | 1.5 | 24 | 24.8 | 2.8 | 1.9 | 2.0 | 1.904 |
| Q99729 | Heterogeneous nuclear ribonucleoprotein A/B OS=Homo sapiens GN=HNRNPAB PE=1 SV=2 | 20 | 16.6 | 1.5 | 2.7 | 1.1 | 10 | 6.0 | 0.8 | 1.1 | 1.7 | 54 | 14.8 | 1.8 | 3.9 | 1.5 | 21 | 14.8 | 2.1 | 2.6 | 2.3 | 1.899 |
| P62750 | 60S ribosomal protein L23a OS=Homo sapiens GN=RPL23A PE=1 SV=1 | 8 | 14.1 | 1.1 | 2.6 | 0.7 | 3 | 28.2 | 2.4 | 1.5 | 2.6 | 10 | 27.6 | 1.9 | 2.9 | 1.5 | 8 | 16 | 1.7 | 1.9 | 1.6 | 1.898 |
| P62753 | 40S ribosomal protein S6 OS=Homo sapiens GN=RPS6 PE=1 SV=1 | 14 | 18.1 | 0.7 | 3.2 | 2.0 | 13 | 18.1 | 1.1 | 0.7 | 1.0 | 11 | 13.7 | 1.8 | 3.4 | 1.6 | 13 | 19.7 | 3.6 | 1.8 | 2.6 | 1.897 |
| Q96HE7 | ERO1-like protein alpha OS=Homo sapiens GN=ERO1A PE=1 SV=2 | 7 | 15.0 | 2.2 | 1.2 | 1.7 | * | * | * | * | * | 2 | 5.3 | 0.4 | 2.2 | 0.4 | 17 | 16.5 | 3.8 | 3.3 | 2.2 | 1.897 |
| P30044 | Peroxiredoxin-5, mitochondrial OS=Homo sapiens GN=PRDX5 PE=1 SV=4 | 133 | 37.4 | 0.8 | 2.9 | 0.7 | 105 | 36.9 | 1.9 | 0.9 | 1.2 | 199 | 41.6 | 4.8 | 3.5 | 2.4 | 77 | 36.9 | 0.7 | 1.0 | 1.6 | 1.889 |
| P30046 | D-dopachrome decarboxylase OS=Homo sapiens GN=DDT PE=1 SV=3 | 12 | 33.9 | 0.8 | 2.1 | 1.4 | * | * | * | * | * | 7 | 33.9 | 1.8 | 3.4 | 2.9 | 5 | 16.1 | 1.5 | 1.1 | 0.8 | 1.884 |
| Q9Y2Q3 | Glutathione S-transferase kappa 1 OS=Homo sapiens GN=GSTK1 PE=1 SV=3 | 10 | 35.8 | 0.6 | 1.6 | 0.6 | 4 | 12.8 | 1.8 | 1.8 | 1.5 | 27 | 43.4 | 3.3 | 3.4 | 2.1 | 9 | 29.2 | 1.3 | 2.5 | 1.5 | 1.876 |
| P00338 | L-lactate dehydrogenase A chain OS=Homo sapiens GN=LDHA PE=1 SV=2 | 145 | 48.2 | 1.3 | 2.3 | 1.2 | 125 | 44.0 | 1.6 | 1.1 | 1.2 | 232 | 45.2 | 2.0 | 3.4 | 2.5 | 205 | 51.8 | 2.1 | 2.1 | 1.8 | 1.875 |
| P00491 | Purine nucleoside phosphorylase OS=Homo sapiens GN=PNP PE=1 SV=2 | 3 | 20.8 | 1.7 | 2.5 | 1.4 | 5 | 20.1 | 1.0 | 0.5 | 0.7 | 4 | 21.1 | 3.0 | 5.0 | 1.4 | 12 | 34.3 | 2.0 | 1.3 | 1.5 | 1.875 |
| P01903 | HLA class II histocompatibility antigen, DR alpha chain OS=Homo sapiens GN=HLA-DRA PE=1 SV=1 | 11 | 11.4 | 2.1 | 1.4 | 1.3 | 11 | 11.4 | 2.2 | 1.1 | 1.3 | 4 | 6.7 | 2.4 | 2.7 | 1.4 | 31 | 21.3 | 2.8 | 1.8 | 1.5 | 1.874 |
| P99999 | sp CYC_HUMAN ;Cytochrome c OS=Homo sapiens GN=CYCS PE=1 SV=2 | 64 | 56.7 | 0.9 | 1.8 | 1.3 | 90 | 56.7 | 1.5 | 0.7 | 1.2 | 100 | 56.7 | 3.2 | 3.2 | 3.0 | 64 | 56.7 | 2.1 | 1.6 | 1.7 | 1.874 |
| Q15365 | Poly(rC)-binding protein 1 OS=Homo sapiens GN=PCBP1 PE=1 SV=2 | 17 | 27.5 | 1.1 | 2.2 | 1.6 | 16 | 24.7 | 1.5 | 1.0 | 1.2 | 49 | 28.9 | 2.7 | 3.5 | 1.8 | 16 | 23.9 | 2.6 | 1.4 | 1.6 | 1.871 |
| Q9ULA0 | Aspartyl aminopeptidase OS=Homo sapiens GN=DNPEP PE=1 SV=1 | 2 | 10.7 | 1.5 | 1.0 | 1.1 | 3 | 6.1 | 1.6 | 0.5 | 1.1 | 11 | 8.2 | 2.3 | 2.3 | 2.3 | 7 | 7.8 | 3.2 | 3.6 | 2.9 | 1.866 |
| Q12905 | Interleukin enhancer-binding factor 2 OS=Homo sapiens GN=ILF2 PE=1 SV=2 | 41 | 20.5 | 1.1 | 2.4 | 1.1 | 28 | 25.4 | 1.7 | 0.9 | 2.1 | 43 | 20.3 | 2.7 | 2.2 | 1.7 | 32 | 20.3 | 2.7 | 2.1 | 2.3 | 1.866 |
| Q08211 | ATP-dependent RNA helicase A OS=Homo sapiens GN=DHX9 PE=1 SV=4 | 16 | 7.5 | 1.1 | 2.4 | 1.1 | 10 | 9.8 | 1.0 | 0.5 | 1.2 | 38 | 11.2 | 2.6 | 3.3 | 2.1 | 24 | 12.1 | 3.5 | 1.9 | 2.1 | 1.864 |
| P05141 | ADP/ATP translocase 2 OS=Homo sapiens GN=SLC25A5 PE=1 SV=7 | 34 | 34.2 | 1.1 | 3.0 | 0.7 | 27 | 30.2 | 2.7 | 0.9 | 1.1 | 60 | 34.6 | 1.8 | 4.3 | 1.4 | 38 | 39.6 | 1.5 | 2.1 | 1.6 | 1.863 |
| P25786 | Proteasome subunit alpha type-1 OS=Homo sapiens GN=PSMA1 PE=1 SV=1 | 13 | 28.5 | 0.9 | 1.8 | 1.1 | 8 | 20.9 | 2.2 | 1.2 | 1.1 | 21 | 24.7 | 2.4 | 3.3 | 2.1 | 15 | 24.3 | 2.6 | 1.9 | 1.4 | 1.862 |
| P05388 | 60S acidic ribosomal protein P0 OS=Homo sapiens GN=RPLP0 PE=1 SV=1 | 47 | 37.9 | 1.3 | 2.4 | 1.1 | 22 | 27.4 | 2.3 | 1.2 | 1.5 | 37 | 34.7 | 2.1 | 3.4 | 1.7 | 39 | 19.2 | 1.7 | 1.9 | 1.6 | 1.860 |
| P14866 | Heterogeneous nuclear ribonucleoprotein L OS=Homo sapiens GN=HNRNPL PE=1 SV=2 | 15 | 24.4 | 0.9 | 1.5 | 1.2 | 10 | 6.1 | 1.1 | 0.9 | 1.7 | 10 | 26.3 | 2.6 | 3.9 | 1.7 | 13 | 23.9 | 3.3 | 1.8 | 2.0 | 1.860 |
| P50991 | T-complex protein 1 subunit delta OS=Homo sapiens GN=CCT4 PE=1 SV=4 | 18 | 9.1 | 0.7 | 1.9 | 1.3 | 8 | 8.7 | 1.5 | 0.9 | 1.4 | 29 | 22.1 | 1.6 | 3.0 | 1.8 | 40 | 21.7 | 3.9 | 2.3 | 2.0 | 1.860 |
| O94760 | N(G),N(G)-dimethylarginine dimethylaminohydrolase 1 OS=Homo sapiens GN=DDAH1 PE=1 SV=3 | 9 | 20.4 | 1.1 | 2.1 | 1.6 | 4 | 13.3 | 2.1 | 1.0 | 1.9 | 6 | 23.9 | 2.5 | 3.2 | 2.1 | 2 | 20.4 | 1.2 | 1.7 | 1.0 | 1.858 |
| P62826 | GTP-binding nuclear protein Ran OS=Homo sapiens GN=RAN PE=1 SV=3 | 24 | 43.5 | 1.4 | 2.6 | 1.7 | 29 | 36.6 | 1.9 | 1.2 | 2.0 | 42 | 35.2 | 1.7 | 2.5 | 1.5 | 41 | 43.5 | 2.0 | 1.9 | 1.7 | 1.857 |
| P50454 | Serpin H1 OS=Homo sapiens GN=SERPINH1 PE=1 SV=2 | 48 | 45.9 | 2.0 | 1.9 | 0.9 | 32 | 31.6 | 1.2 | 1.6 | 1.6 | 38 | 36.4 | 2.5 | 2.4 | 2.4 | 57 | 38.3 | 1.7 | 2.3 | 1.7 | 1.854 |
| P62937 | Peptidyl-prolyl cis-trans isomerase A OS=Homo sapiens GN=PPIA PE=1 SV=2;sp PPIA_HUMAN | 163 | 53.0 | 1.3 | 2.4 | 1.1 | 192 | 53.0 | 2.0 | 1.1 | 1.7 | 228 | 56.7 | 2.4 | 3.1 | 1.9 | 148 | 43.9 | 2.1 | 1.4 | 1.8 | 1.854 |
| P07339 | sp CATD_HUMAN ;Cathepsin D OS=Homo sapiens GN=CTSD PE=1 SV=1 | 95 | 29.9 | 1.7 | 1.6 | 1.0 | 79 | 27.2 | 1.6 | 1.1 | 1.0 | 99 | 18.4 | 2.1 | 3.1 | 1.9 | 213 | 34.5 | 1.3 | 4.0 | 1.5 | 1.853 |
| P42677 | 40S ribosomal protein S27 OS=Homo sapiens GN=RPS27 PE=1 SV=3 | 4 | 29.8 | 1.3 | 2.9 | 1.5 | * | * | * | * | * | 4 | 29.8 | 2.0 | 2.7 | 1.4 | 3 | 29.8 | 1.9 | 1.2 | 1.1 | 1.852 |
| O75390 | Citrate synthase, mitochondrial OS=Homo sapiens GN=CS PE=1 SV=2 | 41 | 41.0 | 0.9 | 2.6 | 0.8 | 45 | 33.9 | 1.3 | 0.8 | 1.7 | 76 | 32 | 2.9 | 4.3 | 2.0 | 45 | 34.8 | 1.5 | 1.5 | 1.5 | 1.851 |
| P82933 | 28S ribosomal protein S9, mitochondrial OS=Homo sapiens GN=MRPS9 PE=1 SV=2 | 3 | 3.3 | 1.3 | 3.3 | 0.7 | * | * | * | * | * | 5 | 3.3 | 1.9 | 1.8 | 1.2 | 3 | 3.3 | 2.3 | 2.3 | 1.7 | 1.850 |
| P28062 | Proteasome subunit beta type-8 OS=Homo sapiens GN=PSMB8 PE=1 SV=3 | 3 | 10.1 | 2.2 | 2.6 | 1.0 | 8 | 17.4 | 1.5 | 0.6 | 2.1 | 5 | 9.4 | 2.1 | 3.2 | 2.5 | 7 | 5.1 | 1.2 | 1.4 | 0.9 | 1.849 |
| O95881 | Thioredoxin domain-containing protein 12 OS=Homo sapiens GN=TXNDC12 PE=1 SV=1 | 3 | 16.9 | 2.2 | 1.9 | 0.4 | * | * | * | * | * | 8 | 22.1 | 2.1 | 3.3 | 2.2 | 3 | 16.9 | 1.3 | 1.5 | 1.4 | 1.848 |
| P00558 | Phosphoglycerate kinase 1 OS=Homo sapiens GN=PGK1 PE=1 SV=3 | 241 | 64.7 | 1.5 | 2.4 | 0.9 | 164 | 64.5 | 1.5 | 0.9 | 1.3 | 246 | 60.7 | 2.1 | 3.5 | 1.9 | 209 | 63.1 | 1.8 | 2.5 | 1.8 | 1.847 |
| P40429 | 60S ribosomal protein L13a OS=Homo sapiens GN=RPL13A PE=1 SV=2 | 8 | 18.2 | 1.1 | 2.8 | 0.5 | 5 | 16.7 | 2.3 | 2.1 | 0.8 | 7 | 18.2 | 2.1 | 3.5 | 2.3 | 10 | 18.2 | 0.9 | 1.9 | 1.6 | 1.843 |
| P63241 | Eukaryotic translation initiation factor 5A-1 OS=Homo sapiens GN=EIF5A PE=1 SV=2;Eukaryotic translation initiation factor 5A-2 OS=Homo sapiens GN=EIF5A2 PE=1 SV=3 | 45 | 13.7 | 1.3 | 2.6 | 1.3 | 52 | 13.1 | 1.7 | 0.9 | 1.4 | 56 | 27.9 | 2.5 | 3.4 | 1.6 | 51 | 13.7 | 1.6 | 1.9 | 1.6 | 1.840 |
| P17987 | T-complex protein 1 subunit alpha OS=Homo sapiens GN=TCP1 PE=1 SV=1 | 5 | 17.4 | 0.9 | 3.1 | 1.1 | * | * | * | * | * | 22 | 23.2 | 1.8 | 2.8 | 1.4 | 7 | 18.7 | 1.8 | 1.7 | 2.1 | 1.839 |
| Q15121 | Astrocytic phosphoprotein PEA-15 OS=Homo sapiens GN=PEA15 PE=1 SV=2 | 9 | 16.9 | 1.1 | 2.1 | 0.8 | 2 | 9.2 | 1.5 | 1.7 | 1.1 | 4 | 16.9 | 4.4 | 3.4 | 1.4 | 5 | 9.2 | 1.2 | 1.5 | 1.6 | 1.839 |
| P67936 | Tropomyosin alpha-4 chain OS=Homo sapiens GN=TPM4 PE=1 SV=3 | 35 | 54.0 | 2.4 | 1.6 | 1.1 | 39 | 59.3 | 1.1 | 1.7 | 1.6 | 10 | 48.4 | 1.6 | 2.1 | 1.4 | 32 | 45.6 | 2.2 | 3.5 | 1.5 | 1.838 |
| P46776 | 60S ribosomal protein L27a OS=Homo sapiens GN=RPL27A PE=1 SV=2 | 8 | 7.4 | 0.9 | 2.4 | 0.8 | * | * | * | * | * | 14 | 20.9 | 2.2 | 2.8 | 1.5 | 4 | 16.2 | 1.4 | 2.8 | 1.9 | 1.836 |
| P51858 | Hepatoma-derived growth factor OS=Homo sapiens GN=HDGF PE=1 SV=1 | 14 | 22.5 | 1.3 | 2.1 | 1.5 | 19 | 21.7 | 1.5 | 1.2 | 2.5 | 15 | 21.7 | 2.4 | 2.3 | 1.8 | 19 | 25.8 | 2.1 | 1.5 | 1.9 | 1.834 |
| Q02878 | 60S ribosomal protein L6 OS=Homo sapiens GN=RPL6 PE=1 SV=3 | 26 | 37.8 | 1.1 | 2.4 | 1.2 | 29 | 35.1 | 1.4 | 0.9 | 1.7 | 46 | 34.4 | 2.5 | 3.3 | 1.8 | 28 | 28.8 | 1.9 | 1.8 | 1.5 | 1.826 |
| P12956 | X-ray repair cross-complementing protein 6 OS=Homo sapiens GN=XRCC6 PE=1 SV=2 | 54 | 32.0 | 0.8 | 1.9 | 1.1 | 40 | 37.3 | 1.7 | 1.1 | 1.9 | 78 | 28.7 | 2.8 | 2.4 | 2.3 | 60 | 26.6 | 2.1 | 2.1 | 1.9 | 1.824 |
| P62906 | 60S ribosomal protein L10a OS=Homo sapiens GN=RPL10A PE=1 SV=2 | 21 | 27.2 | 1.6 | 2.7 | 1.0 | 14 | 19.4 | 2.1 | 1.2 | 2.0 | 24 | 27.6 | 2.1 | 3.4 | 1.4 | 19 | 23.5 | 1.2 | 1.4 | 1.5 | 1.822 |
| P05386 | 60S acidic ribosomal protein P1 OS=Homo sapiens GN=RPLP1 PE=1 SV=1 | 72 | 32.5 | 1.3 | 2.5 | 1.1 | 52 | 32.5 | 1.3 | 0.9 | 1.5 | 76 | 32.5 | 2.6 | 3.4 | 1.9 | 89 | 39.5 | 1.9 | 1.7 | 1.9 | 1.822 |
| P55854 | Small ubiquitin-related modifier 3 OS=Homo sapiens GN=SUMO3 PE=1 SV=2;Small ubiquitin-related modifier 2 OS=Homo sapiens GN=SUMO2 PE=1 SV=3 | 16 | 46.3 | 1.1 | 2.1 | 0.9 | 15 | 12.6 | 1.4 | 0.9 | 2.1 | 19 | 12.6 | 2.2 | 3.5 | 1.5 | 19 | 12.6 | 2.5 | 1.8 | 1.8 | 1.822 |
| Q9UJU6 | Drebrin-like protein OS=Homo sapiens GN=DBNL PE=1 SV=1 | * | * | * | * | * | 3 | 7.0 | 2.2 | 1.4 | 2.5 | 3 | 7 | 1.4 | 2.9 | 1.5 | 2 | 8.6 | 1.5 | 1.1 | 1.5 | 1.821 |
| P52597 | Heterogeneous nuclear ribonucleoprotein F OS=Homo sapiens GN=HNRNPF PE=1 SV=3 | 14 | 20.2 | 0.8 | 2.9 | 1.9 | 9 | 18.3 | 1.9 | 1.6 | 1.8 | 10 | 20.2 | 1.7 | 2.5 | 0.9 | 16 | 16.4 | 2.4 | 1.8 | 1.5 | 1.819 |
| P13796 | Plastin-2 OS=Homo sapiens GN=LCP1 PE=1 SV=6 | 33 | 26.2 | 2.0 | 1.8 | 1.1 | 56 | 30.6 | 1.7 | 0.9 | 1.6 | 22 | 23.6 | 2.4 | 2.8 | 2.0 | 96 | 37 | 2.0 | 1.7 | 1.6 | 1.819 |
| P40121 | Macrophage-capping protein OS=Homo sapiens GN=CAPG PE=1 SV=2 | 36 | 22.7 | 1.7 | 1.5 | 0.9 | 38 | 35.3 | 1.6 | 1.1 | 1.7 | 37 | 19.8 | 2.3 | 2.5 | 1.9 | 94 | 43.7 | 1.5 | 3.2 | 1.4 | 1.815 |
| P01833 | Polymeric immunoglobulin receptor OS=Homo sapiens GN=PIGR PE=1 SV=4 | 32 | 17.9 | 1.3 | 1.5 | 1.5 | 82 | 17.4 | 4.7 | 0.8 | 1.4 | 25 | 14.3 | 1.0 | 2.8 | 1.5 | 58 | 19.1 | 2.1 | 1.3 | 1.9 | 1.812 |
| Q03252 | Lamin-B2 OS=Homo sapiens GN=LMNB2 PE=1 SV=4 | 16 | 18.4 | 1.3 | 2.3 | 1.3 | 5 | 11.9 | 2.6 | 2.3 | 3.6 | 3 | 11 | 0.6 | 1.2 | 1.7 | 20 | 18.4 | 1.9 | 1.2 | 1.2 | 1.808 |
| P28838 | Cytosol aminopeptidase OS=Homo sapiens GN=LAP3 PE=1 SV=3 | 32 | 28.9 | 1.6 | 2.1 | 1.0 | 42 | 30.1 | 1.6 | 1.2 | 1.8 | 39 | 30.8 | 2.0 | 3.0 | 1.8 | 83 | 43 | 1.4 | 2.6 | 1.6 | 1.808 |
| P60174 | Triosephosphate isomerase OS=Homo sapiens GN=TPI1 PE=1 SV=3 | 335 | 51.4 | 1.3 | 2.5 | 1.3 | 305 | 52.1 | 1.6 | 1.0 | 1.4 | 328 | 51.7 | 1.8 | 3.3 | 1.7 | 289 | 55.6 | 2.1 | 1.9 | 1.6 | 1.808 |
| Q96FQ6 | Protein S100-A16 OS=Homo sapiens GN=S100A16 PE=1 SV=1 | 2 | 33.0 | 0.6 | 1.6 | 0.8 | * | * | * | * | * | 4 | 40.8 | 2.1 | 4.2 | 2.2 | 6 | 22.3 | 1.8 | 1.2 | 1.2 | 1.808 |
| O15347 | High mobility group protein B3 OS=Homo sapiens GN=HMGB3 PE=1 SV=4 | 7 | 6.5 | 2.0 | 2.1 | 1.4 | 3 | 6.5 | 0.6 | 0.7 | 1.1 | 5 | 6.5 | 3.3 | 1.5 | 1.8 | 7 | 10.5 | 3.3 | 2.1 | 2.5 | 1.802 |
| P12724 | Eosinophil cationic protein OS=Homo sapiens GN=RNASE3 PE=1 SV=2 | 27 | 16.9 | 0.7 | 3.2 | 0.7 | 27 | 16.9 | 1.3 | 0.6 | 2.0 | 55 | 16.9 | 2.0 | 4.7 | 1.5 | 24 | 16.9 | 2.5 | 0.6 | 1.8 | 1.801 |
| P07858 | Cathepsin B OS=Homo sapiens GN=CTSB PE=1 SV=3 | 8 | 7.7 | 1.5 | 1.2 | 0.8 | 10 | 7.7 | 1.5 | 1.5 | 1.5 | 6 | 5.3 | 2.5 | 1.9 | 1.1 | 12 | 7.7 | 3.0 | 3.2 | 2.5 | 1.799 |
| O14980 | Exportin-1 OS=Homo sapiens GN=XPO1 PE=1 SV=1 | 7 | 5.1 | 1.2 | 3.4 | 1.3 | 3 | 4.8 | 1.0 | 0.7 | 1.6 | 22 | 8.1 | 2.6 | 2.5 | 1.6 | 24 | 8.4 | 2.0 | 1.8 | 1.2 | 1.798 |
| P07900 | Heat shock protein HSP 90-alpha OS=Homo sapiens GN=HSP90AA1 PE=1 SV=5 | 125 | 48.2 | 1.1 | 2.7 | 1.0 | 112 | 42.6 | 1.5 | 1.0 | 1.6 | 107 | 38.4 | 2.0 | 2.9 | 1.8 | 138 | 51.5 | 2.6 | 1.6 | 1.5 | 1.797 |
| P07237 | Protein disulfide-isomerase OS=Homo sapiens GN=P4HB PE=1 SV=3 | 177 | 66.7 | 1.3 | 2.1 | 1.4 | 149 | 51.2 | 1.3 | 1.1 | 1.3 | 181 | 60.8 | 2.9 | 3.0 | 1.5 | 163 | 55.1 | 2.1 | 1.7 | 1.7 | 1.789 |
| P02656 | Apolipoprotein C-III OS=Homo sapiens GN=APOC3 PE=1 SV=1 | 5 | 16.2 | 0.8 | 0.5 | 0.8 | 11 | 27.3 | 1.3 | 1.0 | 1.2 | 2 | 16.2 | 4.3 | 5.4 | 2.9 | 2 | 16.2 | 0.5 | 1.0 | 0.6 | 1.788 |
| Q9Y678 | Coatomer subunit gamma-1 OS=Homo sapiens GN=COPG1 PE=1 SV=1 | 8 | 6.5 | 0.8 | 2.2 | 1.4 | 3 | 5.5 | 2.4 | 1.6 | 1.2 | 24 | 16.1 | 2.4 | 3.5 | 1.5 | 11 | 14.1 | 1.4 | 1.2 | 1.4 | 1.780 |
| Q13162 | Peroxiredoxin-4 OS=Homo sapiens GN=PRDX4 PE=1 SV=1 | 32 | 34.7 | 1.2 | 1.9 | 1.0 | 25 | 29.5 | 2.1 | 0.8 | 1.6 | 33 | 36.2 | 2.2 | 2.8 | 2.0 | 36 | 36.2 | 1.9 | 2.2 | 2.2 | 1.780 |
| P07108 | Acyl-CoA-binding protein OS=Homo sapiens GN=DBI PE=1 SV=2 | 26 | 63.2 | 1.1 | 2.1 | 1.4 | 24 | 41.4 | 1.2 | 0.8 | 1.6 | 15 | 63.2 | 2.2 | 3.7 | 1.8 | 20 | 65.5 | 1.9 | 1.8 | 1.7 | 1.779 |
| P22087 | rRNA 2'-O-methyltransferase fibrillarin OS=Homo sapiens GN=FBL PE=1 SV=2 | 3 | 9.7 | 0.9 | 2.8 | 1.4 | 2 | 5.9 | 0.9 | 1.4 | 1.4 | 3 | 5.9 | 3.0 | 2.2 | 1.5 | 4 | 5.9 | 1.6 | 2.6 | 1.9 | 1.779 |
| Q8WU39 | Marginal zone B- and B1-cell-specific protein OS=Homo sapiens GN=MZB1 PE=1 SV=1 | 11 | 45.5 | 1.6 | 2.5 | 1.4 | 11 | 45.5 | 2.1 | 0.8 | 0.9 | 23 | 45.5 | 3.5 | 2.4 | 1.4 | 14 | 41.3 | 1.6 | 1.4 | 1.2 | 1.777 |
| P48643 | T-complex protein 1 subunit epsilon OS=Homo sapiens GN=CCT5 PE=1 SV=1 | 22 | 12.6 | 1.7 | 3.4 | 1.4 | 9 | 5.0 | 1.4 | 0.8 | 1.0 | 37 | 17.2 | 1.9 | 2.7 | 1.5 | 22 | 16.6 | 1.7 | 2.0 | 1.5 | 1.775 |
| P52566 | Rho GDP-dissociation inhibitor 2 OS=Homo sapiens GN=ARHGDIB PE=1 SV=3 | 2 | 14.9 | 1.4 | 1.4 | 1.3 | 6 | 14.9 | 1.7 | 1.3 | 1.8 | * | * | * | * | * | 4 | 6.5 | 3.5 | 1.7 | 2.5 | 1.774 |
| O75533 | Splicing factor 3B subunit 1 OS=Homo sapiens GN=SF3B1 PE=1 SV=3 | 4 | 3.8 | 1.3 | 2.7 | 1.3 | * | * | * | * | * | 14 | 11.1 | 1.3 | 2.6 | 1.2 | 8 | 4.1 | 2.4 | 1.4 | 1.6 | 1.773 |
| P02788 | Lactotransferrin OS=Homo sapiens GN=LTF PE=1 SV=6;sp TRFL_HUMAN | 35 | 11.1 | 1.3 | 1.7 | 1.0 | 68 | 27.7 | 1.8 | 0.8 | 1.6 | 27 | 15.2 | 1.9 | 2.2 | 1.5 | 74 | 23 | 4.2 | 1.5 | 1.6 | 1.773 |
| P60842 | Eukaryotic initiation factor 4A-I OS=Homo sapiens GN=EIF4A1 PE=1 SV=1 | 13 | 20.0 | 0.8 | 2.3 | 0.9 | 15 | 22.4 | 1.6 | 1.1 | 1.5 | 18 | 24.1 | 1.8 | 2.8 | 1.6 | 21 | 28.1 | 3.1 | 2.0 | 1.7 | 1.767 |
| P12268 | Inosine-5'-monophosphate dehydrogenase 2 OS=Homo sapiens GN=IMPDH2 PE=1 SV=2 | * | * | * | * | * | 3 | 3.7 | 2.5 | 1.3 | 3.0 | 4 | 9.9 | 1.8 | 1.8 | 1.1 | 5 | 7.6 | 1.4 | 1.2 | 1.5 | 1.765 |
| P25788 | Proteasome subunit alpha type-3 OS=Homo sapiens GN=PSMA3 PE=1 SV=2 | 6 | 14.9 | 0.9 | 2.2 | 1.1 | 8 | 5.5 | 1.4 | 1.1 | 1.2 | 9 | 10.2 | 1.6 | 2.8 | 2.6 | 6 | 17.3 | 2.3 | 2.1 | 2.1 | 1.765 |
| P38646 | Stress-70 protein, mitochondrial OS=Homo sapiens GN=HSPA9 PE=1 SV=2 | 107 | 40.4 | 1.1 | 2.4 | 1.0 | 89 | 30.2 | 1.6 | 0.9 | 1.6 | 162 | 37.3 | 2.5 | 3.2 | 1.5 | 98 | 34.2 | 1.9 | 1.7 | 1.5 | 1.764 |
| O00764 | Pyridoxal kinase OS=Homo sapiens GN=PDXK PE=1 SV=1 | 7 | 25.6 | 1.3 | 3.2 | 1.3 | * | * | * | * | * | 6 | 17 | 1.3 | 1.9 | 1.2 | 13 | 16.7 | 1.7 | 2.1 | 1.4 | 1.763 |
| O75208 | Ubiquinone biosynthesis protein COQ9, mitochondrial OS=Homo sapiens GN=COQ9 PE=1 SV=1 | 3 | 3.5 | 0.5 | 1.3 | 0.8 | 4 | 3.5 | 0.7 | 0.4 | 1.3 | 7 | 9.1 | 4.4 | 5.6 | 2.7 | 2 | 3.5 | 1.0 | 0.6 | 0.9 | 1.762 |
| P83731 | 60S ribosomal protein L24 OS=Homo sapiens GN=RPL24 PE=1 SV=1 | 17 | 19.1 | 1.2 | 4.2 | 0.8 | 8 | 17.8 | 2.3 | 0.8 | 1.0 | 41 | 23.6 | 2.2 | 3.1 | 1.4 | 14 | 14 | 0.8 | 1.6 | 1.3 | 1.760 |
| P61204 | ADP-ribosylation factor 3 OS=Homo sapiens GN=ARF3 PE=1 SV=2;ADP-ribosylation factor 1 OS=Homo sapiens GN=ARF1 PE=1 SV=2 | 27 | 47.5 | 1.1 | 2.1 | 1.0 | 26 | 19.9 | 1.4 | 0.8 | 1.3 | 58 | 28.2 | 2.1 | 3.1 | 1.7 | 29 | 54.7 | 2.5 | 2.3 | 1.9 | 1.758 |
| P31946 | 14-3-3 protein beta/alpha OS=Homo sapiens GN=YWHAB PE=1 SV=3 | 20 | 60.6 | 0.8 | 1.8 | 0.8 | 27 | 61.4 | 1.5 | 1.3 | 1.8 | 31 | 67.1 | 2.6 | 1.8 | 2.5 | 12 | 61 | 2.4 | 1.9 | 2.7 | 1.752 |
| P30101 | Protein disulfide-isomerase A3 OS=Homo sapiens GN=PDIA3 PE=1 SV=4 | 151 | 51.3 | 1.1 | 2.2 | 1.1 | 157 | 47.5 | 1.6 | 1.0 | 1.5 | 150 | 45.7 | 3.1 | 2.6 | 1.5 | 149 | 49.1 | 1.9 | 1.7 | 1.5 | 1.752 |
| P55072 | Transitional endoplasmic reticulum ATPase OS=Homo sapiens GN=VCP PE=1 SV=4 | 65 | 33.5 | 1.3 | 2.1 | 1.3 | 43 | 25.2 | 1.6 | 1.2 | 1.1 | 76 | 33.9 | 2.2 | 3.5 | 1.6 | 72 | 37.3 | 1.7 | 1.8 | 1.6 | 1.751 |
| Q15181 | Inorganic pyrophosphatase OS=Homo sapiens GN=PPA1 PE=1 SV=2 | 31 | 29.8 | 1.2 | 1.7 | 1.0 | 3 | 11.1 | 1.8 | 0.7 | 0.7 | 53 | 23.9 | 3.0 | 4.4 | 2.0 | 27 | 27 | 0.9 | 1.8 | 1.6 | 1.749 |
| P09467 | Fructose-1,6-bisphosphatase 1 OS=Homo sapiens GN=FBP1 PE=1 SV=5 | 5 | 23.4 | 1.7 | 1.3 | 1.3 | * | * | * | * | * | 4 | 4.7 | 1.5 | 2.7 | 1.3 | 10 | 15.4 | 1.2 | 3.0 | 1.8 | 1.748 |
| Q04837 | Single-stranded DNA-binding protein, mitochondrial OS=Homo sapiens GN=SSBP1 PE=1 SV=1 | 17 | 45.9 | 1.1 | 1.9 | 1.2 | 14 | 31.1 | 0.8 | 1.0 | 1.5 | 26 | 37.8 | 2.4 | 3.2 | 1.6 | 28 | 33.8 | 2.8 | 1.8 | 1.6 | 1.748 |
| Q7KZF4 | Staphylococcal nuclease domain-containing protein 1 OS=Homo sapiens GN=SND1 PE=1 SV=1 | 10 | 7.7 | 0.9 | 1.6 | 1.2 | 6 | 9.3 | 1.8 | 0.8 | 1.8 | 15 | 10.2 | 2.7 | 2.9 | 1.9 | 29 | 14.1 | 1.7 | 1.9 | 1.5 | 1.748 |
| P42167 | Lamina-associated polypeptide 2, isoforms beta/gamma OS=Homo sapiens GN=TMPO PE=1 SV=2 | 15 | 16.5 | 2.1 | 2.0 | 1.2 | 13 | 13.9 | 2.0 | 0.7 | 1.7 | 27 | 11.2 | 2.1 | 3.5 | 1.2 | 17 | 16.5 | 1.5 | 1.2 | 1.2 | 1.744 |
| P29401 | Transketolase OS=Homo sapiens GN=TKT PE=1 SV=3 | 99 | 35.6 | 1.0 | 1.9 | 1.1 | 86 | 38.2 | 1.8 | 1.0 | 1.9 | 89 | 38 | 2.1 | 2.8 | 1.7 | 108 | 31 | 2.3 | 1.7 | 1.6 | 1.744 |
| P23396 | 40S ribosomal protein S3 OS=Homo sapiens GN=RPS3 PE=1 SV=2 | 21 | 42.8 | 1.1 | 2.1 | 1.1 | 20 | 39.1 | 1.6 | 1.1 | 2.2 | 23 | 35 | 1.8 | 2.1 | 1.4 | 31 | 43.6 | 3.3 | 1.5 | 1.9 | 1.743 |
| Q13510 | Acid ceramidase OS=Homo sapiens GN=ASAH1 PE=1 SV=5 | 6 | 15.4 | 1.4 | 1.9 | 1.6 | 5 | 12.4 | 2.4 | 1.5 | 1.5 | 7 | 13.2 | 1.9 | 2.6 | 0.9 | 22 | 21.8 | 1.3 | 2.4 | 1.3 | 1.743 |
| P23526 | Adenosylhomocysteinase OS=Homo sapiens GN=AHCY PE=1 SV=4 | 40 | 17.4 | 1.1 | 1.9 | 1.1 | 19 | 19.4 | 1.2 | 0.9 | 1.8 | 79 | 24.3 | 3.0 | 3.4 | 1.9 | 21 | 17.4 | 1.5 | 1.4 | 1.4 | 1.739 |
| Q99832 | T-complex protein 1 subunit eta OS=Homo sapiens GN=CCT7 PE=1 SV=2 | 14 | 14.7 | 1.0 | 2.3 | 0.9 | 12 | 12.5 | 1.6 | 0.8 | 1.5 | 29 | 16.4 | 2.2 | 3.2 | 1.6 | 20 | 18.8 | 2.3 | 1.8 | 1.8 | 1.737 |
| Q9Y3Z3 | Deoxynucleoside triphosphate triphosphohydrolase SAMHD1 OS=Homo sapiens GN=SAMHD1 PE=1 SV=2 | * | * | * | * | * | 4 | 2.9 | 1.3 | 0.8 | 0.9 | 9 | 4.5 | 2.8 | 2.6 | 2.1 | 3 | 6.9 | 0.8 | 2.6 | 1.2 | 1.732 |
| P26885 | Peptidyl-prolyl cis-trans isomerase FKBP2 OS=Homo sapiens GN=FKBP2 PE=1 SV=2 | 9 | 23.2 | 1.1 | 1.5 | 1.3 | 4 | 9.2 | 2.3 | 2.1 | 2.2 | 4 | 19.7 | 1.2 | 2.3 | 1.7 | * | * | * | * | * | 1.731 |
| Q15102 | Platelet-activating factor acetylhydrolase IB subunit gamma OS=Homo sapiens GN=PAFAH1B3 PE=1 SV=1 | 3 | 7.8 | 0.9 | 2.0 | 0.8 | 3 | 7.4 | 1.9 | 1.4 | 1.4 | 10 | 11.3 | 2.9 | 3.4 | 1.2 | 2 | 11.3 | 1.2 | 1.9 | 1.2 | 1.728 |
| P43490 | Nicotinamide phosphoribosyltransferase OS=Homo sapiens GN=NAMPT PE=1 SV=1 | 3 | 11.2 | 0.7 | 1.8 | 1.4 | 14 | 22.0 | 2.2 | 1.0 | 1.2 | 54 | 29.1 | 2.0 | 3.1 | 1.7 | 21 | 22.4 | 1.7 | 2.3 | 1.6 | 1.727 |
| P21912 | Succinate dehydrogenase [ubiquinone] iron-sulfur subunit, mitochondrial OS=Homo sapiens GN=SDHB PE=1 SV=3 | 5 | 19.3 | 1.4 | 2.3 | 1.2 | * | * | * | * | * | 4 | 16.4 | 1.5 | 2.3 | 1.3 | 4 | 9.6 | 1.3 | 2.5 | 1.5 | 1.725 |
| Q01469 | Fatty acid-binding protein, epidermal OS=Homo sapiens GN=FABP5 PE=1 SV=3 | 17 | 18.5 | 1.1 | 2.8 | 1.4 | 15 | 18.5 | 2.4 | 0.8 | 1.8 | * | * | * | * | * | 8 | 18.5 | 2.1 | 1.5 | 2.1 | 1.722 |
| P50990 | T-complex protein 1 subunit theta OS=Homo sapiens GN=CCT8 PE=1 SV=4 | 27 | 26.3 | 0.7 | 2.1 | 0.7 | * | * | * | * | * | 36 | 22.4 | 1.6 | 2.8 | 1.6 | 26 | 25.5 | 2.5 | 1.7 | 1.6 | 1.721 |
| P55209 | Nucleosome assembly protein 1-like 1 OS=Homo sapiens GN=NAP1L1 PE=1 SV=1 | 9 | 12.8 | 1.4 | 2.7 | 1.4 | * | * | * | * | * | 15 | 9.7 | 1.4 | 3.0 | 0.9 | 27 | 5.4 | 0.9 | 2.0 | 1.4 | 1.717 |
| P25815 | Protein S100-P OS=Homo sapiens GN=S100P PE=1 SV=2 | 15 | 40.0 | 0.8 | 3.1 | 1.3 | 5 | 13.7 | 1.8 | 0.9 | 0.9 | 21 | 24.2 | 1.3 | 3.0 | 1.0 | 7 | 31.6 | 3.4 | 1.5 | 1.6 | 1.716 |
| P62266 | 40S ribosomal protein S23 OS=Homo sapiens GN=RPS23 PE=1 SV=3 | 5 | 15.4 | 0.6 | 2.0 | 1.0 | 5 | 7.7 | 0.6 | 1.1 | 1.3 | 10 | 23.1 | 2.1 | 4.5 | 1.8 | 2 | 7.7 | 1.5 | 2.3 | 1.7 | 1.713 |
| P53007 | Tricarboxylate transport protein, mitochondrial OS=Homo sapiens GN=SLC25A1 PE=1 SV=2 | 2 | 6.4 | 1.0 | 1.3 | 1.2 | * | * | * | * | * | 6 | 3.9 | 1.8 | 1.8 | 1.4 | 2 | 6.4 | 3.2 | 2.0 | 3.1 | 1.713 |
| P11142 | Heat shock cognate 71 kDa protein OS=Homo sapiens GN=HSPA8 PE=1 SV=1 | 252 | 54.5 | 1.0 | 2.1 | 1.1 | 246 | 54.2 | 1.4 | 0.9 | 1.7 | 372 | 58.4 | 2.1 | 3.0 | 1.5 | 313 | 58.7 | 2.4 | 1.6 | 1.6 | 1.710 |
| P61158 | Actin-related protein 3 OS=Homo sapiens GN=ACTR3 PE=1 SV=3 | 20 | 17.2 | 2.2 | 1.3 | 1.3 | 12 | 16.3 | 0.7 | 1.1 | 1.1 | 34 | 15.3 | 1.9 | 1.4 | 1.5 | 37 | 13.6 | 2.8 | 3.6 | 2.6 | 1.709 |
| P39687 | Acidic leucine-rich nuclear phosphoprotein 32 family member A OS=Homo sapiens GN=ANP32A PE=1 SV=1 | 11 | 17.3 | 0.6 | 1.5 | 0.8 | 5 | 13.7 | 1.8 | 1.4 | 2.1 | 3 | 12.4 | 2.2 | 2.8 | 1.2 | 17 | 12.4 | 2.9 | 1.5 | 2.1 | 1.708 |
| P14868 | Aspartate--tRNA ligase, cytoplasmic OS=Homo sapiens GN=DARS PE=1 SV=2 | 2 | 8.4 | 1.2 | 1.6 | 0.8 | * | * | * | * | * | 13 | 8.4 | 2.1 | 2.8 | 1.5 | 8 | 8.6 | 1.7 | 2.0 | 1.7 | 1.708 |
| P13639 | Elongation factor 2 OS=Homo sapiens GN=EEF2 PE=1 SV=4 | 143 | 29.7 | 1.1 | 2.2 | 1.2 | 120 | 31.6 | 1.9 | 1.3 | 1.2 | 215 | 35.1 | 1.9 | 2.8 | 1.8 | 201 | 36.5 | 1.8 | 1.8 | 1.6 | 1.707 |
| O14818 | Proteasome subunit alpha type-7 OS=Homo sapiens GN=PSMA7 PE=1 SV=1 | 23 | 40.7 | 1.0 | 2.0 | 1.2 | 15 | 41.9 | 1.6 | 1.0 | 1.4 | 28 | 37.1 | 1.8 | 2.9 | 1.6 | 22 | 41.9 | 2.4 | 1.8 | 1.6 | 1.705 |
| P14406 | Cytochrome c oxidase subunit 7A2, mitochondrial OS=Homo sapiens GN=COX7A2 PE=1 SV=1 | 5 | 41.0 | 0.7 | 2.1 | 1.3 | * | * | * | * | * | 14 | 41 | 2.3 | 2.4 | 1.9 | 14 | 56.6 | 1.8 | 1.2 | 1.3 | 1.704 |
| P53597 | Succinate--CoA ligase [ADP/GDP-forming] subunit alpha, mitochondrial OS=Homo sapiens GN=SUCLG1 PE=1 SV=4 | * | * | * | * | * | 4 | 4.3 | 0.7 | 1.0 | 1.0 | 33 | 25.1 | 2.0 | 2.9 | 1.0 | 3 | 13.3 | 2.6 | 2.3 | 2.3 | 1.703 |
| P06703 | Protein S100-A6 OS=Homo sapiens GN=S100A6 PE=1 SV=1 | 47 | 65.6 | 1.9 | 2.2 | 0.4 | 62 | 65.6 | 0.4 | 0.9 | 0.5 | 39 | 55.6 | 1.5 | 5.0 | 1.7 | 68 | 55.6 | 2.6 | 1.6 | 1.4 | 1.703 |
| P62913 | 60S ribosomal protein L11 OS=Homo sapiens GN=RPL11 PE=1 SV=2 | 21 | 12.9 | 0.9 | 1.7 | 1.1 | 15 | 12.9 | 2.0 | 1.2 | 1.6 | 21 | 12.9 | 1.9 | 2.9 | 1.8 | 18 | 12.9 | 1.9 | 1.7 | 1.2 | 1.700 |
| P28070 | Proteasome subunit beta type-4 OS=Homo sapiens GN=PSMB4 PE=1 SV=4 | 6 | 20.5 | 0.7 | 1.6 | 0.5 | 2 | 9.1 | 3.1 | 0.8 | 1.1 | 10 | 22 | 0.9 | 1.4 | 0.9 | 6 | 20.8 | 4.6 | 3.1 | 2.0 | 1.698 |
| Q92688 | Acidic leucine-rich nuclear phosphoprotein 32 family member B OS=Homo sapiens GN=ANP32B PE=1 SV=1 | 7 | 17.1 | 1.2 | 2.3 | 1.3 | 3 | 17.1 | 1.2 | 1.2 | 1.8 | 8 | 13.9 | 2.4 | 1.9 | 1.6 | 4 | 14.3 | 2.4 | 1.4 | 2.0 | 1.693 |
| Q06830 | sp PRDX1_HUMAN ;Peroxiredoxin-1 OS=Homo sapiens GN=PRDX1 PE=1 SV=1 | 161 | 50.3 | 1.2 | 1.6 | 1.1 | 150 | 50.3 | 1.4 | 0.7 | 1.1 | 176 | 52.3 | 2.6 | 2.4 | 1.8 | 179 | 64.3 | 2.8 | 1.9 | 1.5 | 1.691 |
| P41091 | Eukaryotic translation initiation factor 2 subunit 3 OS=Homo sapiens GN=EIF2S3 PE=1 SV=3 | 3 | 10.6 | 1.4 | 3.1 | 1.8 | * | * | * | * | * | 4 | 7.6 | 2.2 | 1.9 | 1.0 | 6 | 7.6 | 1.4 | 0.9 | 1.0 | 1.691 |
| P60866 | 40S ribosomal protein S20 OS=Homo sapiens GN=RPS20 PE=1 SV=1 | 34 | 22.7 | 1.2 | 2.1 | 0.7 | 22 | 22.7 | 1.7 | 0.8 | 1.5 | 50 | 22.7 | 2.5 | 3.3 | 1.9 | 23 | 22.7 | 1.1 | 1.8 | 1.6 | 1.687 |
| P13667 | Protein disulfide-isomerase A4 OS=Homo sapiens GN=PDIA4 PE=1 SV=2 | 104 | 42.9 | 1.3 | 2.5 | 1.3 | 59 | 38.4 | 1.4 | 0.8 | 1.4 | 119 | 37.8 | 2.3 | 2.8 | 1.4 | 86 | 39.4 | 1.5 | 1.7 | 1.5 | 1.685 |
| P10319 | HLA class I histocompatibility antigen, B-58 alpha chain OS=Homo sapiens GN=HLA-B PE=1 SV=1;HLA class I histocompatibility antigen, B-51 alpha chain OS=Homo sapiens GN=HLA-B PE=1 SV=1;HLA class I histocompatibility antigen, B-57 alpha chain OS=Homo sapiens GN=HLA-B PE=1 SV=1;HLA class I histocompatibility antigen, B-15 alpha chain OS=Homo sapiens GN=HLA-B PE=1 SV=2;HLA class I histocompatibility antigen, B-46 alpha chain OS=Homo sapiens GN=HLA-B PE=1 SV=1;HLA class I histocompatibility antigen, B-52 alpha chain OS=Homo sapiens GN=HLA-B PE=1 SV=1;HLA class I histocompatibility antigen, B-53 alpha chain OS=Homo sapiens GN=HLA-B PE=1 SV=1;HLA class I histocompatibility antigen, B-54 alpha chain OS=Homo sapiens GN=HLA-B PE=1 SV=1;HLA class I histocompatibility antigen, B-55 alpha chain OS=Homo sapiens GN=HLA-B PE=1 SV=1;HLA class I histocompatibility antigen, B-56 alpha chain OS=Homo sapiens GN=HLA-B PE=1 SV=1;HLA class I histocompatibility antigen, B-78 alpha chain OS=Homo sapiens GN=HLA-B PE=1 SV=1;HLA class I histocompatibility antigen, B-35 alpha chain OS=Homo sapiens GN=HLA-B PE=1 SV=1;HLA class I histocompatibility antigen, B-59 alpha chain OS=Homo sapiens GN=HLA-B PE=1 SV=1 | 4 | 19.3 | 0.8 | 1.3 | 0.6 | * | * | * | * | * | 9 | 16.9 | 3.5 | 2.6 | 1.4 | 14 | 20.7 | 1.6 | 1.6 | 1.1 | 1.684 |
| Q92945 | Far upstream element-binding protein 2 OS=Homo sapiens GN=KHSRP PE=1 SV=4 | 12 | 18.3 | 1.1 | 1.8 | 1.1 | 11 | 16.9 | 1.8 | 0.9 | 1.9 | 38 | 25.2 | 2.2 | 2.8 | 2.2 | 22 | 22.1 | 1.4 | 1.5 | 1.2 | 1.683 |
| Q9BS26 | Endoplasmic reticulum resident protein 44 OS=Homo sapiens GN=ERP44 PE=1 SV=1 | 10 | 11.1 | 0.9 | 2.3 | 1.4 | 6 | 6.2 | 1.1 | 1.3 | 1.5 | 6 | 6.7 | 1.7 | 3.0 | 1.4 | 8 | 7.1 | 2.1 | 1.8 | 1.5 | 1.680 |
| P62701 | 40S ribosomal protein S4, X isoform OS=Homo sapiens GN=RPS4X PE=1 SV=2 | 21 | 31.9 | 1.2 | 3.1 | 1.1 | 10 | 11.8 | 1.9 | 0.6 | 1.3 | 28 | 29.7 | 2.3 | 2.5 | 1.5 | 10 | 26.6 | 1.2 | 1.8 | 1.5 | 1.679 |
| P24534 | Elongation factor 1-beta OS=Homo sapiens GN=EEF1B2 PE=1 SV=3 | 21 | 22.7 | 0.9 | 2.3 | 0.7 | 17 | 22.7 | 2.1 | 1.1 | 0.9 | 35 | 22.7 | 2.3 | 3.3 | 1.8 | 15 | 15.6 | 1.3 | 1.9 | 1.7 | 1.678 |
| Q04760 | Lactoylglutathione lyase OS=Homo sapiens GN=GLO1 PE=1 SV=4 | 3 | 15.8 | 0.7 | 1.2 | 0.7 | 4 | 29.3 | 1.5 | 0.5 | 2.0 | 5 | 15.8 | 3.6 | 3.3 | 1.7 | * | * | * | * | * | 1.678 |
| P04179 | Superoxide dismutase [Mn], mitochondrial OS=Homo sapiens GN=SOD2 PE=1 SV=2 | 67 | 29.7 | 1.4 | 1.5 | 1.4 | 79 | 55.4 | 1.2 | 1.3 | 1.8 | 63 | 48.6 | 1.8 | 2.2 | 1.9 | 105 | 43.2 | 1.4 | 2.4 | 1.3 | 1.677 |
| P54578 | Ubiquitin carboxyl-terminal hydrolase 14 OS=Homo sapiens GN=USP14 PE=1 SV=3 | 2 | 2.6 | 0.8 | 2.1 | 0.8 | 2 | 2.6 | 2.6 | 1.4 | 3.0 | 9 | 4.9 | 1.2 | 2.7 | 1.1 | 3 | 2.6 | 1.7 | 1.0 | 1.0 | 1.675 |
| Q01813 | ATP-dependent 6-phosphofructokinase, platelet type OS=Homo sapiens GN=PFKP PE=1 SV=2 | 3 | 3.7 | 0.8 | 1.1 | 1.5 | * | * | * | * | * | 4 | 5.7 | 2.3 | 3.5 | 1.8 | 14 | 8.7 | 1.5 | 1.0 | 1.0 | 1.673 |
| O15144 | Actin-related protein 2/3 complex subunit 2 OS=Homo sapiens GN=ARPC2 PE=1 SV=1 | * | * | * | * | * | 9 | 3.7 | 1.6 | 0.7 | 1.8 | 7 | 7.3 | 1.5 | 2.1 | 1.1 | 13 | 21.3 | 2.8 | 1.8 | 2.2 | 1.671 |
| P08311 | sp CATG_HUMAN ;Cathepsin G OS=Homo sapiens GN=CTSG PE=1 SV=2 | * | * | * | * | * | 22 | 20.0 | 1.5 | 1.4 | 3.0 | 5 | 8.6 | 0.9 | 1.0 | 0.9 | 15 | 19.2 | 3.8 | 0.9 | 1.2 | 1.671 |
| P53618 | Coatomer subunit beta OS=Homo sapiens GN=COPB1 PE=1 SV=3 | 3 | 6.3 | 0.5 | 0.9 | 0.8 | 2 | 2.0 | 1.0 | 0.7 | 1.1 | 12 | 13 | 3.8 | 3.8 | 2.6 | 15 | 8.5 | 1.7 | 1.6 | 1.5 | 1.670 |
| Q16181 | Septin-7 OS=Homo sapiens GN=SEPT7 PE=1 SV=2 | 7 | 16.2 | 0.7 | 1.6 | 0.5 | 3 | 8.2 | 1.9 | 1.0 | 1.2 | 5 | 11.2 | 2.3 | 3.4 | 2.8 | 12 | 16.2 | 1.4 | 1.6 | 1.3 | 1.670 |
| P14618 | Pyruvate kinase PKM OS=Homo sapiens GN=PKM PE=1 SV=4 | 183 | 55.0 | 1.7 | 2.1 | 1.0 | 135 | 46.0 | 1.6 | 1.1 | 1.2 | 164 | 45.2 | 1.6 | 2.6 | 1.6 | 226 | 51.6 | 1.9 | 1.9 | 1.6 | 1.665 |
| Q14697 | Neutral alpha-glucosidase AB OS=Homo sapiens GN=GANAB PE=1 SV=3 | 44 | 23.9 | 1.0 | 1.7 | 0.8 | 31 | 20.8 | 1.6 | 1.1 | 1.4 | 59 | 19.8 | 2.9 | 2.5 | 2.1 | 58 | 27.8 | 1.6 | 1.6 | 1.6 | 1.663 |
| P15880 | 40S ribosomal protein S2 OS=Homo sapiens GN=RPS2 PE=1 SV=2 | 23 | 21.8 | 1.1 | 2.5 | 0.9 | 23 | 21.8 | 1.8 | 0.9 | 1.9 | 54 | 21.8 | 2.3 | 2.7 | 1.7 | 21 | 27.6 | 1.1 | 1.3 | 1.4 | 1.663 |
| P14625 | Endoplasmin OS=Homo sapiens GN=HSP90B1 PE=1 SV=1 | 224 | 46.2 | 1.2 | 1.6 | 1.1 | 227 | 37.6 | 1.2 | 0.9 | 1.4 | 239 | 41 | 2.8 | 2.3 | 1.5 | 296 | 46.6 | 2.3 | 2.0 | 1.8 | 1.662 |
| P22234 | Multifunctional protein ADE2 OS=Homo sapiens GN=PAICS PE=1 SV=3 | 14 | 16.7 | 1.1 | 2.9 | 0.9 | * | * | * | * | * | 7 | 9.9 | 1.6 | 2.0 | 1.5 | 12 | 12.9 | 2.0 | 1.3 | 1.2 | 1.662 |
| Q14651 | Plastin-1 OS=Homo sapiens GN=PLS1 PE=1 SV=2 | 13 | 21.8 | 1.2 | 2.4 | 1.1 | * | * | * | * | * | 10 | 8.9 | 1.8 | 2.4 | 1.5 | 9 | 11.9 | 2.3 | 0.6 | 1.1 | 1.660 |
| P53621 | Coatomer subunit alpha OS=Homo sapiens GN=COPA PE=1 SV=2 | 3 | 9.4 | 1.3 | 1.5 | 1.2 | 5 | 3.3 | 0.7 | 0.7 | 1.5 | 17 | 11.4 | 2.8 | 2.8 | 1.9 | 19 | 11.2 | 2.3 | 1.6 | 1.6 | 1.660 |
| P53396 | ATP-citrate synthase OS=Homo sapiens GN=ACLY PE=1 SV=3 | 5 | 10.7 | 1.4 | 3.2 | 0.8 | 2 | 2.0 | 1.2 | 0.5 | 0.9 | 10 | 9 | 1.8 | 3.4 | 1.8 | 5 | 9.1 | 1.4 | 1.9 | 1.6 | 1.657 |
| O43491 | Band 4.1-like protein 2 OS=Homo sapiens GN=EPB41L2 PE=1 SV=1 | 3 | 1.1 | 0.5 | 1.0 | 2.0 | 4 | 3.6 | 0.9 | 0.5 | 0.6 | 12 | 6.9 | 2.3 | 3.7 | 1.5 | 5 | 2.8 | 2.6 | 2.5 | 2.3 | 1.657 |
| P30042 | ES1 protein homolog, mitochondrial OS=Homo sapiens GN=C21orf33 PE=1 SV=3 | 6 | 17.5 | 1.0 | 1.4 | 0.6 | 3 | 14.9 | 1.4 | 1.7 | 1.6 | 7 | 20.5 | 3.5 | 2.1 | 1.6 | * | * | * | * | * | 1.655 |
| P35232 | Prohibitin OS=Homo sapiens GN=PHB PE=1 SV=1 | 85 | 71.0 | 1.1 | 2.4 | 1.0 | 30 | 51.1 | 1.8 | 1.1 | 1.8 | 90 | 63.6 | 2.1 | 2.8 | 1.3 | 50 | 56.2 | 1.3 | 1.6 | 1.5 | 1.655 |
| Q15637 | Splicing factor 1 OS=Homo sapiens GN=SF1 PE=1 SV=4 | 6 | 8.3 | 1.9 | 1.6 | 0.8 | 2 | 6.6 | 0.8 | 0.6 | 0.6 | 5 | 8.6 | 3.3 | 2.8 | 2.5 | * | * | * | * | * | 1.655 |
| Q9Y320 | Thioredoxin-related transmembrane protein 2 OS=Homo sapiens GN=TMX2 PE=1 SV=1 | 3 | 5.1 | 0.9 | 2.2 | 3.9 | 2 | 5.1 | 0.9 | 1.5 | 0.6 | 4 | 5.1 | 1.1 | 1.7 | 1.4 | 6 | 5.1 | 1.9 | 2.0 | 1.5 | 1.652 |
| P20700 | Lamin-B1 OS=Homo sapiens GN=LMNB1 PE=1 SV=2 | 35 | 22.7 | 1.2 | 1.6 | 1.3 | 22 | 21.8 | 1.2 | 1.0 | 1.9 | 18 | 16 | 2.4 | 2.1 | 1.7 | 33 | 17.7 | 2.6 | 1.1 | 1.4 | 1.651 |
| P51991 | Heterogeneous nuclear ribonucleoprotein A3 OS=Homo sapiens GN=HNRNPA3 PE=1 SV=2 | 12 | 20.4 | 1.2 | 2.2 | 1.1 | * | * | * | * | * | 19 | 25.7 | 1.9 | 2.2 | 1.5 | 17 | 16.1 | 1.3 | 1.9 | 1.6 | 1.650 |
| P35221 | Catenin alpha-1 OS=Homo sapiens GN=CTNNA1 PE=1 SV=1 | 12 | 13.7 | 0.6 | 1.6 | 0.9 | * | * | * | * | * | 13 | 18.7 | 2.0 | 3.2 | 1.6 | 5 | 13.8 | 2.0 | 1.3 | 1.6 | 1.650 |
| P62491 | Ras-related protein Rab-11A OS=Homo sapiens GN=RAB11A PE=1 SV=3 | 24 | 38.0 | 1.1 | 1.8 | 0.8 | 9 | 20.8 | 1.1 | 0.7 | 1.5 | 26 | 24.5 | 1.8 | 2.7 | 1.7 | 30 | 38.4 | 2.5 | 2.4 | 1.9 | 1.650 |
| P18124 | 60S ribosomal protein L7 OS=Homo sapiens GN=RPL7 PE=1 SV=1 | 55 | 36.3 | 1.1 | 2.4 | 0.9 | 32 | 25.8 | 1.4 | 0.8 | 1.6 | 50 | 21.4 | 2.1 | 2.8 | 1.5 | 43 | 35.9 | 2.1 | 1.5 | 1.5 | 1.649 |
| P42766 | 60S ribosomal protein L35 OS=Homo sapiens GN=RPL35 PE=1 SV=2 | 3 | 8.1 | 0.8 | 1.1 | 0.6 | 2 | 8.1 | 2.1 | 2.5 | 2.2 | 10 | 8.1 | 3.3 | 2.6 | 1.5 | 2 | 8.1 | 0.9 | 0.7 | 1.9 | 1.649 |
| Q06210 | Glutamine--fructose-6-phosphate aminotransferase [isomerizing] 1 OS=Homo sapiens GN=GFPT1 PE=1 SV=3 | 24 | 17.6 | 1.3 | 2.3 | 1.2 | 11 | 11.3 | 1.8 | 1.0 | 1.2 | 25 | 14.2 | 1.5 | 2.0 | 1.3 | 41 | 17.6 | 3.1 | 1.2 | 1.6 | 1.645 |
| O95336 | 6-phosphogluconolactonase OS=Homo sapiens GN=PGLS PE=1 SV=2 | 10 | 29.5 | 1.6 | 2.6 | 1.4 | 13 | 31.4 | 1.6 | 1.0 | 0.8 | 21 | 36 | 1.8 | 1.9 | 1.8 | 26 | 51.9 | 1.6 | 2.1 | 1.4 | 1.644 |
| P27824 | Calnexin OS=Homo sapiens GN=CANX PE=1 SV=2 | 36 | 20.3 | 1.1 | 1.8 | 1.1 | 27 | 16.0 | 0.8 | 0.8 | 1.1 | 54 | 31.1 | 2.2 | 2.6 | 1.8 | 51 | 22 | 3.1 | 1.8 | 2.0 | 1.644 |
| P17931 | Galectin-3 OS=Homo sapiens GN=LGALS3 PE=1 SV=5 | 87 | 48.0 | 1.0 | 1.7 | 1.2 | 111 | 40.8 | 1.2 | 0.6 | 2.1 | 89 | 50 | 1.8 | 2.9 | 1.1 | 114 | 46.8 | 2.7 | 1.8 | 1.7 | 1.640 |
| O43809 | Cleavage and polyadenylation specificity factor subunit 5 OS=Homo sapiens GN=NUDT21 PE=1 SV=1 | 3 | 33.9 | 1.7 | 2.0 | 1.5 | * | * | * | * | * | 7 | 28.6 | 2.0 | 2.0 | 1.5 | 7 | 26 | 1.3 | 1.1 | 1.1 | 1.640 |
| P07686 | Beta-hexosaminidase subunit beta OS=Homo sapiens GN=HEXB PE=1 SV=3 | 7 | 16.9 | 1.0 | 2.1 | 1.4 | * | * | * | * | * | 3 | 7.4 | 1.2 | 2.3 | 1.4 | 7 | 11.2 | 1.9 | 1.9 | 1.5 | 1.639 |
| P61960 | Ubiquitin-fold modifier 1 OS=Homo sapiens GN=UFM1 PE=1 SV=1 | 16 | 58.8 | 0.9 | 1.5 | 0.6 | 8 | 17.6 | 1.2 | 0.5 | 0.8 | 18 | 58.8 | 2.6 | 5.3 | 2.4 | 24 | 58.8 | 1.1 | 1.1 | 1.1 | 1.636 |
| P61160 | Actin-related protein 2 OS=Homo sapiens GN=ACTR2 PE=1 SV=1 | 17 | 17.0 | 1.1 | 1.3 | 0.7 | 7 | 17.5 | 1.1 | 1.0 | 1.5 | 21 | 11.2 | 2.4 | 3.3 | 1.9 | 23 | 26.6 | 2.0 | 1.9 | 1.6 | 1.636 |
| P12429 | Annexin A3 OS=Homo sapiens GN=ANXA3 PE=1 SV=3 | 12 | 26.3 | 0.9 | 2.5 | 1.9 | 11 | 31.6 | 0.9 | 0.7 | 1.3 | 3 | 12.1 | 2.3 | 1.1 | 1.4 | 18 | 20.4 | 3.3 | 1.7 | 1.8 | 1.636 |
| P52209 | 6-phosphogluconate dehydrogenase, decarboxylating OS=Homo sapiens GN=PGD PE=1 SV=3 | 14 | 19.5 | 0.8 | 1.8 | 1.0 | 9 | 13.9 | 1.4 | 1.1 | 2.3 | 8 | 21.3 | 1.6 | 2.6 | 1.4 | 12 | 19 | 1.8 | 2.2 | 1.3 | 1.633 |
| O00299 | Chloride intracellular channel protein 1 OS=Homo sapiens GN=CLIC1 PE=1 SV=4 | 41 | 35.7 | 1.4 | 2.1 | 1.1 | 61 | 39.0 | 1.1 | 0.7 | 1.9 | 67 | 39 | 2.5 | 2.1 | 1.4 | 70 | 35.7 | 2.3 | 1.3 | 1.6 | 1.633 |
| P60900 | Proteasome subunit alpha type-6 OS=Homo sapiens GN=PSMA6 PE=1 SV=1 | 5 | 19.9 | 1.2 | 1.8 | 0.9 | 11 | 17.9 | 2.4 | 0.8 | 1.3 | 14 | 19.9 | 1.4 | 3.2 | 1.7 | 10 | 15.9 | 1.6 | 1.6 | 1.3 | 1.626 |
| Q99623 | Prohibitin-2 OS=Homo sapiens GN=PHB2 PE=1 SV=2 | 45 | 36.5 | 0.9 | 1.9 | 0.9 | 21 | 23.1 | 1.0 | 0.8 | 1.9 | 95 | 36.8 | 2.5 | 3.0 | 1.7 | 52 | 29.1 | 1.6 | 1.7 | 1.5 | 1.620 |
| P15311 | Ezrin OS=Homo sapiens GN=EZR PE=1 SV=4 | 19 | 21.8 | 0.9 | 1.9 | 0.9 | 11 | 16.0 | 1.2 | 0.7 | 1.1 | 14 | 15.9 | 2.1 | 2.8 | 1.8 | 27 | 28.2 | 2.8 | 1.8 | 1.8 | 1.619 |
| P22626 | Heterogeneous nuclear ribonucleoproteins A2/B1 OS=Homo sapiens GN=HNRNPA2B1 PE=1 SV=2 | 74 | 43.6 | 0.8 | 2.2 | 1.3 | 45 | 43.9 | 1.1 | 0.8 | 1.3 | 106 | 43.9 | 2.4 | 3.1 | 1.7 | 69 | 39.7 | 1.6 | 1.5 | 1.3 | 1.616 |
| P30048 | Thioredoxin-dependent peroxide reductase, mitochondrial OS=Homo sapiens GN=PRDX3 PE=1 SV=3 | 18 | 19.9 | 0.8 | 1.9 | 1.2 | 15 | 16.8 | 2.1 | 1.0 | 2.1 | 38 | 21.5 | 1.7 | 2.2 | 1.3 | 19 | 26.2 | 1.7 | 1.7 | 1.1 | 1.615 |
| P16070 | CD44 antigen OS=Homo sapiens GN=CD44 PE=1 SV=3 | 10 | 3.8 | 1.6 | 1.6 | 1.8 | 6 | 1.6 | 0.9 | 0.8 | 1.6 | 4 | 1.6 | 1.3 | 2.4 | 1.9 | 13 | 2.7 | 1.8 | 2.1 | 1.6 | 1.615 |
| P05387 | 60S acidic ribosomal protein P2 OS=Homo sapiens GN=RPLP2 PE=1 SV=1 | 91 | 80.9 | 1.0 | 1.8 | 0.9 | 79 | 77.4 | 1.3 | 0.9 | 1.6 | 86 | 69.6 | 2.3 | 2.6 | 1.4 | 106 | 73.9 | 2.4 | 1.6 | 1.6 | 1.614 |
| P27635 | 60S ribosomal protein L10 OS=Homo sapiens GN=RPL10 PE=1 SV=4 | 10 | 14.5 | 1.1 | 2.1 | 0.9 | 8 | 15.0 | 0.9 | 0.7 | 0.9 | 18 | 15.4 | 2.3 | 2.9 | 1.8 | 9 | 12.6 | 2.4 | 1.7 | 1.8 | 1.613 |
| P35270 | Sepiapterin reductase OS=Homo sapiens GN=SPR PE=1 SV=1 | 2 | 19.5 | 1.6 | 3.6 | 0.8 | * | * | * | * | * | 5 | 19.5 | 1.8 | 1.6 | 0.9 | 8 | 19.9 | 0.8 | 1.7 | 1.3 | 1.613 |
| P16949 | Stathmin OS=Homo sapiens GN=STMN1 PE=1 SV=3 | 12 | 20.1 | 1.3 | 2.2 | 1.0 | 4 | 24.2 | 1.5 | 0.5 | 1.6 | 22 | 14.1 | 2.3 | 2.8 | 1.4 | 9 | 34.2 | 1.9 | 1.2 | 1.3 | 1.610 |
| Q9NYU2 | UDP-glucose:glycoprotein glucosyltransferase 1 OS=Homo sapiens GN=UGGT1 PE=1 SV=3 | 5 | 3.2 | 0.9 | 0.4 | 0.4 | 3 | 3.4 | 0.4 | 0.2 | 0.8 | 5 | 3.2 | 3.6 | 4.6 | 2.8 | 13 | 3.6 | 2.1 | 1.5 | 1.3 | 1.610 |
| Q00839 | Heterogeneous nuclear ribonucleoprotein U OS=Homo sapiens GN=HNRNPU PE=1 SV=6 | 35 | 18.2 | 0.8 | 1.8 | 0.9 | 28 | 20.5 | 1.4 | 1.1 | 1.4 | 66 | 19.2 | 2.5 | 2.8 | 1.9 | 25 | 13 | 1.4 | 1.6 | 1.2 | 1.608 |
| P06748 | Nucleophosmin OS=Homo sapiens GN=NPM1 PE=1 SV=2 | 96 | 39.1 | 0.9 | 1.8 | 1.0 | 56 | 39.1 | 1.4 | 0.9 | 1.5 | 101 | 41.5 | 2.4 | 2.9 | 1.5 | 101 | 32 | 1.6 | 1.6 | 1.6 | 1.608 |
| P68104 | Elongation factor 1-alpha 1 OS=Homo sapiens GN=EEF1A1 PE=1 SV=1;Putative elongation factor 1-alpha-like 3 OS=Homo sapiens GN=EEF1A1P5 PE=5 SV=1 | 223 | 43.5 | 0.8 | 2.2 | 0.7 | 212 | 37.4 | 1.4 | 1.0 | 0.9 | 301 | 43.1 | 2.5 | 3.0 | 1.7 | 281 | 45.2 | 1.8 | 1.8 | 1.7 | 1.607 |
| P11586 | C-1-tetrahydrofolate synthase, cytoplasmic OS=Homo sapiens GN=MTHFD1 PE=1 SV=3 | 9 | 11.4 | 1.0 | 2.8 | 1.0 | 8 | 10.3 | 1.4 | 1.2 | 1.6 | 25 | 11.6 | 1.9 | 2.7 | 1.4 | 9 | 11.6 | 1.3 | 1.4 | 0.9 | 1.604 |
| P06753 | Tropomyosin alpha-3 chain OS=Homo sapiens GN=TPM3 PE=1 SV=2 | 15 | 35.8 | 1.1 | 1.5 | 1.3 | 8 | 40.7 | 1.6 | 1.3 | 1.5 | 11 | 29.1 | 1.4 | 3.1 | 1.1 | 13 | 31.9 | 1.5 | 2.3 | 1.4 | 1.603 |
| P09960 | Leukotriene A-4 hydrolase OS=Homo sapiens GN=LTA4H PE=1 SV=2 | * | * | * | * | * | 6 | 9.2 | 1.2 | 0.3 | 1.3 | 10 | 11 | 2.2 | 2.3 | 2.6 | 11 | 13.4 | 0.8 | 2.1 | 1.1 | 1.602 |
| P37802 | Transgelin-2 OS=Homo sapiens GN=TAGLN2 PE=1 SV=3 | 46 | 55.8 | 1.5 | 2.0 | 0.8 | 51 | 59.8 | 1.4 | 1.1 | 1.5 | 55 | 55.8 | 2.3 | 2.7 | 1.6 | 34 | 55.3 | 1.0 | 1.8 | 1.4 | 1.602 |
| P62269 | 40S ribosomal protein S18 OS=Homo sapiens GN=RPS18 PE=1 SV=3 | 27 | 30.3 | 0.9 | 1.9 | 1.4 | 26 | 38.8 | 1.6 | 0.6 | 2.0 | 35 | 31.6 | 2.0 | 2.4 | 1.3 | 19 | 35.5 | 1.7 | 1.8 | 1.4 | 1.600 |
| Q15084 | Protein disulfide-isomerase A6 OS=Homo sapiens GN=PDIA6 PE=1 SV=1 | 99 | 44.3 | 1.3 | 1.9 | 1.3 | 62 | 34.1 | 1.3 | 0.9 | 1.3 | 107 | 47 | 2.6 | 2.4 | 1.5 | 95 | 43.9 | 1.8 | 1.6 | 1.3 | 1.600 |
| P62333 | 26S protease regulatory subunit 10B OS=Homo sapiens GN=PSMC6 PE=1 SV=1 | 12 | 3.6 | 0.9 | 1.6 | 1.3 | 4 | 7.5 | 1.1 | 0.7 | 1.5 | 14 | 10.5 | 2.1 | 2.3 | 1.5 | 12 | 6.7 | 2.8 | 1.8 | 2.2 | 1.600 |
| P42765 | 3-ketoacyl-CoA thiolase, mitochondrial OS=Homo sapiens GN=ACAA2 PE=1 SV=2 | 20 | 36.5 | 1.2 | 1.1 | 1.1 | 18 | 28.7 | 1.1 | 0.9 | 1.8 | 37 | 26.7 | 2.2 | 3.1 | 1.8 | 24 | 25.9 | 1.3 | 2.1 | 1.7 | 1.598 |
| P10155 | 60 kDa SS-A/Ro ribonucleoprotein OS=Homo sapiens GN=TROVE2 PE=1 SV=2 | 6 | 7.6 | 2.2 | 2.0 | 1.8 | * | * | * | * | * | 4 | 4.1 | 1.2 | 1.0 | 1.1 | 4 | 4.6 | 1.9 | 1.6 | 1.3 | 1.597 |
| Q16629 | Serine/arginine-rich splicing factor 7 OS=Homo sapiens GN=SRSF7 PE=1 SV=1 | 5 | 16.8 | 0.8 | 1.9 | 1.3 | * | * | * | * | * | 4 | 7.1 | 4.0 | 1.8 | 0.8 | 5 | 13 | 1.3 | 0.9 | 0.7 | 1.594 |
| P49419 | Alpha-aminoadipic semialdehyde dehydrogenase OS=Homo sapiens GN=ALDH7A1 PE=1 SV=5 | 3 | 9.5 | 0.7 | 1.1 | 0.6 | 2 | 5.8 | 2.3 | 1.5 | 1.6 | 15 | 13 | 2.1 | 2.0 | 1.4 | 6 | 12.4 | 2.8 | 1.6 | 1.5 | 1.593 |
| O15371 | Eukaryotic translation initiation factor 3 subunit D OS=Homo sapiens GN=EIF3D PE=1 SV=1 | 5 | 2.6 | 0.5 | 1.7 | 1.4 | * | * | * | * | * | 7 | 4.7 | 1.4 | 2.3 | 1.4 | 5 | 2.6 | 1.8 | 2.2 | 1.5 | 1.593 |
| P05783 | Keratin, type I cytoskeletal 18 OS=Homo sapiens GN=KRT18 PE=1 SV=2 | 177 | 76.0 | 1.0 | 2.4 | 1.7 | 22 | 35.8 | 0.6 | 0.6 | 1.2 | 145 | 64.9 | 2.3 | 2.7 | 1.6 | 107 | 67 | 2.3 | 1.3 | 1.7 | 1.592 |
| P26640 | Valine--tRNA ligase OS=Homo sapiens GN=VARS PE=1 SV=4 | 5 | 5.0 | 1.2 | 1.6 | 1.4 | * | * | * | * | * | 17 | 9 | 1.3 | 1.9 | 1.3 | 10 | 6.5 | 2.6 | 1.5 | 1.5 | 1.592 |
| P62258 | 14-3-3 protein epsilon OS=Homo sapiens GN=YWHAE PE=1 SV=1 | 30 | 48.2 | 0.7 | 1.4 | 1.0 | 27 | 54.5 | 2.0 | 1.1 | 1.8 | 16 | 36.9 | 1.8 | 2.7 | 2.3 | 21 | 40.4 | 1.6 | 1.1 | 1.2 | 1.587 |
| P09972 | Fructose-bisphosphate aldolase C OS=Homo sapiens GN=ALDOC PE=1 SV=2 | 16 | 19.8 | 1.0 | 1.6 | 1.4 | 13 | 19.0 | 1.1 | 0.8 | 1.8 | 13 | 23.6 | 1.5 | 2.8 | 1.8 | 33 | 19.5 | 2.4 | 1.2 | 1.4 | 1.586 |
| Q16851 | UTP--glucose-1-phosphate uridylyltransferase OS=Homo sapiens GN=UGP2 PE=1 SV=5 | 24 | 17.1 | 0.8 | 1.3 | 0.7 | * | * | * | * | * | 12 | 15.2 | 1.7 | 3.1 | 2.0 | 14 | 23 | 1.6 | 1.4 | 1.3 | 1.585 |
| P26599 | Polypyrimidine tract-binding protein 1 OS=Homo sapiens GN=PTBP1 PE=1 SV=1 | 58 | 50.1 | 1.1 | 2.6 | 1.5 | 45 | 22.4 | 1.3 | 0.9 | 1.5 | 83 | 51.6 | 1.6 | 2.0 | 1.5 | 107 | 55.6 | 1.9 | 1.5 | 1.5 | 1.584 |
| P61769 | sp B2MG_HUMAN ;Beta-2-microglobulin OS=Homo sapiens GN=B2M PE=1 SV=1 | 5 | 16.8 | 1.0 | 1.7 | 1.4 | 23 | 19.3 | 1.4 | 0.5 | 1.5 | 10 | 16.8 | 2.0 | 1.8 | 1.3 | 11 | 16.8 | 3.1 | 1.7 | 2.3 | 1.581 |
| Q9Y3I0 | tRNA-splicing ligase RtcB homolog OS=Homo sapiens GN=RTCB PE=1 SV=1 | 5 | 5.0 | 1.5 | 1.5 | 1.1 | * | * | * | * | * | 5 | 5.9 | 2.8 | 1.9 | 1.6 | 6 | 9.5 | 1.0 | 1.3 | 1.1 | 1.579 |
| P42704 | Leucine-rich PPR motif-containing protein, mitochondrial OS=Homo sapiens GN=LRPPRC PE=1 SV=3 | 24 | 8.2 | 0.8 | 2.2 | 1.2 | 17 | 7.8 | 1.5 | 0.9 | 1.2 | 79 | 15.3 | 2.2 | 2.3 | 1.3 | 60 | 17.4 | 1.9 | 1.9 | 1.8 | 1.579 |
| P61088 | Ubiquitin-conjugating enzyme E2 N OS=Homo sapiens GN=UBE2N PE=1 SV=1 | 27 | 35.5 | 0.8 | 1.7 | 0.6 | 32 | 36.8 | 1.4 | 1.2 | 1.6 | 48 | 36.8 | 2.5 | 3.4 | 1.8 | 42 | 48 | 1.3 | 1.2 | 1.2 | 1.578 |
| P46940 | Ras GTPase-activating-like protein IQGAP1 OS=Homo sapiens GN=IQGAP1 PE=1 SV=1 | 55 | 24.5 | 1.4 | 2.1 | 1.4 | 32 | 15.6 | 1.2 | 0.8 | 1.0 | 32 | 15.3 | 1.3 | 2.5 | 1.3 | 54 | 22.9 | 2.2 | 2.1 | 1.4 | 1.577 |
| P35579 | Myosin-9 OS=Homo sapiens GN=MYH9 PE=1 SV=4 | 317 | 43.0 | 1.1 | 2.0 | 1.1 | 218 | 37.0 | 1.2 | 1.1 | 1.6 | 261 | 37 | 1.5 | 2.4 | 1.6 | 268 | 39.1 | 1.8 | 1.9 | 1.5 | 1.577 |
| P22392 | Nucleoside diphosphate kinase B OS=Homo sapiens GN=NME2 PE=1 SV=1 | 47 | 67.8 | 1.5 | 2.5 | 1.2 | 13 | 50.7 | 0.8 | 0.7 | 1.1 | 43 | 48.7 | 2.2 | 2.8 | 1.4 | 54 | 69.7 | 1.3 | 1.9 | 1.4 | 1.574 |
| P78417 | Glutathione S-transferase omega-1 OS=Homo sapiens GN=GSTO1 PE=1 SV=2 | 11 | 22.0 | 1.5 | 1.4 | 1.5 | 30 | 17.0 | 0.9 | 0.9 | 1.8 | 31 | 20.7 | 1.6 | 2.3 | 1.1 | 33 | 28.2 | 2.5 | 1.7 | 1.4 | 1.574 |
| P55011 | Solute carrier family 12 member 2 OS=Homo sapiens GN=SLC12A2 PE=1 SV=1 | 3 | 2.8 | 1.8 | 1.9 | 1.9 | 7 | 5.9 | 1.2 | 1.3 | 1.2 | 8 | 5.4 | 1.3 | 1.8 | 1.6 | 9 | 7.2 | 1.8 | 1.6 | 1.4 | 1.573 |
| P50914 | 60S ribosomal protein L14 OS=Homo sapiens GN=RPL14 PE=1 SV=4 | 5 | 11.2 | 0.9 | 2.1 | 0.5 | 5 | 11.2 | 3.0 | 1.3 | 1.1 | 11 | 11.2 | 2.4 | 2.6 | 1.8 | 9 | 11.2 | 0.8 | 0.8 | 1.1 | 1.571 |
| Q06323 | Proteasome activator complex subunit 1 OS=Homo sapiens GN=PSME1 PE=1 SV=1 | 53 | 51.0 | 1.3 | 1.3 | 1.1 | 46 | 59.4 | 1.3 | 1.0 | 1.3 | 74 | 52.6 | 2.4 | 2.1 | 1.5 | 100 | 59.8 | 2.4 | 1.7 | 1.4 | 1.568 |
| Q14204 | Cytoplasmic dynein 1 heavy chain 1 OS=Homo sapiens GN=DYNC1H1 PE=1 SV=5 | 23 | 5.3 | 1.2 | 2.2 | 1.4 | 17 | 3.7 | 1.3 | 1.1 | 1.4 | 21 | 5.9 | 1.6 | 1.6 | 1.5 | 32 | 5.9 | 2.4 | 1.5 | 1.4 | 1.568 |
| P05204 | Non-histone chromosomal protein HMG-17 OS=Homo sapiens GN=HMGN2 PE=1 SV=3 | * | * | * | * | * | 6 | 16.7 | 1.1 | 0.9 | 1.8 | 3 | 24.4 | 3.1 | 2.1 | 0.9 | 2 | 45.6 | 1.7 | 1.0 | 1.3 | 1.567 |
| P45880 | Voltage-dependent anion-selective channel protein 2 OS=Homo sapiens GN=VDAC2 PE=1 SV=2 | 11 | 12.2 | 1.2 | 1.5 | 1.0 | * | * | * | * | * | 40 | 20.1 | 1.9 | 2.6 | 1.2 | 22 | 18.7 | 1.9 | 1.4 | 1.0 | 1.566 |
| P58546 | Myotrophin OS=Homo sapiens GN=MTPN PE=1 SV=2 | 5 | 14.4 | 0.9 | 1.1 | 1.2 | 3 | 14.4 | 1.5 | 1.3 | 4.2 | 3 | 14.4 | 1.2 | 1.3 | 0.9 | 6 | 14.4 | 2.3 | 1.4 | 1.6 | 1.565 |
| P05787 | Keratin, type II cytoskeletal 8 OS=Homo sapiens GN=KRT8 PE=1 SV=7 | 394 | 78.9 | 0.9 | 2.4 | 1.4 | 176 | 66.0 | 0.5 | 0.4 | 1.2 | 344 | 74.1 | 2.8 | 3.0 | 1.4 | 284 | 70 | 2.0 | 1.2 | 1.5 | 1.563 |
| Q9Y2V2 | Calcium-regulated heat-stable protein 1 OS=Homo sapiens GN=CARHSP1 PE=1 SV=2 | 5 | 10.9 | 1.5 | 1.0 | 1.0 | 8 | 10.9 | 1.3 | 1.3 | 0.6 | 17 | 10.9 | 2.1 | 4.3 | 1.7 | 4 | 10.9 | 0.6 | 2.0 | 1.3 | 1.562 |
| P08865 | 40S ribosomal protein SA OS=Homo sapiens GN=RPSA PE=1 SV=4 | 32 | 29.5 | 1.0 | 2.5 | 0.8 | 31 | 33.6 | 1.6 | 0.6 | 0.8 | 55 | 29.2 | 2.2 | 3.1 | 1.8 | 22 | 29.2 | 1.0 | 1.8 | 1.1 | 1.561 |
| Q15149 | Plectin OS=Homo sapiens GN=PLEC PE=1 SV=3 | 12 | 5.6 | 1.1 | 1.9 | 0.9 | 5 | 1.3 | 1.3 | 1.8 | 2.0 | 18 | 3.5 | 1.4 | 1.9 | 1.2 | 38 | 7.8 | 2.4 | 1.5 | 1.3 | 1.558 |
| P49748 | Very long-chain specific acyl-CoA dehydrogenase, mitochondrial OS=Homo sapiens GN=ACADVL PE=1 SV=1 | 18 | 21.8 | 1.1 | 1.8 | 1.1 | 8 | 11.1 | 0.8 | 0.6 | 1.1 | 12 | 20.8 | 2.5 | 2.9 | 2.2 | 14 | 14.5 | 1.5 | 1.5 | 1.5 | 1.557 |
| P31146 | Coronin-1A OS=Homo sapiens GN=CORO1A PE=1 SV=4 | 7 | 5.9 | 1.5 | 1.4 | 1.1 | 8 | 8.0 | 1.1 | 0.7 | 2.1 | 7 | 9.8 | 1.3 | 1.6 | 1.4 | 11 | 11.9 | 3.0 | 2.0 | 1.9 | 1.557 |
| P21399 | Cytoplasmic aconitate hydratase OS=Homo sapiens GN=ACO1 PE=1 SV=3 | 3 | 6.0 | 1.4 | 3.0 | 0.9 | 3 | 5.7 | 1.1 | 1.6 | 0.6 | 9 | 6 | 1.7 | 1.9 | 1.5 | 12 | 15.1 | 1.3 | 2.1 | 1.3 | 1.555 |
| P62942 | Peptidyl-prolyl cis-trans isomerase FKBP1A OS=Homo sapiens GN=FKBP1A PE=1 SV=2 | 15 | 25.0 | 1.2 | 1.3 | 1.3 | 32 | 25.0 | 1.1 | 0.9 | 1.7 | 8 | 25 | 1.5 | 2.8 | 1.5 | 22 | 56.5 | 2.3 | 1.5 | 1.4 | 1.553 |
| P62857 | 40S ribosomal protein S28 OS=Homo sapiens GN=RPS28 PE=1 SV=1 | 10 | 30.4 | 1.3 | 2.3 | 1.4 | 5 | 30.4 | 1.5 | 1.5 | 1.7 | 3 | 30.4 | 0.9 | 1.9 | 1.4 | 2 | 13 | 1.2 | 1.9 | 1.7 | 1.551 |
| P47756 | F-actin-capping protein subunit beta OS=Homo sapiens GN=CAPZB PE=1 SV=4 | 15 | 20.6 | 0.9 | 1.4 | 1.0 | 15 | 8.7 | 0.8 | 1.0 | 1.5 | 15 | 8.3 | 1.9 | 3.2 | 1.9 | 21 | 20.6 | 1.7 | 1.8 | 1.5 | 1.548 |
| P51149 | Ras-related protein Rab-7a OS=Homo sapiens GN=RAB7A PE=1 SV=1 | 8 | 29.5 | 1.8 | 2.3 | 0.7 | 6 | 13.0 | 1.3 | 1.1 | 1.3 | 7 | 23.2 | 1.8 | 2.8 | 1.6 | 13 | 25.1 | 0.9 | 1.3 | 0.9 | 1.546 |
| Q9Y4L1 | Hypoxia up-regulated protein 1 OS=Homo sapiens GN=HYOU1 PE=1 SV=1 | 7 | 10.4 | 1.5 | 2.3 | 1.1 | 5 | 3.6 | 1.4 | 1.2 | 1.1 | 16 | 13 | 2.1 | 2.0 | 1.0 | 18 | 13.7 | 2.2 | 1.2 | 1.0 | 1.544 |
| P0CG47 | Polyubiquitin-B OS=Homo sapiens GN=UBB PE=1 SV=1;Polyubiquitin-C OS=Homo sapiens GN=UBC PE=1 SV=3;Ubiquitin-40S ribosomal protein S27a OS=Homo sapiens GN=RPS27A PE=1 SV=2;Ubiquitin-60S ribosomal protein L40 OS=Homo sapiens GN=UBA52 PE=1 SV=2;sp RS27A_HUMAN | 50 | 61.8 | 1.1 | 1.8 | 1.1 | 63 | 61.8 | 1.6 | 0.9 | 1.6 | 59 | 44.7 | 1.9 | 2.4 | 1.4 | 59 | 61.8 | 1.9 | 1.5 | 1.4 | 1.543 |
| P05455 | Lupus La protein OS=Homo sapiens GN=SSB PE=1 SV=2 | 14 | 10.0 | 0.9 | 2.2 | 0.7 | 5 | 9.6 | 2.0 | 1.1 | 1.8 | 14 | 10.3 | 1.7 | 1.5 | 1.5 | 20 | 16.2 | 1.7 | 1.7 | 1.9 | 1.542 |
| P05198 | Eukaryotic translation initiation factor 2 subunit 1 OS=Homo sapiens GN=EIF2S1 PE=1 SV=3 | 4 | 8.6 | 1.5 | 2.1 | 0.8 | 7 | 8.6 | 2.3 | 1.5 | 1.4 | 3 | 4.8 | 2.3 | 1.4 | 1.2 | 4 | 8.6 | 1.8 | 0.6 | 1.1 | 1.540 |
| P68032 | Actin, alpha cardiac muscle 1 OS=Homo sapiens GN=ACTC1 PE=1 SV=1 | 5 | 80.6 | 0.6 | 0.8 | 0.8 | 3 | 79.0 | 2.2 | 2.3 | 3.6 | * | * | * | * | * | 4 | 77.5 | 1.0 | 1.0 | 0.7 | 1.539 |
| Q9Y265 | RuvB-like 1 OS=Homo sapiens GN=RUVBL1 PE=1 SV=1 | 5 | 9.0 | 1.2 | 2.3 | 0.7 | 3 | 9.0 | 0.2 | 0.8 | 0.7 | 15 | 13.8 | 2.8 | 4.1 | 1.5 | 5 | 8.3 | 1.4 | 1.2 | 1.0 | 1.538 |
| Q13263 | Transcription intermediary factor 1-beta OS=Homo sapiens GN=TRIM28 PE=1 SV=5 | * | * | * | * | * | 5 | 2.8 | 1.4 | 0.9 | 1.7 | 2 | 5.3 | 1.1 | 2.3 | 1.8 | 8 | 9.6 | 1.4 | 1.7 | 1.0 | 1.538 |
| Q00610 | Clathrin heavy chain 1 OS=Homo sapiens GN=CLTC PE=1 SV=5 | 98 | 20.0 | 1.2 | 1.7 | 1.1 | 33 | 11.8 | 0.9 | 1.0 | 1.3 | 83 | 20.1 | 2.0 | 2.9 | 1.8 | 83 | 19.3 | 1.6 | 1.5 | 1.3 | 1.538 |
| P16401 | Histone H1.5 OS=Homo sapiens GN=HIST1H1B PE=1 SV=3 | 24 | 18.6 | 1.0 | 1.1 | 1.4 | 41 | 29.6 | 0.7 | 0.7 | 1.9 | 29 | 21.2 | 3.4 | 1.9 | 1.5 | 23 | 18.6 | 1.8 | 1.6 | 1.4 | 1.537 |
| P35222 | Catenin beta-1 OS=Homo sapiens GN=CTNNB1 PE=1 SV=1 | 13 | 14.9 | 0.8 | 1.4 | 1.0 | * | * | * | * | * | 8 | 14.1 | 2.5 | 2.7 | 1.9 | 10 | 8.5 | 1.0 | 0.9 | 1.3 | 1.535 |
| Q16401 | 26S proteasome non-ATPase regulatory subunit 5 OS=Homo sapiens GN=PSMD5 PE=1 SV=3;26S proteasome non-ATPase regulatory subunit 5 OS=Macaca fascicularis GN=PSMD5 PE=2 SV=3 | * | * | * | * | * | 4 | 5.6 | 1.3 | 1.0 | 2.4 | 3 | 3 | 2.2 | 1.4 | 0.9 | 4 | 5.6 | 2.0 | 1.1 | 1.4 | 1.534 |
| P12236 | ADP/ATP translocase 3 OS=Homo sapiens GN=SLC25A6 PE=1 SV=4 | 25 | 31.5 | 0.9 | 2.2 | 0.5 | 14 | 24.8 | 2.4 | 0.9 | 0.9 | 37 | 31.9 | 2.5 | 2.7 | 1.7 | 13 | 38.9 | 0.7 | 1.6 | 1.2 | 1.534 |
| P07195 | L-lactate dehydrogenase B chain OS=Homo sapiens GN=LDHB PE=1 SV=2 | 53 | 36.5 | 0.8 | 2.4 | 0.6 | 64 | 33.2 | 1.5 | 0.9 | 2.1 | 53 | 27.8 | 1.6 | 2.8 | 1.8 | 40 | 41.3 | 1.1 | 1.4 | 1.3 | 1.532 |
| P06733 | Alpha-enolase OS=Homo sapiens GN=ENO1 PE=1 SV=2 | 269 | 61.3 | 1.4 | 1.7 | 0.8 | 287 | 62.7 | 1.7 | 1.0 | 1.2 | 318 | 55.8 | 1.8 | 2.5 | 1.5 | 280 | 62.2 | 1.3 | 1.9 | 1.4 | 1.531 |
| P07741 | Adenine phosphoribosyltransferase OS=Homo sapiens GN=APRT PE=1 SV=2 | 14 | 37.2 | 1.2 | 2.1 | 1.1 | * | * | * | * | * | 41 | 37.2 | 2.0 | 2.5 | 1.4 | 19 | 37.2 | 0.8 | 1.1 | 1.1 | 1.531 |
| Q14847 | LIM and SH3 domain protein 1 OS=Homo sapiens GN=LASP1 PE=1 SV=2 | 20 | 34.9 | 1.5 | 1.8 | 1.1 | 7 | 13.4 | 1.0 | 1.0 | 1.4 | 15 | 26.8 | 1.6 | 1.9 | 1.6 | 24 | 29.1 | 2.5 | 1.4 | 1.4 | 1.531 |
| P00390 | Glutathione reductase, mitochondrial OS=Homo sapiens GN=GSR PE=1 SV=2 | * | * | * | * | * | 2 | 2.3 | 1.4 | 0.5 | 1.7 | 5 | 2.3 | 2.2 | 2.8 | 1.2 | 2 | 4.2 | 1.0 | 1.5 | 0.3 | 1.531 |
| P68366 | Tubulin alpha-4A chain OS=Homo sapiens GN=TUBA4A PE=1 SV=1 | 11 | 43.8 | 1.0 | 1.0 | 1.0 | * | * | * | * | * | 6 | 42.9 | 2.4 | 2.4 | 1.5 | 37 | 41.7 | 1.4 | 1.4 | 1.2 | 1.528 |
| Q8NBS9 | Thioredoxin domain-containing protein 5 OS=Homo sapiens GN=TXNDC5 PE=1 SV=2 | 54 | 42.8 | 0.9 | 1.9 | 1.4 | 37 | 34.0 | 1.7 | 1.2 | 1.2 | 35 | 29.9 | 2.3 | 2.2 | 1.4 | 33 | 27.3 | 1.6 | 1.0 | 1.3 | 1.528 |
| P52272 | Heterogeneous nuclear ribonucleoprotein M OS=Homo sapiens GN=HNRNPM PE=1 SV=3 | 41 | 20.7 | 0.7 | 1.9 | 1.0 | 21 | 22.3 | 1.2 | 0.8 | 1.6 | 79 | 36.8 | 1.8 | 2.5 | 1.8 | 41 | 25.9 | 1.9 | 1.8 | 1.7 | 1.527 |
| P31942 | Heterogeneous nuclear ribonucleoprotein H3 OS=Homo sapiens GN=HNRNPH3 PE=1 SV=2 | 6 | 11.8 | 1.1 | 2.4 | 1.1 | 6 | 2.9 | 0.8 | 0.8 | 1.0 | 11 | 11.3 | 2.6 | 2.9 | 1.8 | 10 | 7.8 | 0.8 | 1.5 | 1.6 | 1.526 |
| P06744 | Glucose-6-phosphate isomerase OS=Homo sapiens GN=GPI PE=1 SV=4 | 180 | 41.9 | 0.9 | 1.7 | 1.0 | 119 | 41.9 | 1.7 | 0.9 | 1.3 | 168 | 35.8 | 1.3 | 2.8 | 1.6 | 257 | 44.8 | 1.9 | 1.6 | 1.3 | 1.525 |
| Q02818 | Nucleobindin-1 OS=Homo sapiens GN=NUCB1 PE=1 SV=4 | 3 | 7.8 | 1.2 | 1.3 | 1.1 | * | * | * | * | * | 4 | 13.7 | 1.7 | 2.1 | 2.4 | 4 | 11.1 | 1.4 | 1.1 | 0.9 | 1.525 |
| P22314 | Ubiquitin-like modifier-activating enzyme 1 OS=Homo sapiens GN=UBA1 PE=1 SV=3 | 126 | 28.4 | 1.1 | 2.1 | 0.9 | 66 | 26.8 | 1.3 | 0.9 | 1.0 | 132 | 34.4 | 2.3 | 2.7 | 1.7 | 97 | 28.6 | 1.2 | 1.7 | 1.2 | 1.525 |
| P51659 | Peroxisomal multifunctional enzyme type 2 OS=Homo sapiens GN=HSD17B4 PE=1 SV=3 | 11 | 16.0 | 1.4 | 2.1 | 0.7 | 7 | 11.5 | 1.4 | 0.7 | 1.9 | 19 | 16.7 | 1.8 | 2.4 | 1.3 | 24 | 17.4 | 1.4 | 2.0 | 1.6 | 1.524 |
| P63104 | 14-3-3 protein zeta/delta OS=Homo sapiens GN=YWHAZ PE=1 SV=1 | 134 | 60.0 | 0.9 | 2.5 | 1.0 | 126 | 60.8 | 1.4 | 0.7 | 1.6 | 83 | 64.1 | 1.5 | 2.1 | 1.5 | 93 | 60.8 | 1.9 | 1.6 | 1.5 | 1.522 |
| P61978 | Heterogeneous nuclear ribonucleoprotein K OS=Homo sapiens GN=HNRNPK PE=1 SV=1 | 145 | 39.7 | 0.9 | 1.8 | 0.9 | 90 | 40.6 | 1.4 | 1.0 | 1.6 | 173 | 38.4 | 1.8 | 2.7 | 1.4 | 152 | 38.4 | 1.7 | 1.5 | 1.3 | 1.522 |
| P07384 | Calpain-1 catalytic subunit OS=Homo sapiens GN=CAPN1 PE=1 SV=1 | 16 | 8.7 | 1.2 | 1.1 | 0.9 | 7 | 6.0 | 1.4 | 0.7 | 1.5 | 18 | 6.6 | 2.0 | 2.7 | 1.8 | 16 | 8 | 2.0 | 1.4 | 1.1 | 1.522 |
| Q9P2E9 | Ribosome-binding protein 1 OS=Homo sapiens GN=RRBP1 PE=1 SV=4 | 26 | 13.2 | 1.2 | 1.6 | 1.1 | 27 | 31.0 | 1.0 | 0.9 | 1.4 | 28 | 16.5 | 1.9 | 2.2 | 1.3 | 41 | 28.9 | 2.7 | 1.5 | 1.5 | 1.520 |
| O14602 | Eukaryotic translation initiation factor 1A, Y-chromosomal OS=Homo sapiens GN=EIF1AY PE=1 SV=4;Eukaryotic translation initiation factor 1A, X-chromosomal OS=Homo sapiens GN=EIF1AX PE=1 SV=2 | 5 | 37.5 | 0.9 | 2.4 | 1.3 | * | * | * | * | * | 4 | 16.7 | 1.4 | 1.2 | 1.9 | 3 | 16 | 1.6 | 1.4 | 1.8 | 1.519 |
| P52565 | Rho GDP-dissociation inhibitor 1 OS=Homo sapiens GN=ARHGDIA PE=1 SV=3 | 8 | 22.1 | 0.7 | 2.0 | 0.8 | 11 | 15.2 | 1.0 | 0.9 | 1.2 | 9 | 25.5 | 1.5 | 3.3 | 1.6 | 7 | 22.1 | 2.0 | 1.8 | 1.2 | 1.517 |
| P29692 | Elongation factor 1-delta OS=Homo sapiens GN=EEF1D PE=1 SV=5 | 72 | 28.8 | 1.2 | 2.3 | 0.8 | 39 | 28.8 | 1.5 | 0.7 | 1.4 | 76 | 32 | 1.9 | 2.4 | 1.6 | 62 | 26.3 | 1.6 | 1.2 | 1.3 | 1.517 |
| O14773 | Tripeptidyl-peptidase 1 OS=Homo sapiens GN=TPP1 PE=1 SV=2;Tripeptidyl-peptidase 1 OS=Pan troglodytes GN=TPP1 PE=2 SV=1 | 8 | 11.7 | 1.8 | 1.3 | 0.7 | 20 | 11.7 | 1.5 | 1.1 | 0.9 | 24 | 11.7 | 2.1 | 2.1 | 1.6 | 21 | 15.1 | 1.4 | 2.1 | 1.7 | 1.516 |
| P01593 | Ig kappa chain V-I region AG OS=Homo sapiens PE=1 SV=1 | 10 | 31.5 | 2.1 | 1.2 | 1.4 | 25 | 38.0 | 1.6 | 1.2 | 1.6 | 6 | 16.7 | 0.7 | 2.3 | 1.8 | 15 | 31.5 | 1.3 | 1.5 | 0.9 | 1.515 |
| O15127 | Secretory carrier-associated membrane protein 2 OS=Homo sapiens GN=SCAMP2 PE=1 SV=2 | 4 | 13.7 | 0.8 | 1.3 | 0.8 | 2 | 9.7 | 1.0 | 0.5 | 1.0 | 5 | 13.7 | 2.3 | 3.3 | 1.3 | 4 | 9.7 | 2.5 | 2.0 | 2.0 | 1.514 |
| P55769 | NHP2-like protein 1 OS=Homo sapiens GN=SNU13 PE=1 SV=3 | 3 | 18.0 | 0.7 | 2.1 | 1.0 | * | * | * | * | * | 8 | 18 | 1.8 | 1.7 | 1.3 | 6 | 18 | 1.5 | 2.1 | 1.9 | 1.512 |
| P11021 | 78 kDa glucose-regulated protein OS=Homo sapiens GN=HSPA5 PE=1 SV=2 | 373 | 54.1 | 1.2 | 1.9 | 1.1 | 279 | 51.1 | 1.2 | 0.9 | 1.1 | 395 | 52.1 | 2.0 | 2.7 | 1.3 | 316 | 54.7 | 1.6 | 1.7 | 1.4 | 1.510 |
| P28066 | Proteasome subunit alpha type-5 OS=Homo sapiens GN=PSMA5 PE=1 SV=3 | 31 | 22.4 | 1.1 | 1.8 | 1.0 | 16 | 19.9 | 1.1 | 0.7 | 1.3 | 42 | 22.4 | 2.1 | 2.2 | 1.5 | 29 | 22.4 | 2.1 | 1.7 | 1.4 | 1.510 |
| Q16698 | 2,4-dienoyl-CoA reductase, mitochondrial OS=Homo sapiens GN=DECR1 PE=1 SV=1 | 9 | 17.3 | 0.8 | 1.7 | 0.6 | 2 | 13.4 | 1.2 | 0.7 | 1.0 | 21 | 17.3 | 3.2 | 2.9 | 2.3 | 5 | 14.3 | 1.0 | 1.1 | 1.2 | 1.508 |
| O75874 | Isocitrate dehydrogenase [NADP] cytoplasmic OS=Homo sapiens GN=IDH1 PE=1 SV=2 | 73 | 50.5 | 1.1 | 1.6 | 1.1 | 52 | 40.3 | 1.4 | 1.2 | 1.3 | 71 | 43 | 2.3 | 2.9 | 1.4 | 61 | 45.4 | 0.8 | 1.6 | 1.3 | 1.505 |
| P31040 | Succinate dehydrogenase [ubiquinone] flavoprotein subunit, mitochondrial OS=Homo sapiens GN=SDHA PE=1 SV=2 | 4 | 2.1 | 1.9 | 0.9 | 1.3 | * | * | * | * | * | 3 | 2.1 | 2.0 | 2.4 | 0.9 | 24 | 6.2 | 1.1 | 1.5 | 1.3 | 1.501 |
| P00505 | Aspartate aminotransferase, mitochondrial OS=Homo sapiens GN=GOT2 PE=1 SV=3 | 37 | 26.5 | 0.9 | 1.7 | 1.0 | 23 | 17.2 | 1.6 | 0.9 | 1.0 | 78 | 22.8 | 2.3 | 3.1 | 1.8 | 28 | 24.7 | 0.9 | 1.4 | 1.1 | 1.497 |
| Q9HC38 | Glyoxalase domain-containing protein 4 OS=Homo sapiens GN=GLOD4 PE=1 SV=1 | 22 | 20.4 | 1.0 | 1.7 | 1.3 | 13 | 13.1 | 1.8 | 1.3 | 1.4 | 15 | 7.7 | 1.7 | 2.3 | 1.3 | 22 | 13.1 | 1.4 | 1.4 | 1.3 | 1.496 |
| P67809 | Nuclease-sensitive element-binding protein 1 OS=Homo sapiens GN=YBX1 PE=1 SV=3 | 5 | 26.5 | 1.2 | 1.8 | 1.5 | 5 | 17.3 | 1.1 | 0.3 | 0.8 | 18 | 20.4 | 2.4 | 2.3 | 1.4 | 6 | 26.9 | 1.9 | 1.7 | 2.2 | 1.496 |
| P68402 | Platelet-activating factor acetylhydrolase IB subunit beta OS=Homo sapiens GN=PAFAH1B2 PE=1 SV=1 | 5 | 12.2 | 1.3 | 2.1 | 1.1 | 5 | 3.9 | 1.1 | 1.0 | 1.4 | 5 | 3.9 | 1.7 | 2.3 | 1.1 | 5 | 16.6 | 1.8 | 1.7 | 1.7 | 1.496 |
| Q01082 | Spectrin beta chain, non-erythrocytic 1 OS=Homo sapiens GN=SPTBN1 PE=1 SV=2 | 44 | 17.5 | 1.0 | 1.7 | 1.1 | 14 | 7.6 | 1.2 | 0.7 | 1.5 | 28 | 8.4 | 1.8 | 2.6 | 1.6 | 53 | 15.2 | 1.9 | 1.4 | 1.2 | 1.494 |
| P13611 | Versican core protein OS=Homo sapiens GN=VCAN PE=1 SV=3 | 15 | 2.3 | 2.4 | 1.1 | 0.6 | 5 | 1.1 | 1.5 | 2.7 | 0.7 | * | * | * | * | * | 5 | 0.9 | 1.2 | 1.7 | 2.3 | 1.488 |
| O43399 | Tumor protein D54 OS=Homo sapiens GN=TPD52L2 PE=1 SV=2 | 3 | 20.9 | 0.9 | 0.7 | 0.3 | 2 | 6.8 | 0.8 | 0.8 | 0.3 | 7 | 11.7 | 3.7 | 4.0 | 1.7 | 5 | 32 | 1.5 | 1.6 | 1.8 | 1.487 |
| O60664 | Perilipin-3 OS=Homo sapiens GN=PLIN3 PE=1 SV=3 | 19 | 30.2 | 1.0 | 1.9 | 0.7 | 6 | 10.4 | 2.6 | 1.9 | 1.2 | 28 | 20.7 | 1.4 | 2.1 | 1.1 | 21 | 27.2 | 0.9 | 1.5 | 1.1 | 1.486 |
| P06899 | Histone H2B type 1-J OS=Homo sapiens GN=HIST1H2BJ PE=1 SV=3;Histone H2B type 1-O OS=Homo sapiens GN=HIST1H2BO PE=1 SV=3;Histone H2B type 1-B OS=Homo sapiens GN=HIST1H2BB PE=1 SV=2;Histone H2B type 2-E OS=Homo sapiens GN=HIST2H2BE PE=1 SV=3 | 2 | 47.6 | 1.2 | 0.7 | 0.7 | 6 | 48.4 | 1.7 | 0.8 | 3.0 | * | * | * | * | * | 6 | 38.1 | 2.3 | 1.5 | 1.5 | 1.485 |
| Q9UIJ7 | GTP:AMP phosphotransferase AK3, mitochondrial OS=Homo sapiens GN=AK3 PE=1 SV=4 | 14 | 37.0 | 1.0 | 1.6 | 0.9 | 6 | 13.7 | 0.9 | 0.7 | 1.4 | 19 | 26 | 2.9 | 2.6 | 2.0 | 6 | 13.2 | 1.2 | 1.1 | 1.4 | 1.485 |
| P20618 | Proteasome subunit beta type-1 OS=Homo sapiens GN=PSMB1 PE=1 SV=2 | 23 | 29.5 | 1.1 | 1.2 | 1.0 | 10 | 29.5 | 1.8 | 0.9 | 1.9 | 15 | 31.5 | 1.8 | 1.6 | 1.6 | 31 | 44 | 1.9 | 1.5 | 1.2 | 1.483 |
| P09874 | Poly [ADP-ribose] polymerase 1 OS=Homo sapiens GN=PARP1 PE=1 SV=4 | 8 | 5.1 | 1.2 | 1.7 | 0.6 | 3 | 3.7 | 1.4 | 0.9 | 1.4 | 10 | 10.8 | 2.2 | 2.8 | 1.8 | 11 | 6.3 | 1.2 | 1.1 | 1.2 | 1.481 |
| O14561 | Acyl carrier protein, mitochondrial OS=Homo sapiens GN=NDUFAB1 PE=1 SV=3 | 5 | 9.0 | 1.3 | 1.6 | 1.0 | 5 | 9.0 | 0.9 | 0.5 | 1.5 | 4 | 9 | 1.5 | 1.2 | 1.3 | 9 | 9 | 4.7 | 0.9 | 2.3 | 1.479 |
| P23381 | Tryptophan--tRNA ligase, cytoplasmic OS=Homo sapiens GN=WARS PE=1 SV=2 | 27 | 15.9 | 1.2 | 1.6 | 0.7 | 23 | 20.0 | 1.4 | 0.8 | 1.5 | 30 | 12.7 | 1.8 | 1.7 | 1.1 | 80 | 30.6 | 2.6 | 1.8 | 1.5 | 1.478 |
| Q9UJZ1 | Stomatin-like protein 2, mitochondrial OS=Homo sapiens GN=STOML2 PE=1 SV=1 | 7 | 13.8 | 0.8 | 2.3 | 0.6 | * | * | * | * | * | 2 | 21.9 | 1.0 | 4.0 | 1.2 | 5 | 12.9 | 0.4 | 1.5 | 1.0 | 1.478 |
| P04207 | Ig kappa chain V-III region CLL OS=Homo sapiens PE=4 SV=2 | 5 | 14.0 | 0.7 | 0.8 | 0.9 | 9 | 26.4 | 1.8 | 0.7 | 1.1 | * | * | * | * | * | 6 | 14 | 2.8 | 3.1 | 1.5 | 1.478 |
| P17980 | 26S protease regulatory subunit 6A OS=Homo sapiens GN=PSMC3 PE=1 SV=3 | 3 | 10.3 | 0.6 | 0.9 | 0.5 | * | * | * | * | * | 6 | 7.1 | 2.4 | 2.8 | 2.1 | 9 | 13.2 | 1.1 | 1.4 | 1.1 | 1.477 |
| P25325 | 3-mercaptopyruvate sulfurtransferase OS=Homo sapiens GN=MPST PE=1 SV=3 | 4 | 13.5 | 0.3 | 1.3 | 0.4 | * | * | * | * | * | 26 | 30.6 | 2.5 | 3.0 | 1.8 | 8 | 9.8 | 1.0 | 1.4 | 1.6 | 1.476 |
| Q13011 | Delta(3,5)-Delta(2,4)-dienoyl-CoA isomerase, mitochondrial OS=Homo sapiens GN=ECH1 PE=1 SV=2 | 23 | 39.9 | 1.2 | 1.4 | 1.3 | 16 | 18.0 | 1.5 | 0.8 | 1.4 | 20 | 32 | 1.8 | 2.7 | 2.2 | 13 | 27.7 | 1.3 | 0.9 | 1.3 | 1.473 |
| O75369 | Filamin-B OS=Homo sapiens GN=FLNB PE=1 SV=2 | 101 | 20.5 | 1.0 | 1.3 | 1.0 | 37 | 13.3 | 1.1 | 0.8 | 1.4 | 65 | 13.8 | 1.9 | 2.5 | 1.5 | 104 | 21.6 | 1.9 | 1.7 | 1.3 | 1.470 |
| Q99536 | Synaptic vesicle membrane protein VAT-1 homolog OS=Homo sapiens GN=VAT1 PE=1 SV=2 | 5 | 12.2 | 1.0 | 1.7 | 1.2 | 4 | 12.2 | 0.9 | 0.8 | 0.9 | 4 | 8.1 | 2.0 | 1.8 | 2.1 | 13 | 21.6 | 1.9 | 2.0 | 1.3 | 1.469 |
| P30040 | Endoplasmic reticulum resident protein 29 OS=Homo sapiens GN=ERP29 PE=1 SV=4 | 26 | 25.7 | 0.9 | 1.7 | 0.7 | 20 | 19.9 | 1.7 | 1.0 | 0.9 | 40 | 19.9 | 2.0 | 3.1 | 2.0 | 26 | 35.2 | 0.7 | 1.6 | 1.0 | 1.468 |
| P12532 | Creatine kinase U-type, mitochondrial OS=Homo sapiens GN=CKMT1A PE=1 SV=1 | 42 | 28.1 | 0.7 | 1.7 | 1.4 | 13 | 12.5 | 1.4 | 0.7 | 1.5 | 44 | 28.5 | 1.6 | 4.3 | 0.9 | 22 | 12.5 | 1.2 | 0.8 | 1.3 | 1.468 |
| O43707 | Alpha-actinin-4 OS=Homo sapiens GN=ACTN4 PE=1 SV=2 | 83 | 48.0 | 1.2 | 1.4 | 1.4 | 52 | 40.6 | 0.9 | 0.7 | 1.4 | 42 | 38.1 | 2.5 | 2.2 | 1.4 | 67 | 49 | 1.8 | 1.4 | 1.2 | 1.468 |
| P05023 | Sodium/potassium-transporting ATPase subunit alpha-1 OS=Homo sapiens GN=ATP1A1 PE=1 SV=1 | 87 | 21.5 | 0.9 | 1.7 | 1.2 | 65 | 17.6 | 1.0 | 0.7 | 1.3 | 121 | 24.9 | 1.8 | 2.8 | 1.5 | 61 | 19.3 | 1.5 | 1.6 | 1.4 | 1.465 |
| P01602 | Ig heavy chain V-I region 5 OS=Homo sapiens GN=IGKV1-5 PE=1 SV=2 | 4 | 24.8 | 2.1 | 1.4 | 1.8 | 11 | 24.8 | 1.2 | 0.6 | 0.8 | * | * | * | * | * | 10 | 24.8 | 2.1 | 1.7 | 1.3 | 1.463 |
| O43242 | 26S proteasome non-ATPase regulatory subunit 3 OS=Homo sapiens GN=PSMD3 PE=1 SV=2 | 7 | 5.2 | 1.0 | 1.7 | 1.1 | 2 | 2.4 | 0.2 | 1.1 | 1.0 | 8 | 5.2 | 1.7 | 3.5 | 1.5 | 12 | 5.2 | 1.5 | 1.9 | 1.0 | 1.463 |
| P05026 | Sodium/potassium-transporting ATPase subunit beta-1 OS=Homo sapiens GN=ATP1B1 PE=1 SV=1 | 28 | 16.5 | 0.9 | 1.7 | 1.6 | 24 | 16.5 | 1.1 | 0.3 | 1.3 | 32 | 16.5 | 2.1 | 2.6 | 1.2 | 40 | 20.1 | 1.5 | 1.7 | 1.2 | 1.463 |
| O00571 | ATP-dependent RNA helicase DDX3X OS=Homo sapiens GN=DDX3X PE=1 SV=3 | 16 | 11.5 | 0.6 | 2.0 | 0.7 | * | * | * | * | * | 14 | 13.3 | 1.3 | 2.3 | 1.3 | 11 | 9.4 | 1.5 | 2.1 | 2.1 | 1.461 |
| Q6P587 | Acylpyruvase FAHD1, mitochondrial OS=Homo sapiens GN=FAHD1 PE=1 SV=2 | 3 | 13.8 | 2.8 | 2.1 | 1.3 | * | * | * | * | * | 4 | 17 | 1.5 | 1.1 | 0.7 | 4 | 13.8 | 1.1 | 1.1 | 2.3 | 1.461 |
| P32969 | 60S ribosomal protein L9 OS=Homo sapiens GN=RPL9 PE=1 SV=1;60S ribosomal protein L9 OS=Macaca fascicularis GN=RPL9 PE=2 SV=1 | 11 | 15.6 | 0.8 | 2.0 | 0.7 | * | * | * | * | * | 5 | 13 | 1.5 | 1.7 | 1.4 | 3 | 5.7 | 1.6 | 1.9 | 1.2 | 1.461 |
| P08397 | Porphobilinogen deaminase OS=Homo sapiens GN=HMBS PE=1 SV=2 | 3 | 5.5 | 0.5 | 1.8 | 0.8 | * | * | * | * | * | 2 | 5.5 | 1.4 | 1.7 | 0.6 | 5 | 5.5 | 3.7 | 1.1 | 1.2 | 1.461 |
| P09622 | Dihydrolipoyl dehydrogenase, mitochondrial OS=Homo sapiens GN=DLD PE=1 SV=2 | 19 | 10.6 | 0.8 | 1.1 | 0.8 | 10 | 5.7 | 2.1 | 0.9 | 1.8 | 33 | 19.1 | 2.0 | 3.0 | 1.8 | 17 | 7.9 | 1.1 | 0.8 | 0.8 | 1.461 |
| P54136 | Arginine--tRNA ligase, cytoplasmic OS=Homo sapiens GN=RARS PE=1 SV=2 | 7 | 6.8 | 0.8 | 1.1 | 0.9 | 4 | 3.6 | 1.2 | 0.8 | 0.8 | 10 | 7 | 2.3 | 3.0 | 1.6 | 12 | 10 | 2.1 | 1.5 | 2.2 | 1.452 |
| Q02790 | Peptidyl-prolyl cis-trans isomerase FKBP4 OS=Homo sapiens GN=FKBP4 PE=1 SV=3 | 11 | 23.1 | 1.0 | 1.9 | 0.8 | * | * | * | * | * | 4 | 12.6 | 1.2 | 2.2 | 0.9 | 7 | 10.9 | 2.2 | 1.4 | 1.7 | 1.452 |
| P17655 | Calpain-2 catalytic subunit OS=Homo sapiens GN=CAPN2 PE=1 SV=6 | 2 | 5.9 | 0.9 | 0.4 | 0.2 | * | * | * | * | * | 7 | 2.1 | 1.7 | 1.9 | 1.4 | 2 | 3.9 | 3.7 | 1.4 | 3.3 | 1.452 |
| P31937 | 3-hydroxyisobutyrate dehydrogenase, mitochondrial OS=Homo sapiens GN=HIBADH PE=1 SV=2 | 7 | 28.9 | 0.6 | 1.6 | 0.6 | 5 | 24.7 | 1.5 | 0.4 | 1.3 | 32 | 21.7 | 2.9 | 2.2 | 2.0 | 13 | 23.5 | 1.5 | 1.3 | 1.0 | 1.452 |
| O15511 | Actin-related protein 2/3 complex subunit 5 OS=Homo sapiens GN=ARPC5 PE=1 SV=3 | 5 | 29.1 | 1.5 | 1.3 | 0.9 | 4 | 7.9 | 1.1 | 0.9 | 0.9 | 12 | 16.6 | 2.0 | 2.8 | 1.6 | * | * | * | * | * | 1.449 |
| Q14240 | Eukaryotic initiation factor 4A-II OS=Homo sapiens GN=EIF4A2 PE=1 SV=2 | 4 | 13.5 | 0.7 | 1.4 | 1.2 | 4 | 13.5 | 1.2 | 0.8 | 0.5 | 5 | 18.4 | 3.2 | 2.1 | 2.6 | 6 | 16.5 | 1.1 | 1.1 | 1.0 | 1.449 |
| Q9BSJ8 | Extended synaptotagmin-1 OS=Homo sapiens GN=ESYT1 PE=1 SV=1 | 5 | 2.6 | 1.8 | 1.1 | 1.2 | 5 | 1.2 | 1.6 | 0.9 | 0.8 | 12 | 3.4 | 2.3 | 2.4 | 1.0 | 4 | 5.8 | 1.5 | 1.3 | 0.8 | 1.446 |
| P38117 | Electron transfer flavoprotein subunit beta OS=Homo sapiens GN=ETFB PE=1 SV=3 | 9 | 31.8 | 0.8 | 1.5 | 0.7 | 7 | 22.4 | 2.1 | 0.9 | 1.9 | 17 | 21.2 | 1.9 | 2.3 | 1.0 | 3 | 8.6 | 1.2 | 1.4 | 1.4 | 1.443 |
| P78371 | T-complex protein 1 subunit beta OS=Homo sapiens GN=CCT2 PE=1 SV=4 | 25 | 20.7 | 0.7 | 2.0 | 0.6 | 12 | 18.7 | 1.5 | 1.2 | 1.0 | 45 | 37.6 | 1.8 | 2.8 | 1.2 | 25 | 23.6 | 1.4 | 1.8 | 1.8 | 1.440 |
| P62820 | Ras-related protein Rab-1A OS=Homo sapiens GN=RAB1A PE=1 SV=3 | 8 | 37.1 | 0.7 | 1.3 | 0.7 | 4 | 19.0 | 2.0 | 1.3 | 2.1 | 7 | 35.1 | 1.9 | 2.2 | 1.1 | 8 | 46.3 | 1.2 | 1.5 | 0.9 | 1.438 |
| P49368 | T-complex protein 1 subunit gamma OS=Homo sapiens GN=CCT3 PE=1 SV=4 | 31 | 14.7 | 0.9 | 2.1 | 0.7 | 21 | 13.2 | 1.5 | 0.8 | 0.9 | 55 | 16.5 | 2.2 | 2.4 | 1.5 | 26 | 16.5 | 1.4 | 1.4 | 1.2 | 1.432 |
| Q9NVA2 | Septin-11 OS=Homo sapiens GN=SEPT11 PE=1 SV=3 | 4 | 8.9 | 1.7 | 2.0 | 1.7 | 2 | 6.5 | 2.0 | 1.4 | 1.1 | 3 | 8.6 | 1.0 | 1.4 | 0.6 | * | * | * | * | * | 1.432 |
| P25398 | 40S ribosomal protein S12 OS=Homo sapiens GN=RPS12 PE=1 SV=3 | 7 | 7.6 | 1.2 | 2.0 | 0.6 | 5 | 13.6 | 1.0 | 0.8 | 0.9 | 8 | 13.6 | 1.9 | 2.3 | 1.3 | 4 | 13.6 | 1.6 | 2.1 | 1.9 | 1.428 |
| P49720 | Proteasome subunit beta type-3 OS=Homo sapiens GN=PSMB3 PE=1 SV=2 | 17 | 28.8 | 0.9 | 1.7 | 0.8 | 9 | 32.2 | 1.6 | 0.7 | 1.4 | 20 | 25.4 | 1.6 | 2.0 | 1.2 | 46 | 36.1 | 2.1 | 1.9 | 1.4 | 1.426 |
| P07355 | Annexin A2 OS=Homo sapiens GN=ANXA2 PE=1 SV=2 | 302 | 67.6 | 1.3 | 1.9 | 0.9 | 272 | 64.9 | 1.0 | 1.0 | 1.0 | 281 | 59.6 | 2.0 | 2.4 | 1.5 | 285 | 74.6 | 1.3 | 1.4 | 1.3 | 1.426 |
| P13804 | Electron transfer flavoprotein subunit alpha, mitochondrial OS=Homo sapiens GN=ETFA PE=1 SV=1 | 30 | 39.9 | 0.9 | 1.9 | 0.7 | 11 | 31.8 | 1.6 | 1.2 | 0.8 | 41 | 34.8 | 2.3 | 2.5 | 1.5 | 26 | 30.9 | 0.8 | 1.5 | 1.4 | 1.424 |
| P14550 | Alcohol dehydrogenase [NADP(+)] OS=Homo sapiens GN=AKR1A1 PE=1 SV=3 | 18 | 20.6 | 0.9 | 1.4 | 0.9 | 14 | 28.6 | 1.0 | 1.1 | 1.0 | 22 | 20.6 | 2.3 | 2.7 | 1.6 | 15 | 21.2 | 0.9 | 1.9 | 1.1 | 1.422 |
| P14174 | Macrophage migration inhibitory factor OS=Homo sapiens GN=MIF PE=1 SV=4 | 14 | 40.0 | 1.5 | 2.0 | 1.8 | 12 | 26.1 | 0.8 | 0.5 | 0.8 | 19 | 40 | 1.4 | 2.5 | 0.9 | 28 | 40 | 2.0 | 1.4 | 1.7 | 1.421 |
| O43488 | Aflatoxin B1 aldehyde reductase member 2 OS=Homo sapiens GN=AKR7A2 PE=1 SV=3 | 9 | 12.8 | 1.2 | 2.9 | 1.1 | 10 | 12.8 | 0.9 | 0.9 | 1.6 | 24 | 15.9 | 1.8 | 1.4 | 0.8 | 8 | 12.8 | 1.6 | 1.3 | 1.1 | 1.420 |
| P52907 | F-actin-capping protein subunit alpha-1 OS=Homo sapiens GN=CAPZA1 PE=1 SV=3 | 6 | 20.3 | 0.7 | 1.5 | 0.5 | 3 | 10.5 | 2.2 | 0.5 | 1.6 | 14 | 15 | 1.5 | 2.1 | 1.5 | 6 | 20.3 | 1.8 | 1.8 | 1.1 | 1.417 |
| P09211 | sp GSTP1_HUMAN ;Glutathione S-transferase P OS=Homo sapiens GN=GSTP1 PE=1 SV=2 | 61 | 52.6 | 1.2 | 0.8 | 0.9 | 87 | 65.1 | 1.2 | 1.0 | 1.2 | 104 | 60.3 | 2.9 | 1.7 | 1.4 | 74 | 64.1 | 1.9 | 1.4 | 1.6 | 1.416 |
| P04075 | Fructose-bisphosphate aldolase A OS=Homo sapiens GN=ALDOA PE=1 SV=2 | 266 | 60.4 | 0.9 | 1.3 | 0.8 | 349 | 54.7 | 1.3 | 0.8 | 1.4 | 368 | 62.1 | 1.4 | 2.6 | 1.4 | 345 | 56.3 | 2.4 | 1.4 | 1.3 | 1.415 |
| P11413 | Glucose-6-phosphate 1-dehydrogenase OS=Homo sapiens GN=G6PD PE=1 SV=4 | 9 | 7.8 | 1.0 | 2.2 | 0.7 | * | * | * | * | * | 5 | 3.1 | 1.7 | 1.5 | 1.3 | 25 | 11.7 | 1.2 | 1.7 | 1.2 | 1.414 |
| Q15056 | Eukaryotic translation initiation factor 4H OS=Homo sapiens GN=EIF4H PE=1 SV=5 | 4 | 16.9 | 1.7 | 1.0 | 1.9 | * | * | * | * | * | 5 | 19.8 | 1.5 | 1.4 | 1.4 | 2 | 15.3 | 1.1 | 1.3 | 2.0 | 1.413 |
| Q15942 | Zyxin OS=Homo sapiens GN=ZYX PE=1 SV=1 | * | * | * | * | * | 6 | 14.5 | 0.7 | 1.0 | 1.6 | 8 | 14.7 | 1.9 | 1.7 | 2.1 | 7 | 7.7 | 1.2 | 1.1 | 0.8 | 1.413 |
| P48735 | Isocitrate dehydrogenase [NADP], mitochondrial OS=Homo sapiens GN=IDH2 PE=1 SV=2 | 39 | 29.6 | 0.5 | 1.5 | 0.9 | 30 | 32.5 | 0.9 | 0.7 | 0.9 | 56 | 36.1 | 3.6 | 1.9 | 1.9 | 46 | 32.5 | 1.2 | 1.4 | 1.4 | 1.410 |
| P02545 | Prelamin-A/C OS=Homo sapiens GN=LMNA PE=1 SV=1 | 174 | 54.1 | 1.2 | 1.3 | 1.3 | 150 | 54.8 | 1.0 | 1.0 | 1.6 | 109 | 46.8 | 1.5 | 2.2 | 1.2 | 151 | 50.6 | 2.2 | 1.2 | 1.1 | 1.409 |
| P29966 | Myristoylated alanine-rich C-kinase substrate OS=Homo sapiens GN=MARCKS PE=1 SV=4 | 8 | 30.7 | 1.0 | 0.8 | 0.9 | 9 | 28.6 | 1.2 | 1.2 | 1.2 | * | * | * | * | * | 6 | 23.2 | 3.3 | 1.7 | 1.8 | 1.405 |
| Q12864 | Cadherin-17 OS=Homo sapiens GN=CDH17 PE=2 SV=3 | 60 | 22.1 | 0.8 | 1.7 | 0.7 | 8 | 12.9 | 1.4 | 0.5 | 1.0 | 67 | 20.8 | 2.4 | 3.5 | 1.5 | 53 | 22.2 | 0.8 | 1.3 | 1.2 | 1.402 |
| Q01518 | Adenylyl cyclase-associated protein 1 OS=Homo sapiens GN=CAP1 PE=1 SV=5 | 65 | 27.6 | 1.4 | 1.7 | 0.6 | 48 | 37.7 | 1.4 | 1.2 | 1.1 | 62 | 33.1 | 1.4 | 2.0 | 2.0 | 73 | 32.4 | 0.8 | 1.7 | 1.4 | 1.400 |
| Q9NUQ9 | Protein FAM49B OS=Homo sapiens GN=FAM49B PE=1 SV=1 | 3 | 6.2 | 1.1 | 1.8 | 1.0 | 6 | 7.4 | 1.5 | 1.3 | 1.1 | 5 | 7.4 | 1.3 | 1.8 | 1.8 | 6 | 13.3 | 1.7 | 1.3 | 1.3 | 1.397 |
| P35268 | 60S ribosomal protein L22 OS=Homo sapiens GN=RPL22 PE=1 SV=2 | 33 | 30.5 | 1.2 | 2.3 | 1.0 | 13 | 18.8 | 1.2 | 0.8 | 1.0 | 34 | 18.8 | 2.0 | 2.0 | 1.5 | 15 | 25.8 | 1.2 | 1.2 | 1.4 | 1.397 |
| Q14764 | Major vault protein OS=Homo sapiens GN=MVP PE=1 SV=4 | 24 | 26.2 | 1.0 | 1.2 | 0.8 | 13 | 11.8 | 1.4 | 0.9 | 1.1 | 38 | 27.4 | 1.8 | 3.2 | 1.4 | 30 | 27.5 | 1.3 | 1.3 | 1.2 | 1.396 |
| P32455 | Guanylate-binding protein 1 OS=Homo sapiens GN=GBP1 PE=1 SV=2;Guanylate-binding protein 2 OS=Homo sapiens GN=GBP2 PE=1 SV=3 | 3 | 2.7 | 0.6 | 1.5 | 0.7 | * | * | * | * | * | 3 | 5.9 | 1.1 | 1.8 | 2.0 | 9 | 6.6 | 1.6 | 1.9 | 1.2 | 1.395 |
| Q96QK1 | Vacuolar protein sorting-associated protein 35 OS=Homo sapiens GN=VPS35 PE=1 SV=2 | * | * | * | * | * | 3 | 5.7 | 1.3 | 1.0 | 1.3 | 7 | 8.5 | 1.3 | 1.6 | 0.9 | 11 | 12.7 | 1.8 | 1.9 | 2.1 | 1.393 |
| Q12907 | Vesicular integral-membrane protein VIP36 OS=Homo sapiens GN=LMAN2 PE=1 SV=1 | 5 | 18.3 | 0.9 | 0.7 | 1.0 | * | * | * | * | * | 17 | 25.6 | 2.2 | 2.8 | 1.2 | 2 | 5.6 | 1.0 | 1.3 | 0.8 | 1.392 |
| P05091 | Aldehyde dehydrogenase, mitochondrial OS=Homo sapiens GN=ALDH2 PE=1 SV=2 | 68 | 34.0 | 0.9 | 1.6 | 0.8 | 32 | 21.3 | 1.3 | 1.0 | 1.0 | 112 | 39.7 | 2.0 | 3.0 | 1.5 | 56 | 27.1 | 0.7 | 1.5 | 1.2 | 1.392 |
| Q9NX40 | OCIA domain-containing protein 1 OS=Homo sapiens GN=OCIAD1 PE=1 SV=1 | 6 | 7.3 | 0.7 | 1.6 | 1.0 | 3 | 7.3 | 1.3 | 1.9 | 2.3 | 11 | 7.3 | 1.7 | 2.2 | 1.1 | 2 | 7.3 | 0.9 | 0.6 | 1.1 | 1.391 |
| P14923 | Junction plakoglobin OS=Homo sapiens GN=JUP PE=1 SV=3 | 3 | 10.2 | 1.7 | 0.9 | 0.8 | * | * | * | * | * | 11 | 12.1 | 1.9 | 1.6 | 1.2 | 7 | 6.2 | 1.1 | 1.9 | 1.1 | 1.391 |
| P00403 | Cytochrome c oxidase subunit 2 OS=Homo sapiens GN=MT-CO2 PE=1 SV=1 | 37 | 21.6 | 0.8 | 1.4 | 0.8 | 34 | 16.3 | 0.6 | 0.5 | 0.6 | 48 | 13.2 | 2.7 | 3.7 | 2.0 | 37 | 13.2 | 0.7 | 1.5 | 1.0 | 1.391 |
| P48047 | ATP synthase subunit O, mitochondrial OS=Homo sapiens GN=ATP5O PE=1 SV=1 | 13 | 30.0 | 1.4 | 2.1 | 1.1 | 10 | 30.5 | 1.2 | 0.7 | 0.7 | 36 | 48.4 | 2.2 | 2.9 | 1.7 | 6 | 32.4 | 0.7 | 0.7 | 0.7 | 1.391 |
| Q96HC4 | PDZ and LIM domain protein 5 OS=Homo sapiens GN=PDLIM5 PE=1 SV=5 | * | * | * | * | * | 3 | 3.5 | 1.2 | 0.9 | 1.4 | 5 | 6.4 | 1.1 | 1.8 | 1.3 | 8 | 5.4 | 0.9 | 2.5 | 1.3 | 1.389 |
| P30050 | 60S ribosomal protein L12 OS=Homo sapiens GN=RPL12 PE=1 SV=1 | 23 | 29.1 | 0.7 | 1.5 | 0.6 | 16 | 29.7 | 1.4 | 0.9 | 1.6 | 43 | 24.2 | 1.9 | 2.1 | 1.5 | 24 | 24.2 | 1.8 | 1.4 | 1.7 | 1.389 |
| P07954 | Fumarate hydratase, mitochondrial OS=Homo sapiens GN=FH PE=1 SV=3 | 21 | 21.8 | 0.8 | 1.3 | 0.7 | 25 | 22.7 | 1.1 | 0.7 | 1.0 | 37 | 19.6 | 1.8 | 3.3 | 1.5 | 30 | 25.7 | 1.7 | 1.5 | 1.4 | 1.387 |
| Q96IU4 | Protein ABHD14B OS=Homo sapiens GN=ABHD14B PE=1 SV=1 | 9 | 28.6 | 0.5 | 1.3 | 0.6 | * | * | * | * | * | 6 | 23.3 | 2.2 | 2.5 | 1.9 | 3 | 11.4 | 0.9 | 1.2 | 0.9 | 1.387 |
| Q14974 | Importin subunit beta-1 OS=Homo sapiens GN=KPNB1 PE=1 SV=2 | 16 | 12.4 | 1.3 | 2.4 | 1.3 | 4 | 9.9 | 1.9 | 1.5 | 1.1 | 12 | 9.5 | 0.7 | 0.9 | 0.7 | 13 | 7.9 | 1.4 | 2.1 | 1.8 | 1.387 |
| P01857 | Ig gamma-1 chain C region OS=Homo sapiens GN=IGHG1 PE=1 SV=1 | 210 | 48.2 | 2.6 | 0.7 | 0.6 | 365 | 50.6 | 4.0 | 0.6 | 1.0 | 149 | 44.5 | 1.1 | 1.5 | 1.3 | 148 | 48.2 | 0.7 | 1.1 | 1.3 | 1.386 |
| Q15717 | ELAV-like protein 1 OS=Homo sapiens GN=ELAVL1 PE=1 SV=2 | 10 | 7.4 | 0.6 | 1.5 | 0.9 | * | * | * | * | * | 22 | 20.2 | 1.8 | 1.8 | 1.7 | 8 | 19.3 | 1.8 | 1.0 | 1.8 | 1.386 |
| Q96KP4 | Cytosolic non-specific dipeptidase OS=Homo sapiens GN=CNDP2 PE=1 SV=2 | 23 | 32.0 | 0.9 | 1.5 | 0.4 | * | * | * | * | * | 18 | 23.2 | 1.5 | 2.3 | 1.1 | 33 | 26.5 | 1.7 | 1.7 | 1.5 | 1.385 |
| P04406 | Glyceraldehyde-3-phosphate dehydrogenase OS=Homo sapiens GN=GAPDH PE=1 SV=3 | 461 | 73.4 | 1.4 | 1.8 | 0.5 | 380 | 59.7 | 1.6 | 1.1 | 0.7 | 630 | 72.8 | 1.5 | 3.0 | 1.4 | 579 | 77 | 0.8 | 1.4 | 1.2 | 1.383 |
| P61163 | Alpha-centractin OS=Homo sapiens GN=ACTR1A PE=1 SV=1 | 4 | 9.3 | 1.7 | 2.8 | 1.4 | 4 | 9.3 | 1.4 | 0.5 | 1.0 | 10 | 11.4 | 0.8 | 1.7 | 1.0 | 9 | 18.6 | 1.3 | 1.6 | 0.8 | 1.383 |
| P35900 | Keratin, type I cytoskeletal 20 OS=Homo sapiens GN=KRT20 PE=1 SV=1 | 51 | 48.3 | 0.6 | 1.7 | 0.8 | * | * | * | * | * | 15 | 39.2 | 1.8 | 2.5 | 1.5 | 18 | 23.1 | 1.3 | 0.9 | 0.8 | 1.379 |
| Q9NR45 | Sialic acid synthase OS=Homo sapiens GN=NANS PE=1 SV=2 | 10 | 24.2 | 1.0 | 1.6 | 0.5 | 3 | 4.7 | 1.6 | 0.7 | 1.0 | 19 | 21.4 | 1.8 | 3.3 | 1.6 | 9 | 19.2 | 0.9 | 1.1 | 1.0 | 1.377 |
| Q8WUM4 | Programmed cell death 6-interacting protein OS=Homo sapiens GN=PDCD6IP PE=1 SV=1 | 7 | 6.8 | 0.9 | 2.2 | 1.1 | * | * | * | * | * | 7 | 8.6 | 1.5 | 2.2 | 0.9 | 4 | 4.4 | 1.2 | 1.0 | 1.6 | 1.377 |
| Q15366 | Poly(rC)-binding protein 2 OS=Homo sapiens GN=PCBP2 PE=1 SV=1 | 9 | 23.0 | 1.3 | 2.2 | 1.5 | 5 | 14.2 | 0.7 | 0.5 | 0.7 | 24 | 20 | 2.0 | 2.7 | 1.4 | 3 | 11.2 | 1.0 | 1.2 | 0.8 | 1.374 |
| O14950 | Myosin regulatory light chain 12B OS=Homo sapiens GN=MYL12B PE=1 SV=2;Myosin regulatory light chain 12A OS=Homo sapiens GN=MYL12A PE=1 SV=2 | 10 | 48.5 | 1.2 | 2.1 | 0.6 | 9 | 62.0 | 1.0 | 0.8 | 0.5 | 10 | 55 | 1.8 | 2.4 | 2.2 | 8 | 56.7 | 1.4 | 1.2 | 1.3 | 1.373 |
| P00367 | Glutamate dehydrogenase 1, mitochondrial OS=Homo sapiens GN=GLUD1 PE=1 SV=2 | 15 | 19.5 | 0.9 | 1.4 | 1.0 | 5 | 8.6 | 1.0 | 0.7 | 1.4 | 28 | 24.9 | 2.0 | 2.5 | 1.6 | 19 | 22 | 1.1 | 1.4 | 1.0 | 1.372 |
| P00568 | Adenylate kinase isoenzyme 1 OS=Homo sapiens GN=AK1 PE=1 SV=3 | * | * | * | * | * | 2 | 19.1 | 1.6 | 1.3 | 2.3 | 11 | 20.1 | 1.4 | 2.0 | 1.1 | 9 | 19.1 | 0.4 | 0.8 | 0.6 | 1.371 |
| P49411 | Elongation factor Tu, mitochondrial OS=Homo sapiens GN=TUFM PE=1 SV=2 | 37 | 38.3 | 0.5 | 1.9 | 0.8 | 41 | 37.2 | 1.2 | 0.9 | 1.1 | 98 | 41.4 | 1.9 | 2.5 | 1.5 | 51 | 38.1 | 1.4 | 1.2 | 1.4 | 1.369 |
| P31153 | S-adenosylmethionine synthase isoform type-2 OS=Homo sapiens GN=MAT2A PE=1 SV=1 | 5 | 7.6 | 0.5 | 0.9 | 0.6 | * | * | * | * | * | 10 | 6.6 | 2.6 | 3.7 | 1.2 | 4 | 10.9 | 0.6 | 0.8 | 0.6 | 1.368 |
| P54819 | Adenylate kinase 2, mitochondrial OS=Homo sapiens GN=AK2 PE=1 SV=2 | 32 | 47.3 | 0.9 | 1.3 | 1.2 | 28 | 31.8 | 1.4 | 0.6 | 1.1 | 51 | 38.9 | 1.4 | 2.1 | 1.3 | 61 | 38.5 | 1.9 | 1.9 | 1.6 | 1.366 |
| P61981 | 14-3-3 protein gamma OS=Homo sapiens GN=YWHAG PE=1 SV=2 | 21 | 46.6 | 0.6 | 1.6 | 0.8 | 9 | 41.3 | 1.2 | 1.2 | 1.4 | 7 | 40.9 | 1.2 | 2.0 | 1.5 | 17 | 40.1 | 2.1 | 1.6 | 1.3 | 1.366 |
| Q99714 | 3-hydroxyacyl-CoA dehydrogenase type-2 OS=Homo sapiens GN=HSD17B10 PE=1 SV=3 | 28 | 29.1 | 1.1 | 1.6 | 0.9 | 9 | 12.3 | 1.1 | 0.8 | 1.8 | 56 | 51.3 | 1.7 | 2.2 | 1.5 | 26 | 28 | 1.2 | 1.2 | 1.3 | 1.364 |
| O60888 | Protein CutA OS=Homo sapiens GN=CUTA PE=1 SV=2 | 4 | 30.7 | 2.5 | 1.4 | 1.3 | * | * | * | * | * | 6 | 7.8 | 1.4 | 1.6 | 1.4 | 5 | 22.9 | 0.8 | 0.5 | 0.6 | 1.362 |
| Q13200 | 26S proteasome non-ATPase regulatory subunit 2 OS=Homo sapiens GN=PSMD2 PE=1 SV=3 | 6 | 7.6 | 1.5 | 1.0 | 0.8 | 5 | 1.7 | 0.8 | 0.6 | 0.7 | 11 | 9.6 | 1.9 | 2.8 | 1.7 | 6 | 5.8 | 1.4 | 1.7 | 0.7 | 1.360 |
| Q86VP6 | Cullin-associated NEDD8-dissociated protein 1 OS=Homo sapiens GN=CAND1 PE=1 SV=2 | 12 | 12.7 | 1.1 | 1.6 | 0.6 | 9 | 9.3 | 1.1 | 1.3 | 0.9 | 34 | 10.5 | 1.7 | 2.1 | 1.6 | 31 | 12.8 | 1.4 | 1.7 | 1.2 | 1.359 |
| O15173 | Membrane-associated progesterone receptor component 2 OS=Homo sapiens GN=PGRMC2 PE=1 SV=1 | 5 | 6.3 | 1.3 | 1.6 | 1.5 | 5 | 6.3 | 0.8 | 0.5 | 0.9 | 6 | 6.3 | 2.3 | 1.6 | 1.4 | 4 | 6.3 | 1.6 | 1.6 | 1.2 | 1.358 |
| Q14624 | Inter-alpha-trypsin inhibitor heavy chain H4 OS=Homo sapiens GN=ITIH4 PE=1 SV=4 | 3 | 9.5 | 1.9 | 1.4 | 1.2 | 3 | 9.0 | 1.2 | 0.9 | 2.2 | * | * | * | * | * | 5 | 5.4 | 1.0 | 1.0 | 1.2 | 1.356 |
| Q00325 | Phosphate carrier protein, mitochondrial OS=Homo sapiens GN=SLC25A3 PE=1 SV=2 | 3 | 7.7 | 1.4 | 1.0 | 0.3 | 11 | 3.3 | 1.1 | 1.3 | 1.6 | 7 | 5.5 | 2.1 | 2.3 | 0.8 | 3 | 7.5 | 1.2 | 1.7 | 0.9 | 1.355 |
| Q15691 | Microtubule-associated protein RP/EB family member 1 OS=Homo sapiens GN=MAPRE1 PE=1 SV=3 | 5 | 11.2 | 1.2 | 2.1 | 0.3 | 3 | 7.5 | 1.6 | 0.8 | 0.8 | 12 | 25.7 | 1.9 | 2.0 | 1.4 | 11 | 20.1 | 1.5 | 1.3 | 1.3 | 1.354 |
| Q13813 | Spectrin alpha chain, non-erythrocytic 1 OS=Homo sapiens GN=SPTAN1 PE=1 SV=3 | 95 | 22.3 | 0.8 | 1.6 | 1.1 | 37 | 11.5 | 1.2 | 0.8 | 1.6 | 65 | 13.5 | 1.4 | 2.1 | 1.2 | 121 | 20.4 | 1.9 | 1.2 | 1.1 | 1.354 |
| P50395 | Rab GDP dissociation inhibitor beta OS=Homo sapiens GN=GDI2 PE=1 SV=2 | 103 | 40.2 | 1.2 | 1.8 | 0.8 | 51 | 31.7 | 1.2 | 0.9 | 0.7 | 109 | 37.1 | 1.8 | 2.5 | 1.5 | 47 | 39.6 | 0.9 | 1.6 | 1.3 | 1.351 |
| P49257 | Protein ERGIC-53 OS=Homo sapiens GN=LMAN1 PE=1 SV=2 | 4 | 12.5 | 1.7 | 1.8 | 1.1 | * | * | * | * | * | 2 | 6.9 | 1.1 | 1.2 | 0.5 | 8 | 18.8 | 1.7 | 1.8 | 1.6 | 1.349 |
| P08603 | Complement factor H OS=Homo sapiens GN=CFH PE=1 SV=4 | 19 | 8.4 | 1.6 | 1.4 | 0.9 | 14 | 6.0 | 1.3 | 0.9 | 1.9 | 8 | 2.6 | 0.8 | 1.8 | 1.2 | 9 | 3.2 | 1.7 | 1.2 | 1.4 | 1.346 |
| P30837 | Aldehyde dehydrogenase X, mitochondrial OS=Homo sapiens GN=ALDH1B1 PE=1 SV=3 | 25 | 27.7 | 1.0 | 1.6 | 0.8 | 32 | 29.0 | 1.8 | 1.0 | 0.8 | 108 | 25.7 | 1.8 | 2.6 | 1.5 | 28 | 24.4 | 0.5 | 1.3 | 1.1 | 1.345 |
| P40939 | Trifunctional enzyme subunit alpha, mitochondrial OS=Homo sapiens GN=HADHA PE=1 SV=2 | 66 | 28.0 | 0.9 | 1.6 | 0.6 | 38 | 17.2 | 1.3 | 1.0 | 1.3 | 120 | 26.5 | 2.1 | 2.2 | 1.6 | 66 | 28.4 | 0.9 | 1.4 | 1.0 | 1.345 |
| P68371 | Tubulin beta-4B chain OS=Homo sapiens GN=TUBB4B PE=1 SV=1 | 25 | 54.8 | 0.7 | 2.0 | 0.5 | 17 | 56.9 | 1.8 | 1.0 | 0.9 | 34 | 52.6 | 1.2 | 3.2 | 1.2 | 12 | 61.1 | 0.8 | 1.5 | 1.3 | 1.343 |
| Q16891 | MICOS complex subunit MIC60 OS=Homo sapiens GN=IMMT PE=1 SV=1 | 2 | 8.2 | 1.2 | 1.9 | 0.9 | 3 | 8.6 | 1.2 | 0.4 | 1.0 | 11 | 14.5 | 2.4 | 1.8 | 1.1 | 12 | 11.3 | 1.5 | 1.2 | 1.1 | 1.341 |
| P04844 | Dolichyl-diphosphooligosaccharide--protein glycosyltransferase subunit 2 OS=Homo sapiens GN=RPN2 PE=1 SV=3 | 24 | 25.4 | 0.8 | 1.4 | 0.5 | 5 | 7.9 | 0.7 | 0.9 | 0.8 | 27 | 20.6 | 2.3 | 2.7 | 1.8 | 18 | 27.1 | 1.1 | 1.9 | 1.6 | 1.338 |
| O75347 | Tubulin-specific chaperone A OS=Homo sapiens GN=TBCA PE=1 SV=3 | 12 | 16.7 | 0.7 | 1.1 | 0.8 | 7 | 10.2 | 1.0 | 1.1 | 1.1 | 12 | 10.2 | 1.9 | 2.0 | 1.5 | 8 | 10.2 | 1.7 | 1.7 | 0.9 | 1.337 |
| P51148 | Ras-related protein Rab-5C OS=Homo sapiens GN=RAB5C PE=1 SV=2 | 4 | 18.5 | 1.2 | 2.2 | 0.4 | 2 | 18.5 | 1.4 | 1.3 | 1.2 | 18 | 17.6 | 1.5 | 2.1 | 0.9 | 9 | 17.1 | 1.5 | 1.1 | 1.6 | 1.331 |
| P31930 | Cytochrome b-c1 complex subunit 1, mitochondrial OS=Homo sapiens GN=UQCRC1 PE=1 SV=3 | 30 | 19.0 | 1.0 | 1.9 | 0.8 | 14 | 10.8 | 1.0 | 0.6 | 1.0 | 43 | 16.9 | 1.7 | 2.4 | 1.5 | 21 | 21.9 | 1.3 | 1.4 | 1.3 | 1.331 |
| Q99497 | Protein deglycase DJ-1 OS=Homo sapiens GN=PARK7 PE=1 SV=2 | 35 | 38.6 | 1.0 | 1.6 | 0.5 | 31 | 33.3 | 0.8 | 0.9 | 0.8 | 28 | 33.3 | 2.2 | 2.6 | 1.4 | 35 | 51.9 | 1.7 | 1.1 | 1.2 | 1.328 |
| O60716 | Catenin delta-1 OS=Homo sapiens GN=CTNND1 PE=1 SV=1 | 22 | 11.2 | 0.6 | 1.9 | 0.6 | 8 | 4.2 | 1.3 | 1.5 | 0.6 | 17 | 5.5 | 2.2 | 2.4 | 1.3 | 14 | 4.2 | 0.9 | 1.4 | 1.1 | 1.326 |
| P10606 | Cytochrome c oxidase subunit 5B, mitochondrial OS=Homo sapiens GN=COX5B PE=1 SV=2 | 30 | 39.5 | 1.0 | 1.3 | 1.1 | 28 | 38.8 | 1.1 | 0.8 | 1.8 | 28 | 39.5 | 1.5 | 2.3 | 1.5 | 45 | 29.5 | 1.3 | 1.2 | 1.2 | 1.326 |
| P45974 | Ubiquitin carboxyl-terminal hydrolase 5 OS=Homo sapiens GN=USP5 PE=1 SV=2 | 7 | 8.3 | 1.0 | 2.7 | 1.0 | * | * | * | * | * | 3 | 5.5 | 2.2 | 1.1 | 1.2 | 3 | 8.9 | 0.4 | 1.0 | 0.7 | 1.324 |
| Q07065 | Cytoskeleton-associated protein 4 OS=Homo sapiens GN=CKAP4 PE=1 SV=2 | 58 | 30.2 | 1.3 | 1.5 | 0.9 | 31 | 23.3 | 1.3 | 0.9 | 1.3 | 48 | 22.6 | 1.3 | 1.8 | 1.3 | 62 | 33.9 | 1.2 | 1.8 | 1.2 | 1.324 |
| Q6YN16 | Hydroxysteroid dehydrogenase-like protein 2 OS=Homo sapiens GN=HSDL2 PE=1 SV=1 | 8 | 12.9 | 0.7 | 1.6 | 0.5 | * | * | * | * | * | 3 | 4.1 | 1.7 | 3.1 | 1.1 | 2 | 7.7 | 0.8 | 1.0 | 1.3 | 1.323 |
| P20073 | Annexin A7 OS=Homo sapiens GN=ANXA7 PE=1 SV=3 | 20 | 10.7 | 0.7 | 1.1 | 0.5 | 6 | 4.9 | 1.5 | 0.8 | 1.8 | 20 | 4.9 | 1.1 | 1.8 | 1.3 | 24 | 13.7 | 2.2 | 1.7 | 1.3 | 1.323 |
| Q13283 | Ras GTPase-activating protein-binding protein 1 OS=Homo sapiens GN=G3BP1 PE=1 SV=1 | * | * | * | * | * | 2 | 10.3 | 2.0 | 0.5 | 1.8 | 9 | 14.6 | 1.2 | 1.8 | 0.7 | 7 | 10.9 | 1.5 | 1.1 | 0.9 | 1.320 |
| P36542 | ATP synthase subunit gamma, mitochondrial OS=Homo sapiens GN=ATP5C1 PE=1 SV=1 | 12 | 15.4 | 0.6 | 2.1 | 0.7 | 12 | 11.1 | 1.1 | 0.9 | 1.6 | 36 | 22.1 | 1.8 | 2.6 | 1.2 | 11 | 14.8 | 0.6 | 1.2 | 0.8 | 1.320 |
| P30153 | Serine/threonine-protein phosphatase 2A 65 kDa regulatory subunit A alpha isoform OS=Homo sapiens GN=PPP2R1A PE=1 SV=4 | 13 | 20.7 | 0.8 | 1.2 | 0.7 | 7 | 11.7 | 1.3 | 0.8 | 0.9 | 13 | 16.1 | 2.4 | 3.1 | 1.1 | 18 | 16.5 | 1.1 | 1.0 | 1.0 | 1.319 |
| O75531 | Barrier-to-autointegration factor OS=Homo sapiens GN=BANF1 PE=1 SV=1 | 4 | 13.5 | 1.1 | 0.8 | 1.4 | 2 | 40.4 | 1.0 | 1.0 | 1.5 | * | * | * | * | * | 6 | 44.9 | 2.4 | 1.4 | 1.5 | 1.319 |
| P56385 | ATP synthase subunit e, mitochondrial OS=Homo sapiens GN=ATP5I PE=1 SV=2 | 4 | 34.8 | 0.9 | 1.5 | 1.5 | 2 | 14.5 | 0.7 | 0.5 | 1.2 | 10 | 31.9 | 2.1 | 2.2 | 0.9 | 4 | 34.8 | 1.7 | 1.3 | 0.7 | 1.317 |
| P26373 | 60S ribosomal protein L13 OS=Homo sapiens GN=RPL13 PE=1 SV=4 | 10 | 24.6 | 1.0 | 1.6 | 0.7 | 8 | 16.1 | 1.7 | 1.3 | 0.7 | 26 | 24.2 | 1.3 | 2.7 | 1.0 | 9 | 17.5 | 1.6 | 0.8 | 2.0 | 1.316 |
| P33176 | Kinesin-1 heavy chain OS=Homo sapiens GN=KIF5B PE=1 SV=1 | 7 | 7.6 | 1.3 | 1.6 | 1.8 | * | * | * | * | * | 5 | 5.8 | 1.4 | 1.9 | 0.6 | 11 | 8.2 | 1.1 | 0.9 | 1.1 | 1.315 |
| P05155 | Plasma protease C1 inhibitor OS=Homo sapiens GN=SERPING1 PE=1 SV=2 | 24 | 18.2 | 1.3 | 1.0 | 0.8 | 14 | 7.8 | 1.5 | 1.7 | 2.1 | 6 | 5.4 | 1.2 | 1.3 | 1.4 | 9 | 7.8 | 1.3 | 0.9 | 1.0 | 1.313 |
| P35908 | Keratin, type II cytoskeletal 2 epidermal OS=Homo sapiens GN=KRT2 PE=1 SV=2 | 2 | 38.8 | 1.3 | 1.4 | 2.6 | 34 | 44.3 | 0.9 | 0.6 | 1.4 | 14 | 35.5 | 0.9 | 1.4 | 1.3 | * | * | * | * | * | 1.309 |
| Q01844 | RNA-binding protein EWS OS=Homo sapiens GN=EWSR1 PE=1 SV=1 | 3 | 2.1 | 0.3 | 0.9 | 0.9 | 4 | 2.1 | 1.6 | 0.7 | 1.3 | 3 | 4.9 | 2.0 | 2.1 | 1.9 | * | * | * | * | * | 1.309 |
| P08727 | Keratin, type I cytoskeletal 19 OS=Homo sapiens GN=KRT19 PE=1 SV=4 | 161 | 74.0 | 0.8 | 1.6 | 1.2 | 49 | 50.2 | 0.6 | 0.5 | 1.4 | 146 | 67.8 | 2.1 | 2.1 | 1.1 | 166 | 60.8 | 1.8 | 1.1 | 1.2 | 1.309 |
| P02751 | Fibronectin OS=Homo sapiens GN=FN1 PE=1 SV=4 | 54 | 14.2 | 2.1 | 0.9 | 0.8 | 41 | 9.8 | 1.4 | 0.9 | 1.7 | 7 | 2.4 | 1.3 | 1.4 | 1.3 | 33 | 11.4 | 1.2 | 1.6 | 1.2 | 1.305 |
| Q9H4A4 | Aminopeptidase B OS=Homo sapiens GN=RNPEP PE=1 SV=2 | 3 | 8.9 | 0.4 | 1.0 | 1.0 | 2 | 3.8 | 0.4 | 1.3 | 0.7 | 12 | 13.2 | 1.8 | 3.5 | 1.8 | 8 | 8.9 | 1.1 | 1.3 | 1.7 | 1.302 |
| P30085 | UMP-CMP kinase OS=Homo sapiens GN=CMPK1 PE=1 SV=3 | 42 | 48.0 | 0.8 | 2.3 | 0.9 | 8 | 23.0 | 0.8 | 0.6 | 0.5 | 58 | 50.5 | 1.8 | 2.8 | 1.8 | 33 | 42.9 | 0.6 | 1.4 | 1.0 | 1.301 |
| P04899 | Guanine nucleotide-binding protein G(i) subunit alpha-2 OS=Homo sapiens GN=GNAI2 PE=1 SV=3 | 4 | 11.8 | 0.5 | 0.8 | 0.9 | 6 | 7.6 | 1.2 | 0.8 | 1.2 | 8 | 15.2 | 1.1 | 2.6 | 1.4 | 9 | 15.8 | 1.6 | 2.2 | 2.2 | 1.301 |
| O95865 | N(G),N(G)-dimethylarginine dimethylaminohydrolase 2 OS=Homo sapiens GN=DDAH2 PE=1 SV=1 | 5 | 37.2 | 1.6 | 1.1 | 1.3 | * | * | * | * | * | 5 | 12.6 | 0.9 | 1.4 | 0.9 | 5 | 16.5 | 1.8 | 1.4 | 1.3 | 1.301 |
| P19823 | Inter-alpha-trypsin inhibitor heavy chain H2 OS=Homo sapiens GN=ITIH2 PE=1 SV=2 | 7 | 3.7 | 1.4 | 0.7 | 0.5 | 2 | 3.0 | 1.5 | 2.0 | 2.7 | * | * | * | * | * | 7 | 3.7 | 0.8 | 0.7 | 1.1 | 1.300 |
| P55327 | Tumor protein D52 OS=Homo sapiens GN=TPD52 PE=1 SV=2 | 36 | 44.2 | 0.7 | 2.2 | 1.0 | 11 | 19.6 | 1.2 | 0.6 | 1.7 | 18 | 38.4 | 1.5 | 1.6 | 0.8 | 17 | 26.3 | 1.8 | 1.3 | 1.4 | 1.300 |
| P01717 | Ig lambda chain V-IV region Hil OS=Homo sapiens PE=1 SV=1 | 8 | 17.8 | 1.6 | 1.1 | 0.6 | 11 | 17.8 | 1.0 | 0.9 | 0.7 | 9 | 17.8 | 1.9 | 2.4 | 1.9 | 10 | 17.8 | 1.0 | 1.0 | 0.9 | 1.298 |
| P60903 | Protein S100-A10 OS=Homo sapiens GN=S100A10 PE=1 SV=2 | 16 | 35.1 | 1.5 | 1.9 | 0.4 | 14 | 45.4 | 0.7 | 0.7 | 0.4 | 13 | 45.4 | 2.7 | 2.3 | 1.2 | 23 | 46.4 | 1.2 | 1.2 | 1.1 | 1.297 |
| P50995 | Annexin A11 OS=Homo sapiens GN=ANXA11 PE=1 SV=1 | 10 | 12.5 | 1.1 | 1.1 | 0.5 | 5 | 11.9 | 1.3 | 0.9 | 1.5 | 17 | 13.1 | 1.8 | 1.9 | 1.1 | 9 | 13.3 | 1.6 | 1.5 | 1.2 | 1.296 |
| Q6XQN6 | Nicotinate phosphoribosyltransferase OS=Homo sapiens GN=NAPRT PE=1 SV=2 | 4 | 9.7 | 0.6 | 1.3 | 1.6 | * | * | * | * | * | 3 | 10.4 | 0.8 | 1.4 | 1.7 | 13 | 17.1 | 1.7 | 1.2 | 1.1 | 1.295 |
| P60953 | Cell division control protein 42 homolog OS=Homo sapiens GN=CDC42 PE=1 SV=2 | 10 | 25.7 | 1.2 | 1.4 | 1.1 | 9 | 21.5 | 1.1 | 0.5 | 0.6 | 18 | 21.5 | 2.3 | 2.6 | 1.6 | 11 | 29.8 | 0.7 | 1.3 | 0.7 | 1.295 |
| Q16795 | NADH dehydrogenase [ubiquinone] 1 alpha subcomplex subunit 9, mitochondrial OS=Homo sapiens GN=NDUFA9 PE=1 SV=2 | 15 | 17.0 | 0.8 | 1.3 | 0.6 | * | * | * | * | * | 13 | 16.2 | 1.2 | 2.4 | 1.6 | 4 | 13.5 | 1.2 | 1.1 | 1.2 | 1.295 |
| O95571 | Persulfide dioxygenase ETHE1, mitochondrial OS=Homo sapiens GN=ETHE1 PE=1 SV=2 | 12 | 24.4 | 0.7 | 1.6 | 0.8 | * | * | * | * | * | 11 | 20.9 | 1.4 | 3.0 | 0.9 | 9 | 12.2 | 0.9 | 1.1 | 1.0 | 1.291 |
| P49189 | 4-trimethylaminobutyraldehyde dehydrogenase OS=Homo sapiens GN=ALDH9A1 PE=1 SV=3 | 5 | 12.1 | 0.6 | 2.7 | 1.3 | 6 | 6.9 | 0.9 | 0.7 | 1.1 | 10 | 12.1 | 1.9 | 2.2 | 1.3 | 9 | 9.7 | 0.6 | 0.8 | 0.7 | 1.288 |
| P25787 | Proteasome subunit alpha type-2 OS=Homo sapiens GN=PSMA2 PE=1 SV=2 | 27 | 38.9 | 1.1 | 1.6 | 1.2 | 21 | 38.5 | 1.2 | 1.0 | 1.0 | 52 | 48.7 | 1.3 | 2.3 | 1.1 | 55 | 44.4 | 1.2 | 1.3 | 1.1 | 1.288 |
| P30626 | Sorcin OS=Homo sapiens GN=SRI PE=1 SV=1 | 8 | 24.7 | 0.8 | 1.5 | 0.6 | 4 | 11.1 | 1.0 | 1.0 | 1.4 | 9 | 20.2 | 1.5 | 2.2 | 1.1 | 8 | 17.7 | 1.8 | 1.3 | 1.4 | 1.287 |
| Q14980 | Nuclear mitotic apparatus protein 1 OS=Homo sapiens GN=NUMA1 PE=1 SV=2 | 4 | 5.1 | 0.6 | 1.7 | 1.9 | * | * | * | * | * | 4 | 2.5 | 1.4 | 1.5 | 1.1 | 5 | 2.1 | 1.3 | 1.0 | 0.7 | 1.286 |
| O75431 | Metaxin-2 OS=Homo sapiens GN=MTX2 PE=1 SV=1 | 2 | 8.4 | 2.4 | 3.2 | 2.0 | 2 | 8.4 | 1.0 | 0.6 | 0.6 | 5 | 8.4 | 1.6 | 0.8 | 1.1 | 2 | 8.4 | 0.3 | 0.5 | 1.2 | 1.280 |
| Q13148 | TAR DNA-binding protein 43 OS=Homo sapiens GN=TARDBP PE=1 SV=1 | 7 | 8.7 | 0.5 | 1.4 | 0.7 | * | * | * | * | * | 12 | 11.6 | 1.8 | 2.3 | 1.5 | 5 | 8.7 | 1.0 | 1.0 | 1.3 | 1.279 |
| O00264 | Membrane-associated progesterone receptor component 1 OS=Homo sapiens GN=PGRMC1 PE=1 SV=3 | 2 | 7.7 | 0.8 | 1.2 | 0.4 | * | * | * | * | * | 8 | 16.4 | 1.5 | 1.4 | 1.4 | 7 | 16.4 | 2.3 | 1.3 | 1.4 | 1.277 |
| P06312 | Ig kappa chain V-IV region OS=Homo sapiens GN=IGKV4-1 PE=1 SV=1 | 11 | 22.3 | 1.7 | 1.0 | 0.6 | 7 | 14.9 | 1.5 | 0.5 | 2.5 | 3 | 14.9 | 1.3 | 1.8 | 1.2 | 23 | 22.3 | 1.1 | 0.9 | 1.0 | 1.274 |
| P11216 | Glycogen phosphorylase, brain form OS=Homo sapiens GN=PYGB PE=1 SV=5 | 18 | 17.6 | 1.0 | 1.4 | 0.9 | 9 | 8.3 | 0.9 | 0.6 | 1.0 | 46 | 18.6 | 1.7 | 2.4 | 1.8 | 31 | 13.3 | 1.4 | 1.2 | 1.1 | 1.273 |
| P22061 | Protein-L-isoaspartate(D-aspartate) O-methyltransferase OS=Homo sapiens GN=PCMT1 PE=1 SV=4 | 10 | 21.1 | 0.9 | 1.6 | 1.0 | * | * | * | * | * | 7 | 36.1 | 1.4 | 2.3 | 1.3 | 9 | 26 | 0.5 | 1.2 | 0.8 | 1.273 |
| P42126 | Enoyl-CoA delta isomerase 1, mitochondrial OS=Homo sapiens GN=ECI1 PE=1 SV=1 | 2 | 13.9 | 0.3 | 0.5 | 0.4 | 3 | 13.9 | 2.8 | 1.9 | 2.2 | 2 | 7.6 | 0.7 | 2.3 | 1.1 | 4 | 9.6 | 1.0 | 0.7 | 0.8 | 1.272 |
| P18669 | Phosphoglycerate mutase 1 OS=Homo sapiens GN=PGAM1 PE=1 SV=2 | 70 | 61.8 | 1.0 | 1.1 | 0.7 | 80 | 52.4 | 1.0 | 0.8 | 1.6 | 95 | 63.4 | 1.4 | 2.3 | 1.2 | 112 | 64.6 | 1.7 | 1.2 | 1.2 | 1.266 |
| P55786 | Puromycin-sensitive aminopeptidase OS=Homo sapiens GN=NPEPPS PE=1 SV=2 | 20 | 14.3 | 1.2 | 2.2 | 0.7 | 2 | 5.0 | 0.9 | 0.2 | 0.3 | 34 | 12.5 | 1.6 | 2.6 | 1.4 | 16 | 10.6 | 1.6 | 1.3 | 1.2 | 1.265 |
| P50502 | Hsc70-interacting protein OS=Homo sapiens GN=ST13 PE=1 SV=2;Putative protein FAM10A5 OS=Homo sapiens GN=ST13P5 PE=5 SV=1 | 7 | 9.8 | 0.8 | 2.0 | 0.9 | 4 | 12.9 | 0.6 | 0.5 | 0.8 | 4 | 7.1 | 1.4 | 2.0 | 1.7 | 8 | 9.8 | 1.3 | 2.0 | 1.5 | 1.265 |
| P56134 | ATP synthase subunit f, mitochondrial OS=Homo sapiens GN=ATP5J2 PE=1 SV=3 | 14 | 11.7 | 0.6 | 1.4 | 0.8 | 7 | 11.7 | 1.1 | 1.1 | 0.7 | 18 | 25.5 | 1.7 | 2.1 | 1.5 | 17 | 25.5 | 1.5 | 1.5 | 1.5 | 1.263 |
| P68036 | Ubiquitin-conjugating enzyme E2 L3 OS=Homo sapiens GN=UBE2L3 PE=1 SV=1 | 24 | 29.9 | 0.9 | 1.6 | 0.9 | 11 | 24.0 | 1.3 | 0.8 | 0.5 | 19 | 24 | 1.7 | 2.5 | 1.1 | 16 | 24 | 1.0 | 1.6 | 1.1 | 1.263 |
| Q9UHD8 | Septin-9 OS=Homo sapiens GN=SEPT9 PE=1 SV=2 | 3 | 8.0 | 0.5 | 1.4 | 1.0 | * | * | * | * | * | 10 | 14.7 | 1.2 | 2.3 | 0.9 | 4 | 7.8 | 1.5 | 1.3 | 1.0 | 1.262 |
| P04040 | sp CATA_HUMAN ;Catalase OS=Homo sapiens GN=CAT PE=1 SV=3 | 13 | 24.5 | 0.9 | 1.3 | 1.2 | 17 | 25.9 | 0.8 | 0.7 | 1.2 | 18 | 18.1 | 1.9 | 2.4 | 1.3 | 15 | 20.2 | 1.0 | 1.2 | 1.2 | 1.259 |
| P62158 | Calmodulin OS=Homo sapiens GN=CALM1 PE=1 SV=2 | 46 | 49.7 | 0.7 | 1.3 | 0.8 | 31 | 74.5 | 0.8 | 0.6 | 1.2 | 11 | 69.8 | 1.5 | 2.1 | 2.0 | 20 | 49.7 | 1.6 | 1.3 | 1.2 | 1.258 |
| P49006 | MARCKS-related protein OS=Homo sapiens GN=MARCKSL1 PE=1 SV=2 | 6 | 14.4 | 1.4 | 1.5 | 2.2 | 4 | 14.4 | 1.9 | 1.3 | 2.0 | 3 | 14.4 | 0.6 | 0.5 | 0.7 | 3 | 14.4 | 1.0 | 0.7 | 2.2 | 1.256 |
| P08758 | sp ANXA5_HUMAN ;Annexin A5 OS=Homo sapiens GN=ANXA5 PE=1 SV=2 | 106 | 67.1 | 1.3 | 1.2 | 0.6 | 114 | 61.8 | 0.8 | 1.1 | 0.8 | 122 | 62.1 | 1.7 | 1.7 | 1.7 | 158 | 51.1 | 1.1 | 1.8 | 1.1 | 1.243 |
| Q71U36 | Tubulin alpha-1A chain OS=Homo sapiens GN=TUBA1A PE=1 SV=1 | 95 | 54.5 | 0.7 | 1.6 | 0.5 | 180 | 53.7 | 1.1 | 1.0 | 0.7 | 87 | 51 | 1.7 | 2.7 | 1.5 | 130 | 51.2 | 1.0 | 1.1 | 1.1 | 1.239 |
| P22695 | Cytochrome b-c1 complex subunit 2, mitochondrial OS=Homo sapiens GN=UQCRC2 PE=1 SV=3 | 33 | 41.9 | 0.5 | 1.3 | 0.7 | 10 | 20.3 | 1.3 | 0.7 | 1.7 | 48 | 31.3 | 1.5 | 2.1 | 1.7 | 30 | 37.7 | 1.0 | 1.3 | 1.1 | 1.239 |
| Q9NTK5 | Obg-like ATPase 1 OS=Homo sapiens GN=OLA1 PE=1 SV=2 | 15 | 10.6 | 1.2 | 2.1 | 0.8 | 16 | 15.9 | 1.0 | 0.8 | 1.0 | 33 | 17.7 | 1.7 | 2.1 | 1.2 | 32 | 8.8 | 0.7 | 1.1 | 0.9 | 1.239 |
| Q5JTH9 | RRP12-like protein OS=Homo sapiens GN=RRP12 PE=1 SV=2 | * | * | * | * | * | 22 | 0.5 | 1.1 | 0.8 | 0.8 | 11 | 2.7 | 1.3 | 2.2 | 1.7 | 10 | 2.7 | 1.3 | 0.7 | 0.9 | 1.237 |
| Q96I99 | Succinate--CoA ligase [GDP-forming] subunit beta, mitochondrial OS=Homo sapiens GN=SUCLG2 PE=1 SV=2 | 9 | 17.1 | 0.5 | 1.0 | 0.8 | 9 | 7.9 | 1.6 | 0.7 | 1.7 | 16 | 16.2 | 1.9 | 2.2 | 1.7 | 5 | 18.5 | 0.6 | 1.0 | 1.0 | 1.235 |
| P31939 | Bifunctional purine biosynthesis protein PURH OS=Homo sapiens GN=ATIC PE=1 SV=3 | 12 | 23.1 | 0.7 | 1.6 | 0.7 | 7 | 14.0 | 0.9 | 0.5 | 1.0 | 37 | 28.2 | 2.1 | 2.0 | 1.3 | 8 | 18.8 | 1.8 | 1.1 | 1.6 | 1.235 |
| Q14247 | Src substrate cortactin OS=Homo sapiens GN=CTTN PE=1 SV=2 | 2 | 6.9 | 0.1 | 1.1 | 0.4 | * | * | * | * | * | 8 | 4 | 1.9 | 2.4 | 0.7 | 10 | 6.9 | 1.5 | 1.8 | 1.4 | 1.234 |
| P13861 | cAMP-dependent protein kinase type II-alpha regulatory subunit OS=Homo sapiens GN=PRKAR2A PE=1 SV=2 | 2 | 3.2 | 1.1 | 2.1 | 2.3 | * | * | * | * | * | 4 | 10.6 | 1.0 | 1.3 | 0.6 | 2 | 3.5 | 0.6 | 0.8 | 0.9 | 1.234 |
| P01834 | Ig kappa chain C region OS=Homo sapiens GN=IGKC PE=1 SV=1 | 262 | 62.3 | 1.8 | 1.0 | 0.8 | 321 | 61.3 | 1.8 | 0.7 | 1.0 | 241 | 49.1 | 1.3 | 1.6 | 1.5 | 237 | 49.1 | 0.8 | 1.1 | 1.1 | 1.232 |
| Q16836 | Hydroxyacyl-coenzyme A dehydrogenase, mitochondrial OS=Homo sapiens GN=HADH PE=1 SV=3 | 60 | 52.2 | 0.7 | 1.8 | 1.0 | 22 | 27.1 | 0.8 | 0.9 | 0.9 | 53 | 58.3 | 1.8 | 1.9 | 1.6 | 48 | 42 | 0.7 | 1.5 | 1.1 | 1.231 |
| P35527 | sp K1C9_HUMAN ;Keratin, type I cytoskeletal 9 OS=Homo sapiens GN=KRT9 PE=1 SV=3 | 7 | 24.2 | 0.9 | 1.2 | 0.8 | 22 | 31.1 | 1.1 | 0.7 | 1.7 | 11 | 25.2 | 1.4 | 1.3 | 1.8 | 24 | 32.4 | 1.3 | 1.3 | 1.5 | 1.227 |
| O75083 | WD repeat-containing protein 1 OS=Homo sapiens GN=WDR1 PE=1 SV=4 | 43 | 28.4 | 0.9 | 2.0 | 0.9 | 18 | 19.3 | 1.0 | 0.7 | 1.7 | 35 | 29 | 1.4 | 2.3 | 1.2 | 33 | 25.4 | 0.9 | 0.7 | 0.8 | 1.226 |
| O15145 | Actin-related protein 2/3 complex subunit 3 OS=Homo sapiens GN=ARPC3 PE=1 SV=3 | 5 | 6.2 | 1.0 | 1.7 | 0.3 | 4 | 10.1 | 1.3 | 1.0 | 0.9 | 9 | 6.2 | 0.8 | 3.6 | 0.6 | * | * | * | * | * | 1.226 |
| P00441 | Superoxide dismutase [Cu-Zn] OS=Homo sapiens GN=SOD1 PE=1 SV=2;sp SODC_HUMAN | 27 | 13.7 | 0.6 | 1.4 | 1.0 | 30 | 13.7 | 1.4 | 0.9 | 1.5 | 24 | 13.7 | 1.5 | 1.6 | 1.4 | 24 | 21.6 | 1.2 | 1.1 | 0.9 | 1.226 |
| P61026 | Ras-related protein Rab-10 OS=Homo sapiens GN=RAB10 PE=1 SV=1 | 5 | 16.5 | 0.5 | 1.3 | 0.7 | * | * | * | * | * | 2 | 11 | 1.3 | 3.5 | 0.5 | 5 | 22.5 | 0.6 | 1.5 | 1.3 | 1.226 |
| P36957 | Dihydrolipoyllysine-residue succinyltransferase component of 2-oxoglutarate dehydrogenase complex, mitochondrial OS=Homo sapiens GN=DLST PE=1 SV=4 | 18 | 21.6 | 0.8 | 1.6 | 0.9 | 24 | 22.7 | 1.5 | 0.6 | 1.3 | 9 | 9.7 | 1.0 | 1.7 | 1.1 | 15 | 16.6 | 1.4 | 1.5 | 0.9 | 1.222 |
| P23142 | Fibulin-1 OS=Homo sapiens GN=FBLN1 PE=1 SV=4 | 21 | 13.1 | 2.1 | 1.1 | 0.7 | * | * | * | * | * | 2 | 4.7 | 0.7 | 0.2 | 1.1 | 14 | 5.7 | 1.7 | 2.2 | 1.2 | 1.222 |
| Q15257 | Serine/threonine-protein phosphatase 2A activator OS=Homo sapiens GN=PPP2R4 PE=1 SV=3 | 3 | 11.7 | 0.5 | 0.5 | 0.6 | * | * | * | * | * | 4 | 3.6 | 1.6 | 2.6 | 0.9 | 8 | 11.7 | 1.4 | 1.7 | 1.3 | 1.221 |
| P10768 | S-formylglutathione hydrolase OS=Homo sapiens GN=ESD PE=1 SV=2 | 6 | 23.0 | 1.2 | 1.1 | 0.8 | * | * | * | * | * | 17 | 34 | 1.6 | 1.7 | 0.9 | 12 | 34.8 | 0.9 | 1.4 | 1.3 | 1.221 |
| P25705 | ATP synthase subunit alpha, mitochondrial OS=Homo sapiens GN=ATP5A1 PE=1 SV=1 | 203 | 40.3 | 0.8 | 1.5 | 0.7 | 129 | 38.0 | 1.0 | 0.7 | 1.0 | 207 | 44.3 | 1.9 | 2.5 | 1.1 | 199 | 43.8 | 1.0 | 1.4 | 1.1 | 1.219 |
| P07437 | Tubulin beta chain OS=Homo sapiens GN=TUBB PE=1 SV=2 | 77 | 48.0 | 0.9 | 1.7 | 0.6 | 118 | 57.0 | 0.9 | 0.8 | 0.7 | 140 | 58.8 | 1.9 | 1.7 | 1.4 | 103 | 61.3 | 1.1 | 1.8 | 1.2 | 1.219 |
| P17174 | Aspartate aminotransferase, cytoplasmic OS=Homo sapiens GN=GOT1 PE=1 SV=3 | 2 | 11.6 | 0.4 | 1.0 | 0.6 | * | * | * | * | * | 12 | 24.9 | 1.8 | 3.4 | 0.9 | 5 | 11.9 | 1.0 | 0.7 | 1.3 | 1.218 |
| P00387 | NADH-cytochrome b5 reductase 3 OS=Homo sapiens GN=CYB5R3 PE=1 SV=3 | 13 | 30.6 | 1.0 | 1.3 | 0.5 | 9 | 18.3 | 1.1 | 1.1 | 0.8 | 16 | 27.2 | 1.3 | 2.0 | 1.7 | 10 | 19.6 | 1.5 | 1.2 | 1.2 | 1.215 |
| P26447 | Protein S100-A4 OS=Homo sapiens GN=S100A4 PE=1 SV=1 | 26 | 27.7 | 1.3 | 1.3 | 0.5 | 22 | 27.7 | 0.7 | 0.9 | 1.0 | 21 | 27.7 | 1.7 | 2.1 | 1.5 | 24 | 27.7 | 1.2 | 1.5 | 1.3 | 1.215 |
| B9A064 | Immunoglobulin lambda-like polypeptide 5 OS=Homo sapiens GN=IGLL5 PE=2 SV=2 | 39 | 19.6 | 1.2 | 1.0 | 0.9 | 26 | 31.3 | 2.5 | 0.9 | 1.1 | 12 | 19.6 | 1.0 | 1.3 | 1.1 | 25 | 34.6 | 1.0 | 1.4 | 1.0 | 1.214 |
| P55084 | Trifunctional enzyme subunit beta, mitochondrial OS=Homo sapiens GN=HADHB PE=1 SV=3 | 28 | 20.0 | 0.7 | 1.3 | 0.9 | 21 | 17.3 | 1.0 | 0.8 | 1.0 | 43 | 26.6 | 1.9 | 2.1 | 1.3 | 24 | 21.7 | 0.9 | 1.4 | 1.2 | 1.213 |
| P09104 | Gamma-enolase OS=Homo sapiens GN=ENO2 PE=1 SV=3 | 2 | 23.7 | 1.9 | 2.0 | 1.9 | 2 | 20.7 | 0.7 | 0.3 | 1.1 | * | * | * | * | * | 4 | 21.9 | 1.0 | 0.8 | 0.6 | 1.209 |
| P40227 | T-complex protein 1 subunit zeta OS=Homo sapiens GN=CCT6A PE=1 SV=3 | 26 | 27.1 | 0.8 | 1.7 | 0.5 | 13 | 11.5 | 0.8 | 0.9 | 1.2 | 41 | 24.3 | 1.3 | 1.9 | 1.3 | 33 | 19.6 | 1.2 | 1.6 | 1.4 | 1.204 |
| P24539 | ATP synthase F(0) complex subunit B1, mitochondrial OS=Homo sapiens GN=ATP5F1 PE=1 SV=2 | 9 | 35.2 | 1.1 | 1.2 | 0.6 | 8 | 24.2 | 1.2 | 1.0 | 1.6 | 16 | 24.6 | 1.5 | 2.4 | 0.9 | 10 | 18 | 0.6 | 1.2 | 0.8 | 1.202 |
| P30084 | Enoyl-CoA hydratase, mitochondrial OS=Homo sapiens GN=ECHS1 PE=1 SV=4 | 19 | 18.3 | 0.9 | 1.5 | 0.8 | 9 | 7.2 | 0.9 | 0.6 | 1.4 | 27 | 19.7 | 1.5 | 1.5 | 1.0 | 22 | 22.8 | 1.2 | 2.0 | 1.4 | 1.200 |
| P60709 | Actin, cytoplasmic 1 OS=Homo sapiens GN=ACTB PE=1 SV=1;Actin, cytoplasmic 2 OS=Homo sapiens GN=ACTG1 PE=1 SV=1 | 570 | 76.0 | 1.1 | 1.4 | 0.6 | 573 | 82.4 | 1.1 | 0.9 | 0.8 | 707 | 76.3 | 1.4 | 2.1 | 1.3 | 924 | 76.8 | 1.0 | 1.4 | 1.1 | 1.200 |
| P26639 | Threonine--tRNA ligase, cytoplasmic OS=Homo sapiens GN=TARS PE=1 SV=3 | 5 | 5.8 | 0.8 | 2.2 | 0.8 | 2 | 3.0 | 1.1 | 0.3 | 1.2 | 2 | 3 | 0.7 | 1.6 | 1.4 | 24 | 10.7 | 1.8 | 1.3 | 1.2 | 1.198 |
| P04433 | Ig kappa chain V-III region VG (Fragment) OS=Homo sapiens PE=1 SV=1 | 16 | 23.5 | 1.3 | 1.0 | 0.8 | 29 | 29.6 | 2.4 | 0.8 | 1.0 | 9 | 23.5 | 1.0 | 1.3 | 1.5 | 10 | 23.5 | 1.0 | 1.0 | 1.0 | 1.194 |
| Q16543 | Hsp90 co-chaperone Cdc37 OS=Homo sapiens GN=CDC37 PE=1 SV=1 | 5 | 10.1 | 0.8 | 0.7 | 1.0 | 6 | 15.3 | 1.0 | 0.8 | 1.9 | 2 | 10.8 | 0.9 | 2.1 | 1.0 | 6 | 10.1 | 2.3 | 0.6 | 1.3 | 1.193 |
| P25311 | Zinc-alpha-2-glycoprotein OS=Homo sapiens GN=AZGP1 PE=1 SV=2 | 12 | 22.1 | 1.7 | 1.6 | 0.9 | 5 | 9.4 | 0.9 | 0.5 | 0.8 | 4 | 9.4 | 1.6 | 1.6 | 0.9 | 5 | 14.4 | 1.2 | 1.4 | 1.1 | 1.192 |
| P01008 | sp ANT3_HUMAN ;Antithrombin-III OS=Homo sapiens GN=SERPINC1 PE=1 SV=1 | 7 | 2.6 | 0.6 | 0.8 | 0.5 | 5 | 2.6 | 0.5 | 0.5 | 1.0 | 6 | 5 | 2.7 | 2.6 | 1.8 | 4 | 5 | 1.2 | 1.0 | 0.9 | 1.190 |
| Q9NQC3 | Reticulon-4 OS=Homo sapiens GN=RTN4 PE=1 SV=2 | 17 | 3.6 | 0.8 | 1.3 | 0.9 | 33 | 3.9 | 0.6 | 0.7 | 0.9 | 27 | 5.8 | 1.4 | 1.9 | 1.1 | 22 | 4 | 1.9 | 1.6 | 1.1 | 1.190 |
| P54868 | Hydroxymethylglutaryl-CoA synthase, mitochondrial OS=Homo sapiens GN=HMGCS2 PE=1 SV=1 | 12 | 21.7 | 0.8 | 1.4 | 1.6 | 5 | 18.3 | 0.5 | 0.3 | 1.0 | 4 | 14.2 | 1.2 | 2.1 | 0.7 | 10 | 11.8 | 1.9 | 1.7 | 1.7 | 1.186 |
| O75947 | ATP synthase subunit d, mitochondrial OS=Homo sapiens GN=ATP5H PE=1 SV=3 | 5 | 47.2 | 1.4 | 1.8 | 0.5 | * | * | * | * | * | 9 | 41.6 | 1.1 | 1.6 | 1.1 | 8 | 20.5 | 1.0 | 1.0 | 0.8 | 1.184 |
| P04083 | Annexin A1 OS=Homo sapiens GN=ANXA1 PE=1 SV=2 | 84 | 46.8 | 1.5 | 1.2 | 0.5 | 67 | 45.7 | 1.2 | 1.0 | 0.9 | 57 | 42.2 | 1.3 | 1.4 | 1.8 | 94 | 44.5 | 0.7 | 1.5 | 1.0 | 1.179 |
| P02766 | Transthyretin OS=Homo sapiens GN=TTR PE=1 SV=1 | 32 | 50.3 | 1.6 | 1.4 | 1.1 | 11 | 50.3 | 1.0 | 0.4 | 1.4 | 5 | 40.8 | 1.7 | 1.2 | 1.0 | 11 | 40.1 | 1.1 | 0.9 | 0.8 | 1.176 |
| Q15582 | Transforming growth factor-beta-induced protein ig-h3 OS=Homo sapiens GN=TGFBI PE=1 SV=1 | 32 | 15.2 | 1.6 | 1.0 | 1.0 | 25 | 13.8 | 0.7 | 0.9 | 1.0 | 18 | 16.4 | 0.9 | 1.4 | 1.1 | 20 | 13.9 | 1.6 | 1.8 | 1.6 | 1.176 |
| P02765 | Alpha-2-HS-glycoprotein OS=Homo sapiens GN=AHSG PE=1 SV=1 | 9 | 12.8 | 1.7 | 1.8 | 0.8 | 18 | 12.8 | 1.3 | 0.9 | 1.0 | 10 | 12.8 | 1.2 | 1.5 | 1.1 | 7 | 10.9 | 0.9 | 0.6 | 0.7 | 1.175 |
| P01620 | Ig kappa chain V-III region SIE OS=Homo sapiens PE=1 SV=1;Ig kappa chain V-III region WOL OS=Homo sapiens PE=1 SV=1 | 56 | 39.4 | 1.5 | 0.7 | 0.6 | * | * | * | * | * | 52 | 31.2 | 1.1 | 1.7 | 1.4 | 53 | 31.2 | 1.0 | 1.3 | 1.0 | 1.174 |
| Q92499 | ATP-dependent RNA helicase DDX1 OS=Homo sapiens GN=DDX1 PE=1 SV=2 | 6 | 5.0 | 0.8 | 1.1 | 0.6 | 5 | 4.9 | 0.7 | 1.5 | 0.8 | 7 | 10.5 | 1.9 | 2.6 | 1.2 | 11 | 10.5 | 0.6 | 0.9 | 1.0 | 1.171 |
| P27816 | Microtubule-associated protein 4 OS=Homo sapiens GN=MAP4 PE=1 SV=3 | 3 | 7.2 | 1.1 | 1.4 | 1.1 | 7 | 3.6 | 0.6 | 0.8 | 0.9 | 12 | 6 | 1.2 | 2.2 | 1.2 | 6 | 4.3 | 1.2 | 1.4 | 0.8 | 1.169 |
| Q9Y6N5 | Sulfide:quinone oxidoreductase, mitochondrial OS=Homo sapiens GN=SQRDL PE=1 SV=1 | 42 | 31.3 | 0.8 | 1.3 | 1.3 | 20 | 27.6 | 0.9 | 0.7 | 0.8 | 43 | 38 | 1.5 | 2.5 | 1.1 | 32 | 25.6 | 0.8 | 0.9 | 0.9 | 1.160 |
| P30086 | Phosphatidylethanolamine-binding protein 1 OS=Homo sapiens GN=PEBP1 PE=1 SV=3 | 101 | 54.5 | 0.9 | 1.2 | 1.0 | 110 | 58.8 | 0.9 | 0.7 | 1.0 | 83 | 58.8 | 1.5 | 1.8 | 1.2 | 94 | 56.7 | 1.3 | 1.2 | 1.0 | 1.160 |
| Q15843 | sp NEDD8_HUMAN ;NEDD8 OS=Homo sapiens GN=NEDD8 PE=1 SV=1 | 3 | 17.3 | 0.3 | 1.3 | 0.6 | 2 | 17.3 | 1.2 | 1.8 | 1.0 | 3 | 17.3 | 0.5 | 2.1 | 0.6 | 4 | 17.3 | 1.7 | 1.5 | 2.0 | 1.156 |
| P04350 | Tubulin beta-4A chain OS=Homo sapiens GN=TUBB4A PE=1 SV=2 | 21 | 44.8 | 0.9 | 1.4 | 1.0 | 11 | 48.0 | 0.8 | 1.0 | 0.5 | 14 | 40.3 | 0.9 | 1.9 | 1.2 | 10 | 54.1 | 1.8 | 1.3 | 1.2 | 1.151 |
| P01861 | Ig gamma-4 chain C region OS=Homo sapiens GN=IGHG4 PE=1 SV=1 | 13 | 43.7 | 3.3 | 1.0 | 0.6 | 18 | 30.3 | 1.4 | 1.2 | 1.0 | 2 | 24.2 | 0.7 | 0.6 | 0.9 | 8 | 30.3 | 0.9 | 1.2 | 1.1 | 1.143 |
| P23141 | Liver carboxylesterase 1 OS=Homo sapiens GN=CES1 PE=1 SV=2 | 2 | 4.9 | 0.5 | 1.1 | 0.3 | * | * | * | * | * | 8 | 21 | 2.9 | 1.2 | 1.5 | 3 | 9.7 | 0.6 | 1.0 | 0.8 | 1.143 |
| P23528 | Cofilin-1 OS=Homo sapiens GN=CFL1 PE=1 SV=3 | 106 | 54.2 | 1.0 | 1.7 | 0.5 | 137 | 51.8 | 1.3 | 1.0 | 0.7 | 130 | 54.2 | 1.6 | 2.4 | 1.0 | 47 | 54.2 | 0.6 | 0.7 | 0.8 | 1.143 |
| O60701 | UDP-glucose 6-dehydrogenase OS=Homo sapiens GN=UGDH PE=1 SV=1 | 10 | 19.0 | 1.3 | 1.7 | 0.7 | 5 | 12.8 | 0.8 | 0.8 | 1.0 | 5 | 4.5 | 0.9 | 2.5 | 1.1 | 6 | 7.1 | 0.5 | 1.3 | 1.1 | 1.143 |
| P32119 | Peroxiredoxin-2 OS=Homo sapiens GN=PRDX2 PE=1 SV=5 | 187 | 42.4 | 0.7 | 1.1 | 0.9 | 133 | 42.4 | 1.0 | 0.6 | 1.2 | 187 | 38.9 | 1.5 | 2.1 | 1.1 | 137 | 46 | 1.4 | 1.0 | 1.0 | 1.141 |
| P07148 | Fatty acid-binding protein, liver OS=Homo sapiens GN=FABP1 PE=1 SV=1 | 67 | 51.2 | 0.3 | 1.1 | 0.8 | 194 | 51.2 | 0.7 | 0.4 | 2.3 | 194 | 62.2 | 0.8 | 3.4 | 1.3 | 72 | 61.4 | 0.6 | 0.8 | 1.0 | 1.138 |
| P62873 | Guanine nucleotide-binding protein G(I)/G(S)/G(T) subunit beta-1 OS=Homo sapiens GN=GNB1 PE=1 SV=3;Guanine nucleotide-binding protein G(I)/G(S)/G(T) subunit beta-2 OS=Homo sapiens GN=GNB2 PE=1 SV=3;Guanine nucleotide-binding protein subunit beta-4 OS=Homo sapiens GN=GNB4 PE=1 SV=3 | 5 | 6.2 | 0.9 | 1.3 | 1.5 | 2 | 2.9 | 0.9 | 0.5 | 2.0 | 4 | 2.9 | 1.0 | 1.5 | 1.0 | 6 | 6.2 | 0.6 | 1.3 | 0.5 | 1.137 |
| Q14019 | Coactosin-like protein OS=Homo sapiens GN=COTL1 PE=1 SV=3 | 5 | 18.3 | 0.4 | 0.8 | 0.9 | * | * | * | * | * | 7 | 24.6 | 1.5 | 1.2 | 1.2 | 19 | 12.7 | 1.1 | 2.1 | 1.1 | 1.136 |
| P04264 | sp K2C1_HUMAN ;Keratin, type II cytoskeletal 1 OS=Homo sapiens GN=KRT1 PE=1 SV=6 | 52 | 44.6 | 0.8 | 0.8 | 0.6 | 160 | 43.5 | 1.5 | 0.9 | 1.8 | 44 | 34.6 | 1.1 | 1.1 | 1.3 | 105 | 41.8 | 1.2 | 1.4 | 1.7 | 1.135 |
| P01876 | Ig alpha-1 chain C region OS=Homo sapiens GN=IGHA1 PE=1 SV=2 | 38 | 26.6 | 1.4 | 0.7 | 0.8 | 60 | 21.0 | 1.8 | 0.4 | 0.8 | 33 | 21 | 1.3 | 1.9 | 1.1 | 34 | 21 | 1.1 | 1.1 | 1.2 | 1.131 |
| O60844 | Zymogen granule membrane protein 16 OS=Homo sapiens GN=ZG16 PE=1 SV=2 | 2 | 10.8 | 0.5 | 1.9 | 2.3 | * | * | * | * | * | 5 | 20.4 | 1.0 | 1.7 | 0.6 | 2 | 20.4 | 0.5 | 0.5 | 0.4 | 1.131 |
| P26038 | Moesin OS=Homo sapiens GN=MSN PE=1 SV=3 | 41 | 36.2 | 1.3 | 1.3 | 0.7 | 35 | 27.7 | 0.7 | 1.0 | 0.9 | 21 | 22.7 | 0.9 | 1.2 | 1.0 | 48 | 38.6 | 1.8 | 1.6 | 1.1 | 1.128 |
| Q9H0R4 | Haloacid dehalogenase-like hydrolase domain-containing protein 2 OS=Homo sapiens GN=HDHD2 PE=1 SV=1 | * | * | * | * | * | 3 | 5.0 | 0.8 | 0.4 | 1.2 | 6 | 5 | 1.9 | 1.8 | 1.1 | 2 | 5 | 0.6 | 1.4 | 1.0 | 1.126 |
| P04632 | Calpain small subunit 1 OS=Homo sapiens GN=CAPNS1 PE=1 SV=1 | 10 | 39.2 | 0.8 | 1.2 | 0.5 | 3 | 38.4 | 1.2 | 1.2 | 0.8 | 7 | 36.6 | 1.7 | 1.9 | 0.9 | 16 | 38.8 | 1.2 | 1.0 | 1.1 | 1.125 |
| P13489 | Ribonuclease inhibitor OS=Homo sapiens GN=RNH1 PE=1 SV=2 | 9 | 5.6 | 1.1 | 1.6 | 1.0 | 6 | 5.6 | 1.1 | 1.0 | 1.0 | * | * | * | * | * | 12 | 8.9 | 1.1 | 1.0 | 1.0 | 1.122 |
| Q16555 | Dihydropyrimidinase-related protein 2 OS=Homo sapiens GN=DPYSL2 PE=1 SV=1 | 5 | 17.0 | 1.0 | 0.9 | 0.5 | 4 | 17.0 | 0.9 | 1.1 | 0.6 | 8 | 12.9 | 2.2 | 2.4 | 1.2 | 9 | 15.9 | 0.6 | 0.9 | 0.7 | 1.122 |
| P30049 | ATP synthase subunit delta, mitochondrial OS=Homo sapiens GN=ATP5D PE=1 SV=2 | 15 | 37.5 | 0.6 | 1.4 | 0.3 | 8 | 37.5 | 0.9 | 0.7 | 1.2 | 9 | 32.1 | 1.8 | 3.2 | 1.1 | 20 | 44 | 0.5 | 0.7 | 0.8 | 1.120 |
| P98179 | RNA-binding protein 3 OS=Homo sapiens GN=RBM3 PE=1 SV=1 | 9 | 11.5 | 0.7 | 1.2 | 0.8 | 7 | 11.5 | 0.8 | 0.9 | 0.6 | 7 | 11.5 | 1.2 | 2.4 | 1.5 | 7 | 11.5 | 1.2 | 1.0 | 1.0 | 1.118 |
| P19367 | Hexokinase-1 OS=Homo sapiens GN=HK1 PE=1 SV=3 | 7 | 3.1 | 1.2 | 1.4 | 1.1 | 2 | 1.6 | 0.3 | 0.3 | 0.2 | 7 | 4.3 | 1.6 | 2.3 | 1.1 | 8 | 6.2 | 1.2 | 1.6 | 1.5 | 1.114 |
| Q9UFN0 | Protein NipSnap homolog 3A OS=Homo sapiens GN=NIPSNAP3A PE=1 SV=2 | 2 | 9.3 | 0.8 | 2.0 | 0.2 | * | * | * | * | * | 3 | 9.3 | 1.4 | 1.2 | 0.9 | 2 | 9.3 | 0.9 | 1.4 | 1.2 | 1.111 |
| P04114 | Apolipoprotein B-100 OS=Homo sapiens GN=APOB PE=1 SV=2 | 10 | 2.9 | 1.6 | 1.3 | 0.8 | 18 | 4.0 | 1.8 | 0.7 | 1.3 | 4 | 1.1 | 1.2 | 0.9 | 0.8 | 10 | 2.7 | 0.9 | 0.9 | 1.5 | 1.110 |
| P02679 | Fibrinogen gamma chain OS=Homo sapiens GN=FGG PE=1 SV=3 | 80 | 34.4 | 1.4 | 1.0 | 0.7 | 90 | 32.0 | 1.5 | 0.6 | 1.6 | 55 | 24.9 | 1.0 | 1.6 | 1.1 | 58 | 30.2 | 1.1 | 0.8 | 1.0 | 1.110 |
| P56470 | Galectin-4 OS=Homo sapiens GN=LGALS4 PE=1 SV=1 | 56 | 30.0 | 0.7 | 1.7 | 1.3 | 23 | 28.2 | 0.5 | 0.6 | 0.7 | 44 | 26 | 1.5 | 2.0 | 1.1 | 46 | 26.6 | 1.5 | 0.7 | 1.0 | 1.108 |
| P00747 | Plasminogen OS=Homo sapiens GN=PLG PE=1 SV=2 | 2 | 5.7 | 0.9 | 0.9 | 0.6 | 4 | 5.3 | 2.1 | 0.4 | 2.3 | * | * | * | * | * | 2 | 2.7 | 1.0 | 0.7 | 1.6 | 1.107 |
| Q15063 | Periostin OS=Homo sapiens GN=POSTN PE=1 SV=2 | 10 | 7.3 | 1.3 | 1.0 | 0.8 | 8 | 6.6 | 0.9 | 1.5 | 1.0 | * | * | * | * | * | 8 | 5.5 | 0.4 | 1.9 | 0.9 | 1.106 |
| Q15019 | Septin-2 OS=Homo sapiens GN=SEPT2 PE=1 SV=1 | 18 | 37.1 | 0.8 | 1.5 | 0.7 | 8 | 15.8 | 0.9 | 0.8 | 0.5 | 14 | 22.7 | 1.9 | 2.4 | 0.9 | 15 | 22.7 | 0.8 | 1.0 | 1.0 | 1.103 |
| P16152 | Carbonyl reductase [NADPH] 1 OS=Homo sapiens GN=CBR1 PE=1 SV=3 | 33 | 28.2 | 0.8 | 1.2 | 0.7 | 18 | 30.7 | 1.0 | 0.5 | 0.9 | 30 | 22.7 | 1.3 | 1.5 | 1.2 | 26 | 32.5 | 1.4 | 1.6 | 1.1 | 1.100 |
| Q9P0J0 | NADH dehydrogenase [ubiquinone] 1 alpha subcomplex subunit 13 OS=Homo sapiens GN=NDUFA13 PE=1 SV=3 | 7 | 25.7 | 2.1 | 2.6 | 0.6 | 2 | 9.0 | 0.5 | 0.8 | 0.3 | 13 | 18.8 | 1.0 | 1.4 | 1.0 | 4 | 18.8 | 1.0 | 0.8 | 1.6 | 1.098 |
| P19827 | Inter-alpha-trypsin inhibitor heavy chain H1 OS=Homo sapiens GN=ITIH1 PE=1 SV=3 | 2 | 2.5 | 1.5 | 1.3 | 0.5 | 3 | 3.4 | 0.9 | 1.3 | 0.8 | * | * | * | * | * | 5 | 1.4 | 1.3 | 1.3 | 1.4 | 1.094 |
| A8K7I4 | Calcium-activated chloride channel regulator 1 OS=Homo sapiens GN=CLCA1 PE=1 SV=3 | 81 | 25.1 | 0.6 | 0.9 | 2.6 | 2 | 7.9 | 2.4 | 1.1 | 0.9 | 9 | 9.6 | 0.8 | 1.2 | 0.5 | 17 | 13.8 | 0.4 | 0.5 | 0.6 | 1.092 |
| P02749 | Beta-2-glycoprotein 1 OS=Homo sapiens GN=APOH PE=1 SV=3 | 4 | 5.2 | 1.1 | 1.0 | 0.7 | 5 | 8.4 | 1.4 | 0.8 | 1.2 | * | * | * | * | * | 5 | 2.6 | 1.4 | 1.1 | 0.9 | 1.088 |
| P28331 | NADH-ubiquinone oxidoreductase 75 kDa subunit, mitochondrial OS=Homo sapiens GN=NDUFS1 PE=1 SV=3 | 2 | 6.3 | 0.6 | 0.5 | 0.6 | 4 | 4.5 | 1.0 | 0.8 | 1.5 | 3 | 9.8 | 1.3 | 2.4 | 1.1 | 3 | 4.5 | 0.8 | 1.3 | 1.1 | 1.087 |
| P06576 | ATP synthase subunit beta, mitochondrial OS=Homo sapiens GN=ATP5B PE=1 SV=3 | 278 | 61.6 | 0.9 | 1.6 | 0.5 | 263 | 59.9 | 0.9 | 0.6 | 0.6 | 481 | 58 | 1.6 | 2.4 | 1.1 | 289 | 60.7 | 0.6 | 1.2 | 0.9 | 1.081 |
| P01024 | Complement C3 OS=Homo sapiens GN=C3 PE=1 SV=2 | 172 | 38.1 | 1.2 | 1.1 | 0.8 | 165 | 36.2 | 1.4 | 0.7 | 1.4 | 84 | 23.2 | 1.2 | 1.5 | 1.1 | 83 | 31.1 | 0.8 | 0.7 | 0.9 | 1.080 |
| P30041 | Peroxiredoxin-6 OS=Homo sapiens GN=PRDX6 PE=1 SV=3 | 66 | 62.5 | 0.9 | 1.4 | 0.6 | 46 | 62.5 | 0.9 | 0.6 | 1.0 | 56 | 53.6 | 1.6 | 2.0 | 1.2 | 80 | 62.5 | 0.8 | 0.9 | 0.9 | 1.080 |
| P0C0L4 | Complement C4-A OS=Homo sapiens GN=C4A PE=1 SV=2;Complement C4-B OS=Homo sapiens GN=C4B PE=1 SV=2 | 60 | 21.4 | 1.3 | 1.0 | 0.8 | * | * | * | * | * | 35 | 13.6 | 1.3 | 1.5 | 1.2 | 63 | 17.1 | 0.8 | 0.8 | 1.1 | 1.079 |
| P02675 | Fibrinogen beta chain OS=Homo sapiens GN=FGB PE=1 SV=2 | 66 | 29.5 | 1.3 | 1.1 | 0.9 | 82 | 37.9 | 1.2 | 0.6 | 1.3 | 26 | 24 | 1.2 | 1.6 | 1.0 | 44 | 26.7 | 0.8 | 0.8 | 1.0 | 1.076 |
| P07737 | Profilin-1 OS=Homo sapiens GN=PFN1 PE=1 SV=2 | 380 | 67.9 | 0.9 | 1.2 | 0.7 | 368 | 67.1 | 0.8 | 0.6 | 1.1 | 377 | 67.1 | 1.2 | 1.8 | 1.1 | 391 | 69.3 | 1.3 | 1.1 | 1.0 | 1.076 |
| Q16822 | Phosphoenolpyruvate carboxykinase [GTP], mitochondrial OS=Homo sapiens GN=PCK2 PE=1 SV=3 | 3 | 10.8 | 0.6 | 0.9 | 0.5 | 3 | 6.6 | 0.4 | 0.4 | 0.4 | 18 | 15.9 | 1.9 | 2.3 | 2.0 | 6 | 12 | 1.0 | 1.5 | 1.5 | 1.074 |
| P02671 | Fibrinogen alpha chain OS=Homo sapiens GN=FGA PE=1 SV=2 | 113 | 27.1 | 1.3 | 1.0 | 0.7 | 105 | 30.5 | 1.1 | 0.5 | 1.3 | 42 | 21.4 | 1.2 | 1.7 | 1.2 | 56 | 24.8 | 1.1 | 0.7 | 1.1 | 1.073 |
| P05556 | Integrin beta-1 OS=Homo sapiens GN=ITGB1 PE=1 SV=2 | 17 | 13.3 | 0.7 | 0.8 | 0.6 | 31 | 12.0 | 0.8 | 1.1 | 1.0 | 22 | 9.3 | 1.5 | 2.1 | 1.6 | 21 | 7.9 | 0.7 | 1.0 | 0.7 | 1.069 |
| P21810 | Biglycan OS=Homo sapiens GN=BGN PE=1 SV=2 | 14 | 16.6 | 1.9 | 1.0 | 0.5 | 5 | 16.8 | 1.1 | 1.2 | 1.3 | * | * | * | * | * | 6 | 7.6 | 0.4 | 1.1 | 1.0 | 1.068 |
| P00488 | Coagulation factor XIII A chain OS=Homo sapiens GN=F13A1 PE=1 SV=4 | 3 | 7.5 | 1.1 | 0.9 | 0.6 | 6 | 7.9 | 0.5 | 1.8 | 1.3 | 5 | 2.3 | 0.6 | 1.9 | 0.8 | 5 | 4 | 0.8 | 1.4 | 0.8 | 1.060 |
| P00751 | Complement factor B OS=Homo sapiens GN=CFB PE=1 SV=2 | 11 | 14.4 | 1.1 | 1.1 | 0.6 | 16 | 11.5 | 1.2 | 0.7 | 1.0 | 5 | 3.3 | 1.6 | 2.1 | 1.3 | 7 | 3.3 | 0.5 | 0.6 | 0.9 | 1.059 |
| Q15417 | Calponin-3 OS=Homo sapiens GN=CNN3 PE=1 SV=1 | 4 | 22.2 | 0.8 | 0.8 | 0.5 | * | * | * | * | * | 2 | 23.1 | 0.8 | 1.8 | 1.2 | 3 | 12.8 | 1.0 | 1.5 | 0.6 | 1.059 |
| P27348 | 14-3-3 protein theta OS=Homo sapiens GN=YWHAQ PE=1 SV=1 | 5 | 27.3 | 0.5 | 1.5 | 0.8 | 10 | 42.4 | 0.8 | 0.8 | 0.7 | 3 | 35.1 | 1.1 | 2.1 | 0.8 | 5 | 34.3 | 1.2 | 1.5 | 1.1 | 1.059 |
| O95994 | Anterior gradient protein 2 homolog OS=Homo sapiens GN=AGR2 PE=1 SV=1 | 163 | 54.9 | 0.6 | 1.8 | 1.4 | 93 | 46.9 | 0.6 | 0.3 | 0.9 | 141 | 52.6 | 1.3 | 2.4 | 0.8 | 111 | 49.1 | 1.2 | 0.5 | 0.9 | 1.047 |
| P01871 | Ig mu chain C region OS=Homo sapiens GN=IGHM PE=1 SV=3 | 54 | 24.3 | 0.9 | 1.2 | 1.5 | 96 | 27.9 | 1.5 | 0.9 | 0.8 | 33 | 21.5 | 1.3 | 1.4 | 0.8 | 27 | 19.2 | 0.6 | 0.7 | 1.0 | 1.046 |
| P00450 | Ceruloplasmin OS=Homo sapiens GN=CP PE=1 SV=1 | 39 | 8.1 | 1.5 | 1.1 | 0.8 | 39 | 9.1 | 1.8 | 1.2 | 1.2 | 16 | 5 | 0.8 | 0.8 | 1.0 | 25 | 10.2 | 0.4 | 0.9 | 1.0 | 1.043 |
| P0CG05 | Ig lambda-2 chain C regions OS=Homo sapiens GN=IGLC2 PE=1 SV=1 | 38 | 48.1 | 1.6 | 0.7 | 0.7 | 177 | 62.3 | 1.9 | 0.8 | 1.1 | 83 | 41.5 | 1.0 | 1.1 | 0.9 | 100 | 62.3 | 0.8 | 1.0 | 1.0 | 1.041 |
| P02760 | Protein AMBP OS=Homo sapiens GN=AMBP PE=1 SV=1 | 10 | 11.1 | 1.8 | 1.4 | 0.5 | * | * | * | * | * | 3 | 3.4 | 0.8 | 0.6 | 0.9 | 4 | 3.4 | 1.4 | 1.0 | 0.8 | 1.039 |
| Q9UHX1 | Poly(U)-binding-splicing factor PUF60 OS=Homo sapiens GN=PUF60 PE=1 SV=1 | 4 | 11.4 | 0.3 | 0.4 | 0.8 | * | * | * | * | * | 6 | 11.4 | 1.2 | 2.1 | 0.9 | 4 | 5.9 | 1.5 | 1.0 | 1.4 | 1.037 |
| P08294 | Extracellular superoxide dismutase [Cu-Zn] OS=Homo sapiens GN=SOD3 PE=1 SV=2 | 6 | 16.7 | 1.2 | 1.1 | 1.0 | 11 | 12.1 | 0.6 | 0.8 | 1.0 | 6 | 6.7 | 1.2 | 1.5 | 0.9 | * | * | * | * | * | 1.037 |
| P02763 | Alpha-1-acid glycoprotein 1 OS=Homo sapiens GN=ORM1 PE=1 SV=1 | 36 | 22.4 | 1.4 | 0.8 | 0.7 | 40 | 16.4 | 1.7 | 0.6 | 0.9 | 22 | 7.5 | 1.4 | 1.3 | 1.2 | 34 | 19.9 | 0.7 | 0.8 | 0.9 | 1.035 |
| P04217 | Alpha-1B-glycoprotein OS=Homo sapiens GN=A1BG PE=1 SV=4 | 21 | 16.2 | 1.3 | 0.9 | 0.6 | 25 | 20.2 | 1.7 | 0.6 | 1.0 | 30 | 18.6 | 1.1 | 1.3 | 1.2 | 22 | 24 | 0.8 | 0.8 | 1.0 | 1.034 |
| P24752 | Acetyl-CoA acetyltransferase, mitochondrial OS=Homo sapiens GN=ACAT1 PE=1 SV=1 | 7 | 23.9 | 0.8 | 0.9 | 0.4 | 6 | 4.0 | 1.3 | 0.6 | 0.9 | 23 | 14.1 | 2.2 | 2.0 | 1.1 | 10 | 7 | 0.3 | 0.8 | 0.7 | 1.033 |
| Q14195 | Dihydropyrimidinase-related protein 3 OS=Homo sapiens GN=DPYSL3 PE=1 SV=1 | 28 | 31.6 | 1.3 | 0.9 | 0.5 | 23 | 35.6 | 0.7 | 1.3 | 1.0 | 24 | 21.4 | 1.2 | 1.3 | 1.6 | 32 | 23.9 | 0.6 | 1.1 | 0.7 | 1.031 |
| P61224 | Ras-related protein Rap-1b OS=Homo sapiens GN=RAP1B PE=1 SV=1 | 41 | 35.3 | 1.0 | 1.2 | 0.7 | 22 | 26.1 | 1.0 | 0.8 | 0.9 | 46 | 21.7 | 0.9 | 1.4 | 1.4 | 36 | 29.9 | 0.8 | 1.1 | 1.2 | 1.027 |
| Q99798 | Aconitate hydratase, mitochondrial OS=Homo sapiens GN=ACO2 PE=1 SV=2 | 24 | 20.4 | 0.9 | 1.2 | 0.7 | 7 | 7.6 | 0.8 | 0.9 | 1.0 | 43 | 21.2 | 1.3 | 1.8 | 1.2 | 25 | 16.5 | 0.6 | 0.9 | 0.6 | 1.021 |
| P08670 | Vimentin OS=Homo sapiens GN=VIM PE=1 SV=4 | 189 | 71.9 | 1.2 | 1.0 | 0.6 | 329 | 73.2 | 1.0 | 1.1 | 1.0 | 181 | 61.2 | 0.7 | 1.2 | 0.8 | 236 | 70.8 | 1.2 | 1.6 | 1.1 | 1.018 |
| P02743 | Serum amyloid P-component OS=Homo sapiens GN=APCS PE=1 SV=2 | 5 | 12.1 | 2.2 | 0.6 | 1.0 | 10 | 19.7 | 1.1 | 0.6 | 1.2 | * | * | * | * | * | 4 | 4.9 | 1.1 | 0.4 | 0.9 | 1.018 |
| P01023 | Alpha-2-macroglobulin OS=Homo sapiens GN=A2M PE=1 SV=3 | 107 | 24.2 | 0.9 | 1.0 | 0.9 | 103 | 23.1 | 1.7 | 0.9 | 1.0 | 55 | 15 | 1.0 | 1.1 | 1.2 | 83 | 19.1 | 0.9 | 0.7 | 0.8 | 1.017 |
| Q07960 | Rho GTPase-activating protein 1 OS=Homo sapiens GN=ARHGAP1 PE=1 SV=1 | 6 | 19.8 | 1.0 | 1.5 | 0.8 | 4 | 21.6 | 1.1 | 0.6 | 0.5 | 17 | 26.9 | 1.0 | 1.5 | 1.1 | 10 | 24.6 | 0.6 | 1.6 | 1.0 | 1.017 |
| P02647 | Apolipoprotein A-I OS=Homo sapiens GN=APOA1 PE=1 SV=1 | 257 | 76.8 | 1.4 | 0.7 | 0.6 | 459 | 72.3 | 1.2 | 0.7 | 1.0 | 174 | 65.5 | 1.5 | 1.1 | 1.2 | 202 | 68.9 | 0.8 | 0.8 | 1.0 | 1.011 |
| Q9BUF5 | Tubulin beta-6 chain OS=Homo sapiens GN=TUBB6 PE=1 SV=1 | 11 | 23.5 | 0.7 | 1.2 | 0.8 | 8 | 29.4 | 0.8 | 0.7 | 0.8 | 12 | 31.6 | 1.6 | 2.1 | 1.1 | 6 | 36.8 | 0.5 | 0.9 | 0.9 | 1.010 |
| P01859 | Ig gamma-2 chain C region OS=Homo sapiens GN=IGHG2 PE=1 SV=2 | 86 | 32.8 | 2.0 | 0.8 | 0.6 | 118 | 33.1 | 1.2 | 0.9 | 1.2 | 25 | 29.1 | 0.8 | 0.6 | 1.3 | 89 | 32.8 | 0.5 | 1.2 | 0.9 | 1.003 |
| P01591 | Immunoglobulin J chain OS=Homo sapiens GN=JCHAIN PE=1 SV=4 | 4 | 11.9 | 0.9 | 0.7 | 1.2 | 4 | 7.5 | 2.4 | 0.6 | 1.3 | * | * | * | * | * | 4 | 7.5 | 0.6 | 0.4 | 0.9 | 1.003 |
| P02787 | Serotransferrin OS=Homo sapiens GN=TF PE=1 SV=3;sp TRFE_HUMAN | * | * | * | * | * | 404 | 35.5 | 1.4 | 0.7 | 1.1 | 201 | 34.2 | 1.0 | 1.2 | 1.1 | 220 | 34.2 | 0.6 | 0.8 | 0.9 | 0.997 |
| P20674 | Cytochrome c oxidase subunit 5A, mitochondrial OS=Homo sapiens GN=COX5A PE=1 SV=2 | 13 | 60.0 | 0.7 | 0.9 | 0.4 | 2 | 44.0 | 0.7 | 0.7 | 0.8 | 14 | 58.7 | 1.9 | 1.5 | 1.5 | 8 | 30.7 | 0.6 | 1.2 | 0.8 | 0.991 |
| P0DMV8 | Heat shock 70 kDa protein 1A OS=Homo sapiens GN=HSPA1A PE=1 SV=1;Heat shock 70 kDa protein 1B OS=Homo sapiens GN=HSPA1B PE=1 SV=1 | 161 | 54.3 | 0.7 | 1.3 | 0.6 | 119 | 44.3 | 0.8 | 0.6 | 0.8 | 180 | 45.7 | 1.3 | 1.6 | 1.1 | 165 | 53.2 | 0.8 | 1.1 | 0.9 | 0.981 |
| P02774 | Vitamin D-binding protein OS=Homo sapiens GN=GC PE=1 SV=1 | 40 | 21.9 | 1.1 | 0.8 | 0.5 | 31 | 20.5 | 1.3 | 0.6 | 1.0 | 20 | 13.1 | 1.2 | 1.5 | 1.3 | 16 | 19 | 0.8 | 0.7 | 1.0 | 0.974 |
| P43034 | Platelet-activating factor acetylhydrolase IB subunit alpha OS=Homo sapiens GN=PAFAH1B1 PE=1 SV=2 | 2 | 5.4 | 1.0 | 1.5 | 1.7 | 2 | 3.7 | 0.2 | 0.6 | 0.8 | 2 | 3.7 | 0.4 | 1.2 | 1.4 | * | * | * | * | * | 0.974 |
| Q96DG6 | Carboxymethylenebutenolidase homolog OS=Homo sapiens GN=CMBL PE=1 SV=1 | 8 | 13.5 | 1.0 | 1.3 | 0.6 | * | * | * | * | * | 3 | 10.2 | 1.4 | 1.6 | 0.5 | 11 | 10.2 | 0.9 | 0.5 | 0.6 | 0.965 |
| P07305 | Histone H1.0 OS=Homo sapiens GN=H1F0 PE=1 SV=3 | 9 | 16.0 | 0.6 | 1.0 | 0.8 | 6 | 16.0 | 0.4 | 0.7 | 0.4 | 5 | 16 | 2.1 | 1.1 | 1.9 | 8 | 10.8 | 0.9 | 0.6 | 0.7 | 0.965 |
| P13645 | sp K1C10_HUMAN ;Keratin, type I cytoskeletal 10 OS=Homo sapiens GN=KRT10 PE=1 SV=6 | 30 | 32.2 | 0.9 | 0.8 | 0.8 | 61 | 42.3 | 1.0 | 0.7 | 1.3 | 20 | 23.3 | 0.7 | 0.9 | 1.1 | 45 | 36.3 | 0.9 | 1.4 | 1.6 | 0.963 |
| O75368 | SH3 domain-binding glutamic acid-rich-like protein OS=Homo sapiens GN=SH3BGRL PE=1 SV=1 | 8 | 23.7 | 0.9 | 1.1 | 0.8 | 10 | 47.4 | 0.3 | 0.5 | 0.4 | 7 | 38.6 | 1.7 | 1.0 | 1.7 | 5 | 38.6 | 1.1 | 1.1 | 1.1 | 0.962 |
| Q13228 | Selenium-binding protein 1 OS=Homo sapiens GN=SELENBP1 PE=1 SV=2 | 77 | 36.2 | 0.4 | 1.5 | 0.6 | 44 | 33.9 | 0.7 | 0.4 | 0.6 | 119 | 41.5 | 1.4 | 3.1 | 0.9 | 30 | 28 | 0.5 | 0.4 | 0.8 | 0.959 |
| P04196 | Histidine-rich glycoprotein OS=Homo sapiens GN=HRG PE=1 SV=1 | 5 | 10.3 | 1.1 | 1.2 | 0.5 | 13 | 14.5 | 1.1 | 0.4 | 0.9 | 7 | 7 | 1.4 | 1.3 | 1.0 | 5 | 7 | 0.6 | 1.0 | 0.9 | 0.955 |
| P10153 | Non-secretory ribonuclease OS=Homo sapiens GN=RNASE2 PE=1 SV=2 | 5 | 9.3 | 0.9 | 1.3 | 0.2 | 4 | 9.3 | 0.5 | 0.4 | 0.6 | 13 | 9.3 | 1.3 | 2.2 | 1.1 | 9 | 9.3 | 1.3 | 0.7 | 1.1 | 0.951 |
| Q15404 | Ras suppressor protein 1 OS=Homo sapiens GN=RSU1 PE=1 SV=3 | 5 | 33.9 | 0.4 | 0.5 | 0.3 | 10 | 17.0 | 0.7 | 1.2 | 1.0 | 3 | 18.4 | 1.1 | 1.9 | 2.1 | 19 | 44 | 0.7 | 0.6 | 0.5 | 0.950 |
| P30043 | Flavin reductase (NADPH) OS=Homo sapiens GN=BLVRB PE=1 SV=3 | 26 | 55.3 | 0.7 | 1.0 | 0.8 | 14 | 32.0 | 0.9 | 0.8 | 0.9 | 31 | 54.9 | 1.2 | 1.7 | 0.9 | 27 | 54.9 | 0.6 | 0.9 | 0.7 | 0.948 |
| P69891 | Hemoglobin subunit gamma-1 OS=Homo sapiens GN=HBG1 PE=1 SV=2;Hemoglobin subunit gamma-2 OS=Homo sapiens GN=HBG2 PE=1 SV=2 | 3 | 38.8 | 0.2 | 0.5 | 0.3 | * | * | * | * | * | 9 | 28.6 | 1.8 | 1.6 | 1.4 | 15 | 28.6 | 0.9 | 0.8 | 0.7 | 0.943 |
| P00738 | Haptoglobin OS=Homo sapiens GN=HP PE=1 SV=1 | 188 | 30.3 | 1.8 | 0.5 | 0.6 | 186 | 30.5 | 1.5 | 0.6 | 0.9 | 135 | 27.1 | 0.6 | 1.6 | 0.9 | 165 | 34.2 | 0.7 | 0.8 | 0.8 | 0.942 |
| P06727 | Apolipoprotein A-IV OS=Homo sapiens GN=APOA4 PE=1 SV=3 | 8 | 13.1 | 0.9 | 0.8 | 0.9 | 11 | 22.5 | 0.6 | 0.6 | 1.3 | * | * | * | * | * | 4 | 14.4 | 1.0 | 1.5 | 0.9 | 0.938 |
| O14949 | Cytochrome b-c1 complex subunit 8 OS=Homo sapiens GN=UQCRQ PE=1 SV=4 | 5 | 15.9 | 0.5 | 1.2 | 0.3 | 3 | 24.4 | 0.7 | 0.7 | 0.3 | 9 | 25.6 | 1.4 | 1.9 | 1.3 | * | * | * | * | * | 0.927 |
| Q8WWA0 | Intelectin-1 OS=Homo sapiens GN=ITLN1 PE=1 SV=1 | 3 | 16.0 | 0.5 | 0.4 | 2.8 | 4 | 6.1 | 0.1 | 0.2 | 0.2 | 4 | 6.1 | 1.0 | 2.2 | 0.8 | * | * | * | * | * | 0.910 |
| P09382 | Galectin-1 OS=Homo sapiens GN=LGALS1 PE=1 SV=2 | 29 | 38.5 | 1.4 | 0.8 | 0.5 | 27 | 48.9 | 0.3 | 0.9 | 0.6 | 15 | 38.5 | 0.8 | 1.2 | 1.3 | 31 | 48.9 | 0.7 | 1.3 | 0.8 | 0.893 |
| P01011 | Alpha-1-antichymotrypsin OS=Homo sapiens GN=SERPINA3 PE=1 SV=2 | 8 | 11.6 | 0.5 | 0.8 | 0.5 | 18 | 20.3 | 1.3 | 0.6 | 0.8 | * | * | * | * | * | 3 | 6.4 | 1.2 | 1.4 | 1.0 | 0.890 |
| P13073 | Cytochrome c oxidase subunit 4 isoform 1, mitochondrial OS=Homo sapiens GN=COX4I1 PE=1 SV=1 | 17 | 26.0 | 0.6 | 1.5 | 0.3 | 8 | 30.2 | 0.6 | 0.5 | 0.3 | 26 | 20.1 | 1.4 | 2.3 | 0.9 | 12 | 19.5 | 0.6 | 0.8 | 0.8 | 0.885 |
| P62140 | Serine/threonine-protein phosphatase PP1-beta catalytic subunit OS=Homo sapiens GN=PPP1CB PE=1 SV=3 | 3 | 15.9 | 0.7 | 1.2 | 0.4 | 10 | 12.5 | 0.7 | 0.5 | 0.9 | * | * | * | * | * | 18 | 16.8 | 1.4 | 1.3 | 1.0 | 0.881 |
| P01877 | Ig alpha-2 chain C region OS=Homo sapiens GN=IGHA2 PE=1 SV=3 | 13 | 25.0 | 0.8 | 1.3 | 1.2 | 9 | 19.1 | 1.1 | 0.4 | 0.7 | 10 | 19.1 | 1.1 | 1.4 | 0.8 | 5 | 19.1 | 0.8 | 0.2 | 0.9 | 0.881 |
| P40925 | Malate dehydrogenase, cytoplasmic OS=Homo sapiens GN=MDH1 PE=1 SV=4 | 14 | 19.5 | 0.6 | 1.3 | 0.4 | 18 | 18.6 | 0.7 | 0.7 | 0.8 | 16 | 25.7 | 1.1 | 1.5 | 0.9 | 18 | 29 | 0.5 | 1.0 | 0.5 | 0.874 |
| P02790 | Hemopexin OS=Homo sapiens GN=HPX PE=1 SV=2 | 45 | 25.8 | 1.2 | 1.0 | 0.6 | 57 | 13.9 | 1.4 | 0.5 | 0.7 | 14 | 7.4 | 0.8 | 1.1 | 1.0 | 16 | 12.8 | 0.5 | 0.7 | 0.8 | 0.871 |
| P08133 | Annexin A6 OS=Homo sapiens GN=ANXA6 PE=1 SV=3 | 83 | 40.9 | 0.8 | 0.9 | 0.4 | 114 | 46.1 | 0.7 | 0.9 | 0.7 | 91 | 34.5 | 1.2 | 1.3 | 1.1 | 93 | 40 | 0.5 | 0.9 | 0.7 | 0.863 |
| P02768 | sp ALBU_HUMAN ;Serum albumin OS=Homo sapiens GN=ALB PE=1 SV=2 | 1221 | 52.5 | 1.2 | 0.8 | 0.6 | 2537 | 46.0 | 1.2 | 0.7 | 0.8 | 1142 | 44.3 | 0.8 | 1.0 | 1.1 | 1105 | 44.5 | 0.5 | 0.7 | 0.8 | 0.851 |
| Q562R1 | Beta-actin-like protein 2 OS=Homo sapiens GN=ACTBL2 PE=1 SV=2 | 3 | 41.8 | 1.2 | 1.2 | 0.7 | 3 | 42.3 | 0.7 | 0.3 | 0.7 | 4 | 35.6 | 1.0 | 1.8 | 0.6 | 5 | 47.6 | 0.4 | 0.6 | 0.6 | 0.843 |
| P02652 | Apolipoprotein A-II OS=Homo sapiens GN=APOA2 PE=1 SV=1 | 13 | 64.0 | 1.0 | 0.7 | 0.7 | 28 | 44.0 | 1.0 | 0.8 | 1.1 | 10 | 44 | 0.7 | 1.0 | 0.7 | 19 | 44 | 0.9 | 0.7 | 0.9 | 0.837 |
| P43121 | Cell surface glycoprotein MUC18 OS=Homo sapiens GN=MCAM PE=1 SV=2 | 2 | 5.0 | 0.2 | 0.8 | 0.5 | * | * | * | * | * | 4 | 4.2 | 1.0 | 1.6 | 1.0 | 5 | 6.5 | 0.9 | 0.7 | 1.0 | 0.826 |
| Q9Y6R7 | IgGFc-binding protein OS=Homo sapiens GN=FCGBP PE=1 SV=3 | 62 | 9.0 | 0.8 | 1.2 | 1.6 | * | * | * | * | * | 5 | 2.1 | 0.7 | 0.8 | 0.8 | 2 | 1.6 | 0.5 | 0.4 | 0.5 | 0.823 |
| Q09666 | Neuroblast differentiation-associated protein AHNAK OS=Homo sapiens GN=AHNAK PE=1 SV=2 | 306 | 45.5 | 0.8 | 1.0 | 0.6 | 218 | 37.4 | 0.5 | 0.7 | 0.7 | 134 | 24.8 | 0.9 | 1.2 | 0.8 | 294 | 38.2 | 0.9 | 0.9 | 0.7 | 0.817 |
| Q99584 | Protein S100-A13 OS=Homo sapiens GN=S100A13 PE=1 SV=1 | * | * | * | * | * | 7 | 23.5 | 0.9 | 0.8 | 0.6 | 8 | 12.2 | 0.6 | 1.4 | 0.6 | 5 | 23.5 | 0.7 | 1.0 | 0.9 | 0.812 |
| Q02817 | Mucin-2 OS=Homo sapiens GN=MUC2 PE=1 SV=2 | 23 | 0.6 | 1.0 | 1.2 | 0.7 | 3 | 0.3 | 0.5 | 0.8 | 1.0 | 9 | 0.3 | 0.8 | 0.9 | 0.5 | 5 | 0.9 | 0.7 | 0.9 | 0.9 | 0.811 |
| Q9Y490 | Talin-1 OS=Homo sapiens GN=TLN1 PE=1 SV=3 | 176 | 33.3 | 0.6 | 0.9 | 0.4 | 275 | 35.4 | 0.7 | 0.8 | 0.8 | 251 | 29.1 | 1.0 | 1.2 | 1.0 | 229 | 30.8 | 0.6 | 0.7 | 0.7 | 0.803 |
| P02042 | Hemoglobin subunit delta OS=Homo sapiens GN=HBD PE=1 SV=2 | 48 | 85.0 | 0.7 | 0.7 | 0.6 | 58 | 80.3 | 0.6 | 0.5 | 0.5 | 64 | 80.3 | 1.4 | 1.6 | 1.1 | 27 | 78.2 | 0.5 | 0.7 | 0.7 | 0.803 |
| P06396 | sp GELS_HUMAN ;Gelsolin OS=Homo sapiens GN=GSN PE=1 SV=1 | 102 | 32.2 | 0.8 | 0.9 | 0.5 | 103 | 39.3 | 0.8 | 0.6 | 0.6 | 64 | 28.1 | 0.8 | 1.2 | 1.0 | 121 | 37.5 | 0.6 | 1.0 | 0.7 | 0.802 |
| Q9C002 | Normal mucosa of esophagus-specific gene 1 protein OS=Homo sapiens GN=NMES1 PE=2 SV=1 | 3 | 32.5 | 0.4 | 1.1 | 0.1 | 5 | 32.5 | 0.5 | 0.4 | 1.9 | 8 | 32.5 | 0.5 | 1.5 | 0.6 | * | * | * | * | * | 0.796 |
| P20231 | Tryptase beta-2 OS=Homo sapiens GN=TPSB2 PE=1 SV=2;Tryptase alpha/beta-1 OS=Homo sapiens GN=TPSAB1 PE=1 SV=1 | 4 | 9.8 | 0.5 | 0.5 | 0.1 | 9 | 14.9 | 0.7 | 1.1 | 1.5 | 5 | 9.8 | 1.1 | 0.7 | 1.0 | 19 | 14.9 | 0.7 | 0.9 | 0.8 | 0.792 |
| P36871 | Phosphoglucomutase-1 OS=Homo sapiens GN=PGM1 PE=1 SV=3 | 5 | 24.7 | 0.7 | 0.9 | 0.9 | 2 | 10.9 | 0.7 | 0.8 | 0.4 | 11 | 13.9 | 1.0 | 0.8 | 0.8 | 9 | 16 | 0.8 | 0.9 | 0.7 | 0.790 |
| Q04917 | 14-3-3 protein eta OS=Homo sapiens GN=YWHAH PE=1 SV=4 | 5 | 27.2 | 0.4 | 1.1 | 0.8 | 2 | 24.8 | 0.9 | 0.7 | 0.6 | * | * | * | * | * | 2 | 28.9 | 1.0 | 0.7 | 1.1 | 0.781 |
| P01614 | Ig kappa chain V-II region Cum OS=Homo sapiens PE=1 SV=1;Ig kappa chain V-II region TEW OS=Homo sapiens PE=1 SV=1;Ig kappa chain V-II region GM607 (Fragment) OS=Homo sapiens PE=4 SV=1;Ig kappa chain V-II region RPMI 6410 OS=Homo sapiens PE=4 SV=1 | 9 | 11.5 | 0.7 | 0.5 | 0.6 | 19 | 17.4 | 1.2 | 0.5 | 0.8 | 11 | 11.5 | 0.6 | 1.3 | 0.5 | 9 | 11.5 | 0.9 | 0.9 | 1.2 | 0.770 |
| O00159 | Unconventional myosin-Ic OS=Homo sapiens GN=MYO1C PE=1 SV=4 | 3 | 1.7 | 0.5 | 0.7 | 0.4 | 4 | 4.3 | 0.8 | 0.8 | 0.9 | 4 | 4.3 | 1.4 | 0.7 | 0.7 | 10 | 8.6 | 0.8 | 0.7 | 0.7 | 0.760 |
| Q9UGI8 | Testin OS=Homo sapiens GN=TES PE=1 SV=1 | * | * | * | * | * | 3 | 5.7 | 0.2 | 0.6 | 0.6 | 6 | 8.8 | 1.0 | 1.5 | 1.0 | 2 | 7.6 | 0.3 | 0.9 | 0.5 | 0.752 |
| P19652 | Alpha-1-acid glycoprotein 2 OS=Homo sapiens GN=ORM2 PE=1 SV=2 | 10 | 9.5 | 1.3 | 0.7 | 0.4 | 7 | 9.5 | 0.7 | 0.4 | 0.8 | 5 | 5 | 0.7 | 1.0 | 0.8 | 11 | 12.9 | 0.7 | 0.7 | 0.7 | 0.742 |
| Q96AC1 | Fermitin family homolog 2 OS=Homo sapiens GN=FERMT2 PE=1 SV=1 | 3 | 9.3 | 0.8 | 0.7 | 0.4 | 5 | 7.8 | 1.2 | 1.0 | 0.8 | * | * | * | * | * | 3 | 7.8 | 0.4 | 0.5 | 0.6 | 0.729 |
| Q9ULV4 | Coronin-1C OS=Homo sapiens GN=CORO1C PE=1 SV=1 | 18 | 10.3 | 1.0 | 1.3 | 0.5 | 16 | 12.4 | 0.8 | 0.6 | 0.7 | 8 | 3.4 | 0.7 | 0.6 | 0.6 | 20 | 6.1 | 0.5 | 0.6 | 0.7 | 0.716 |
| P01009 | Alpha-1-antitrypsin OS=Homo sapiens GN=SERPINA1 PE=1 SV=3 | 296 | 68.2 | 1.6 | 0.5 | 0.3 | 298 | 61.0 | 1.0 | 0.8 | 0.4 | 159 | 50.7 | 0.6 | 0.9 | 0.6 | 238 | 59.6 | 0.3 | 0.7 | 0.7 | 0.716 |
| P18206 | Vinculin OS=Homo sapiens GN=VCL PE=1 SV=4 | 349 | 48.4 | 0.6 | 0.7 | 0.5 | 359 | 54.7 | 0.5 | 0.7 | 0.7 | 284 | 41.4 | 0.8 | 0.9 | 1.0 | 289 | 47.9 | 0.6 | 0.7 | 0.6 | 0.704 |
| Q93052 | Lipoma-preferred partner OS=Homo sapiens GN=LPP PE=1 SV=1 | 11 | 10.8 | 0.5 | 0.8 | 0.4 | 29 | 16.3 | 0.6 | 0.8 | 0.8 | 18 | 20.3 | 0.6 | 1.0 | 0.8 | 15 | 18.8 | 0.5 | 0.6 | 0.6 | 0.680 |
| O15061 | Synemin OS=Homo sapiens GN=SYNM PE=1 SV=2 | 3 | 3.3 | 0.5 | 0.2 | 0.2 | 3 | 6.1 | 0.6 | 0.6 | 0.7 | * | * | * | * | * | 2 | 1.9 | 1.3 | 1.2 | 0.6 | 0.676 |
| P12814 | Alpha-actinin-1 OS=Homo sapiens GN=ACTN1 PE=1 SV=2 | 63 | 49.0 | 0.6 | 0.7 | 0.5 | 93 | 49.0 | 0.5 | 0.6 | 0.8 | 37 | 31.1 | 0.7 | 0.8 | 0.7 | 75 | 46.9 | 0.8 | 0.8 | 0.6 | 0.671 |
| Q13418 | Integrin-linked protein kinase OS=Homo sapiens GN=ILK PE=1 SV=2 | 6 | 10.0 | 0.6 | 0.4 | 0.3 | 11 | 17.9 | 0.6 | 0.9 | 0.5 | 12 | 13.7 | 0.8 | 1.3 | 0.9 | 18 | 10.6 | 0.5 | 0.7 | 0.5 | 0.671 |
| P69905 | sp HBA_HUMAN ;Hemoglobin subunit alpha OS=Homo sapiens GN=HBA1 PE=1 SV=2 | 1393 | 75.9 | 0.8 | 0.6 | 0.4 | 1280 | 78.0 | 0.4 | 0.4 | 0.3 | 1938 | 75.9 | 1.5 | 1.4 | 0.9 | 1286 | 78 | 0.3 | 0.4 | 0.6 | 0.655 |
| P68871 | sp HBB_HUMAN ;Hemoglobin subunit beta OS=Homo sapiens GN=HBB PE=1 SV=2 | 416 | 89.7 | 0.7 | 0.6 | 0.5 | 543 | 84.9 | 0.4 | 0.4 | 0.3 | 548 | 82.9 | 1.2 | 1.2 | 0.7 | 345 | 82.9 | 0.5 | 0.5 | 0.6 | 0.647 |
| P12109 | Collagen alpha-1(VI) chain OS=Homo sapiens GN=COL6A1 PE=1 SV=3 | 3 | 2.5 | 0.5 | 0.7 | 0.3 | 4 | 5.4 | 0.4 | 1.0 | 1.2 | 2 | 2.4 | 0.7 | 1.0 | 0.8 | 3 | 2.4 | 0.3 | 0.4 | 0.5 | 0.646 |
| P12111 | Collagen alpha-3(VI) chain OS=Homo sapiens GN=COL6A3 PE=1 SV=5 | 50 | 11.9 | 0.6 | 0.6 | 0.4 | 58 | 10.5 | 0.6 | 0.7 | 0.7 | 14 | 5.3 | 0.4 | 0.8 | 0.7 | 51 | 8.3 | 0.6 | 0.7 | 0.6 | 0.618 |
| Q05707 | Collagen alpha-1(XIV) chain OS=Homo sapiens GN=COL14A1 PE=1 SV=3 | 8 | 5.8 | 0.5 | 0.6 | 0.4 | 28 | 9.6 | 0.6 | 1.0 | 0.8 | * | * | * | * | * | 6 | 4 | 0.6 | 0.5 | 0.5 | 0.604 |
| P12277 | Creatine kinase B-type OS=Homo sapiens GN=CKB PE=1 SV=1 | 135 | 64.0 | 0.3 | 1.3 | 0.4 | 163 | 57.5 | 0.5 | 0.4 | 0.6 | 89 | 52.2 | 0.6 | 1.2 | 0.6 | 111 | 57.7 | 0.3 | 0.3 | 0.5 | 0.603 |
| P00325 | Alcohol dehydrogenase 1B OS=Homo sapiens GN=ADH1B PE=1 SV=2;Alcohol dehydrogenase 1A OS=Homo sapiens GN=ADH1A PE=1 SV=2 | * | * | * | * | * | 4 | 7.5 | 0.2 | 0.5 | 0.4 | 4 | 9.9 | 0.8 | 0.7 | 0.4 | 2 | 13.9 | 1.0 | 0.7 | 0.9 | 0.588 |
| Q53GG5 | PDZ and LIM domain protein 3 OS=Homo sapiens GN=PDLIM3 PE=1 SV=1 | 6 | 8.2 | 0.8 | 1.0 | 0.4 | 16 | 12.4 | 0.4 | 0.7 | 0.6 | 8 | 8.2 | 0.4 | 0.7 | 0.5 | 10 | 8.2 | 0.3 | 0.5 | 0.3 | 0.570 |
| Q6NZI2 | Polymerase I and transcript release factor OS=Homo sapiens GN=PTRF PE=1 SV=1 | 15 | 21.0 | 0.3 | 0.6 | 0.5 | 40 | 24.4 | 0.5 | 0.7 | 0.6 | 19 | 14.1 | 0.5 | 0.8 | 0.5 | 16 | 17.7 | 0.7 | 0.5 | 0.7 | 0.561 |
| P51884 | Lumican OS=Homo sapiens GN=LUM PE=1 SV=2 | 106 | 38.5 | 1.1 | 0.5 | 0.4 | 106 | 38.5 | 0.5 | 0.7 | 0.4 | 56 | 29.9 | 0.5 | 0.5 | 0.5 | 92 | 38.5 | 0.3 | 0.6 | 0.4 | 0.551 |
| Q05682 | Caldesmon OS=Homo sapiens GN=CALD1 PE=1 SV=3 | 61 | 25.6 | 0.6 | 0.5 | 0.3 | 75 | 32.4 | 0.4 | 0.6 | 0.6 | 32 | 19.7 | 0.6 | 0.8 | 0.8 | 55 | 29.4 | 0.4 | 0.5 | 0.4 | 0.538 |
| P00352 | Retinal dehydrogenase 1 OS=Homo sapiens GN=ALDH1A1 PE=1 SV=2 | 11 | 17.2 | 0.3 | 0.5 | 0.3 | 18 | 18.4 | 0.7 | 0.4 | 0.3 | 15 | 12 | 0.8 | 0.6 | 0.4 | 38 | 25.1 | 0.6 | 0.8 | 0.5 | 0.534 |
| Q14315 | Filamin-C OS=Homo sapiens GN=FLNC PE=1 SV=3 | 17 | 9.0 | 0.4 | 0.6 | 0.4 | 53 | 16.7 | 0.5 | 0.7 | 0.6 | 12 | 7.1 | 0.5 | 0.8 | 0.7 | 21 | 12 | 0.4 | 0.3 | 0.4 | 0.533 |
| Q9NZN4 | EH domain-containing protein 2 OS=Homo sapiens GN=EHD2 PE=1 SV=2 | 34 | 20.8 | 0.2 | 0.4 | 0.2 | 52 | 30.8 | 0.5 | 0.5 | 0.5 | 35 | 18.8 | 0.6 | 0.9 | 0.8 | 35 | 20.6 | 0.5 | 0.5 | 0.6 | 0.529 |
| P00915 | sp CAH1_HUMAN ;Carbonic anhydrase 1 OS=Homo sapiens GN=CA1 PE=1 SV=2 | 149 | 59.6 | 0.4 | 0.6 | 0.5 | 72 | 59.6 | 0.4 | 0.3 | 0.3 | 139 | 53.5 | 0.8 | 1.0 | 0.7 | 101 | 53.5 | 0.5 | 0.4 | 0.5 | 0.527 |
| Q8WX93 | Palladin OS=Homo sapiens GN=PALLD PE=1 SV=3 | 14 | 6.2 | 0.6 | 0.7 | 0.3 | 31 | 7.7 | 0.4 | 0.6 | 0.5 | 20 | 5.6 | 0.4 | 0.6 | 0.6 | 13 | 4.3 | 0.5 | 0.6 | 0.4 | 0.525 |
| O75310 | UDP-glucuronosyltransferase 2B11 OS=Homo sapiens GN=UGT2B11 PE=2 SV=1;UDP-glucuronosyltransferase 2B17 OS=Homo sapiens GN=UGT2B17 PE=1 SV=1;UDP-glucuronosyltransferase 2B4 OS=Homo sapiens GN=UGT2B4 PE=1 SV=2;UDP-glucuronosyltransferase 2B7 OS=Homo sapiens GN=UGT2B7 PE=1 SV=1;UDP-glucuronosyltransferase 2B10 OS=Homo sapiens GN=UGT2B10 PE=1 SV=1;UDP-glucuronosyltransferase 2B15 OS=Homo sapiens GN=UGT2B15 PE=1 SV=3 | 4 | 2.3 | 0.3 | 0.4 | 0.3 | * | * | * | * | * | 2 | 2.3 | 0.6 | 1.1 | 0.6 | 2 | 2.3 | 0.5 | 0.3 | 0.4 | 0.515 |
| Q9BX66 | Sorbin and SH3 domain-containing protein 1 OS=Homo sapiens GN=SORBS1 PE=1 SV=3 | 7 | 2.9 | 0.2 | 0.4 | 0.3 | 15 | 10.7 | 0.4 | 0.7 | 0.7 | 4 | 2 | 0.8 | 0.4 | 0.8 | * | * | * | * | * | 0.514 |
| Q16853 | Membrane primary amine oxidase OS=Homo sapiens GN=AOC3 PE=1 SV=3 | 16 | 12.7 | 0.3 | 0.6 | 0.4 | 40 | 15.1 | 0.4 | 0.5 | 0.6 | 20 | 11.4 | 0.7 | 0.7 | 0.9 | 40 | 14 | 0.3 | 0.3 | 0.4 | 0.512 |
| P07585 | Decorin OS=Homo sapiens GN=DCN PE=1 SV=1 | 35 | 35.1 | 0.7 | 0.5 | 0.4 | 24 | 29.0 | 0.4 | 0.7 | 0.4 | 14 | 21.4 | 0.4 | 0.5 | 0.4 | 29 | 30.1 | 0.5 | 0.7 | 0.6 | 0.501 |
| P60981 | Destrin OS=Homo sapiens GN=DSTN PE=1 SV=3 | 6 | 10.9 | 0.2 | 0.5 | 0.4 | 8 | 16.4 | 0.3 | 0.5 | 0.5 | * | * | * | * | * | 10 | 21.2 | 0.7 | 0.7 | 0.8 | 0.495 |
| P04792 | Heat shock protein beta-1 OS=Homo sapiens GN=HSPB1 PE=1 SV=2 | 176 | 83.4 | 0.3 | 0.8 | 0.3 | 195 | 87.3 | 0.4 | 0.6 | 0.6 | 125 | 85.4 | 0.5 | 0.5 | 0.8 | 133 | 83.4 | 0.4 | 0.5 | 0.4 | 0.492 |
| P51888 | Prolargin OS=Homo sapiens GN=PRELP PE=1 SV=1 | 42 | 22.0 | 1.1 | 0.5 | 0.4 | 27 | 19.1 | 0.5 | 0.6 | 0.6 | 11 | 13.6 | 0.3 | 0.3 | 0.4 | 24 | 19.6 | 0.3 | 0.4 | 0.4 | 0.477 |
| P21333 | Filamin-A OS=Homo sapiens GN=FLNA PE=1 SV=4 | 773 | 45.8 | 0.4 | 0.4 | 0.3 | 1202 | 47.9 | 0.4 | 0.6 | 0.5 | 729 | 45.7 | 0.5 | 0.6 | 0.7 | 822 | 47.2 | 0.4 | 0.5 | 0.4 | 0.463 |
| P00918 | sp CAH2_HUMAN ;Carbonic anhydrase 2 OS=Homo sapiens GN=CA2 PE=1 SV=2 | 65 | 34.0 | 0.4 | 0.7 | 0.4 | 24 | 25.5 | 0.3 | 0.3 | 0.4 | 51 | 25.1 | 0.5 | 0.8 | 0.5 | 61 | 26.6 | 0.3 | 0.3 | 0.3 | 0.456 |
| P04271 | Protein S100-B OS=Homo sapiens GN=S100B PE=1 SV=2 | 2 | 16.3 | 0.4 | 0.2 | 0.3 | 4 | 16.3 | 0.5 | 0.5 | 0.5 | 2 | 16.3 | 0.5 | 0.4 | 0.5 | 4 | 16.3 | 0.3 | 0.8 | 0.4 | 0.444 |
| Q15746 | Myosin light chain kinase, smooth muscle OS=Homo sapiens GN=MYLK PE=1 SV=4 | 25 | 4.5 | 0.3 | 0.4 | 0.3 | 38 | 9.5 | 0.3 | 0.5 | 0.5 | 15 | 4.8 | 0.5 | 0.6 | 0.7 | 25 | 5.9 | 0.3 | 0.2 | 0.3 | 0.426 |
| P21291 | Cysteine and glycine-rich protein 1 OS=Homo sapiens GN=CSRP1 PE=1 SV=3 | 70 | 28.0 | 0.3 | 0.4 | 0.2 | 86 | 28.0 | 0.3 | 0.6 | 0.4 | 53 | 28 | 0.4 | 0.6 | 0.6 | 74 | 28 | 0.3 | 0.3 | 0.4 | 0.411 |
| Q9NR12 | PDZ and LIM domain protein 7 OS=Homo sapiens GN=PDLIM7 PE=1 SV=1 | 14 | 8.3 | 0.2 | 0.3 | 0.2 | 37 | 12.7 | 0.3 | 0.6 | 0.4 | 18 | 5.9 | 0.5 | 0.7 | 0.7 | 17 | 12.9 | 0.2 | 0.3 | 0.3 | 0.408 |
| O14558 | Heat shock protein beta-6 OS=Homo sapiens GN=HSPB6 PE=1 SV=2 | 9 | 68.8 | 0.2 | 0.3 | 0.2 | 16 | 60.6 | 0.3 | 0.3 | 0.4 | 8 | 38.8 | 0.7 | 0.8 | 0.6 | 14 | 68.8 | 0.3 | 0.3 | 0.3 | 0.402 |
| Q15124 | Phosphoglucomutase-like protein 5 OS=Homo sapiens GN=PGM5 PE=1 SV=2 | 7 | 12.2 | 0.3 | 0.5 | 0.3 | 14 | 15.5 | 0.3 | 0.7 | 0.5 | 7 | 10.1 | 0.3 | 0.3 | 0.6 | 10 | 12.2 | 0.2 | 0.3 | 0.2 | 0.391 |
| Q13642 | Four and a half LIM domains protein 1 OS=Homo sapiens GN=FHL1 PE=1 SV=4 | 2 | 7.1 | 0.4 | 0.3 | 0.3 | 15 | 9.6 | 0.3 | 0.5 | 0.4 | 6 | 9.6 | 0.3 | 0.5 | 0.6 | 5 | 9.6 | 0.4 | 0.3 | 0.3 | 0.381 |
| Q03135 | Caveolin-1 OS=Homo sapiens GN=CAV1 PE=1 SV=4 | 9 | 16.9 | 0.6 | 0.8 | 0.3 | 12 | 21.9 | 0.2 | 0.6 | 0.3 | 4 | 16.9 | 0.3 | 0.3 | 0.2 | 7 | 16.9 | 0.2 | 0.2 | 0.3 | 0.367 |
| Q9UMS6 | Synaptopodin-2 OS=Homo sapiens GN=SYNPO2 PE=1 SV=2 | 4 | 7.1 | 0.3 | 0.3 | 0.4 | 5 | 7.0 | 0.3 | 0.4 | 0.8 | 7 | 3.2 | 0.3 | 0.3 | 0.2 | 6 | 6.5 | 0.3 | 0.1 | 0.3 | 0.347 |
| P17661 | Desmin OS=Homo sapiens GN=DES PE=1 SV=3 | 30 | 52.6 | 0.1 | 0.2 | 0.2 | 101 | 53.8 | 0.4 | 0.5 | 0.5 | 32 | 45.1 | 0.4 | 0.4 | 0.3 | 40 | 42.8 | 0.4 | 0.2 | 0.3 | 0.332 |
| P60660 | Myosin light polypeptide 6 OS=Homo sapiens GN=MYL6 PE=1 SV=2 | 158 | 51.0 | 0.2 | 0.4 | 0.1 | 220 | 55.6 | 0.2 | 0.4 | 0.3 | 189 | 45 | 0.4 | 0.6 | 0.4 | 175 | 52.3 | 0.2 | 0.3 | 0.2 | 0.317 |
| P09493 | Tropomyosin alpha-1 chain OS=Homo sapiens GN=TPM1 PE=1 SV=2 | 46 | 52.8 | 0.2 | 0.3 | 0.2 | 70 | 58.5 | 0.2 | 0.4 | 0.2 | 24 | 39.1 | 0.3 | 0.7 | 0.3 | 42 | 45.8 | 0.2 | 0.2 | 0.2 | 0.287 |
| P35749 | Myosin-11 OS=Homo sapiens GN=MYH11 PE=1 SV=3 | 490 | 51.4 | 0.1 | 0.3 | 0.2 | 810 | 52.5 | 0.3 | 0.5 | 0.4 | 348 | 47.4 | 0.3 | 0.4 | 0.4 | 452 | 48.9 | 0.2 | 0.2 | 0.2 | 0.279 |
| Q01995 | Transgelin OS=Homo sapiens GN=TAGLN PE=1 SV=4 | 393 | 79.1 | 0.3 | 0.2 | 0.1 | 857 | 76.1 | 0.2 | 0.6 | 0.3 | 488 | 78.6 | 0.2 | 0.3 | 0.5 | 350 | 75.6 | 0.1 | 0.2 | 0.2 | 0.259 |
| P51911 | Calponin-1 OS=Homo sapiens GN=CNN1 PE=1 SV=2 | 116 | 53.2 | 0.2 | 0.2 | 0.2 | 238 | 53.5 | 0.2 | 0.4 | 0.3 | 136 | 53.9 | 0.2 | 0.3 | 0.4 | 103 | 50.8 | 0.2 | 0.1 | 0.2 | 0.253 |
| P07951 | Tropomyosin beta chain OS=Homo sapiens GN=TPM2 PE=1 SV=1 | 24 | 51.4 | 0.1 | 0.2 | 0.1 | 51 | 56.0 | 0.1 | 0.4 | 0.4 | 7 | 43.3 | 0.2 | 0.4 | 0.3 | 28 | 48.9 | 0.2 | 0.1 | 0.2 | 0.227 |
| P24844 | Myosin regulatory light polypeptide 9 OS=Homo sapiens GN=MYL9 PE=1 SV=4 | 20 | 48.3 | 0.2 | 0.3 | 0.2 | 28 | 61.6 | 0.2 | 0.4 | 0.2 | 10 | 54.7 | 0.2 | 0.3 | 0.3 | 15 | 48.3 | 0.2 | 0.2 | 0.2 | 0.224 |
| P63267 | Actin, gamma-enteric smooth muscle OS=Homo sapiens GN=ACTG2 PE=1 SV=1 | 6 | 80.9 | 0.1 | 0.2 | 0.2 | 9 | 80.1 | 0.2 | 0.3 | 0.2 | * | * | * | * | * | 19 | 77.7 | 0.1 | 0.1 | 0.1 | 0.190 |
