# Supplementary material for: Quantitative mass spectrometry analysis reveals a panel of nine proteins as diagnostic markers for colon adenocarcinomas
Source: Oncotarget. 2018 Feb 5;9(17):13530–44. doi: 10.18632/oncotarget.24418 (PMC5862596; doi:10.18632/oncotarget.24418)
Supplement: Supplementary file 4 [file oncotarget-09-13530-s004.docx]

| **Supplementary Table 1C: List of significantly differentially expressed proteins identified from iTRAQ analysis using Spectrum Mill and Trans Proteome Pipeline (Common between the two)** | | | | | | | | |
| --- | --- | --- | --- | --- | --- | --- | --- | --- |
| accession_number | entry_name | protein_mw | TPP_Average Fold change (n=11) | Number of patients showing upregulation | Number of patients showing downregulation | SpectrumMill_Average Fold change (n=11) | Number of patients showing upregulation | Number of patients showing downregulation |
| O14558 | Heat shock protein beta-6 OS=Homo sapiens GN=HSPB6 PE=1 SV=2 | 17624.8 | 0.4 | 0 | 9 | 0.4 | 0 | 8 |
| O14818 | Proteasome subunit alpha type-7 OS=Homo sapiens GN=PSMA7 PE=1 SV=1 | 31228.9 | 1.7 | 7 | 0 | 1.8 | 9 | 0 |
| O14980 | Exportin-1 OS=Homo sapiens GN=XPO1 PE=1 SV=1 | 133462.3 | 1.8 | 7 | 0 | 2.2 | 8 | 0 |
| O60506 | Heterogeneous nuclear ribonucleoprotein Q OS=Homo sapiens GN=SYNCRIP PE=1 SV=2 | 76460.7 | 1.9 | 7 | 0 | 1.7 | 7 | 0 |
| O95336 | 6-phosphogluconolactonase OS=Homo sapiens GN=PGLS PE=1 SV=2 | 28984.9 | 1.6 | 8 | 0 | 1.8 | 7 | 0 |
| P00338 | L-lactate dehydrogenase A chain OS=Homo sapiens GN=LDHA PE=1 SV=2 | 41009.6 | 1.9 | 7 | 0 | 1.9 | 8 | 0 |
| P00558 | Phosphoglycerate kinase 1 OS=Homo sapiens GN=PGK1 PE=1 SV=3 | 51067.8 | 1.8 | 8 | 0 | 2.0 | 9 | 0 |
| P00915 | sp CAH1_HUMAN ;Carbonic anhydrase 1 OS=Homo sapiens GN=CA1 PE=1 SV=2 | 31521.5 | 0.5 | 0 | 9 | 0.7 | 0 | 7 |
| P00918 | sp CAH2_HUMAN ;Carbonic anhydrase 2 OS=Homo sapiens GN=CA2 PE=1 SV=2 | 32762.2 | 0.5 | 0 | 10 | 0.5 | 0 | 9 |
| P04080 | Cystatin-B OS=Homo sapiens GN=CSTB PE=1 SV=2 | 12493.8 | 2.1 | 10 | 0 | 2.2 | 10 | 0 |
| P04792 | Heat shock protein beta-1 OS=Homo sapiens GN=HSPB1 PE=1 SV=2 | 23848.2 | 0.5 | 0 | 9 | 0.6 | 0 | 9 |
| P04843 | Dolichyl-diphosphooligosaccharide--protein glycosyltransferase subunit 1 OS=Homo sapiens GN=RPN1 PE=1 SV=1 | 74448.4 | 1.9 | 7 | 0 | 2.0 | 8 | 0 |
| P05109 | Protein S100-A8 OS=Homo sapiens GN=S100A8 PE=1 SV=1 | 12621.2 | 3.2 | 9 | 0 | 2.8 | 9 | 0 |
| P05164 | Myeloperoxidase OS=Homo sapiens GN=MPO PE=1 SV=1 | 88008.4 | 2.2 | 8 | 0 | 2.3 | 9 | 0 |
| P06702 | Protein S100-A9 OS=Homo sapiens GN=S100A9 PE=1 SV=1 | 14884.5 | 3.2 | 9 | 0 | 3.0 | 10 | 0 |
| P06731 | Carcinoembryonic antigen-related cell adhesion molecule 5 OS=Homo sapiens GN=CEACAM5 PE=1 SV=3 | 79985.6 | 2.6 | 10 | 0 | 2.3 | 7 | 1 |
| P06748 | Nucleophosmin OS=Homo sapiens GN=NPM1 PE=1 SV=2 | 37502.7 | 1.6 | 7 | 0 | 2.1 | 10 | 0 |
| P07108 | Acyl-CoA-binding protein OS=Homo sapiens GN=DBI PE=1 SV=2 | 11918.2 | 1.8 | 7 | 0 | 2.0 | 8 | 0 |
| P07339 | Cathepsin D OS=Homo sapiens GN=CTSD PE=1 SV=1 | 48380.4 | 1.9 | 7 | 0 | 2.0 | 7 | 0 |
| P07585 | Decorin OS=Homo sapiens GN=DCN PE=1 SV=1 | 43980.5 | 0.5 | 0 | 10 | 0.5 | 0 | 8 |
| P07951 | Tropomyosin beta chain OS=Homo sapiens GN=TPM2 PE=1 SV=1 | 38586.3 | 0.2 | 0 | 11 | 0.3 | 0 | 8 |
| P08195 | 4F2 cell-surface antigen heavy chain OS=Homo sapiens GN=SLC3A2 PE=1 SV=3 | 72401.4 | 3.0 | 10 | 0 | 2.8 | 8 | 0 |
| P09429 | High mobility group protein B1 OS=Homo sapiens GN=HMGB1 PE=1 SV=3 | 31263.1 | 2.2 | 9 | 0 | 2.0 | 8 | 0 |
| P09525 | Annexin A4 OS=Homo sapiens GN=ANXA4 PE=1 SV=4 | 39281.7 | 2.0 | 8 | 0 | 2.0 | 8 | 0 |
| P10599 | Thioredoxin OS=Homo sapiens GN=TXN PE=1 SV=3;sp THIO_HUMAN | 13752.4 | 2.0 | 8 | 1 | 1.8 | 7 | 0 |
| P11940 | Polyadenylate-binding protein 1 OS=Homo sapiens GN=PABPC1 PE=1 SV=2 | 76808.2 | 2.2 | 8 | 0 | 2.4 | 8 | 0 |
| P12277 | Creatine kinase B-type OS=Homo sapiens GN=CKB PE=1 SV=1 | 45956 | 0.6 | 0 | 9 | 0.7 | 0 | 9 |
| P12956 | X-ray repair cross-complementing protein 6 OS=Homo sapiens GN=XRCC6 PE=1 SV=2 | 78632.1 | 1.8 | 8 | 0 | 2.0 | 7 | 0 |
| P13639 | Elongation factor 2 OS=Homo sapiens GN=EEF2 PE=1 SV=4 | 105676.4 | 1.7 | 7 | 0 | 1.8 | 8 | 0 |
| P14314 | Glucosidase 2 subunit beta OS=Homo sapiens GN=PRKCSH PE=1 SV=2 | 65151.4 | 2.0 | 8 | 0 | 2.0 | 7 | 0 |
| P14618 | Pyruvate kinase PKM OS=Homo sapiens GN=PKM PE=1 SV=4 | 63840.1 | 1.7 | 8 | 0 | 1.6 | 7 | 0 |
| P18124 | 60S ribosomal protein L7 OS=Homo sapiens GN=RPL7 PE=1 SV=1 | 34183.5 | 1.6 | 7 | 0 | 1.7 | 9 | 0 |
| P19338 | Nucleolin OS=Homo sapiens GN=NCL PE=1 SV=3 | 89643.9 | 2.3 | 9 | 0 | 2.5 | 9 | 0 |
| P20290 | Transcription factor BTF3 OS=Homo sapiens GN=BTF3 PE=1 SV=1 | 24531.1 | 2.1 | 8 | 0 | 2.4 | 7 | 0 |
| P20618 | Proteasome subunit beta type-1 OS=Homo sapiens GN=PSMB1 PE=1 SV=2 | 28591.1 | 1.5 | 7 | 0 | 1.9 | 10 | 0 |
| P21291 | Cysteine and glycine-rich protein 1 OS=Homo sapiens GN=CSRP1 PE=1 SV=3 | 24738.4 | 0.4 | 0 | 11 | 0.4 | 0 | 10 |
| P21333 | Filamin-A OS=Homo sapiens GN=FLNA PE=1 SV=4 | 306537.1 | 0.5 | 0 | 11 | 0.6 | 0 | 9 |
| P21796 | Voltage-dependent anion-selective channel protein 1 OS=Homo sapiens GN=VDAC1 PE=1 SV=2 | 34490 | 2.1 | 8 | 0 | 2.2 | 8 | 0 |
| P23284 | Peptidyl-prolyl cis-trans isomerase B OS=Homo sapiens GN=PPIB PE=1 SV=2 | 27547.2 | 2.1 | 9 | 0 | 1.9 | 9 | 0 |
| P24844 | Myosin regulatory light polypeptide 9 OS=Homo sapiens GN=MYL9 PE=1 SV=4 | 21902.1 | 0.2 | 0 | 11 | 0.4 | 0 | 8 |
| P26599 | Polypyrimidine tract-binding protein 1 OS=Homo sapiens GN=PTBP1 PE=1 SV=1 | 62148.5 | 1.6 | 8 | 0 | 1.6 | 9 | 0 |
| P26641 | Elongation factor 1-gamma OS=Homo sapiens GN=EEF1G PE=1 SV=3 | 55073.1 | 2.3 | 9 | 0 | 2.5 | 9 | 0 |
| P28838 | Cytosol aminopeptidase OS=Homo sapiens GN=LAP3 PE=1 SV=3 | 61754.3 | 1.8 | 8 | 0 | 1.7 | 8 | 0 |
| P29401 | Transketolase OS=Homo sapiens GN=TKT PE=1 SV=3 | 74615.5 | 1.7 | 8 | 0 | 2.0 | 8 | 0 |
| P31946 | 14-3-3 protein beta/alpha OS=Homo sapiens GN=YWHAB PE=1 SV=3 | 31079.1 | 1.8 | 8 | 0 | 1.7 | 7 | 0 |
| P31948 | Stress-induced-phosphoprotein 1 OS=Homo sapiens GN=STIP1 PE=1 SV=1 | 72347.5 | 2.0 | 7 | 1 | 2.7 | 7 | 0 |
| P31949 | Protein S100-A11 OS=Homo sapiens GN=S100A11 PE=1 SV=2 | 13295.9 | 2.1 | 9 | 0 | 2.3 | 10 | 0 |
| P35749 | Myosin-11 OS=Homo sapiens GN=MYH11 PE=1 SV=3 | 257598.5 | 0.3 | 0 | 11 | 0.5 | 0 | 10 |
| P37837 | Transaldolase OS=Homo sapiens GN=TALDO1 PE=1 SV=2 | 42035.2 | 1.9 | 7 | 0 | 2.2 | 7 | 0 |
| P38646 | Stress-70 protein, mitochondrial OS=Homo sapiens GN=HSPA9 PE=1 SV=2 | 81460.5 | 1.8 | 8 | 0 | 1.9 | 8 | 0 |
| P39019 | 40S ribosomal protein S19 OS=Homo sapiens GN=RPS19 PE=1 SV=2 | 18222.5 | 1.9 | 7 | 0 | 2.4 | 7 | 0 |
| P39687 | Acidic leucine-rich nuclear phosphoprotein 32 family member A OS=Homo sapiens GN=ANP32A PE=1 SV=1 | 31005.5 | 1.7 | 7 | 1 | 2.0 | 9 | 0 |
| P40121 | Macrophage-capping protein OS=Homo sapiens GN=CAPG PE=1 SV=2 | 42242.8 | 1.8 | 9 | 0 | 1.9 | 8 | 0 |
| P40926 | Malate dehydrogenase, mitochondrial OS=Homo sapiens GN=MDH2 PE=1 SV=3 | 39707.1 | 2.1 | 8 | 0 | 2.1 | 8 | 0 |
| P43243 | Matrin-3 OS=Homo sapiens GN=MATR3 PE=1 SV=2 | 104649.2 | 1.9 | 8 | 0 | 2.2 | 10 | 0 |
| P46777 | 60S ribosomal protein L5 OS=Homo sapiens GN=RPL5 PE=1 SV=3 | 39635.6 | 2.0 | 7 | 0 | 1.6 | 7 | 0 |
| P46781 | 40S ribosomal protein S9 OS=Homo sapiens GN=RPS9 PE=1 SV=3 | 25387 | 2.4 | 7 | 0 | 2.2 | 7 | 1 |
| P48643 | T-complex protein 1 subunit epsilon OS=Homo sapiens GN=CCT5 PE=1 SV=1 | 66325.1 | 1.8 | 7 | 0 | 2.0 | 9 | 0 |
| P49327 | Fatty acid synthase OS=Homo sapiens GN=FASN PE=1 SV=3 | 288299.2 | 2.0 | 8 | 0 | 2.1 | 8 | 0 |
| P50454 | Serpin H1 OS=Homo sapiens GN=SERPINH1 PE=1 SV=2 | 51455.1 | 1.9 | 9 | 0 | 2.2 | 10 | 0 |
| P51858 | Hepatoma-derived growth factor OS=Homo sapiens GN=HDGF PE=1 SV=1 | 30794.1 | 1.8 | 9 | 0 | 1.9 | 10 | 0 |
| P51884 | Lumican OS=Homo sapiens GN=LUM PE=1 SV=2 | 42374.5 | 0.6 | 0 | 9 | 0.6 | 0 | 8 |
| P51888 | Prolargin OS=Homo sapiens GN=PRELP PE=1 SV=1 | 46226.6 | 0.5 | 0 | 10 | 0.5 | 0 | 9 |
| P51911 | Calponin-1 OS=Homo sapiens GN=CNN1 PE=1 SV=2 | 36656.6 | 0.3 | 0 | 11 | 0.3 | 0 | 11 |
| P52272 | Heterogeneous nuclear ribonucleoprotein M OS=Homo sapiens GN=HNRNPM PE=1 SV=3 | 83565.8 | 1.5 | 7 | 0 | 1.6 | 7 | 0 |
| P53999 | Activated RNA polymerase II transcriptional coactivator p15 OS=Homo sapiens GN=SUB1 PE=1 SV=3 | 17134 | 2.1 | 9 | 0 | 2.4 | 8 | 0 |
| P55072 | Transitional endoplasmic reticulum ATPase OS=Homo sapiens GN=VCP PE=1 SV=4 | 96780.1 | 1.8 | 7 | 0 | 1.8 | 8 | 0 |
| P60174 | Triosephosphate isomerase OS=Homo sapiens GN=TPI1 PE=1 SV=3 | 33958.8 | 1.8 | 7 | 0 | 1.8 | 8 | 0 |
| P60842 | Eukaryotic initiation factor 4A-I OS=Homo sapiens GN=EIF4A1 PE=1 SV=1 | 49264.4 | 1.8 | 8 | 0 | 2.0 | 8 | 0 |
| P61247 | 40S ribosomal protein S3a OS=Homo sapiens GN=RPS3A PE=1 SV=2 | 35650.5 | 1.9 | 8 | 0 | 2.0 | 9 | 0 |
| P61626 | sp LYSC_HUMAN ;Lysozyme C OS=Homo sapiens GN=LYZ PE=1 SV=1 | 17858.1 | 2.8 | 8 | 0 | 2.8 | 7 | 0 |
| P62241 | 40S ribosomal protein S8 OS=Homo sapiens GN=RPS8 PE=1 SV=2 | 28958.8 | 2.1 | 8 | 0 | 2.3 | 8 | 0 |
| P62277 | 40S ribosomal protein S13 OS=Homo sapiens GN=RPS13 PE=1 SV=2 | 19816.8 | 2.0 | 8 | 0 | 1.9 | 8 | 0 |
| P62937 | Peptidyl-prolyl cis-trans isomerase A OS=Homo sapiens GN=PPIA PE=1 SV=2;sp PPIA_HUMAN | 20258.5 | 1.9 | 7 | 0 | 1.8 | 7 | 0 |
| P68431 | Histone H3.1 OS=Homo sapiens GN=HIST1H3A PE=1 SV=2;Histone H3.3 OS=Homo sapiens GN=H3F3A PE=1 SV=2;Histone H3.1t OS=Homo sapiens GN=HIST3H3 PE=1 SV=3;Histone H3.2 OS=Homo sapiens GN=HIST2H3A PE=1 SV=3 | 17391.9 | 2.9 | 8 | 0 | 2.5 | 10 | 0 |
| P78417 | Glutathione S-transferase omega-1 OS=Homo sapiens GN=GSTO1 PE=1 SV=2 | 31166.2 | 1.6 | 7 | 0 | 2.1 | 8 | 0 |
| P78527 | DNA-dependent protein kinase catalytic subunit OS=Homo sapiens GN=PRKDC PE=1 SV=3 | 513830.6 | 2.0 | 10 | 0 | 2.9 | 7 | 0 |
| Q01105 | Protein SET OS=Homo sapiens GN=SET PE=1 SV=3 | 36803.8 | 2.3 | 9 | 0 | 4.3 | 7 | 0 |
| Q01995 | Transgelin OS=Homo sapiens GN=TAGLN PE=1 SV=4 | 25118.2 | 0.3 | 0 | 11 | 0.3 | 0 | 11 |
| Q02878 | 60S ribosomal protein L6 OS=Homo sapiens GN=RPL6 PE=1 SV=3 | 40280.4 | 1.8 | 7 | 0 | 2.1 | 9 | 0 |
| Q03135 | Caveolin-1 OS=Homo sapiens GN=CAV1 PE=1 SV=4 | 22372.2 | 0.4 | 0 | 10 | 0.4 | 0 | 8 |
| Q04837 | Single-stranded DNA-binding protein, mitochondrial OS=Homo sapiens GN=SSBP1 PE=1 SV=1 | 18412.6 | 1.7 | 7 | 0 | 2.0 | 9 | 0 |
| Q05682 | Caldesmon OS=Homo sapiens GN=CALD1 PE=1 SV=3 | 108278.5 | 0.5 | 0 | 9 | 0.7 | 0 | 7 |
| Q07955 | Serine/arginine-rich splicing factor 1 OS=Homo sapiens GN=SRSF1 PE=1 SV=2 | 29011.4 | 1.9 | 8 | 0 | 1.8 | 9 | 0 |
| Q12905 | Interleukin enhancer-binding factor 2 OS=Homo sapiens GN=ILF2 PE=1 SV=2 | 45740.2 | 1.9 | 8 | 0 | 1.9 | 8 | 0 |
| Q12906 | Interleukin enhancer-binding factor 3 OS=Homo sapiens GN=ILF3 PE=1 SV=3 | 105106.1 | 2.0 | 9 | 0 | 2.0 | 9 | 0 |
| Q13162 | Peroxiredoxin-4 OS=Homo sapiens GN=PRDX4 PE=1 SV=1 | 32641.5 | 1.8 | 8 | 0 | 1.8 | 7 | 0 |
| Q14103 | Heterogeneous nuclear ribonucleoprotein D0 OS=Homo sapiens GN=HNRNPD PE=1 SV=1 | 43217.6 | 2.0 | 8 | 0 | 2.0 | 8 | 0 |
| Q14697 | Neutral alpha-glucosidase AB OS=Homo sapiens GN=GANAB PE=1 SV=3 | 111220.6 | 1.7 | 7 | 0 | 1.6 | 7 | 0 |
| Q15746 | Myosin light chain kinase, smooth muscle OS=Homo sapiens GN=MYLK PE=1 SV=4 | 234352.2 | 0.4 | 0 | 11 | 0.6 | 0 | 8 |
| Q16853 | Membrane primary amine oxidase OS=Homo sapiens GN=AOC3 PE=1 SV=3 | 87209 | 0.5 | 0 | 8 | 0.5 | 0 | 11 |
| Q8WX93 | Palladin OS=Homo sapiens GN=PALLD PE=1 SV=3 | 161877.1 | 0.5 | 0 | 11 | 0.6 | 0 | 7 |
| Q9NZN4 | EH domain-containing protein 2 OS=Homo sapiens GN=EHD2 PE=1 SV=2 | 66809.3 | 0.5 | 0 | 9 | 0.6 | 0 | 8 |
| Q9UL46 | Proteasome activator complex subunit 2 OS=Homo sapiens GN=PSME2 PE=1 SV=4 | 30743.7 | 2.0 | 8 | 0 | 2.0 | 9 | 0 |
