# Supplementary material for: Quantitative mass spectrometry analysis reveals a panel of nine proteins as diagnostic markers for colon adenocarcinomas
Source: Oncotarget. 2018 Feb 5;9(17):13530–44. doi: 10.18632/oncotarget.24418 (PMC5862596; doi:10.18632/oncotarget.24418)
Supplement: Supplementary file 5 [file oncotarget-09-13530-s005.docx]

| **Supplementary Table 1D: Comparison of significantly differentially expressed proteins identified in present study (Supp. Table 1c) with significantly differentially expressed proteins identified from Jankova et al. 2011 and Wisniewski et al. 2012** | | | | | | |
| --- | --- | --- | --- | --- | --- | --- |
| **Uniprot Accession ID** | **protein description** | **protein_Mol. Wt** | **Average Fold Change from Our Study (CAC/Control)_TPP** | **Average Fold Change from Our Study (CAC/Control)_SpectrumMill** | **Fold Change from Jankova et al. 2011 Study (Tumor/Control)** | **Fold Change from Wisniewski et al. 2012 Study (CAC/Control)** |
| P31949* | Protein S100-A11 | 13295.9 | 2.1 | 2.3 | 2.4 | 4.9 |
| P14618* | Pyruvate kinase PKM | 63840.1 | 1.7 | 1.6 | 1.5 | 4.2 |
| P50454 | Serpin H1 | 51455.1 | 1.9 | 2.2 | 1.7 | 3.9 |
| P06702* | Protein S100-A9 | 14884.5 | 3.2 | 3.0 | 4.2 | Not reported |
| P05109* | Protein S100-A8 | 12621.2 | 3.2 | 2.8 | 3.5 | Not reported |
| P05164 | Myeloperoxidase | 88008.4 | 2.2 | 2.3 | 3.2 | Not reported |
| P06731 | Carcinoembryonic antigen-related cell adhesion molecule 5 | 79985.6 | 2.6 | 2.3 | 1.8 | Not reported |
| P00338* | L-lactate dehydrogenase A chain | 41009.6 | 1.9 | 1.9 | 1.6 | Not reported |
| P07339 | Cathepsin D | 48380.4 | 1.9 | 2.0 | -1.5 | Not reported |
| P51884* | Lumican | 42374.5 | -1.8 | -1.7 | -1.9 | Not reported |
| P07585 | Decorin | 43980.5 | -2.0 | -1.9 | -2.2 | Not reported |
| P00918 | Carbonic anhydrase 2 | 32762.2 | -2.2 | -1.9 | -2.8 | -19.1 |
| P51858* | Hepatoma-derived growth factor | 30794.1 | 1.8 | 1.9 | Not reported | 4.8 |
| P20290 | Transcription factor BTF3 | 24531.1 | 2.1 | 2.4 | Not reported | 4.5 |
| P19338 | Nucleolin | 89643.9 | 2.3 | 2.5 | <1.5 | 4.4 |
| P08195 | 4F2 cell-surface antigen heavy chain | 72401.4 | 3.0 | 2.8 | Not reported | 3.8 |
| O14980 | Exportin-1 | 133462.3 | 1.8 | 2.2 | Not reported | 3.7 |
| P78527 | DNA-dependent protein kinase catalytic subunit | 513830.6 | 2.0 | 2.9 | Not reported | 3.6 |
| P09429 | High mobility group protein B1 | 31263.1 | 2.2 | 2.0 | Not reported | 3.2 |
| P49327 | Fatty acid synthase | 288299.2 | 2.0 | 2.1 | Not reported | 3.2 |
| P11940 | Polyadenylate-binding protein 1 | 76808.2 | 2.2 | 2.4 | Not reported | 3.1 |
| Q12905 | Interleukin enhancer-binding factor 2 | 45740.2 | 1.9 | 1.9 | Not reported | 3.1 |
| P31948 | Stress-induced-phosphoprotein 1 | 72347.5 | 2.0 | 2.7 | Not reported | 2.9 |
| P39687 | Acidic leucine-rich nuclear phosphoprotein 32 family member A | 31005.5 | 1.7 | 2.0 | Not reported | 2.9 |
| Q07955 | Serine/arginine-rich splicing factor 1 | 29011.4 | 1.9 | 1.8 | Not reported | 2.9 |
| P10599 | Thioredoxin | 13752.4 | 2.0 | 1.8 | Not reported | 2.8 |
| P46781 | 40S ribosomal protein S9 | 25387 | 2.4 | 2.2 | Not reported | 2.8 |
| Q12906 | Interleukin enhancer-binding factor 3 | 105106.1 | 2.0 | 2.0 | Not reported | 2.8 |
| P62277 | 40S ribosomal protein S13 | 19816.8 | 2.0 | 1.9 | Not reported | 2.7 |
| P38646 | Stress-70 protein, mitochondrial | 81460.5 | 1.8 | 1.9 | Not reported | 2.6 |
| P00558 | Phosphoglycerate kinase 1 | 51067.8 | 1.8 | 2.0 | Not reported | 2.5 |
| P48643 | T-complex protein 1 subunit epsilon | 66325.1 | 1.8 | 2.0 | Not reported | 2.5 |
| P52272 | Heterogeneous nuclear ribonucleoprotein M | 83565.8 | 1.5 | 1.6 | Not reported | 2.5 |
| O60506 | Heterogeneous nuclear ribonucleoprotein Q | 76460.7 | 1.9 | 1.7 | Not reported | 2.4 |
| P13639 | Elongation factor 2 | 105676.4 | 1.7 | 1.8 | Not reported | 2.3 |
| P26641 | Elongation factor 1-gamma | 55073.1 | 2.3 | 2.5 | Not reported | 2.3 |
| P46777 | 60S ribosomal protein L5 | 39635.6 | 2.0 | 1.6 | Not reported | 2.3 |
| P62937 | Peptidyl-prolyl cis-trans isomerase A | 20258.5 | 1.9 | 1.8 | Not reported | 2.3 |
| Q14103 | Heterogeneous nuclear ribonucleoprotein D0 | 43217.6 | 2.0 | 2.0 | Not reported | 2.3 |
| P06748 | Nucleophosmin | 37502.7 | 1.6 | 2.1 | <1.5 | 2.2 |
| P21796 | Voltage-dependent anion-selective channel protein 1 | 34490 | 2.1 | 2.2 | Not reported | 2.2 |
| Q04837 | Single-stranded DNA-binding protein, mitochondrial | 18412.6 | 1.7 | 2.0 | Not reported | 2.2 |
| Q01105 | Protein SET | 36803.8 | 2.3 | 4.3 | <1.5 | 2.1 |
| P29401 | Transketolase | 74615.5 | 1.7 | 2.0 | <1.5 | 2.0 |
| P60842 | Eukaryotic initiation factor 4A-I | 49264.4 | 1.8 | 2.0 | Not reported | 2.0 |
| P28838 | Cytosol aminopeptidase | 61754.3 | 1.8 | 1.7 | Not reported | 1.9 |
| P39019 | 40S ribosomal protein S19 | 18222.5 | 1.9 | 2.4 | Not reported | 1.9 |
| P40926 | Malate dehydrogenase, mitochondrial | 39707.1 | 2.1 | 2.1 | Not reported | 1.9 |
| P31946 | 14-3-3 protein beta/alpha | 31079.1 | 1.8 | 1.7 | Not reported | 1.8 |
| P61247 | 40S ribosomal protein S3a | 35650.5 | 1.9 | 2.0 | Not reported | 1.8 |
| P62241 | 40S ribosomal protein S8 | 28958.8 | 2.1 | 2.3 | Not reported | 1.7 |
| P04843 | Dolichyl-diphosphooligosaccharide--protein glycosyltransferase subunit 1 | 74448.4 | 1.9 | 2.0 | Not reported | 1.6 |
| P37837 | Transaldolase | 42035.2 | 1.9 | 2.2 | Not reported | 1.5 |
| O14558 | Heat shock protein beta-6 | 17624.8 | -2.5 | -2.4 | Not reported | Not reported |
| O14818 | Proteasome subunit alpha type-7 | 31228.9 | 1.7 | 1.8 | Not reported | <1.5 |
| O95336 | 6-phosphogluconolactonase | 28984.9 | 1.6 | 1.8 | Not reported | Not reported |
| P04080 | Cystatin-B | 12493.8 | 2.1 | 2.2 | Not reported | Not reported |
| P04792 | Heat shock protein beta-1 | 23848.2 | -2.0 | -1.6 | Not reported | Not reported |
| P07108 | Acyl-CoA-binding protein | 11918.2 | 1.8 | 2.0 | Not reported | Not reported |
| P07951 | Tropomyosin beta chain | 38586.3 | -4.4 | -2.9 | Not reported | Not reported |
| P09525 | Annexin A4 | 39281.7 | 2.0 | 2.0 | Not reported | Not reported |
| P12277 | Creatine kinase B-type | 45956 | -1.7 | -1.5 | Not reported | Not reported |
| P14314 | Glucosidase 2 subunit beta | 65151.4 | 2.0 | 2.0 | Not reported | Not reported |
| P18124 | 60S ribosomal protein L7 | 34183.5 | 1.6 | 1.7 | Not reported | Not reported |
| P20618 | Proteasome subunit beta type-1 | 28591.1 | 1.5 | 1.9 | Not reported | Not reported |
| P21291 | Cysteine and glycine-rich protein 1 | 24738.4 | -2.4 | -2.3 | Not reported | Not reported |
| P21333 | Filamin-A | 306537.1 | -2.2 | -1.7 | Not reported | Not reported |
| P23284 | Peptidyl-prolyl cis-trans isomerase B | 27547.2 | 2.1 | 1.9 | Not reported | Not reported |
| P24844 | Myosin regulatory light polypeptide 9 | 21902.1 | -4.5 | -2.4 | Not reported | Not reported |
| P26599 | Polypyrimidine tract-binding protein 1 | 62148.5 | 1.6 | 1.6 | Not reported | Not reported |
| P35749 | Myosin-11 | 257598.5 | -3.6 | -2.0 | Not reported | Not reported |
| P40121 | Macrophage-capping protein | 42242.8 | 1.8 | 1.9 | Not reported | Not reported |
| P43243 | Matrin-3 | 104649.2 | 1.9 | 2.2 | Not reported | Not reported |
| P51888 | Prolargin | 46226.6 | -2.1 | -2.0 | Not reported | Not reported |
| P51911 | Calponin-1 | 36656.6 | -4.0 | -3.1 | Not reported | Not reported |
| P53999 | Activated RNA polymerase II transcriptional coactivator p15 | 17134 | 2.1 | 2.4 | Not reported | Not reported |
| P55072 | Transitional endoplasmic reticulum ATPase | 96780.1 | 1.8 | 1.8 | Not reported | Not reported |
| P60174 | Triosephosphate isomerase | 33958.8 | 1.8 | 1.8 | Not reported | Not reported |
| P61626 | Lysozyme C | 17858.1 | 2.8 | 2.8 | Not reported | Not reported |
| P68431 | Histone H3.1 | 17391.9 | 2.9 | 2.5 | Not reported | Not reported |
| P78417 | Glutathione S-transferase omega-1 | 31166.2 | 1.6 | 2.1 | Not reported | Not reported |
| Q01995 | Transgelin | 25118.2 | -3.9 | -2.9 | Not reported | Not reported |
| Q02878 | 60S ribosomal protein L6 | 40280.4 | 1.8 | 2.1 | Not reported | Not reported |
| Q05682 | Caldesmon | 108278.5 | -1.9 | -1.5 | Not reported | Not reported |
| Q13162 | Peroxiredoxin-4 | 32641.5 | 1.8 | 1.8 | Not reported | Not reported |
| Q14697 | Neutral alpha-glucosidase AB | 111220.6 | 1.7 | 1.6 | Not reported | Not reported |
| Q15746 | Myosin light chain kinase, smooth muscle | 234352.2 | -2.3 | -1.7 | Not reported | Not reported |
| Q8WX93 | Palladin | 161877.1 | -1.9 | -1.6 | Not reported | Not reported |
| Q9UL46 | Proteasome activator complex subunit 2 | 30743.7 | 2.0 | 2.0 | Not reported | Not reported |
| P12956 | X-ray repair cross-complementing protein 6 | 78632.1 | 1.8 | 2.0 | Not reported | -2.3 |
| P00915 | Carbonic anhydrase 1 | 31521.5 | -1.9 | -1.5 | Not reported | -4.4 |
| Q03135 | Caveolin-1 | 22372.2 | -2.7 | -2.7 | Not reported | -6.2 |
| Q9NZN4* | EH domain-containing protein 2 | 66809.3 | -1.9 | -1.6 | Not reported | -24.5 |
| Q16853* | Membrane primary amine oxidase | 87209 | -2.0 | -2.0 | Not reported | -30.8 |
|  |  |  |  |  |  |  |
| * Proteins validated using MRM | |  |  |  |  |  |
