# Supplementary material for: Quantitative mass spectrometry analysis reveals a panel of nine proteins as diagnostic markers for colon adenocarcinomas
Source: Oncotarget. 2018 Feb 5;9(17):13530–44. doi: 10.18632/oncotarget.24418 (PMC5862596; doi:10.18632/oncotarget.24418)
Supplement: Supplementary file 6 [file oncotarget-09-13530-s006.docx]

| **Supplementary Table 1E: Proteins involved in biological processes (Output from KEGG pathway database, Database for Annotation, Visualization and Integrated Discovery (DAVID) Functional Annotation Bioinformatics Analysis v6.8) using the 285 dysregulated proteins identified using TPP or Spectrum Mill) altered in colon adenocarcinoma** | | | | | | | |
| --- | --- | --- | --- | --- | --- | --- | --- |
| UniProt Accession number | Protein Name | TPP analysis | | | Spectrum Mill Analysis | | |
|  |  | Average Fold Change | Number of patients with | | Average Fold Change | Number of patients with | |
|  |  |  | up-regulation | down-regulation |  | up-regulation | down-regulation |
| **Ribosomal proteins** | |  |  |  |  |  |  |
| P05386 | 60S acidic ribosomal protein P1 | 1.8 | 7 | 0 | * | * | * |
| P05387 | 60S acidic ribosomal protein P2 | * | * | * | 1.7 | 7 | 0 |
| P05388 | 60S acidic ribosomal protein P0 | 1.9 | 8 | 0 | * | * | * |
| P08708 | 40S ribosomal protein S17 | * | * | * | 1.9 | 7 | 0 |
| P15880 | 40S ribosomal protein S2 | * | * | * | 2.1 | 8 | 0 |
| P18124 | 60S ribosomal protein L7 | 1.6 | 7 | 0 | 1.7 | 9 | 0 |
| P18621 | 60S ribosomal protein L17 | 2.1 | 8 | 1 | * | * | * |
| P23396 | 40S ribosomal protein S3 | 1.7 | 7 | 0 | * | * | * |
| P35268 | 60S ribosomal protein L22 | * | * | * | 1.8 | 7 | 0 |
| P36578 | 60S ribosomal protein L4 | * | * | * | 2.3 | 9 | 0 |
| P39019 | 40S ribosomal protein S19 | 1.9 | 7 | 0 | 2.4 | 7 | 0 |
| P40429 | 60S ribosomal protein L13a | 1.8 | 7 | 1 | * | * | * |
| P46777 | 60S ribosomal protein L5 | 2 | 7 | 0 | 1.6 | 7 | 0 |
| P46781 | 40S ribosomal protein S9 | 2.4 | 7 | 0 | 2.2 | 7 | 1 |
| P46783 | 40S ribosomal protein S10 | * | * | * | 2.3 | 7 | 2 |
| P60866 | 40S ribosomal protein S20 | 1.7 | 7 | 0 | * | * | * |
| P61247 | 40S ribosomal protein S3a | 1.9 | 8 | 0 | 2 | 9 | 0 |
| P61313 | 60S ribosomal protein L15 | 2.1 | 7 | 0 | * | * | * |
| P62081 | 40S ribosomal protein S7 | 2.1 | 7 | 1 | * | * | * |
| P62241 | 40S ribosomal protein S8 | 2.1 | 8 | 0 | 2.3 | 8 | 0 |
| P62244 | 40S ribosomal protein S15a | 3 | 8 | 0 | * | * | * |
| P62249 | 40S ribosomal protein S16 | 2.7 | 8 | 1 | * | * | * |
| P62263 | 40S ribosomal protein S14 | 2.1 | 8 | 1 | * | * | * |
| P62269 | 40S ribosomal protein S18 | 1.6 | 7 | 1 | * | * | * |
| P62277 | 40S ribosomal protein S13 | 2 | 8 | 0 | 1.9 | 8 | 0 |
| P62424 | 60S ribosomal protein L7a | 2.1 | 9 | 0 | * | * | * |
| P62750 | 60S ribosomal protein L23a | 1.9 | 9 | 0 | * | * | * |
| P62753 | 40S ribosomal protein S6 | 1.9 | 7 | 0 | * | * | * |
| P62829 | 60S ribosomal protein L23 | 2.1 | 9 | 0 | * | * | * |
| P62847 | 40S ribosomal protein S24 | 2.1 | 8 | 0 | * | * | * |
| P62851 | 40S ribosomal protein S25 | 2.3 | 8 | 0 | * | * | * |
| P62913 | 60S ribosomal protein L11 | 1.7 | 8 | 0 | * | * | * |
| Q02878 | 60S ribosomal protein L6 | 1.8 | 7 | 0 | 2.1 | 9 | 0 |
| Q07020 | 60S ribosomal protein L18 | 2.4 | 8 | 0 | * | * | * |
|  | | | | | | | |
| **Glycolysis / Gluconeogenesis** | |  |  |  |  |  |  |
| P00338 | L-lactate dehydrogenase A chain | 1.9 | 7 | 0 | 1.9 | 8 | 0 |
| P00558 | Phosphoglycerate kinase 1 | 1.8 | 8 | 0 | 2 | 9 | 0 |
| P04075 | Fructose-bisphosphate aldolase A | * | * | * | 1.5 | 7 | 0 |
| P06733 | Alpha-enolase | * | * | * | 1.6 | 7 | 0 |
| P06744 | Glucose-6-phosphate isomerase | * | * | * | 1.7 | 7 | 0 |
| P09972 | Fructose-bisphosphate aldolase C | * | * | * | 1.8 | 9 | 0 |
| P14550 | Alcohol dehydrogenase [NADP(+)] | * | * | * | 1.7 | 7 | 0 |
| P14618 | Pyruvate kinase PKM | 1.7 | 8 | 0 | 1.6 | 7 | 0 |
| P17858 | ATP-dependent 6-phosphofructokinase, liver type | 2 | 7 | 0 | * | * | * |
| P49419 | Alpha-aminoadipicsemialdehyde dehydrogenase | 1.6 | 7 | 2 | * | * | * |
| P60174 | Triosephosphate isomerase | 1.8 | 7 | 0 | 1.8 | 8 | 0 |
|  | | | | | | | |
| **Biosynthesis of amino acids** | |  |  |  |  |  |  |
| O75390 | Citrate synthase, mitochondrial | 1.9 | 7 | 0 | * | * | * |
| P00558 | Phosphoglycerate kinase 1 | 1.8 | 8 | 0 | 2 | 9 | 0 |
| P04075 | Fructose-bisphosphate aldolase A | * | * | * | 1.5 | 7 | 0 |
| P06733 | Alpha-enolase | * | * | * | 1.6 | 7 | 0 |
| P09972 | Fructose-bisphosphate aldolase C | * | * | * | 1.8 | 9 | 0 |
| P14618 | Pyruvate kinase PKM | 1.7 | 8 | 0 | 1.6 | 7 | 0 |
| P17858 | ATP-dependent 6-phosphofructokinase, liver type | 2 | 7 | 0 | * | * | * |
| P29401 | Transketolase | 1.7 | 8 | 0 | 2 | 8 | 0 |
| P37837 | Transaldolase | 1.9 | 7 | 0 | 2.2 | 7 | 0 |
| P49419 | Alpha-aminoadipicsemialdehyde dehydrogenase | 1.6 | 7 | 2 | * | * | * |
| P60174 | Triosephosphate isomerase | 1.8 | 7 | 0 | 1.8 | 8 | 0 |
|  | | | | | | | |
| **Pentose phosphate pathway** | |  |  |  |  |  |  |
| O95336 | 6-phosphogluconolactonase | 1.6 | 8 | 0 | 1.8 | 7 | 0 |
| P04075 | Fructose-bisphosphate aldolase A | * | * | * | 1.5 | 7 | 0 |
| P06744 | Glucose-6-phosphate isomerase | * | * | * | 1.7 | 7 | 0 |
| P09972 | Fructose-bisphosphate aldolase C | * | * | * | 1.8 | 9 | 0 |
| P17858 | ATP-dependent 6-phosphofructokinase, liver type | 2 | 7 | 0 | * | * | * |
| P29401 | Transketolase | 1.7 | 8 | 0 | 2 | 8 | 0 |
| P37837 | Transaldolase | 1.9 | 7 | 0 | 2.2 | 7 | 0 |
|  | | | | | | | |
| **Protein processing in endoplasmic reticulum** | |  |  |  |  |  |  |
| P04843 | Dolichyl-diphosphooligosaccharide--protein glycosyltransferase subunit 1 | 1.9 | 7 | 0 | 2 | 8 | 0 |
| P07237 | Protein disulfide-isomerase | * | * | * | 1.9 | 7 | 0 |
| P07900 | Heat shock protein HSP 90-alpha | 1.8 | 8 | 0 | * | * | * |
| P08238 | Heat shock protein HSP 90-beta | 2.1 | 9 | 0 | * | * | * |
| P11142 | Heat shock cognate 71 kDa protein | 1.7 | 7 | 0 | * | * | * |
| P14314 | Glucosidase 2 subunit beta | 2 | 8 | 0 | 2 | 7 | 0 |
| P27797 | Calreticulin | 2 | 8 | 0 | * | * | * |
| P27824 | Calnexin | * | * | * | 1.7 | 9 | 0 |
| P30101 | Protein disulfide-isomerase A3 | 1.8 | 8 | 0 | * | * | * |
| P49257 | Protein ERGIC-53 | * | * | * | 2 | 7 | 0 |
| P55072 | Transitional endoplasmic reticulum ATPase | 1.8 | 7 | 0 | 1.8 | 8 | 0 |
| Q14697 | Neutral alpha-glucosidase AB | 1.7 | 7 | 0 | 1.6 | 7 | 0 |
| Q15084 | Protein disulfide-isomerase A6 | * | * | * | 1.8 | 8 | 0 |
| Q15436 | Protein transport protein Sec23A | 2 | 8 | 1 | * | * | * |
| Q9P2E9 | Ribosome-binding protein 1 | * | * | * | 1.6 | 8 | 0 |
|  | | | | | | | |
| **Spliceosome** | |  |  |  |  |  |  |
| O43143 | Pre-mRNA-splicing factor ATP-dependent RNA helicase DHX15 | 2.3 | 8 | 0 | * | * | * |
| P07910 | Heterogeneous nuclear ribonucleoproteins C1/C2 | 2.1 | 8 | 0 | * | * | * |
| P09651 | Heterogeneous nuclear ribonucleoprotein A1 | 2.1 | 9 | 0 | * | * | * |
| P11142 | Heat shock cognate 71 kDa protein | 1.7 | 7 | 0 | * | * | * |
| P51991 | Heterogeneous nuclear ribonucleoprotein A3 | * | * | * | 1.9 | 8 | 0 |
| P52272 | Heterogeneous nuclear ribonucleoprotein M | 1.5 | 7 | 0 | 1.6 | 7 | 0 |
| P62304 | Small nuclear ribonucleoprotein E | 2 | 8 | 1 | * | * | * |
| P62318 | Small nuclear ribonucleoprotein Sm D3 | 2.1 | 9 | 0 | * | * | * |
| Q00839 | Heterogeneous nuclear ribonucleoprotein U | * | * | * | 2 | 9 | 0 |
| Q07955 | Serine/arginine-rich splicing factor 1 | 1.9 | 8 | 0 | 1.8 | 9 | 0 |
| Q13838 | Spliceosome RNA helicase DDX39B | * | * | * | 2.4 | 8 | 0 |
| Q15365 | Poly(rC)-binding protein 1 | 1.9 | 7 | 0 | * | * | * |
| Q86V81 | THO complex subunit 4 | 2.1 | 7 | 0 | * | * | * |
|  | | | | | | | |
| **Proteasome** | |  |  |  |  |  |  |
| O14818 | Proteasome subunit alpha type-7 | 1.7 | 7 | 0 | 1.8 | 9 | 0 |
| P20618 | Proteasome subunit beta type-1 | 1.5 | 7 | 0 | 1.9 | 10 | 0 |
| P25786 | Proteasome subunit alpha type-1 | 1.9 | 7 | 0 | * | * | * |
| P28062 | Proteasome subunit beta type-8 | 1.8 | 7 | 1 | * | * | * |
| P51665 | 26S proteasome non-ATPase regulatory subunit 7 | 2.4 | 7 | 0 | * | * | * |
| P62333 | 26S protease regulatory subunit 10B | 1.6 | 7 | 0 | * | * | * |
| Q9UL46 | Proteasome activator complex subunit 2 | 2 | 8 | 0 | 2 | 9 | 0 |
|  | | | | | | | |
| **Antigen processing and presentation** | |  |  |  |  |  |  |
| P07858 | Cathepsin B | 1.8 | 8 | 0 | * | * | * |
| P07900 | Heat shock protein HSP 90-alpha | 1.8 | 8 | 0 | * | * | * |
| P08238 | Heat shock protein HSP 90-beta | 2.1 | 9 | 0 | * | * | * |
| P11142 | Heat shock cognate 71 kDa protein | 1.7 | 7 | 0 | * | * | * |
| P27797 | Calreticulin | 2 | 8 | 0 | * | * | * |
| P27824 | Calnexin | * | * | * | 1.7 | 9 | 0 |
| P30101 | Protein disulfide-isomerase A3 | 1.8 | 8 | 0 | * | * | * |
| Q9UL46 | Proteasome activator complex subunit 2 | 2 | 8 | 0 | 2 | 9 | 0 |
|  | | | | | | | |
| **Pyruvate metabolism** | |  |  |  |  |  |  |
| P00338 | L-lactate dehydrogenase A chain | 1.9 | 7 | 0 | 1.9 | 8 | 0 |
| P14618 | Pyruvate kinase PKM | 1.7 | 8 | 0 | 1.6 | 7 | 0 |
| P40926 | Malate dehydrogenase, mitochondrial | 2.1 | 8 | 0 | 2.1 | 8 | 0 |
| P49419 | Alpha-aminoadipicsemialdehyde dehydrogenase | 1.6 | 7 | 2 | * | * | * |
| Q9UBQ7 | Glyoxylate reductase/hydroxypyruvate reductase | 2.1 | 7 | 0 | * | * | * |
|  | | | | | | | |
| **Fructose and mannose metabolism** | |  |  |  |  |  |  |
| P04075 | Fructose-bisphosphate aldolase A | * | * | * | 1.5 | 7 | 0 |
| P09972 | Fructose-bisphosphate aldolase C | * | * | * | 1.8 | 9 | 0 |
| P17858 | ATP-dependent 6-phosphofructokinase, liver type | 2 | 7 | 0 | * | * | * |
| P60174 | Triosephosphate isomerase | 1.8 | 7 | 0 | 1.8 | 8 | 0 |
|  | | | | | | | |
| **Focal adhesion** | |  |  |  |  |  |  |
| P12109 | Collagen alpha-1(VI) chain | 0.6 | 0 | 7 | * | * | * |
| P12111 | Collagen alpha-3(VI) chain | 0.6 | 0 | 7 | * | * | * |
| P21333 | Filamin-A | 0.5 | 0 | 11 | 0.6 | 0 | 9 |
| P24844 | Myosin regulatory light polypeptide 9 | 0.2 | 0 | 11 | 0.4 | 0 | 8 |
| Q03135 | Caveolin-1 | 0.4 | 0 | 10 | 0.4 | 0 | 8 |
| Q13418 | Integrin-linked protein kinase | * | * | * | 0.5 | 0 | 7 |
| Q14315 | Filamin-C | 0.5 | 0 | 8 | * | * | * |
| Q15746 | Myosin light chain kinase, smooth muscle | 0.4 | 0 | 11 | 0.6 | 0 | 8 |
|  | | | | | | | |
| **Vascular smooth muscle contraction** | |  |  |  |  |  |  |
| P24844 | Myosin regulatory light polypeptide 9 | 0.2 | 0 | 11 | 0.4 | 0 | 8 |
| P60660 | Myosin light polypeptide 6 | 0.3 | 0 | 11 | * | * | * |
| P63267 | Actin, gamma-enteric smooth muscle | 0.2 | 0 | 8 | * | * | * |
| Q05682 | Caldesmon | 0.5 | 0 | 9 | 0.7 | 0 | 7 |
| Q15746 | Myosin light chain kinase, smooth muscle | 0.4 | 0 | 11 | 0.6 | 0 | 8 |
|  | | | | | | | |
| **Proteoglycans in cancer** | |  |  |  |  |  |  |
| P07585 | Decorin | 0.5 | 0 | 10 | 0.5 | 0 | 8 |
| P21333 | Filamin-A | 0.5 | 0 | 11 | 0.6 | 0 | 9 |
| P51884 | Lumican | 0.6 | 0 | 9 | 0.6 | 0 | 8 |
| Q03135 | Caveolin-1 | 0.4 | 0 | 10 | 0.4 | 0 | 8 |
| Q14315 | Filamin-C | 0.5 | 0 | 8 | * | * | * |
