# Supplementary material for: Quantitative mass spectrometry analysis reveals a panel of nine proteins as diagnostic markers for colon adenocarcinomas
Source: Oncotarget. 2018 Feb 5;9(17):13530–44. doi: 10.18632/oncotarget.24418 (PMC5862596; doi:10.18632/oncotarget.24418)
Supplement: Supplementary file 7 [file oncotarget-09-13530-s007.docx]

| **Supplementary Table 1F: Peptide details for the 9 proteins validated using MRM** | | | | | | | | | | | |
| --- | --- | --- | --- | --- | --- | --- | --- | --- | --- | --- | --- |
| Sr. No. | Gene Name | Peptides | Transitions | Parent m/z | Product m/z | Collision Energy | Retention time | Peptide length | Adjusted p-Value | Fold Change | Proteotypic (Shotgun/MRM) |
| 1 | HDGF | GYPHWPAR | y4 | 328.4997 | 529.2881 | 11.3 | 4.7 | 8 | 0 | 3.13 | Yes/Yes |
|  |  |  | y5 | 328.4997 | 382.2035 |  |  |  |  |  |  |
|  |  |  | y6 | 328.4997 | 333.6772 |  |  |  |  |  |  |
|  |  | IDEMPEAAVK | y6 | 551.7761 | 989.4608 | 21.6 | 4.9 | 10 | 0 | 4.02 | Yes/Yes |
|  |  |  | y8 | 551.7761 | 874.4339 |  |  |  |  |  |  |
|  |  |  | y9 | 551.7761 | 614.3508 |  |  |  |  |  |  |
|  |  | EAENPEGEEK | y6 | 566.2437 | 931.4003 | 22.1 | 5.6 | 10 | 0.0002 | 4.41 | No/Yes |
|  |  |  | y7 | 566.2437 | 802.3577 |  |  |  |  |  |  |
|  |  |  | y8 | 566.2437 | 688.3148 |  |  |  |  |  |  |
| 2 | S100A9 | LGHPDTLNQGEFK | y2 | 485.9124 | 722.3468 | 17.1 | 4.6 | 13 | 0 | 11.47 | Yes/Yes |
|  |  |  | y4 | 485.9124 | 480.2453 |  |  |  |  |  |  |
|  |  |  | y6 | 485.9124 | 294.1812 |  |  |  |  |  |  |
|  |  | LTWASHEK | y4 | 486.2509 | 757.3628 | 18.9 | 4.1 | 8 | 0 | 9.44 | Yes/Yes |
|  |  |  | y5 | 486.2509 | 571.2835 |  |  |  |  |  |  |
|  |  |  | y6 | 486.2509 | 500.2463 |  |  |  |  |  |  |
|  |  | MSQLER | y3 | 382.192 | 632.3362 | 14.8 | 3.8 | 6 | 0 | 10.89 | No/Yes |
|  |  |  | y4 | 382.192 | 545.3042 |  |  |  |  |  |  |
|  |  |  | y5 | 382.192 | 417.2456 |  |  |  |  |  |  |
| 3 | S100A8 | GADVWFK | y2 | 411.7109 | 694.3559 | 16 | 5.7 | 7 | 0 | 9.79 | Yes/Yes |
|  |  |  | y3 | 411.7109 | 480.2605 |  |  |  |  |  |  |
|  |  |  | y5 | 411.7109 | 294.1812 |  |  |  |  |  |  |
|  |  | MLTELEK | y2 | 432.2308 | 732.4138 | 16.8 | 4.9 | 7 | 0 | 5.73 | Yes/Yes |
|  |  |  | y5 | 432.2308 | 619.3297 |  |  |  |  |  |  |
|  |  |  | y6 | 432.2308 | 276.1554 |  |  |  |  |  |  |
|  |  | YSLIK | y2 | 312.1918 | 460.313 | 12 | 4.8 | 5 | 0 | 11.04 | No/Yes |
|  |  |  | y3 | 312.1918 | 373.2809 |  |  |  |  |  |  |
|  |  |  | y4 | 312.1918 | 260.1969 |  |  |  |  |  |  |
| 4 | LDHA | VTLTSEEEAR | y6 | 567.7855 | 934.4476 | 22.2 | 4.4 | 10 | 0.0305 | 1.76 | Yes/Yes |
|  |  |  | y7 | 567.7855 | 821.3636 |  |  |  |  |  |  |
|  |  |  | y8 | 567.7855 | 720.3159 |  |  |  |  |  |  |
|  |  | SADTLWGIQK | y4 | 559.7957 | 960.5149 | 21.9 | 5.7 | 10 | 0 | 3.31 | Yes/Yes |
|  |  |  | y5 | 559.7957 | 631.3562 |  |  |  |  |  |  |
|  |  |  | y8 | 559.7957 | 445.2769 |  |  |  |  |  |  |
|  |  | DYNVTANSK | y5 | 506.2407 | 733.3839 | 19.7 | 4.1 | 9 | 0 | 2.98 | Yes/Yes |
|  |  |  | y6 | 506.2407 | 619.341 |  |  |  |  |  |  |
|  |  |  | y7 | 506.2407 | 520.2726 |  |  |  |  |  |  |
| 5 | PKM | LDIDSPPITAR | y6 | 599.3273 | 856.4523 | 23.5 | 5.6 | 11 | 0 | 2.86 | Yes/Yes |
|  |  |  | y7 | 599.3273 | 741.4254 |  |  |  |  |  |  |
|  |  |  | y8 | 599.3273 | 654.3933 |  |  |  |  |  |  |
|  |  | APIIAVTR | y4 | 420.7687 | 672.4403 | 16.3 | 5.0 | 8 | 0.0248 | 1.72 | No/Yes |
|  |  |  | y5 | 420.7687 | 559.3562 |  |  |  |  |  |  |
|  |  |  | y6 | 420.7687 | 446.2722 |  |  |  |  |  |  |
|  |  | VNFAMNVGK | y5 | 490.2551 | 766.3916 | 19.1 | 5.3 | 9 | 0 | 3.7 | Yes/Yes |
|  |  |  | y6 | 490.2551 | 619.3232 |  |  |  |  |  |  |
|  |  |  | y7 | 490.2551 | 548.2861 |  |  |  |  |  |  |
| 6 | S100A11 | DGYNYTLSK | y2 | 530.7509 | 725.3828 | 20.7 | 4.9 | 9 | 0 | 5.21 | Yes/Yes |
|  |  |  | y5 | 530.7509 | 611.3399 |  |  |  |  |  |  |
|  |  |  | y6 | 530.7509 | 234.1448 |  |  |  |  |  |  |
|  |  | DPGVLDR | y3 | 386.2034 | 559.3198 | 14.9 | 4.5 | 7 | 0 | 4.79 | Yes/Yes |
|  |  |  | y4 | 386.2034 | 502.2984 |  |  |  |  |  |  |
|  |  |  | y5 | 386.2034 | 403.23 |  |  |  |  |  |  |
|  |  | ISSPTETER | y6 | 510.2538 | 906.4163 | 19.9 | 3.9 | 9 | 0 | 5.29 | Yes/Yes |
|  |  |  | y7 | 510.2538 | 819.3843 |  |  |  |  |  |  |
|  |  |  | y8 | 510.2538 | 732.3523 |  |  |  |  |  |  |
| 7 | EHD2 | LEGHGLPANLPR | y2 | 425.2386 | 499.2987 | 14.9 | 4.9 | 12 | 0 | 0.19 | Yes/Yes |
|  |  |  | y4 | 425.2386 | 272.1717 |  |  |  |  |  |  |
|  |  |  | y6 | 425.2386 | 334.1979 |  |  |  |  |  |  |
|  |  | GYDFPAVLR | y5 | 519.2744 | 817.4567 | 20.3 | 6.1 | 9 | 0.0001 | 0.32 | No/Yes |
|  |  |  | y6 | 519.2744 | 702.4297 |  |  |  |  |  |  |
|  |  |  | y7 | 519.2744 | 555.3613 |  |  |  |  |  |  |
|  |  | ADMVETQQLMR | y4 | 661.3156 | 905.4509 | 25.9 | 5.3 | 11 | 0 | 0.11 | No/Yes |
|  |  |  | y6 | 661.3156 | 776.4083 |  |  |  |  |  |  |
|  |  |  | y7 | 661.3156 | 547.3021 |  |  |  |  |  |  |
| 8 | LUM | FNALQYLR | y3 | 512.7824 | 763.4461 | 20 | 6.2 | 8 | 0 | 0.25 | Yes/Yes |
|  |  |  | y4 | 512.7824 | 579.3249 |  |  |  |  |  |  |
|  |  |  | y6 | 512.7824 | 451.2663 |  |  |  |  |  |  |
|  |  | NNQIDHIDEK | y3 | 613.294 | 869.4363 | 24 | 3.9 | 10 | 0.0001 | 0.19 | No/Yes |
|  |  |  | y6 | 613.294 | 756.3523 |  |  |  |  |  |  |
|  |  |  | y7 | 613.294 | 391.1823 |  |  |  |  |  |  |
|  |  | SLEDLQLTHNK | y4 | 649.341 | 853.489 | 25.5 | 5 | 11 | 0.0001 | 0.39 | Yes/Yes |
|  |  |  | y5 | 649.341 | 612.3464 |  |  |  |  |  |  |
|  |  |  | y7 | 649.341 | 499.2623 |  |  |  |  |  |  |
| 9 | AOC3 | YQLAVTQR | y4 | 489.772 | 687.4148 | 19.1 | 4.8 | 8 | 0 | 0.12 | No/Yes |
|  |  |  | y5 | 489.772 | 574.3307 |  |  |  |  |  |  |
|  |  |  | y6 | 489.772 | 503.2936 |  |  |  |  |  |  |
|  |  | NLVTMTTAPR | y6 | 552.2975 | 876.4608 | 21.6 | 5.1 | 10 | 0 | 0.09 | No/Yes |
|  |  |  | y7 | 552.2975 | 777.3924 |  |  |  |  |  |  |
|  |  |  | y8 | 552.2975 | 676.3447 |  |  |  |  |  |  |
|  |  | AAALAHLDR | y4 | 469.2643 | 795.4472 | 18.3 | 4.2 | 9 | 0 | 0.1 | Yes/Yes |
|  |  |  | y5 | 469.2643 | 611.326 |  |  |  |  |  |  |
|  |  |  | y7 | 469.2643 | 540.2889 |  |  |  |  |  |  |
