# Supplementary material for: Quantitative mass spectrometry analysis reveals a panel of nine proteins as diagnostic markers for colon adenocarcinomas
Source: Oncotarget. 2018 Feb 5;9(17):13530–44. doi: 10.18632/oncotarget.24418 (PMC5862596; doi:10.18632/oncotarget.24418)
Supplement: Supplementary file 8 [file oncotarget-09-13530-s008.docx]

| **Supplementary Table 1G: 20 commonest proteins as discovered by the diffusion propagation analysis** | | | | |
| --- | --- | --- | --- | --- |
| EntrezID | Relatedness score | SYMBOL | Common | Percent |
| id:4914 | 0.210610677 | NTRK1 | 163 | 0.571929825 |
| id:6613 | 0.203207981 | SUMO2 | 160 | 0.561403509 |
| id:351 | 0.162345766 | APP | 145 | 0.50877193 |
| id:3725 | 0.204668871 | JUN | 143 | 0.501754386 |
| id:2099 | 0.188206855 | ESR1 | 131 | 0.459649123 |
| id:1994 | 0.148833071 | ELAVL1 | 130 | 0.456140351 |
| id:7412 | 0.187046494 | VCAM1 | 124 | 0.435087719 |
| id:7341 | 0.165477053 | SUMO1 | 111 | 0.389473684 |
| id:4171 | 0.191954573 | MCM2 | 111 | 0.389473684 |
| id:8452 | 0.171525996 | CUL3 | 106 | 0.371929825 |
| id:2335 | 0.186609206 | FN1 | 105 | 0.368421053 |
| id:1017 | 0.179305814 | CDK2 | 99 | 0.347368421 |
| id:4193 | 0.178104146 | MDM2 | 95 | 0.333333333 |
| id:7157 | 0.155068375 | TP53 | 91 | 0.319298246 |
| id:4738 | 0.158083264 | NEDD8 | 89 | 0.312280702 |
| id:9768 | 0.171452017 | PCLAF | 89 | 0.312280702 |
| id:11345 | 0.156937588 | GABARAPL2 | 72 | 0.252631579 |
| id:23710 | 0.164121959 | GABARAPL1 | 71 | 0.249122807 |
| id:9820 | 0.161824095 | CUL7 | 70 | 0.245614035 |
| id:10987 | 0.153685356 | COPS5 | 67 | 0.235087719 |
