# Supplementary material for: Quantitative mass spectrometry analysis reveals a panel of nine proteins as diagnostic markers for colon adenocarcinomas
Source: Oncotarget. 2018 Feb 5;9(17):13530–44. doi: 10.18632/oncotarget.24418 (PMC5862596; doi:10.18632/oncotarget.24418)
Supplement: Supplementary file 9 [file oncotarget-09-13530-s009.docx]

| **Supplementary Table 1H: List of differentially expressed proteins identified from iTRAQ analysis using Spectrum Mill and/or Trans Proteome Pipeline and their comparison with CPTAC and Human Protein Atlas data for colorectal cancer** | | | | | | | | | | | | | | | | | | | | | | | | | | | |
| --- | --- | --- | --- | --- | --- | --- | --- | --- | --- | --- | --- | --- | --- | --- | --- | --- | --- | --- | --- | --- | --- | --- | --- | --- | --- | --- | --- |
| **protein** | **protein description** | **Gene Name** | **Gene Ensemble ID** | **TPP_Average FC** | **TPP_Up_No.of patients** | **TPP_Down_No.of patients** | **SM_Average FC** | **SM_Up_No.of patients** | **SM_Down_No.of patients** | **CPTAC_Avg. Nor.** | **CPTAC_Avg. Tumor** | **CPTAC_FC tumor/normal** | **HPA_Gene name** | **HPA_Tumor** | **HPA_Level** | **HPA_Count patients** | **HPA_Total patients** | **HPA_Percent expression** | **HPA_Gene name** | **HPA_Tumor** | **HPA_Level** | **HPA_Count patients** | **HPA_Total patients** | **HPA_Percent expression** | | | |
| P06702 | Protein S100-A9 | S100A9 | ENSG00000163220 | 3.2 | 9 | 0 | 3.0 | 10 | 0 | 13.7 | 108.0 | 7.9 | S100A9 | colorectal cancer | Not detected | 10 | 12 | 83.33 |  |  |  |  |  |  |  |  |  |
| P05109 | Protein S100-A8 | S100A8 | ENSG00000143546 | 3.2 | 9 | 0 | 2.8 | 9 | 0 | 12.2 | 78.2 | 6.4 | S100A8 | colorectal cancer | Not detected | 11 | 12 | 91.67 |  |  |  |  |  |  |  |  |  |
| P08195 | 4F2 cell-surface antigen heavy chain | SLC3A2 | ENSG00000168003 | 3.0 | 10 | 0 | 2.8 | 8 | 0 | 9.5 | 15.2 | 1.6 | SLC3A2 | colorectal cancer | Medium | 7 | 12 | 58.33 |  |  |  |  |  |  |  |  |  |
| P68431 | Histone H3 | HIST1H3 | * | 2.9 | 8 | 0 | 2.5 | 10 | 0 | * | * | * | * | * | * | * | * | * |  |  |  |  |  |  |  |  |  |
| P61626 | Lysozyme C | LYZ | ENSG00000090382 | 2.8 | 8 | 0 | 2.8 | 7 | 0 | 8.0 | 33.1 | 4.1 | LYZ | colorectal cancer | Not detected | 12 | 12 | 100.00 |  |  |  |  |  |  |  |  |  |
| P06731 | Carcinoembryonic antigen-related cell adhesion molecule 5 | CEACAM5 | ENSG00000105388 | 2.6 | 10 | 0 | 2.3 | 7 | 1 | 17.0 | 19.6 | 1.2 | CEACAM5 | colorectal cancer | High | 6 | 10 | 60.00 |  |  |  |  |  |  |  |  |  |
| P46781 | 40S ribosomal protein S9 | RPS9 | ENSG00000170889 | 2.4 | 7 | 0 | 2.2 | 7 | 1 | 39.0 | 43.7 | 1.1 | RPS9 | colorectal cancer | Medium | 9 | 12 | 75.00 |  |  |  |  |  |  |  |  |  |
| Q01105 | Protein SET | SET | ENSG00000119335 | 2.3 | 9 | 0 | 4.3 | 7 | 0 | 16.7 | 11.8 | 0.7 | SET | colorectal cancer | High | 9 | 10 | 90.00 |  |  |  |  |  |  |  |  |  |
| P19338 | Nucleolin | NCL | ENSG00000115053 | 2.3 | 9 | 0 | 2.5 | 9 | 0 | 53.0 | 70.9 | 1.3 | NCL | colorectal cancer | High | 12 | 12 | 100.00 |  |  |  |  |  |  |  |  |  |
| P26641 | Elongation factor 1-gamma | EEF1G | ENSG00000254772 | 2.3 | 9 | 0 | 2.5 | 9 | 0 | 48.2 | 65.6 | 1.4 | EEF1G | colorectal cancer | Medium | 8 | 12 | 66.67 |  |  |  |  |  |  |  |  |  |
| P11940 | Polyadenylate-binding protein 1 | PABPC1 | ENSG00000070756 | 2.2 | 8 | 0 | 2.4 | 8 | 0 | 31.7 | 47.1 | 1.5 | PABPC1 | colorectal cancer | Medium | 8 | 12 | 66.67 |  |  |  |  |  |  |  |  |  |
| P09429 | High mobility group protein B1 | HMGB1 | ENSG00000189403 | 2.2 | 9 | 0 | 2.0 | 8 | 0 | 71.7 | 69.2 | 1.0 | HMGB1 | colorectal cancer | High | 9 | 11 | 81.82 |  |  |  |  |  |  |  |  |  |
| P05164 | Myeloperoxidase | MPO | ENSG00000005381 | 2.2 | 8 | 0 | 2.3 | 9 | 0 | 31.9 | 72.6 | 2.3 | MPO | colorectal cancer | Not detected | 11 | 11 | 100.00 |  |  |  |  |  |  |  |  |  |
| P53999 | Activated RNA polymerase II transcriptional coactivator p15 | SUB1 | ENSG00000113387 | 2.1 | 9 | 0 | 2.4 | 8 | 0 | 18.0 | 15.9 | 0.9 | SUB1 | colorectal cancer | High | 11 | 12 | 91.67 |  |  |  |  |  |  |  |  |  |
| P31949 | Protein S100-A11 | S100A11 | ENSG00000163191 | 2.1 | 9 | 0 | 2.3 | 10 | 0 | 22.9 | 38.2 | 1.7 | S100A11 | colorectal cancer | Low | 4 | 12 | 33.33 | S100A11 | colorectal cancer | Not detected | 4 | 12 | 33.33 |  |  |  |
| P04080 | Cystatin-B | CSTB | ENSG00000160213 | 2.1 | 10 | 0 | 2.2 | 10 | 0 | 43.1 | 28.6 | 0.7 | CSTB | colorectal cancer | Not detected | 11 | 12 | 91.67 |  |  |  |  |  |  |  |  |  |
| P62241 | 40S ribosomal protein S8 | RPS8 | * | 2.1 | 8 | 0 | 2.3 | 8 | 0 | 50.7 | 57.3 | 1.1 | * | * | * | * | * | * |  |  |  |  |  |  |  |  |  |
| P40926 | Malate dehydrogenase, mitochondrial | MDH2 | ENSG00000146701 | 2.1 | 8 | 0 | 2.1 | 8 | 0 | 149.3 | 137.4 | 0.9 | MDH2 | colorectal cancer | High | 11 | 11 | 100.00 |  |  |  |  |  |  |  |  |  |
| P21796 | Voltage-dependent anion-selective channel protein 1 | VDAC1 | ENSG00000213585 | 2.1 | 8 | 0 | 2.2 | 8 | 0 | 117.1 | 137.9 | 1.2 | VDAC1 | colorectal cancer | Medium | 7 | 12 | 58.33 |  |  |  |  |  |  |  |  |  |
| P20290 | Transcription factor BTF3 | BTF3 | ENSG00000145741 | 2.1 | 8 | 0 | 2.4 | 7 | 0 | 5.1 | 6.4 | 1.2 | BTF3 | colorectal cancer | High | 8 | 12 | 66.67 |  |  |  |  |  |  |  |  |  |
| P23284 | Peptidyl-prolyl cis-trans isomerase B | PPIB | ENSG00000166794 | 2.1 | 9 | 0 | 1.9 | 9 | 0 | 85.1 | 76.2 | 0.9 | PPIB | colorectal cancer | Medium | 10 | 11 | 90.91 |  |  |  |  |  |  |  |  |  |
| P31948 | Stress-induced-phosphoprotein 1 | STIP1 | ENSG00000168439 | 2.0 | 7 | 1 | 2.7 | 7 | 0 | 42.1 | 38.1 | 0.9 | STIP1 | colorectal cancer | High | 7 | 11 | 63.64 |  |  |  |  |  |  |  |  |  |
| P78527 | DNA-dependent protein kinase catalytic subunit | PRKDC | ENSG00000253729 | 2.0 | 10 | 0 | 2.9 | 7 | 0 | 3.9 | 156.2 | 39.9 | PRKDC | colorectal cancer | High | 10 | 10 | 100.00 |  |  |  |  |  |  |  |  |  |
| P09525 | Annexin A4 | ANXA4 | ENSG00000196975 | 2.0 | 8 | 0 | 2.0 | 8 | 0 | 71.2 | 74.6 | 1.0 | ANXA4 | colorectal cancer | Medium | 6 | 11 | 54.55 |  |  |  |  |  |  |  |  |  |
| P10599 | Thioredoxin | TXN | ENSG00000136810 | 2.0 | 8 | 1 | 1.8 | 7 | 0 | 17.4 | 20.4 | 1.2 | TXN | colorectal cancer | Medium | 10 | 11 | 90.91 |  |  |  |  |  |  |  |  |  |
| Q9UL46 | Proteasome activator complex subunit 2 | PSME2 | * | 2.0 | 8 | 0 | 2.0 | 9 | 0 | 35.3 | 37.4 | 1.1 | * | * | * | * | * | * |  |  |  |  |  |  |  |  |  |
| P62277 | 40S ribosomal protein S13 | RPS13 | ENSG00000110700 | 2.0 | 8 | 0 | 1.9 | 8 | 0 | 27.8 | 30.6 | 1.1 | RPS13 | colorectal cancer | High | 4 | 11 | 36.36 | RPS13 | colorectal cancer | Not detected | 4 | 11 | 36.36 |  |  |  |
| P14314 | Glucosidase 2 subunit beta | PRKCSH | ENSG00000130175 | 2.0 | 8 | 0 | 2.0 | 7 | 0 | 51.6 | 29.2 | 0.6 | PRKCSH | colorectal cancer | High | 11 | 12 | 91.67 |  |  |  |  |  |  |  |  |  |
| P46777 | 60S ribosomal protein L5 | RPL5 | ENSG00000122406 | 2.0 | 7 | 0 | 1.6 | 7 | 0 | 36.5 | 35.7 | 1.0 | RPL5 | colorectal cancer | Medium | 8 | 12 | 66.67 |  |  |  |  |  |  |  |  |  |
| Q12906 | Interleukin enhancer-binding factor 3 | ILF3 | ENSG00000129351 | 2.0 | 9 | 0 | 2.0 | 9 | 0 | 4.3 | 57.1 | 13.2 | ILF3 | colorectal cancer | High | 12 | 12 | 100.00 |  |  |  |  |  |  |  |  |  |
| Q14103 | Heterogeneous nuclear ribonucleoprotein D0 | HNRNPD | ENSG00000138668 | 2.0 | 8 | 0 | 2.0 | 8 | 0 | 1.5 | 36.6 | 23.9 | HNRNPD | colorectal cancer | High | 11 | 12 | 91.67 |  |  |  |  |  |  |  |  |  |
| P49327 | Fatty acid synthase | FASN | ENSG00000169710 | 2.0 | 8 | 0 | 2.1 | 8 | 0 | 70.0 | 114.8 | 1.6 | FASN | colorectal cancer | Medium | 4 | 10 | 40.00 | FASN | colorectal cancer | Not detected | 4 | 10 | 40.00 |  |  |  |
| P04843 | Dolichyl-diphosphooligosaccharide--protein glycosyltransferase subunit 1 | RPN1 | ENSG00000163902 | 1.9 | 7 | 0 | 2.0 | 8 | 0 | 68.7 | 70.8 | 1.0 | RPN1 | colorectal cancer | Medium | 9 | 12 | 75.00 |  |  |  |  |  |  |  |  |  |
| O60506 | Heterogeneous nuclear ribonucleoprotein Q | SYNCRIP | ENSG00000135316 | 1.9 | 7 | 0 | 1.7 | 7 | 0 | 27.6 | 48.4 | 1.8 | SYNCRIP | colorectal cancer | Medium | 8 | 12 | 66.67 |  |  |  |  |  |  |  |  |  |
| P43243 | Matrin-3 | MATR3 | ENSG00000015479 | 1.9 | 8 | 0 | 2.2 | 10 | 0 | 38.8 | 42.1 | 1.1 | MATR3 | colorectal cancer | High | 11 | 11 | 100.00 | MATR3 | colorectal cancer | High | 11 | 11 | 100.00 |  |  |  |
| P61247 | 40S ribosomal protein S3a | RPS3A | ENSG00000145425 | 1.9 | 8 | 0 | 2.0 | 9 | 0 | 35.1 | 63.6 | 1.8 | RPS3A | colorectal cancer | Medium | 11 | 12 | 91.67 |  |  |  |  |  |  |  |  |  |
| P37837 | Transaldolase | TALDO1 | * | 1.9 | 7 | 0 | 2.2 | 7 | 0 | 44.6 | 40.7 | 0.9 | * | * | * | * | * | * |  |  |  |  |  |  |  |  |  |
| Q07955 | Serine/arginine-rich splicing factor 1 | SRSF1 | ENSG00000136450 | 1.9 | 8 | 0 | 1.8 | 9 | 0 | 2.5 | 27.2 | 10.8 | SRSF1 | colorectal cancer | High | 12 | 12 | 100.00 |  |  |  |  |  |  |  |  |  |
| P39019 | 40S ribosomal protein S19 | RPS19 | * | 1.9 | 7 | 0 | 2.4 | 7 | 0 | 33.4 | 35.2 | 1.1 | * | * | * | * | * | * |  |  |  |  |  |  |  |  |  |
| P00338 | L-lactate dehydrogenase A chain | LDHA | ENSG00000134333 | 1.9 | 7 | 0 | 1.9 | 8 | 0 | 31.8 | 110.4 | 3.5 | LDHA | colorectal cancer | Medium | 8 | 12 | 66.67 |  |  |  |  |  |  |  |  |  |
| Q12905 | Interleukin enhancer-binding factor 2 | ILF2 | ENSG00000143621 | 1.9 | 8 | 0 | 1.9 | 8 | 0 | 33.4 | 42.8 | 1.3 | ILF2 | colorectal cancer | High | 12 | 12 | 100.00 |  |  |  |  |  |  |  |  |  |
| P50454 | Serpin H1 | SERPINH1 | ENSG00000149257 | 1.9 | 9 | 0 | 2.2 | 10 | 0 | 23.8 | 67.0 | 2.8 | SERPINH1 | colorectal cancer | Medium | 6 | 12 | 50.00 |  |  |  |  |  |  |  |  |  |
| P62937 | Peptidyl-prolyl cis-trans isomerase A | PPIA | ENSG00000196262 | 1.9 | 7 | 0 | 1.8 | 7 | 0 | 156.1 | 162.1 | 1.0 | PPIA | colorectal cancer | High | 7 | 10 | 70.00 |  |  |  |  |  |  |  |  |  |
| P07339 | Cathepsin D | CTSD | ENSG00000117984 | 1.9 | 7 | 0 | 2.0 | 7 | 0 | 126.5 | 59.0 | 0.5 | CTSD | colorectal cancer | Not detected | 9 | 12 | 75.00 |  |  |  |  |  |  |  |  |  |
| P00558 | Phosphoglycerate kinase 1 | PGK1 | ENSG00000102144 | 1.8 | 8 | 0 | 2.0 | 9 | 0 | 166.0 | 163.0 | 1.0 | PGK1 | colorectal cancer | Medium | 7 | 12 | 58.33 |  |  |  |  |  |  |  |  |  |
| P51858 | Hepatoma-derived growth factor | HDGF | ENSG00000143321 | 1.8 | 9 | 0 | 1.9 | 10 | 0 | 3.8 | 11.1 | 2.9 | HDGF | colorectal cancer | High | 10 | 10 | 100.00 |  |  |  |  |  |  |  |  |  |
| Q02878 | 60S ribosomal protein L6 | RPL6 | ENSG00000089009 | 1.8 | 7 | 0 | 2.1 | 9 | 0 | 46.9 | 59.4 | 1.3 | RPL6 | colorectal cancer | Low | 6 | 11 | 54.55 |  |  |  |  |  |  |  |  |  |
| P12956 | X-ray repair cross-complementing protein 6 | XRCC6 | ENSG00000196419 | 1.8 | 8 | 0 | 2.0 | 7 | 0 | 59.8 | 68.6 | 1.1 | XRCC6 | colorectal cancer | High | 10 | 12 | 83.33 |  |  |  |  |  |  |  |  |  |
| P40121 | Macrophage-capping protein | CAPG | ENSG00000042493 | 1.8 | 9 | 0 | 1.9 | 8 | 0 | 3.6 | 34.6 | 9.6 | CAPG | colorectal cancer | Not detected | 10 | 11 | 90.91 |  |  |  |  |  |  |  |  |  |
| P28838 | Cytosol aminopeptidase | LAP3 | ENSG00000002549 | 1.8 | 8 | 0 | 1.7 | 8 | 0 | 49.2 | 22.1 | 0.5 | LAP3 | colorectal cancer | Medium | 6 | 12 | 50.00 |  |  |  |  |  |  |  |  |  |
| P60174 | Triosephosphate isomerase | TPI1 | ENSG00000111669 | 1.8 | 7 | 0 | 1.8 | 8 | 0 | 201.2 | 65.6 | 0.3 | TPI1 | colorectal cancer | Medium | 5 | 11 | 45.45 |  |  |  |  |  |  |  |  |  |
| O14980 | Exportin-1 | XPO1 | ENSG00000082898 | 1.8 | 7 | 0 | 2.2 | 8 | 0 | 12.7 | 24.9 | 2.0 | XPO1 | colorectal cancer | Medium | 9 | 11 | 81.82 |  |  |  |  |  |  |  |  |  |
| Q13162 | Peroxiredoxin-4 | PRDX4 | ENSG00000123131 | 1.8 | 8 | 0 | 1.8 | 7 | 0 | 34.4 | 31.9 | 0.9 | PRDX4 | colorectal cancer | Medium | 7 | 11 | 63.64 |  |  |  |  |  |  |  |  |  |
| P07108 | Acyl-CoA-binding protein | DBI | ENSG00000155368 | 1.8 | 7 | 0 | 2.0 | 8 | 0 | 2.3 | 13.6 | 5.8 | DBI | colorectal cancer | Medium | 8 | 11 | 72.73 |  |  |  |  |  |  |  |  |  |
| P48643 | T-complex protein 1 subunit epsilon | CCT5 | ENSG00000150753 | 1.8 | 7 | 0 | 2.0 | 9 | 0 | 47.0 | 49.0 | 1.0 | CCT5 | colorectal cancer | Low | 6 | 11 | 54.55 |  |  |  |  |  |  |  |  |  |
| P60842 | Eukaryotic initiation factor 4A-I | EIF4A1 | ENSG00000161960 | 1.8 | 8 | 0 | 2.0 | 8 | 0 | 22.3 | 68.9 | 3.1 | EIF4A1 | colorectal cancer | Medium | 4 | 11 | 36.36 |  |  |  |  |  |  |  |  |  |
| P38646 | Stress-70 protein, mitochondrial | HSPA9 | ENSG00000113013 | 1.8 | 8 | 0 | 1.9 | 8 | 0 | 110.6 | 105.3 | 1.0 | HSPA9 | colorectal cancer | High | 11 | 11 | 100.00 |  |  |  |  |  |  |  |  |  |
| P31946 | 14-3-3 protein beta/alpha | YWHAB | ENSG00000166913 | 1.8 | 8 | 0 | 1.7 | 7 | 0 | 84.7 | 75.1 | 0.9 | YWHAB | colorectal cancer | Medium | 9 | 12 | 75.00 |  |  |  |  |  |  |  |  |  |
| P55072 | Transitional endoplasmic reticulum ATPase | VCP | ENSG00000165280 | 1.8 | 7 | 0 | 1.8 | 8 | 0 | 117.5 | 102.5 | 0.9 | VCP | colorectal cancer | High | 9 | 12 | 75.00 |  |  |  |  |  |  |  |  |  |
| Q04837 | Single-stranded DNA-binding protein, mitochondrial | SSBP1 | ENSG00000106028 | 1.7 | 7 | 0 | 2.0 | 9 | 0 | 16.7 | 16.7 | 1.0 | SSBP1 | colorectal cancer | High | 7 | 10 | 70.00 |  |  |  |  |  |  |  |  |  |
| P29401 | Transketolase | TKT | ENSG00000163931 | 1.7 | 8 | 0 | 2.0 | 8 | 0 | 1.5 | 101.5 | 68.0 | TKT | colorectal cancer | Medium | 10 | 12 | 83.33 |  |  |  |  |  |  |  |  |  |
| P39687 | Acidic leucine-rich nuclear phosphoprotein 32 family member A | ANP32A | ENSG00000140350 | 1.7 | 7 | 1 | 2.0 | 9 | 0 | 25.3 | 29.6 | 1.2 | ANP32A | colorectal cancer | Medium | 12 | 12 | 100.00 |  |  |  |  |  |  |  |  |  |
| P13639 | Elongation factor 2 | EEF2 | ENSG00000167658 | 1.7 | 7 | 0 | 1.8 | 8 | 0 | 182.1 | 174.8 | 1.0 | EEF2 | colorectal cancer | High | 10 | 11 | 90.91 |  |  |  |  |  |  |  |  |  |
| O14818 | Proteasome subunit alpha type-7 | PSMA7 | ENSG00000101182 | 1.7 | 7 | 0 | 1.8 | 9 | 0 | 20.6 | 17.7 | 0.9 | PSMA7 | colorectal cancer | Not detected | 6 | 12 | 50.00 |  |  |  |  |  |  |  |  |  |
| P14618 | Pyruvate kinase PKM | PKM | ENSG00000067225 | 1.7 | 8 | 0 | 1.6 | 7 | 0 | 6.4 | 222.1 | 34.5 | PKM | colorectal cancer | Medium | 6 | 11 | 54.55 |  |  |  |  |  |  |  |  |  |
| Q14697 | Neutral alpha-glucosidase AB | GANAB | ENSG00000089597 | 1.7 | 7 | 0 | 1.6 | 7 | 0 | 99.0 | 76.5 | 0.8 | GANAB | colorectal cancer | Medium | 8 | 11 | 72.73 |  |  |  |  |  |  |  |  |  |
| P18124 | 60S ribosomal protein L7 | RPL7 | ENSG00000147604 | 1.6 | 7 | 0 | 1.7 | 9 | 0 | 42.2 | 48.4 | 1.1 | RPL7 | colorectal cancer | Medium | 9 | 10 | 90.00 |  |  |  |  |  |  |  |  |  |
| O95336 | 6-phosphogluconolactonase | PGLS | ENSG00000130313 | 1.6 | 8 | 0 | 1.8 | 7 | 0 | 26.5 | 22.6 | 0.9 | PGLS | colorectal cancer | Medium | 10 | 12 | 83.33 |  |  |  |  |  |  |  |  |  |
| P06748 | Nucleophosmin | NPM1 | ENSG00000181163 | 1.6 | 7 | 0 | 2.1 | 10 | 0 | 1.1 | 48.3 | 42.4 | NPM1 | colorectal cancer | High | 12 | 12 | 100.00 |  |  |  |  |  |  |  |  |  |
| P26599 | Polypyrimidine tract-binding protein 1 | PTBP1 | ENSG00000011304 | 1.6 | 8 | 0 | 1.6 | 9 | 0 | 1.7 | 58.6 | 33.6 | PTBP1 | colorectal cancer | High | 10 | 12 | 83.33 |  |  |  |  |  |  |  |  |  |
| P78417 | Glutathione S-transferase omega-1 | GSTO1 | ENSG00000148834 | 1.6 | 7 | 0 | 2.1 | 8 | 0 | 1.0 | 26.4 | 26.2 | GSTO1 | colorectal cancer | Not detected | 12 | 12 | 100.00 |  |  |  |  |  |  |  |  |  |
| P52272 | Heterogeneous nuclear ribonucleoprotein M | HNRNPM | ENSG00000099783 | 1.5 | 7 | 0 | 1.6 | 7 | 0 | 52.9 | 70.5 | 1.3 | HNRNPM | colorectal cancer | High | 12 | 12 | 100.00 |  |  |  |  |  |  |  |  |  |
| P20618 | Proteasome subunit beta type-1 | PSMB1 | ENSG00000008018 | 1.5 | 7 | 0 | 1.9 | 10 | 0 | 19.0 | 15.7 | 0.8 | PSMB1 | colorectal cancer | Medium | 7 | 12 | 58.33 |  |  |  |  |  |  |  |  |  |
| P12277 | Creatine kinase B-type | CKB | ENSG00000166165 | 0.6 | 0 | 9 | 0.7 | 0 | 9 | 158.6 | 38.2 | 0.2 | CKB | colorectal cancer | Not detected | 10 | 12 | 83.33 |  |  |  |  |  |  |  |  |  |
| P51884 | Lumican | LUM | * | 0.6 | 0 | 9 | 0.6 | 0 | 8 | 74.3 | 47.3 | 0.6 | * | * | * | * | * | * |  |  |  |  |  |  |  |  |  |
| Q05682 | Caldesmon | CALD1 | ENSG00000122786 | 0.5 | 0 | 9 | 0.7 | 0 | 7 | 41.6 | 63.3 | 1.5 | CALD1 | colorectal cancer | Not detected | 12 | 12 | 100.00 |  |  |  |  |  |  |  |  |  |
| Q9NZN4 | EH domain-containing protein 2 | EHD2 | ENSG00000024422 | 0.5 | 0 | 9 | 0.6 | 0 | 8 | 40.3 | 19.3 | 0.5 | EHD2 | colorectal cancer | Not detected | 12 | 12 | 100.00 |  |  |  |  |  |  |  |  |  |
| P00915 | Carbonic anhydrase 1 | CA1 | ENSG00000133742 | 0.5 | 0 | 9 | 0.7 | 0 | 7 | 181.6 | 17.1 | 0.1 | CA1 | colorectal cancer | Not detected | 11 | 11 | 100.00 |  |  |  |  |  |  |  |  |  |
| Q8WX93 | Palladin | PALLD | * | 0.5 | 0 | 11 | 0.6 | 0 | 7 | 15.2 | 21.7 | 1.4 | * | * | * | * | * | * |  |  |  |  |  |  |  |  |  |
| Q16853 | Membrane primary amine oxidase | AOC3 | ENSG00000131471 | 0.5 | 0 | 8 | 0.5 | 0 | 11 | 40.0 | 16.3 | 0.4 | AOC3 | colorectal cancer | Not detected | 12 | 12 | 100.00 |  |  |  |  |  |  |  |  |  |
| P07585 | Decorin | DCN | ENSG00000011465 | 0.5 | 0 | 10 | 0.5 | 0 | 8 | 55.4 | 30.6 | 0.6 | DCN | colorectal cancer | Not detected | 8 | 10 | 80.00 |  |  |  |  |  |  |  |  |  |
| P04792 | Heat shock protein beta-1 | HSPB1 | ENSG00000106211 | 0.5 | 0 | 9 | 0.6 | 0 | 9 | 84.0 | 71.1 | 0.8 | HSPB1 | colorectal cancer | Low | 5 | 10 | 50.00 | HSPB1 | colorectal cancer | Not detected | 5 | 10 | 50.00 |  |  |  |
| P51888 | Prolargin | PRELP | * | 0.5 | 0 | 10 | 0.5 | 0 | 9 | 20.1 | 22.9 | 1.1 | * | * | * | * | * | * |  |  |  |  |  |  |  |  |  |
| P21333 | Filamin-A | FLNA | ENSG00000196924 | 0.5 | 0 | 11 | 0.6 | 0 | 9 | 476.0 | 515.2 | 1.1 | FLNA | colorectal cancer | Not detected | 8 | 10 | 80.00 |  |  |  |  |  |  |  |  |  |
| P00918 | Carbonic anhydrase 2 | CA2 | ENSG00000104267 | 0.5 | 0 | 10 | 0.5 | 0 | 9 | 226.7 | 30.9 | 0.1 | CA2 | colorectal cancer | Not detected | 12 | 12 | 100.00 |  |  |  |  |  |  |  |  |  |
| Q15746 | Myosin light chain kinase, smooth muscle | MYLK | ENSG00000065534 | 0.4 | 0 | 11 | 0.6 | 0 | 8 | 1.9 | 23.4 | 12.2 | MYLK | colorectal cancer | Not detected | 11 | 11 | 100.00 |  |  |  |  |  |  |  |  |  |
| P21291 | Cysteine and glycine-rich protein 1 | CSRP1 | ENSG00000159176 | 0.4 | 0 | 11 | 0.4 | 0 | 10 | 29.2 | 37.7 | 1.3 | CSRP1 | colorectal cancer | Medium | 5 | 12 | 41.67 |  |  |  |  |  |  |  |  |  |
| O14558 | Heat shock protein beta-6 | HSPB6 | ENSG00000004776 | 0.4 | 0 | 9 | 0.4 | 0 | 8 | 5.8 | 3.7 | 0.6 | HSPB6 | colorectal cancer | Not detected | 12 | 12 | 100.00 |  |  |  |  |  |  |  |  |  |
| Q03135 | Caveolin-1 | CAV1 | ENSG00000105974 | 0.4 | 0 | 10 | 0.4 | 0 | 8 | 5.5 | 7.1 | 1.3 | CAV1 | colorectal cancer | Not detected | 12 | 12 | 100.00 |  |  |  |  |  |  |  |  |  |
| P35749 | Myosin-11 | MYH11 | ENSG00000133392 | 0.3 | 0 | 11 | 0.5 | 0 | 10 | 340.9 | 266.1 | 0.8 | MYH11 | colorectal cancer | Not detected | 12 | 12 | 100.00 |  |  |  |  |  |  |  |  |  |
| Q01995 | Transgelin | TAGLN | ENSG00000149591 | 0.3 | 0 | 11 | 0.3 | 0 | 11 | 164.9 | 181.1 | 1.1 | TAGLN | colorectal cancer | Not detected | 11 | 11 | 100.00 |  |  |  |  |  |  |  |  |  |
| P51911 | Calponin-1 | CNN1 | ENSG00000130176 | 0.3 | 0 | 11 | 0.3 | 0 | 11 | 28.5 | 41.0 | 1.4 | CNN1 | colorectal cancer | Not detected | 10 | 10 | 100.00 |  |  |  |  |  |  |  |  |  |
| P07951 | Tropomyosin beta chain | TPM2 | ENSG00000198467 | 0.2 | 0 | 11 | 0.3 | 0 | 8 | 80.3 | 66.0 | 0.8 | TPM2 | colorectal cancer | High | 8 | 10 | 80.00 |  |  |  |  |  |  |  |  |  |
| P24844 | Myosin regulatory light polypeptide 9 | MYL9 | ENSG00000101335 | 0.2 | 0 | 11 | 0.4 | 0 | 8 | 26.8 | 42.9 | 1.6 | MYL9 | colorectal cancer | Not detected | 9 | 11 | 81.82 |  |  |  |  |  |  |  |  |  |
| Q99879 | Histone H2B type 1-M | HIST1H2BM | ENSG00000273703 | * | * | * | 1.8 | 7 | 1 | * | * | * | HIST1H2BM | colorectal cancer | High | 7 | 10 | 70.00 |  |  |  |  |  |  |  |  |  |
| O60812 | Heterogeneous nuclear ribonucleoprotein C-like 1 | HNRNPCL1 | * | * | * | * | 2.3 | 7 | 0 | * | * | * | * | * | * | * | * | * |  |  |  |  |  |  |  |  |  |
| P22392 | Nucleoside diphosphate kinase B | NME2 | ENSG00000243678 | * | * | * | 1.9 | 8 | 0 | * | * | * | NME2 | colorectal cancer | Medium | 11 | 12 | 91.67 |  |  |  |  |  |  |  |  |  |
| P08708 | 40S ribosomal protein S17 | RPS17 | ENSG00000182774 | * | * | * | 1.9 | 7 | 0 | * | * | * | RPS17 | colorectal cancer | Medium | 9 | 11 | 81.82 |  |  |  |  |  |  |  |  |  |
| Q08EQ4 | Thymosin beta-4-like protein 3 | TYB4L | * | * | * | * | 2.1 | 9 | 0 | * | * | * | * | * | * | * | * | * |  |  |  |  |  |  |  |  |  |
| P0CG48 | Polyubiquitin-C | UBC | ENSG00000150991 | * | * | * | 1.6 | 7 | 0 | * | * | * | UBC | colorectal cancer | Medium | 8 | 11 | 72.73 |  |  |  |  |  |  |  |  |  |
| P06744 | Glucose-6-phosphate isomerase | GPI | ENSG00000105220 | * | * | * | 1.7 | 7 | 0 | 2.1 | 98.6 | 46.4 | GPI | colorectal cancer | Medium | 6 | 12 | 50.00 |  |  |  |  |  |  |  |  |  |
| P23381 | Tryptophan--tRNA ligase, cytoplasmic | WARS | * | * | * | * | 1.7 | 7 | 0 | 2.1 | 46.9 | 21.8 | * | * | * | * | * | * |  |  |  |  |  |  |  |  |  |
| P51659 | Peroxisomal multifunctional enzyme type 2 | HSD17B4 | ENSG00000133835 | * | * | * | 1.8 | 8 | 1 | 8.2 | 47.2 | 5.7 | HSD17B4 | colorectal cancer | Medium | 10 | 12 | 83.33 |  |  |  |  |  |  |  |  |  |
| P59998 | Actin-related protein 2/3 complex subunit 4 | ARPC4 | * | * | * | * | 2.0 | 7 | 0 | 3.6 | 19.5 | 5.5 | * | * | * | * | * | * |  |  |  |  |  |  |  |  |  |
| P04179 | Superoxide dismutase [Mn], mitochondrial | SOD2 | ENSG00000112096 | * | * | * | 1.9 | 8 | 0 | 8.1 | 27.4 | 3.4 | SOD2 | colorectal cancer | Medium | 7 | 12 | 58.33 |  |  |  |  |  |  |  |  |  |
| P06733 | Alpha-enolase | ENO1 | ENSG00000074800 | * | * | * | 1.6 | 7 | 0 | 52.7 | 155.2 | 2.9 | ENO1 | colorectal cancer | Medium | 12 | 12 | 100.00 |  |  |  |  |  |  |  |  |  |
| P04114 | Apolipoprotein B-100 | APOB | ENSG00000084674 | * | * | * | 2.2 | 7 | 0 | 22.4 | 61.2 | 2.7 | APOB | colorectal cancer | Not detected | 7 | 10 | 70.00 |  |  |  |  |  |  |  |  |  |
| Q96KP4 | Cytosolic non-specific dipeptidase | CNDP2 | ENSG00000133313 | * | * | * | 2.1 | 8 | 0 | 14.2 | 30.3 | 2.1 | CNDP2 | colorectal cancer | Medium | 8 | 11 | 72.73 |  |  |  |  |  |  |  |  |  |
| P20700 | Lamin-B1 | LMNB1 | ENSG00000113368 | * | * | * | 1.8 | 9 | 0 | 36.7 | 58.8 | 1.6 | LMNB1 | colorectal cancer | Medium | 10 | 12 | 83.33 |  |  |  |  |  |  |  |  |  |
| P36578 | 60S ribosomal protein L4 | RPL4 | ENSG00000174444 | * | * | * | 2.3 | 9 | 0 | 67.8 | 88.2 | 1.3 | RPL4 | colorectal cancer | Medium | 9 | 11 | 81.82 |  |  |  |  |  |  |  |  |  |
| P09972 | Fructose-bisphosphate aldolase C | ALDOC | ENSG00000109107 | * | * | * | 1.8 | 9 | 0 | 37.3 | 48.2 | 1.3 | ALDOC | colorectal cancer | Not detected | 12 | 12 | 100.00 |  |  |  |  |  |  |  |  |  |
| P00491 | Purine nucleoside phosphorylase | PNP | ENSG00000198805 | * | * | * | 2.7 | 7 | 0 | 20.1 | 25.2 | 1.3 | PNP | colorectal cancer | Low | 5 | 11 | 45.45 | PNP | colorectal cancer | Medium | 5 | 11 | 45.45 |  |  |  |
| P15880 | 40S ribosomal protein S2 | RPS2 | ENSG00000140988 | * | * | * | 2.1 | 8 | 0 | 36.4 | 45.1 | 1.2 | RPS2 | colorectal cancer | Not detected | 9 | 12 | 75.00 |  |  |  |  |  |  |  |  |  |
| Q00839 | Heterogeneous nuclear ribonucleoprotein U | HNRNPU | ENSG00000153187 | * | * | * | 2.0 | 9 | 0 | 78.9 | 97.5 | 1.2 | HNRNPU | colorectal cancer | High | 6 | 11 | 54.55 |  |  |  |  |  |  |  |  |  |
| P46783 | 40S ribosomal protein S10 | RPS10 | ENSG00000124614 | * | * | * | 2.3 | 7 | 2 | 15.9 | 19.6 | 1.2 | RPS10 | colorectal cancer | Medium | 11 | 12 | 91.67 |  |  |  |  |  |  |  |  |  |
| O00299 | Chloride intracellular channel protein 1 | CLIC1 | ENSG00000213719 | * | * | * | 1.8 | 8 | 1 | 71.0 | 78.7 | 1.1 | CLIC1 | colorectal cancer | Low | 6 | 12 | 50.00 |  |  |  |  |  |  |  |  |  |
| P01019 | Angiotensinogen | AGT | ENSG00000135744 | * | * | * | 2.0 | 8 | 0 | 8.2 | 9.0 | 1.1 | AGT | colorectal cancer | Low | 10 | 12 | 83.33 |  |  |  |  |  |  |  |  |  |
| P51991 | Heterogeneous nuclear ribonucleoprotein A3 | HNRNPA3 | ENSG00000170144 | * | * | * | 1.9 | 8 | 0 | 51.3 | 55.9 | 1.1 | HNRNPA3 | colorectal cancer | High | 11 | 11 | 100.00 |  |  |  |  |  |  |  |  |  |
| P78371 | T-complex protein 1 subunit beta | CCT2 | ENSG00000166226 | * | * | * | 1.8 | 8 | 1 | 50.9 | 55.4 | 1.1 | CCT2 | colorectal cancer | Low | 9 | 12 | 75.00 |  |  |  |  |  |  |  |  |  |
| Q13838 | Spliceosome RNA helicase DDX39B | DDX39B | ENSG00000198563 | * | * | * | 2.4 | 8 | 0 | 38.3 | 40.1 | 1.0 | DDX39B | colorectal cancer | Not detected | 6 | 10 | 60.00 |  |  |  |  |  |  |  |  |  |
| P22626 | Heterogeneous nuclear ribonucleoproteins A2/B1 | HNRNPA2B1 | ENSG00000122566 | * | * | * | 2.1 | 9 | 0 | 95.9 | 100.1 | 1.0 | HNRNPA2B1 | colorectal cancer | High | 9 | 12 | 75.00 |  |  |  |  |  |  |  |  |  |
| P49257 | Protein ERGIC-53 | LMAN1 | ENSG00000074695 | * | * | * | 2.0 | 7 | 0 | 17.6 | 18.3 | 1.0 | LMAN1 | colorectal cancer | High | 6 | 11 | 54.55 |  |  |  |  |  |  |  |  |  |
| P27824 | Calnexin | CANX | ENSG00000127022 | * | * | * | 1.7 | 9 | 0 | 76.4 | 77.8 | 1.0 | CANX | colorectal cancer | High | 10 | 11 | 90.91 |  |  |  |  |  |  |  |  |  |
| Q13418 | Integrin-linked protein kinase | ILK | ENSG00000166333 | * | * | * | 0.5 | 0 | 7 | 15.8 | 15.9 | 1.0 | ILK | colorectal cancer | Not detected | 8 | 11 | 72.73 |  |  |  |  |  |  |  |  |  |
| P04075 | Fructose-bisphosphate aldolase A | ALDOA | ENSG00000149925 | * | * | * | 1.5 | 7 | 0 | 178.1 | 171.3 | 1.0 | ALDOA | colorectal cancer | Low | 6 | 12 | 50.00 |  |  |  |  |  |  |  |  |  |
| Q15149 | Plectin | PLEC | ENSG00000178209 | * | * | * | 1.7 | 8 | 0 | 375.8 | 349.6 | 0.9 | PLEC | colorectal cancer | High | 9 | 12 | 75.00 |  |  |  |  |  |  |  |  |  |
| Q15084 | Protein disulfide-isomerase A6 | PDIA6 | ENSG00000143870 | * | * | * | 1.8 | 8 | 0 | 85.4 | 78.7 | 0.9 | PDIA6 | colorectal cancer | Medium | 12 | 12 | 100.00 |  |  |  |  |  |  |  |  |  |
| P00387 | NADH-cytochrome b5 reductase 3 | CYB5R3 | ENSG00000100243 | * | * | * | 1.7 | 7 | 0 | 28.5 | 26.2 | 0.9 | CYB5R3 | colorectal cancer | Low | 6 | 12 | 50.00 | CYB5R3 | colorectal cancer | Medium | 6 | 12 | 50.00 |  |  |  |
| P35268 | 60S ribosomal protein L22 | RPL22 | * | * | * | * | 1.8 | 7 | 0 | 11.4 | 10.4 | 0.9 | * | * | * | * | * | * |  |  |  |  |  |  |  |  |  |
| P35232 | Prohibitin | PHB | ENSG00000167085 | * | * | * | 1.7 | 7 | 0 | 48.3 | 43.7 | 0.9 | PHB | colorectal cancer | High | 9 | 9 | 100.00 |  |  |  |  |  |  |  |  |  |
| P42704 | Leucine-rich PPR motif-containing protein, mitochondrial | LRPPRC | ENSG00000138095 | * | * | * | 1.7 | 7 | 1 | 136.4 | 123.1 | 0.9 | LRPPRC | colorectal cancer | High | 10 | 10 | 100.00 |  |  |  |  |  |  |  |  |  |
| P07237 | Protein disulfide-isomerase | P4HB | ENSG00000185624 | * | * | * | 1.9 | 7 | 0 | 144.1 | 127.3 | 0.9 | P4HB | colorectal cancer | Medium | 9 | 12 | 75.00 |  |  |  |  |  |  |  |  |  |
| P05387 | 60S acidic ribosomal protein P2 | RPLP2 | ENSG00000177600 | * | * | * | 1.7 | 7 | 0 | 43.9 | 38.5 | 0.9 | RPLP2 | colorectal cancer | Medium | 8 | 10 | 80.00 |  |  |  |  |  |  |  |  |  |
| Q562R1 | Beta-actin-like protein 2 | ACTBL2 | ENSG00000169067 | * | * | * | 0.6 | 0 | 7 | 353.0 | 308.4 | 0.9 | ACTBL2 | colorectal cancer | High | 7 | 12 | 58.33 |  |  |  |  |  |  |  |  |  |
| P31939 | Bifunctional purine biosynthesis protein PURH | ATIC | ENSG00000138363 | * | * | * | 1.5 | 7 | 2 | 57.0 | 47.7 | 0.8 | ATIC | colorectal cancer | High | 7 | 12 | 58.33 |  |  |  |  |  |  |  |  |  |
| Q9P2E9 | Ribosome-binding protein 1 | RRBP1 | ENSG00000125844 | * | * | * | 1.6 | 8 | 0 | 92.1 | 76.8 | 0.8 | RRBP1 | colorectal cancer | High | 10 | 11 | 90.91 |  |  |  |  |  |  |  |  |  |
| P61160 | Actin-related protein 2 | ACTR2 | ENSG00000138071 | * | * | * | 0.6 | 0 | 8 | 53.9 | 41.1 | 0.8 | ACTR2 | colorectal cancer | Medium | 9 | 10 | 90.00 |  |  |  |  |  |  |  |  |  |
| P54819 | Adenylate kinase 2, mitochondrial | AK2 | ENSG00000004455 | * | * | * | 1.7 | 7 | 1 | 39.8 | 30.2 | 0.8 | AK2 | colorectal cancer | Medium | 9 | 11 | 81.82 |  |  |  |  |  |  |  |  |  |
| Q06210 | Glutamine--fructose-6-phosphate aminotransferase [isomerizing] 1 | GFPT1 | * | * | * | * | 1.8 | 7 | 1 | 69.3 | 51.2 | 0.7 | * | * | * | * | * | * |  |  |  |  |  |  |  |  |  |
| P14550 | Alcohol dehydrogenase [NADP(+)] | AKR1A1 | ENSG00000117448 | * | * | * | 1.7 | 7 | 0 | 55.2 | 38.4 | 0.7 | AKR1A1 | colorectal cancer | Not detected | 10 | 11 | 90.91 |  |  |  |  |  |  |  |  |  |
| Q14764 | Major vault protein | MVP | ENSG00000013364 | * | * | * | 1.7 | 7 | 0 | 138.0 | 94.7 | 0.7 | MVP | colorectal cancer | High | 6 | 12 | 50.00 | MVP | colorectal cancer | Medium | 6 | 12 | 50.00 |  |  |  |
| P19075 | Tetraspanin-8 | TSPAN8 | ENSG00000127324 | * | * | * | 2.3 | 8 | 0 | 8.2 | 5.4 | 0.7 | TSPAN8 | colorectal cancer | Medium | 5 | 12 | 41.67 |  |  |  |  |  |  |  |  |  |
| P46940 | Ras GTPase-activating-like protein IQGAP1 | IQGAP1 | ENSG00000140575 | * | * | * | 1.6 | 7 | 0 | 215.0 | 140.4 | 0.7 | IQGAP1 | colorectal cancer | High | 7 | 11 | 63.64 |  |  |  |  |  |  |  |  |  |
| P55327 | Tumor protein D52 | TPD52 | ENSG00000076554 | * | * | * | 2.1 | 7 | 0 | 26.4 | 16.9 | 0.6 | TPD52 | colorectal cancer | Medium | 6 | 10 | 60.00 |  |  |  |  |  |  |  |  |  |
| Q9HC38 | Glyoxalase domain-containing protein 4 | GLOD4 | ENSG00000167699 | * | * | * | 1.8 | 9 | 0 | 25.0 | 15.5 | 0.6 | GLOD4 | colorectal cancer | High | 7 | 12 | 58.33 |  |  |  |  |  |  |  |  |  |
| P14923 | Junction plakoglobin | JUP | ENSG00000173801 | * | * | * | 2.0 | 7 | 0 | 99.4 | 59.4 | 0.6 | JUP | colorectal cancer | High | 9 | 10 | 90.00 |  |  |  |  |  |  |  |  |  |
| Q8WU39 | Marginal zone B- and B1-cell-specific protein | MZB1 | ENSG00000170476 | * | * | * | 1.8 | 8 | 0 | 46.4 | 12.4 | 0.3 | MZB1 | colorectal cancer | Not detected | 11 | 11 | 100.00 |  |  |  |  |  |  |  |  |  |
| P37108 | Signal recognition particle 14 kDa protein | SRP14 | * | 3.4 | 7 | 0 | * | * | * | 11.3 | 10.2 | 0.9 | * | * | * | * | * | * |  |  |  |  |  |  |  |  |  |
| Q93009 | Ubiquitin carboxyl-terminal hydrolase 7 | USP7 | ENSG00000187555 | 3.2 | 8 | 0 | * | * | * | 15.9 | 17.7 | 1.1 | USP7 | colorectal cancer | Low | 9 | 12 | 75.00 |  |  |  |  |  |  |  |  |  |
| P62244 | 40S ribosomal protein S15a | RPS15A | ENSG00000134419 | 3.0 | 8 | 0 | * | * | * | 27.6 | 25.9 | 0.9 | RPS15A | colorectal cancer | Medium | 11 | 12 | 91.67 |  |  |  |  |  |  |  |  |  |
| P02792 | Ferritin light chain | FTL | ENSG00000087086 | 3.0 | 9 | 0 | * | * | * | 14.4 | 8.5 | 0.6 | FTL | colorectal cancer | Not detected | 6 | 12 | 50.00 |  |  |  |  |  |  |  |  |  |
| P63313 | Thymosin beta-10 | TMSB10 | * | 2.9 | 8 | 0 | * | * | * | 19.4 | 6.2 | 0.3 | * | * | * | * | * | * |  |  |  |  |  |  |  |  |  |
| P61604 | 10 kDa heat shock protein, mitochondrial | HSPE1 | ENSG00000115541 | 2.9 | 9 | 0 | * | * | * | 27.3 | 56.9 | 2.1 | HSPE1 | colorectal cancer | High | 11 | 12 | 91.67 |  |  |  |  |  |  |  |  |  |
| O60749 | Sorting nexin-2 | SNX2 | ENSG00000205302 | 2.7 | 8 | 0 | * | * | * | 24.9 | 15.2 | 0.6 | SNX2 | colorectal cancer | Medium | 7 | 12 | 58.33 |  |  |  |  |  |  |  |  |  |
| Q56VL3 | OCIA domain-containing protein 2 | OCIAD2 | ENSG00000145247 | 2.7 | 9 | 0 | * | * | * | 6.9 | 10.0 | 1.5 | OCIAD2 | colorectal cancer | High | 5 | 12 | 41.67 | OCIAD2 | colorectal cancer | Medium | 5 | 12 | 41.67 |  |  |  |
| P62249 | 40S ribosomal protein S16 | RPS16 | ENSG00000105193 | 2.7 | 8 | 1 | * | * | * | 31.0 | 31.9 | 1.0 | RPS16 | colorectal cancer | Medium | 7 | 10 | 70.00 |  |  |  |  |  |  |  |  |  |
| Q92597 | Protein NDRG1 | NDRG1 | ENSG00000104419 | 2.7 | 8 | 0 | * | * | * | 5.5 | 14.3 | 2.6 | NDRG1 | colorectal cancer | High | 9 | 12 | 75.00 |  |  |  |  |  |  |  |  |  |
| P22102 | Trifunctional purine biosynthetic protein adenosine-3 | GART | ENSG00000159131 | 2.6 | 7 | 0 | * | * | * | 6.5 | 27.8 | 4.3 | GART | colorectal cancer | Medium | 6 | 11 | 54.55 |  |  |  |  |  |  |  |  |  |
| P09327 | Villin-1 | VIL1 | ENSG00000127831 | 2.6 | 9 | 0 | * | * | * | 106.5 | 63.0 | 0.6 | VIL1 | colorectal cancer | Medium | 9 | 12 | 75.00 |  |  |  |  |  |  |  |  |  |
| P35637 | RNA-binding protein FUS | FUS | ENSG00000089280 | 2.6 | 7 | 1 | * | * | * | 16.8 | 17.2 | 1.0 | FUS | colorectal cancer | High | 11 | 11 | 100.00 |  |  |  |  |  |  |  |  |  |
| P13164 | Interferon-induced transmembrane protein 1 | IFITM1 | ENSG00000185885 | 2.5 | 10 | 0 | * | * | * | 1.1 | 6.6 | 6.0 | IFITM1 | colorectal cancer | High | 7 | 11 | 63.64 |  |  |  |  |  |  |  |  |  |
| P00492 | Hypoxanthine-guanine phosphoribosyltransferase | HPRT1 | ENSG00000165704 | 2.5 | 8 | 0 | * | * | * | 10.0 | 3.8 | 0.4 | HPRT1 | colorectal cancer | High | 5 | 11 | 45.45 |  |  |  |  |  |  |  |  |  |
| Q9UBR2 | Cathepsin Z | CTSZ | ENSG00000101160 | 2.5 | 7 | 0 | * | * | * | 20.8 | 8.0 | 0.4 | CTSZ | colorectal cancer | High | 4 | 12 | 33.33 | CTSZ | colorectal cancer | Not detected | 4 | 12 | 33.33 |  |  |  |
| P14780 | Matrix metalloproteinase-9 | MMP9 | ENSG00000100985 | 2.5 | 8 | 0 | * | * | * | 1.1 | 10.0 | 9.2 | MMP9 | colorectal cancer | Not detected | 12 | 12 | 100.00 |  |  |  |  |  |  |  |  |  |
| P51665 | 26S proteasome non-ATPase regulatory subunit 7 | PSMD7 | ENSG00000103035 | 2.4 | 7 | 0 | * | * | * | 13.3 | 10.4 | 0.8 | PSMD7 | colorectal cancer | Medium | 9 | 12 | 75.00 |  |  |  |  |  |  |  |  |  |
| O43169 | Cytochrome b5 type B | CYB5B | ENSG00000103018 | 2.4 | 7 | 0 | * | * | * | 9.0 | 9.1 | 1.0 | CYB5B | colorectal cancer | High | 12 | 12 | 100.00 |  |  |  |  |  |  |  |  |  |
| Q07020 | 60S ribosomal protein L18 | RPL18 | ENSG00000063177 | 2.4 | 8 | 0 | * | * | * | 3.4 | 33.5 | 10.0 | RPL18 | colorectal cancer | Medium | 11 | 12 | 91.67 |  |  |  |  |  |  |  |  |  |
| Q53GQ0 | Very-long-chain 3-oxoacyl-CoA reductase | HSD17B12 | ENSG00000149084 | 2.4 | 10 | 0 | * | * | * | 13.9 | 16.9 | 1.2 | HSD17B12 | colorectal cancer | High | 6 | 12 | 50.00 | HSD17B12 | colorectal cancer | Medium | 6 | 12 | 50.00 |  |  |  |
| P23246 | Splicing factor, proline- and glutamine-rich | SFPQ | ENSG00000116560 | 2.4 | 7 | 0 | * | * | * | 37.0 | 43.3 | 1.2 | SFPQ | colorectal cancer | High | 9 | 9 | 100.00 |  |  |  |  |  |  |  |  |  |
| P80188 | Neutrophil gelatinase-associated lipocalin | LCN2 | ENSG00000148346 | 2.4 | 7 | 0 | * | * | * | 1.8 | 17.1 | 9.7 | LCN2 | colorectal cancer | Not detected | 5 | 11 | 45.45 |  |  |  |  |  |  |  |  |  |
| P62851 | 40S ribosomal protein S25 | RPS25 | ENSG00000118181 | 2.3 | 8 | 0 | * | * | * | 20.5 | 21.5 | 1.0 | RPS25 | colorectal cancer | High | 12 | 12 | 100.00 |  |  |  |  |  |  |  |  |  |
| P80511 | Protein S100-A12 | S100A12 | ENSG00000163221 | 2.3 | 8 | 1 | * | * | * | 1.0 | 4.4 | 4.3 | S100A12 | colorectal cancer | Not detected | 8 | 9 | 88.89 |  |  |  |  |  |  |  |  |  |
| Q92841 | Probable ATP-dependent RNA helicase DDX17 | DDX17 | ENSG00000100201 | 2.3 | 8 | 0 | * | * | * | 58.5 | 56.5 | 1.0 | DDX17 | colorectal cancer | Medium | 9 | 11 | 81.82 |  |  |  |  |  |  |  |  |  |
| P13797 | Plastin-3 | PLS3 | ENSG00000102024 | 2.3 | 7 | 1 | * | * | * | 41.7 | 45.6 | 1.1 | PLS3 | colorectal cancer | Medium | 8 | 11 | 72.73 |  |  |  |  |  |  |  |  |  |
| O43143 | Pre-mRNA-splicing factor ATP-dependent RNA helicase DHX15 | DHX15 | ENSG00000109606 | 2.3 | 8 | 0 | * | * | * | 17.8 | 29.4 | 1.7 | DHX15 | colorectal cancer | Medium | 6 | 12 | 50.00 |  |  |  |  |  |  |  |  |  |
| Q99439 | Calponin-2 | CNN2 | ENSG00000064666 | 2.3 | 8 | 0 | * | * | * | 5.8 | 15.9 | 2.7 | CNN2 | colorectal cancer | Not detected | 12 | 12 | 100.00 |  |  |  |  |  |  |  |  |  |
| P62328 | Thymosin beta-4 | TMSB4X | ENSG00000205542 | 2.2 | 8 | 0 | * | * | * | 85.0 | 21.1 | 0.2 | TMSB4X | colorectal cancer | Low | 7 | 12 | 58.33 |  |  |  |  |  |  |  |  |  |
| Q15233 | Non-POU domain-containing octamer-binding protein | NONO | ENSG00000147140 | 2.2 | 7 | 0 | * | * | * | 4.2 | 33.3 | 7.9 | NONO | colorectal cancer | High | 10 | 10 | 100.00 |  |  |  |  |  |  |  |  |  |
| P06454 | Prothymosin alpha | PTMA | ENSG00000187514 | 2.2 | 9 | 1 | * | * | * | 8.3 | 3.7 | 0.5 | PTMA | colorectal cancer | Medium | 10 | 11 | 90.91 |  |  |  |  |  |  |  |  |  |
| P15531 | Nucleoside diphosphate kinase A | NME1 | ENSG00000239672 | 2.2 | 8 | 0 | * | * | * | 9.0 | 43.3 | 4.8 | NME1 | colorectal cancer | Medium | 9 | 12 | 75.00 |  |  |  |  |  |  |  |  |  |
| P62847 | 40S ribosomal protein S24 | RPS24 | ENSG00000138326 | 2.1 | 8 | 0 | * | * | * | 13.9 | 14.8 | 1.1 | RPS24 | colorectal cancer | Low | 4 | 9 | 44.44 |  |  |  |  |  |  |  |  |  |
| O15143 | Actin-related protein 2/3 complex subunit 1B | ARPC1B | ENSG00000130429 | 2.1 | 8 | 0 | * | * | * | 27.3 | 33.1 | 1.2 | ARPC1B | colorectal cancer | Low | 9 | 12 | 75.00 |  |  |  |  |  |  |  |  |  |
| P62081 | 40S ribosomal protein S7 | RPS7 | ENSG00000171863 | 2.1 | 7 | 1 | * | * | * | 13.7 | 17.0 | 1.2 | RPS7 | colorectal cancer | High | 9 | 11 | 81.82 |  |  |  |  |  |  |  |  |  |
| P09651 | Heterogeneous nuclear ribonucleoprotein A1 | HNRNPA1 | ENSG00000135486 | 2.1 | 9 | 0 | * | * | * | 35.8 | 89.7 | 2.5 | HNRNPA1 | colorectal cancer | High | 11 | 11 | 100.00 |  |  |  |  |  |  |  |  |  |
| Q9UBQ7 | Glyoxylate reductase/hydroxypyruvate reductase | GRHPR | ENSG00000137106 | 2.1 | 7 | 0 | * | * | * | 20.6 | 16.6 | 0.8 | GRHPR | colorectal cancer | Not detected | 7 | 11 | 63.64 |  |  |  |  |  |  |  |  |  |
| P62318 | Small nuclear ribonucleoprotein Sm D3 | SNRPD3 | ENSG00000100028 | 2.1 | 9 | 0 | * | * | * | 6.0 | 6.6 | 1.1 | SNRPD3 | colorectal cancer | High | 6 | 10 | 60.00 |  |  |  |  |  |  |  |  |  |
| P62829 | 60S ribosomal protein L23 | RPL23 | ENSG00000125691 | 2.1 | 9 | 0 | * | * | * | 29.0 | 32.8 | 1.1 | RPL23 | colorectal cancer | High | 6 | 10 | 60.00 |  |  |  |  |  |  |  |  |  |
| P62424 | 60S ribosomal protein L7a | RPL7A | * | 2.1 | 9 | 0 | * | * | * | 43.8 | 49.9 | 1.1 | * | * | * | * | * | * |  |  |  |  |  |  |  |  |  |
| P07910 | Heterogeneous nuclear ribonucleoproteins C1/C2 | HNRNPC | ENSG00000092199 | 2.1 | 8 | 0 | * | * | * | 62.5 | 66.9 | 1.1 | HNRNPC | colorectal cancer | High | 11 | 11 | 100.00 |  |  |  |  |  |  |  |  |  |
| P62263 | 40S ribosomal protein S14 | RPS14 | ENSG00000164587 | 2.1 | 8 | 1 | * | * | * | 26.0 | 25.2 | 1.0 | RPS14 | colorectal cancer | High | 7 | 11 | 63.64 |  |  |  |  |  |  |  |  |  |
| P61313 | 60S ribosomal protein L15 | RPL15 | * | 2.1 | 7 | 0 | * | * | * | 15.5 | 32.3 | 2.1 | * | * | * | * | * | * |  |  |  |  |  |  |  |  |  |
| Q8NC51 | Plasminogen activator inhibitor 1 RNA-binding protein | SERBP1 | ENSG00000142864 | 2.1 | 8 | 0 | * | * | * | 1.0 | 25.6 | 25.4 | SERBP1 | colorectal cancer | Medium | 8 | 12 | 66.67 |  |  |  |  |  |  |  |  |  |
| P19971 | Thymidine phosphorylase | TYMP | ENSG00000025708 | 2.1 | 9 | 1 | * | * | * | 3.5 | 37.0 | 10.5 | TYMP | colorectal cancer | Low | 4 | 12 | 33.33 |  |  |  |  |  |  |  |  |  |
| Q00688 | Peptidyl-prolyl cis-trans isomerase FKBP3 | FKBP3 | ENSG00000100442 | 2.1 | 7 | 0 | * | * | * | 14.3 | 10.7 | 0.7 | FKBP3 | colorectal cancer | Medium | 8 | 11 | 72.73 |  |  |  |  |  |  |  |  |  |
| P08238 | Heat shock protein HSP 90-beta | HSP90AB1 | ENSG00000096384 | 2.1 | 9 | 0 | * | * | * | 193.8 | 256.4 | 1.3 | HSP90AB1 | colorectal cancer | Medium | 7 | 12 | 58.33 |  |  |  |  |  |  |  |  |  |
| Q92820 | Gamma-glutamyl hydrolase | GGH | ENSG00000137563 | 2.1 | 7 | 1 | * | * | * | 21.5 | 15.9 | 0.7 | GGH | colorectal cancer | High | 11 | 12 | 91.67 |  |  |  |  |  |  |  |  |  |
| Q86V81 | THO complex subunit 4 | ALYREF | ENSG00000183684 | 2.1 | 7 | 0 | * | * | * | 11.3 | 15.2 | 1.3 | ALYREF | colorectal cancer | High | 12 | 12 | 100.00 |  |  |  |  |  |  |  |  |  |
| P18621 | 60S ribosomal protein L17 | RPL17 | ENSG00000265681 | 2.1 | 8 | 1 | * | * | * | * | * | * | RPL17 | colorectal cancer | Medium | 8 | 11 | 72.73 |  |  |  |  |  |  |  |  |  |
| P08574 | Cytochrome c1, heme protein, mitochondrial | CYC1 | ENSG00000179091 | 2.0 | 8 | 1 | * | * | * | 30.9 | 25.0 | 0.8 | CYC1 | colorectal cancer | High | 9 | 12 | 75.00 |  |  |  |  |  |  |  |  |  |
| P27797 | Calreticulin | CALR | ENSG00000179218 | 2.0 | 8 | 0 | * | * | * | 106.6 | 107.6 | 1.0 | CALR | colorectal cancer | Low | 9 | 12 | 75.00 |  |  |  |  |  |  |  |  |  |
| P62805 | Histone H4 | HIST4H4 | ENSG00000197837 | 2.0 | 8 | 0 | * | * | * | * | * | * | HIST4H4 | colorectal cancer | High | 7 | 11 | 63.64 |  |  |  |  |  |  |  |  |  |
| P31943 | Heterogeneous nuclear ribonucleoprotein H | HNRNPH1 | ENSG00000169045 | 2.0 | 8 | 0 | * | * | * | 48.0 | 49.4 | 1.0 | HNRNPH1 | colorectal cancer | Medium | 10 | 12 | 83.33 |  |  |  |  |  |  |  |  |  |
| P62304 | Small nuclear ribonucleoprotein E | SNRPE | * | 2.0 | 8 | 1 | * | * | * | 4.4 | 6.2 | 1.4 | * | * | * | * | * | * |  |  |  |  |  |  |  |  |  |
| O60814 | Histone H2B | HIST1H2BK | ENSG00000197903 | 2.0 | 9 | 1 | * | * | * | 1.3 | 343.3 | 257.0 | HIST1H2BK | colorectal cancer | High | 10 | 11 | 90.91 |  |  |  |  |  |  |  |  |  |
| P20962 | Parathymosin | PTMS | ENSG00000159335 | 2.0 | 8 | 0 | * | * | * | * | * | * | PTMS | colorectal cancer | Medium | 6 | 12 | 50.00 |  |  |  |  |  |  |  |  |  |
| Q15436 | Protein transport protein Sec23A | SEC23A | ENSG00000100934 | 2.0 | 8 | 1 | * | * | * | 19.4 | 24.0 | 1.2 | SEC23A | colorectal cancer | Medium | 8 | 12 | 66.67 |  |  |  |  |  |  |  |  |  |
| Q7Z406 | Myosin-14 | MYH14 | ENSG00000105357 | 2.0 | 8 | 0 | * | * | * | 271.9 | 178.2 | 0.7 | MYH14 | colorectal cancer | High | 10 | 12 | 83.33 |  |  |  |  |  |  |  |  |  |
| Q9Y383 | Putative RNA-binding protein Luc7-like 2 | LUC7L2 | ENSG00000146963 | 2.0 | 8 | 0 | * | * | * | 1.5 | 7.9 | 5.2 | LUC7L2 | colorectal cancer | High | 8 | 12 | 66.67 |  |  |  |  |  |  |  |  |  |
| P17858 | ATP-dependent 6-phosphofructokinase, liver type | PFKL | ENSG00000141959 | 2.0 | 7 | 0 | * | * | * | 48.3 | 33.7 | 0.7 | PFKL | colorectal cancer | Not detected | 12 | 12 | 100.00 |  |  |  |  |  |  |  |  |  |
| P49755 | Transmembrane emp24 domain-containing protein 10 | TMED10 | ENSG00000170348 | 2.0 | 9 | 0 | * | * | * | 25.3 | 24.7 | 1.0 | TMED10 | colorectal cancer | High | 6 | 10 | 60.00 |  |  |  |  |  |  |  |  |  |
| P02794 | Ferritin heavy chain | FTH1 | ENSG00000167996 | 2.0 | 7 | 1 | * | * | * | 20.8 | 9.5 | 0.5 | FTH1 | colorectal cancer | Low | 6 | 12 | 50.00 |  |  |  |  |  |  |  |  |  |
| P41250 | Glycine--tRNA ligase | GARS | ENSG00000106105 | 1.9 | 8 | 1 | * | * | * | 17.0 | 26.5 | 1.6 | GARS | colorectal cancer | High | 8 | 12 | 66.67 |  |  |  |  |  |  |  |  |  |
| Q9Y5Z4 | Heme-binding protein 2 | HEBP2 | ENSG00000051620 | 1.9 | 8 | 0 | * | * | * | 17.6 | 8.8 | 0.5 | HEBP2 | colorectal cancer | Medium | 8 | 12 | 66.67 |  |  |  |  |  |  |  |  |  |
| Q08380 | Galectin-3-binding protein | LGALS3BP | ENSG00000108679 | 1.9 | 8 | 1 | * | * | * | 28.6 | 22.5 | 0.8 | LGALS3BP | colorectal cancer | High | 6 | 12 | 50.00 | LGALS3BP | colorectal cancer | Medium | 6 | 12 | 50.00 |  |  |  |
| Q9Y5L4 | Mitochondrial import inner membrane translocase subunit Tim13 | TIMM13 | ENSG00000099800 | 1.9 | 7 | 0 | * | * | * | 12.8 | 7.4 | 0.6 | TIMM13 | colorectal cancer | High | 6 | 11 | 54.55 |  |  |  |  |  |  |  |  |  |
| Q99729 | Heterogeneous nuclear ribonucleoprotein A/B | HNRNPAB | ENSG00000197451 | 1.9 | 8 | 0 | * | * | * | 35.5 | 27.5 | 0.8 | HNRNPAB | colorectal cancer | High | 9 | 9 | 100.00 |  |  |  |  |  |  |  |  |  |
| P62750 | 60S ribosomal protein L23a | RPL23A | ENSG00000198242 | 1.9 | 9 | 0 | * | * | * | 31.0 | 32.8 | 1.1 | RPL23A | colorectal cancer | Not detected | 8 | 11 | 72.73 |  |  |  |  |  |  |  |  |  |
| P62753 | 40S ribosomal protein S6 | RPS6 | ENSG00000137154 | 1.9 | 7 | 0 | * | * | * | 24.6 | 30.1 | 1.2 | RPS6 | colorectal cancer | Medium | 8 | 11 | 72.73 |  |  |  |  |  |  |  |  |  |
| Q9Y2Q3 | Glutathione S-transferase kappa 1 | GSTK1 | ENSG00000197448 | 1.9 | 8 | 2 | * | * | * | 25.6 | 23.0 | 0.9 | GSTK1 | colorectal cancer | High | 8 | 10 | 80.00 |  |  |  |  |  |  |  |  |  |
| P99999 | Cytochrome c | CYCS | ENSG00000172115 | 1.9 | 7 | 0 | * | * | * | 36.4 | 29.3 | 0.8 | CYCS | colorectal cancer | High | 11 | 11 | 100.00 |  |  |  |  |  |  |  |  |  |
| Q15365 | Poly(rC)-binding protein 1 | PCBP1 | ENSG00000169564 | 1.9 | 7 | 0 | * | * | * | 61.9 | 52.8 | 0.9 | PCBP1 | colorectal cancer | High | 10 | 12 | 83.33 |  |  |  |  |  |  |  |  |  |
| Q9ULA0 | Aspartyl aminopeptidase | DNPEP | ENSG00000123992 | 1.9 | 7 | 1 | * | * | * | 19.5 | 6.7 | 0.3 | DNPEP | colorectal cancer | Medium | 8 | 11 | 72.73 |  |  |  |  |  |  |  |  |  |
| P25786 | Proteasome subunit alpha type-1 | PSMA1 | ENSG00000129084 | 1.9 | 7 | 0 | * | * | * | 11.5 | 18.1 | 1.6 | PSMA1 | colorectal cancer | Medium | 11 | 11 | 100.00 |  |  |  |  |  |  |  |  |  |
| P05388 | 60S acidic ribosomal protein P0 | RPLP0 | ENSG00000089157 | 1.9 | 8 | 0 | * | * | * | 40.2 | 45.1 | 1.1 | RPLP0 | colorectal cancer | Medium | 8 | 12 | 66.67 |  |  |  |  |  |  |  |  |  |
| P14866 | Heterogeneous nuclear ribonucleoprotein L | HNRNPL | ENSG00000104824 | 1.9 | 7 | 0 | * | * | * | 7.6 | 53.7 | 7.1 | HNRNPL | colorectal cancer | High | 11 | 11 | 100.00 |  |  |  |  |  |  |  |  |  |
| P50991 | T-complex protein 1 subunit delta | CCT4 | ENSG00000115484 | 1.9 | 7 | 1 | * | * | * | 8.0 | 51.6 | 6.5 | CCT4 | colorectal cancer | Low | 7 | 12 | 58.33 |  |  |  |  |  |  |  |  |  |
| O94760 | N(G),N(G)-dimethylarginine dimethylaminohydrolase 1 | DDAH1 | ENSG00000153904 | 1.9 | 8 | 0 | * | * | * | 34.4 | 17.5 | 0.5 | DDAH1 | colorectal cancer | Medium | 10 | 12 | 83.33 |  |  |  |  |  |  |  |  |  |
| P62826 | GTP-binding nuclear protein Ran | RAN | * | 1.9 | 9 | 0 | * | * | * | 39.7 | 26.5 | 0.7 | * | * | * | * | * | * |  |  |  |  |  |  |  |  |  |
| O75390 | Citrate synthase, mitochondrial | CS | ENSG00000062485 | 1.9 | 7 | 0 | * | * | * | 55.2 | 43.9 | 0.8 | CS | colorectal cancer | High | 8 | 12 | 66.67 |  |  |  |  |  |  |  |  |  |
| P28062 | Proteasome subunit beta type-8 | PSMB8 | ENSG00000204264 | 1.8 | 7 | 1 | * | * | * | 12.8 | 9.9 | 0.8 | PSMB8 | colorectal cancer | High | 7 | 11 | 63.64 |  |  |  |  |  |  |  |  |  |
| P40429 | 60S ribosomal protein L13a | RPL13A | ENSG00000142541 | 1.8 | 7 | 1 | * | * | * | 10.2 | 30.2 | 3.0 | RPL13A | colorectal cancer | Medium | 8 | 12 | 66.67 |  |  |  |  |  |  |  |  |  |
| P63241 | Eukaryotic translation initiation factor 5A-1 | EIF5A | ENSG00000132507 | 1.8 | 7 | 0 | * | * | * | 16.5 | 35.5 | 2.1 | EIF5A | colorectal cancer | Medium | 5 | 10 | 50.00 |  |  |  |  |  |  |  |  |  |
| P67936 | Tropomyosin alpha-4 chain | TPM4 | ENSG00000167460 | 1.8 | 8 | 0 | * | * | * | 62.9 | 51.5 | 0.8 | TPM4 | colorectal cancer | High | 8 | 10 | 80.00 |  |  |  |  |  |  |  |  |  |
| P05386 | 60S acidic ribosomal protein P1 | RPLP1 | ENSG00000137818 | 1.8 | 7 | 0 | * | * | * | 37.0 | 23.8 | 0.6 | RPLP1 | colorectal cancer | Low | 8 | 11 | 72.73 |  |  |  |  |  |  |  |  |  |
| P55854 | Small ubiquitin-related modifier 3 | SUMO3 | ENSG00000184900 | 1.8 | 7 | 0 | * | * | * | * | * | * | SUMO3 | colorectal cancer | Medium | 11 | 12 | 91.67 |  |  |  |  |  |  |  |  |  |
| P52597 | Heterogeneous nuclear ribonucleoprotein F | HNRNPF | ENSG00000169813 | 1.8 | 9 | 0 | * | * | * | 36.8 | 38.1 | 1.0 | HNRNPF | colorectal cancer | Medium | 7 | 9 | 77.78 |  |  |  |  |  |  |  |  |  |
| P13796 | Plastin-2 | LCP1 | ENSG00000136167 | 1.8 | 9 | 0 | * | * | * | 100.3 | 82.7 | 0.8 | LCP1 | colorectal cancer | Not detected | 12 | 12 | 100.00 |  |  |  |  |  |  |  |  |  |
| O15347 | High mobility group protein B3 | HMGB3 | ENSG00000029993 | 1.8 | 7 | 1 | * | * | * | 16.5 | 11.4 | 0.7 | HMGB3 | colorectal cancer | Not detected | 6 | 12 | 50.00 |  |  |  |  |  |  |  |  |  |
| P07858 | Cathepsin B | CTSB | ENSG00000164733 | 1.8 | 8 | 0 | * | * | * | 48.6 | 23.8 | 0.5 | CTSB | colorectal cancer | Medium | 6 | 12 | 50.00 |  |  |  |  |  |  |  |  |  |
| P07900 | Heat shock protein HSP 90-alpha | HSP90AA1 | ENSG00000080824 | 1.8 | 8 | 0 | * | * | * | 185.3 | 232.7 | 1.3 | HSP90AA1 | colorectal cancer | Not detected | 8 | 12 | 66.67 |  |  |  |  |  |  |  |  |  |
| P02788 | Lactotransferrin | LTF | ENSG00000012223 | 1.8 | 8 | 0 | * | * | * | 6.7 | 100.9 | 15.1 | LTF | colorectal cancer | Not detected | 8 | 9 | 88.89 |  |  |  |  |  |  |  |  |  |
| P30101 | Protein disulfide-isomerase A3 | PDIA3 | ENSG00000167004 | 1.8 | 8 | 0 | * | * | * | 211.1 | 177.9 | 0.8 | PDIA3 | colorectal cancer | Medium | 10 | 12 | 83.33 |  |  |  |  |  |  |  |  |  |
| Q7KZF4 | Staphylococcal nuclease domain-containing protein 1 | SND1 | ENSG00000197157 | 1.7 | 8 | 0 | * | * | * | 45.9 | 54.4 | 1.2 | SND1 | colorectal cancer | High | 5 | 12 | 41.67 | SND1 | colorectal cancer | Medium | 5 | 12 | 41.67 |  |  |  |
| P42167 | Lamina-associated polypeptide 2, isoforms beta/gamma | TMPO | ENSG00000120802 | 1.7 | 7 | 0 | * | * | * | 4.5 | 23.6 | 5.3 | TMPO | colorectal cancer | High | 10 | 12 | 83.33 |  |  |  |  |  |  |  |  |  |
| P23396 | 40S ribosomal protein S3 | RPS3 | ENSG00000149273 | 1.7 | 7 | 0 | * | * | * | 56.1 | 61.2 | 1.1 | RPS3 | colorectal cancer | Medium | 9 | 11 | 81.82 |  |  |  |  |  |  |  |  |  |
| Q13510 | Acid ceramidase | ASAH1 | ENSG00000104763 | 1.7 | 8 | 0 | * | * | * | 20.4 | 11.8 | 0.6 | ASAH1 | colorectal cancer | Medium | 7 | 12 | 58.33 |  |  |  |  |  |  |  |  |  |
| Q99832 | T-complex protein 1 subunit eta | CCT7 | ENSG00000135624 | 1.7 | 8 | 0 | * | * | * | 3.4 | 50.8 | 14.8 | CCT7 | colorectal cancer | Medium | 8 | 12 | 66.67 |  |  |  |  |  |  |  |  |  |
| P43490 | Nicotinamide phosphoribosyltransferase | NAMPT | ENSG00000105835 | 1.7 | 7 | 1 | * | * | * | 31.9 | 32.7 | 1.0 | NAMPT | colorectal cancer | Not detected | 9 | 12 | 75.00 |  |  |  |  |  |  |  |  |  |
| P11142 | Heat shock cognate 71 kDa protein | HSPA8 | ENSG00000109971 | 1.7 | 7 | 0 | * | * | * | 65.9 | 184.1 | 2.8 | HSPA8 | colorectal cancer | Medium | 7 | 11 | 63.64 |  |  |  |  |  |  |  |  |  |
| P06703 | Protein S100-A6 | S100A6 | ENSG00000197956 | 1.7 | 7 | 3 | * | * | * | 19.7 | 16.8 | 0.9 | S100A6 | colorectal cancer | Medium | 8 | 11 | 72.73 |  |  |  |  |  |  |  |  |  |
| P62913 | 60S ribosomal protein L11 | RPL11 | ENSG00000142676 | 1.7 | 8 | 0 | * | * | * | 20.9 | 24.8 | 1.2 | RPL11 | colorectal cancer | Medium | 6 | 11 | 54.55 |  |  |  |  |  |  |  |  |  |
| P60866 | 40S ribosomal protein S20 | RPS20 | ENSG00000008988 | 1.7 | 7 | 0 | * | * | * | 16.3 | 17.8 | 1.1 | RPS20 | colorectal cancer | Medium | 10 | 11 | 90.91 |  |  |  |  |  |  |  |  |  |
| Q92945 | Far upstream element-binding protein 2 | KHSRP | ENSG00000088247 | 1.7 | 7 | 0 | * | * | * | 44.9 | 35.0 | 0.8 | KHSRP | colorectal cancer | High | 6 | 12 | 50.00 | KHSRP | colorectal cancer | Medium | 6 | 12 | 50.00 |  |  |  |
| P53621 | Coatomer subunit alpha | COPA | ENSG00000122218 | 1.7 | 7 | 2 | * | * | * | 66.4 | 63.7 | 1.0 | COPA | colorectal cancer | Medium | 9 | 12 | 75.00 |  |  |  |  |  |  |  |  |  |
| P62491 | Ras-related protein Rab-11A | RAB11A | ENSG00000103769 | 1.6 | 7 | 1 | * | * | * | 3.4 | 14.1 | 4.2 | RAB11A | colorectal cancer | Medium | 9 | 12 | 75.00 |  |  |  |  |  |  |  |  |  |
| Q99623 | Prohibitin-2 | PHB2 | ENSG00000215021 | 1.6 | 7 | 0 | * | * | * | 4.5 | 38.3 | 8.5 | PHB2 | colorectal cancer | High | 11 | 11 | 100.00 |  |  |  |  |  |  |  |  |  |
| P30048 | Thioredoxin-dependent peroxide reductase, mitochondrial | PRDX3 | ENSG00000165672 | 1.6 | 7 | 0 | * | * | * | 41.4 | 38.2 | 0.9 | PRDX3 | colorectal cancer | Medium | 10 | 12 | 83.33 |  |  |  |  |  |  |  |  |  |
| P16070 | CD44 antigen | CD44 | ENSG00000026508 | 1.6 | 8 | 0 | * | * | * | 10.7 | 9.8 | 0.9 | CD44 | colorectal cancer | High | 6 | 12 | 50.00 |  |  |  |  |  |  |  |  |  |
| P37802 | Transgelin-2 | TAGLN2 | ENSG00000158710 | 1.6 | 7 | 0 | * | * | * | 118.3 | 119.7 | 1.0 | TAGLN2 | colorectal cancer | Not detected | 10 | 11 | 90.91 |  |  |  |  |  |  |  |  |  |
| P62269 | 40S ribosomal protein S18 | RPS18 | ENSG00000231500 | 1.6 | 7 | 1 | * | * | * | 39.3 | 37.9 | 1.0 | RPS18 | colorectal cancer | High | 9 | 12 | 75.00 |  |  |  |  |  |  |  |  |  |
| P62333 | 26S protease regulatory subunit 10B | PSMC6 | ENSG00000100519 | 1.6 | 7 | 0 | * | * | * | 18.5 | 15.5 | 0.8 | PSMC6 | colorectal cancer | Medium | 9 | 11 | 81.82 |  |  |  |  |  |  |  |  |  |
| P49419 | Alpha-aminoadipic semialdehyde dehydrogenase | ALDH7A1 | ENSG00000164904 | 1.6 | 7 | 2 | * | * | * | 8.2 | 19.5 | 2.4 | ALDH7A1 | colorectal cancer | Not detected | 7 | 12 | 58.33 |  |  |  |  |  |  |  |  |  |
| P35579 | Myosin-9 | MYH9 | ENSG00000100345 | 1.6 | 7 | 0 | * | * | * | 468.5 | 556.3 | 1.2 | MYH9 | colorectal cancer | Medium | 7 | 10 | 70.00 |  |  |  |  |  |  |  |  |  |
| P55011 | Solute carrier family 12 member 2 | SLC12A2 | ENSG00000064651 | 1.6 | 7 | 0 | * | * | * | 26.2 | 37.2 | 1.4 | SLC12A2 | colorectal cancer | High | 11 | 11 | 100.00 |  |  |  |  |  |  |  |  |  |
| P0CG47 | Polyubiquitin | UBB | ENSG00000170315 | 1.5 | 7 | 0 | * | * | * | * | * | * | UBB | colorectal cancer | Medium | 8 | 11 | 72.73 |  |  |  |  |  |  |  |  |  |
| P05455 | Lupus La protein | SSB | ENSG00000138385 | 1.5 | 8 | 0 | * | * | * | 15.0 | 21.8 | 1.5 | SSB | colorectal cancer | High | 9 | 10 | 90.00 |  |  |  |  |  |  |  |  |  |
| P63104 | 14-3-3 protein zeta/delta | YWHAZ | ENSG00000164924 | 1.5 | 7 | 0 | * | * | * | 122.4 | 126.5 | 1.0 | YWHAZ | colorectal cancer | Medium | 7 | 10 | 70.00 |  |  |  |  |  |  |  |  |  |
| P69905 | Hemoglobin subunit alpha | HBA | * | 0.7 | 1 | 7 | * | * | * | * | * | * | * | * | * | * | * | * |  |  |  |  |  |  |  |  |  |
| P68871 | Hemoglobin subunit beta | HBB | ENSG00000244734 | 0.6 | 0 | 8 | * | * | * | 993.5 | 940.4 | 0.9 | HBB | colorectal cancer | Not detected | 7 | 11 | 63.64 |  |  |  |  |  |  |  |  |  |
| P12109 | Collagen alpha-1(VI) chain | COL6A1 | ENSG00000142156 | 0.6 | 0 | 7 | * | * | * | 107.0 | 99.6 | 0.9 | COL6A1 | colorectal cancer | Not detected | 12 | 12 | 100.00 |  |  |  |  |  |  |  |  |  |
| P12111 | Collagen alpha-3(VI) chain | COL6A3 | ENSG00000163359 | 0.6 | 0 | 7 | * | * | * | 408.9 | 374.8 | 0.9 | COL6A3 | colorectal cancer | Not detected | 12 | 12 | 100.00 |  |  |  |  |  |  |  |  |  |
| Q53GG5 | PDZ and LIM domain protein 3 | PDLIM3 | ENSG00000154553 | 0.6 | 0 | 7 | * | * | * | 3.5 | 11.8 | 3.4 | PDLIM3 | colorectal cancer | Not detected | 11 | 12 | 91.67 |  |  |  |  |  |  |  |  |  |
| Q6NZI2 | Polymerase I and transcript release factor | PTRF | ENSG00000177469 | 0.6 | 0 | 9 | * | * | * | 27.5 | 17.2 | 0.6 | PTRF | colorectal cancer | Not detected | 11 | 11 | 100.00 |  |  |  |  |  |  |  |  |  |
| P00352 | Retinal dehydrogenase 1 | ALDH1A1 | ENSG00000165092 | 0.5 | 0 | 8 | * | * | * | 108.7 | 41.9 | 0.4 | ALDH1A1 | colorectal cancer | Not detected | 11 | 12 | 91.67 |  |  |  |  |  |  |  |  |  |
| Q14315 | Filamin-C | FLNC | ENSG00000128591 | 0.5 | 0 | 8 | * | * | * | 38.2 | 88.4 | 2.3 | FLNC | colorectal cancer | Not detected | 9 | 12 | 75.00 |  |  |  |  |  |  |  |  |  |
| O75310 | UDP-glucuronosyltransferase 2B | UGT2B11 | ENSG00000213759 | 0.5 | 0 | 7 | * | * | * | 7.1 | 1.6 | 0.2 | UGT2B11 | colorectal cancer | Not detected | 10 | 12 | 83.33 |  |  |  |  |  |  |  |  |  |
| P04271 | Protein S100-B | S100B | ENSG00000160307 | 0.4 | 0 | 10 | * | * | * | * | * | * | S100B | colorectal cancer | Low | 8 | 11 | 72.73 |  |  |  |  |  |  |  |  |  |
| Q9NR12 | PDZ and LIM domain protein 7 | PDLIM7 | ENSG00000196923 | 0.4 | 0 | 10 | * | * | * | 8.4 | 16.2 | 1.9 | PDLIM7 | colorectal cancer | Not detected | 9 | 12 | 75.00 |  |  |  |  |  |  |  |  |  |
| Q15124 | Phosphoglucomutase-like protein 5 | PGM5 | ENSG00000154330 | 0.4 | 0 | 10 | * | * | * | 23.8 | 13.4 | 0.6 | PGM5 | colorectal cancer | Not detected | 9 | 11 | 81.82 |  |  |  |  |  |  |  |  |  |
| Q13642 | Four and a half LIM domains protein 1 | FHL1 | ENSG00000022267 | 0.4 | 0 | 11 | * | * | * | 13.9 | 15.7 | 1.1 | FHL1 | colorectal cancer | Not detected | 12 | 12 | 100.00 |  |  |  |  |  |  |  |  |  |
| Q9UMS6 | Synaptopodin-2 | SYNPO2 | ENSG00000172403 | 0.3 | 0 | 10 | * | * | * | 2.2 | 15.3 | 6.8 | SYNPO2 | colorectal cancer | Not detected | 11 | 11 | 100.00 |  |  |  |  |  |  |  |  |  |
| P17661 | Desmin | DES | ENSG00000175084 | 0.3 | 0 | 11 | * | * | * | 130.3 | 108.5 | 0.8 | DES | colorectal cancer | Not detected | 11 | 11 | 100.00 |  |  |  |  |  |  |  |  |  |
| P60660 | Myosin light polypeptide 6 | MYL6 | ENSG00000092841 | 0.3 | 0 | 11 | * | * | * | 110.4 | 79.0 | 0.7 | MYL6 | colorectal cancer | Medium | 9 | 10 | 90.00 |  |  |  |  |  |  |  |  |  |
| P09493 | Tropomyosin alpha-1 chain | TPM1 | ENSG00000140416 | 0.3 | 0 | 11 | * | * | * | 64.1 | 59.6 | 0.9 | TPM1 | colorectal cancer | Low | 4 | 10 | 40.00 |  |  |  |  |  |  |  |  |  |
| P63267 | Actin, gamma-enteric smooth muscle | ACTG2 | ENSG00000163017 | 0.2 | 0 | 8 | * | * | * | 724.9 | 780.5 | 1.1 | ACTG2 | colorectal cancer | High | 7 | 12 | 58.33 |  |  |  |  |  |  |  |  |  |
