# Supplementary material for: Quantitative mass spectrometry analysis reveals a panel of nine proteins as diagnostic markers for colon adenocarcinomas
Source: Oncotarget. 2018 Feb 5;9(17):13530–44. doi: 10.18632/oncotarget.24418 (PMC5862596; doi:10.18632/oncotarget.24418)
Supplement: Supplementary file 10 [file oncotarget-09-13530-s010.docx]

| **Supplementary Table 1I: Sample details** | | | | |
| --- | --- | --- | --- | --- |
| Patient ID | Tissue type | Age | Sex | Ethnic Origin |
| 1 | Control | 56 | Female | Morocco |
|  | Tumor |  |  |  |
| 4 | Control | 83 | Female | Unknown |
|  | Tumor |  |  |  |
| 10 | Control | 76 | Female | Russia |
|  | Tumor |  |  |  |
| 17 | Control | 79 | Female | Iraq |
|  | Tumor |  |  |  |
| 20 | Control | 51 | Female | Unknown |
|  | Tumor |  |  |  |
| 3 | Control | 73 | Female | Germany |
|  | Tumor |  |  |  |
| 7 | Control | 67 | Female | Syria |
|  | Tumor |  |  |  |
| 8 | Control | 79 | Female | Syria |
|  | Tumor |  |  |  |
| 9 | Control | 57 | Male | Unknown |
|  | Tumor |  |  |  |
| 11 | Control | 84 | Female | Jordan |
|  | Tumor |  |  |  |
| 14 | Control | 86 | Male | Poland |
|  | Tumor |  |  |  |
